# Cucumber Mosaic Virus Coat Protein Sequesters Host CDPK7‐Like Into Phase‐Separated Condensates to Promote Viral Infection

**DOI:** 10.1111/mpp.70270

**Published:** 2026-05-18

**Authors:** Lei Zhao, Tangbing Yang, Xingjie Zhang, Chunni Zhao, Qingwei Song, Deyu Hu, Lu Yu, Runjiang Song

**Affiliations:** ^1^ State Key Laboratory of Green Pesticide Guizhou University Guiyang China

**Keywords:** agrochemical, biomolecular condensate, coumarin, cucumber mosaic virus, host factor, liquid–liquid phase separation

## Abstract

Plant viruses often manipulate host proteins to facilitate infection, but the molecular mechanisms underlying these interactions remain largely unclear. Here, we provide evidence that the cucumber mosaic virus (CMV) coat protein (CP) interacts with the host kinase CDPK7‐like in *Nicotiana benthamiana*. In an overexpression system, this interaction facilitates liquid–liquid phase separation (LLPS), forming condensate‐like structures that could influence host immune‐related signalling. Strikingly, plants overexpressing the *CDPK7‐like* gene exhibited significantly enhanced resistance against diverse viruses, including potato virus Y and pepper mild mottle virus, yet displayed heightened susceptibility to CMV, suggesting virus‐specific hijacking. Notably, a coumarin‐based small‐molecule LLPS modulator **D3** is developed, which selectively targets the critical Thr52 residue within CMV CP to rescue CDPK7‐like from LLPS initiation. Stands in contrast to the commercial drug ribavirin (EC_50_ = 195 μg/mL), **D3** shows better inactivating property against CMV (EC_50_ = 70.8 μg/mL). This study reveals a mechanism by which CMV manipulates a host factor through phase separation and contributes a promising lead compound **D3** for strategies aimed at enabling plants to win pathogen battles.

## Graphical abstract

This study reveals a tripartite pesticide–pathogen–host interaction in which the host kinase CDPK7‐like in *Nicotiana benthamiana* interacts with cucumber mosaic virus coat protein (CMV CP) to form LLPS‐driven condensates that facilitate viral infection. The antiviral compound **D3** disrupts phase separation by targeting the Thr52 residue of CMV CP.

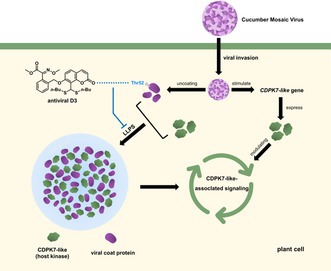

## 1 Introduction

The virus resides within the host's cells and utilises its components to fulfil its own biological processes (Davey et al. 2011; De Monerri and Kim 2014). This hijacking has become fundamental to the viral life cycle. A substantial body of evidence in medical research indicates that biomolecular condensates (BMCs) formed through liquid–liquid phase separation (LLPS) are extensively involved in regulating the pathogenic behaviour of viruses (Li, Ernst, et al. 2022; Zhang et al. 2023; Brocca et al. 2020). Viral functional proteins and their RNA can form condensates, and they can also aggregate with host factors. For instance, the LLPS of dimerised severe acute respiratory syndrome coronavirus 2 (SARS‐CoV‐2) nucleocapsid protein and RNA plays a significant role in evading host innate immune‐driven ubiquitination (Wang et al. 2021). Cellular heat shock proteins, as pro‐viral factors, have been reported to be recruited by several viruses to generate condensates to circumvent the host defence system (Lubkowska et al. 2021; Brown et al. 2005). Given these, the strategic inhibition of these LLPS has emerged as a promising antiviral strategy (Martin et al. 2024). Indeed, there are already numerous modulators capable of countering this process, such as specially designed peptides, bioactive natural products, and antibiotics (Robinson et al. 2021; Zhao et al. 2021; Iserman et al. 2020). Nevertheless, whether such intervention methods can be applied to the protection of agricultural crops remains largely underexplored. Specifically, the design of agrochemicals that can block LLPS in plant viruses may hold promise for the effective management of viral pests.

In addition to participating in plant development, abiotic stress, and interspecies competition, the importance of LLPS‐driven condensates in phytopathology is being elucidated (Huang et al. 2021; May 2024). To date, it has been discovered that components of several phytoviruses can form viral inclusion bodies (IBs) within the host and regulate viral pathogenicity, with the number and size of these bodies often positively correlating with plant disease symptoms, although it is not yet clear if all these aggregates are produced through LLPS (Dolnik et al. 2021; Fang et al. 2019). The turnip mosaic virus viral genome‐linked protein can utilise the host RNA helicase to undergo LLPS, thereby interfering with the formation of nuclear dicing bodies (Li et al. 2021). The barley yellow striate mosaic virus phosphoprotein has been shown to afford droplets with its nucleotides and polymerase, a process that can be inhibited by host casein kinase 1‐mediated phosphorylation (Fang et al. 2022). Moreover, the nucleoprotein and phosphoprotein of the tomato yellow mottle‐associated virus can experience phase separation to produce membrane‐less IBs, which may facilitate viral replication (Liang et al. 2023). Although plants are guarded by innate immune systems against pathogen invasion, the molecular basis by which specific viruses breach or evade such immune surveillance remains less clearly understood. A deeper understanding of this host–pathogen interaction offers promising opportunities for engineered resistance breeding and the development of antiviral agrochemicals.

Cucumber mosaic virus (CMV), a member of the *Bromoviridae* family and the *Cucumovirus* genus, is one of the most widespread and economically significant plant viruses globally, possessing a challenge for chemical control (Jacquemond 2012). CMV is characterised by its tripartite, single‐stranded RNA genome, consisting of RNA1, RNA2, and RNA3, each encoding essential viral proteins (Roossinck 2001). RNA1 and RNA2 encode the 1a and 2a proteins, which form the viral replicase complex, while RNA3 encodes the 3a movement protein (MP) and the coat protein (CP), both of which are critical for viral movement within the host and transmission by insect vectors, primarily (Canto et al. 1997). Recent research has highlighted the role of CMV functional proteins in modulating host factors to create a favourable environment for viral propagation (Tilsner and Kriechbaumer 2022; Cui et al. 2024; Akbarimotlagh et al. 2022). Consequently, a resistive protocol against these virus–host interactions provides the opportunity for antiviral purposes. Here we disclose evidence that the CMV CP co‐opts *Nicotiana benthamiana* calcium‐dependent protein kinase 7‐like (CDPK7‐like) to undergo LLPS, forming droplet‐like BMCs. Surprisingly, transgenic plants overexpressing *CDPK7‐like* exhibited enhanced susceptibility to CMV infection, concomitant with larger droplet formation in vivo, while demonstrating heightened resistance against potato virus Y (PVY) and pepper mild mottle virus (PMMoV) without observable condensates. Notably, we identified compound **D3** as a candidate molecule associated with modulation of the CMV CP–CDPK7‐like condensate‐related process. Our results suggest that **D3** may act on CMV CP at the Thr52 residue and show favourable antiviral activity against CMV compared with ribavirin under our experimental conditions. Collectively, this study provides insights into virus–host interactions and highlights **D3** as a promising lead compound for plant protection.

## 2 Results

### 2.1 Screening for Proteins Interacting With CMV CP

**FIGURE 1:**
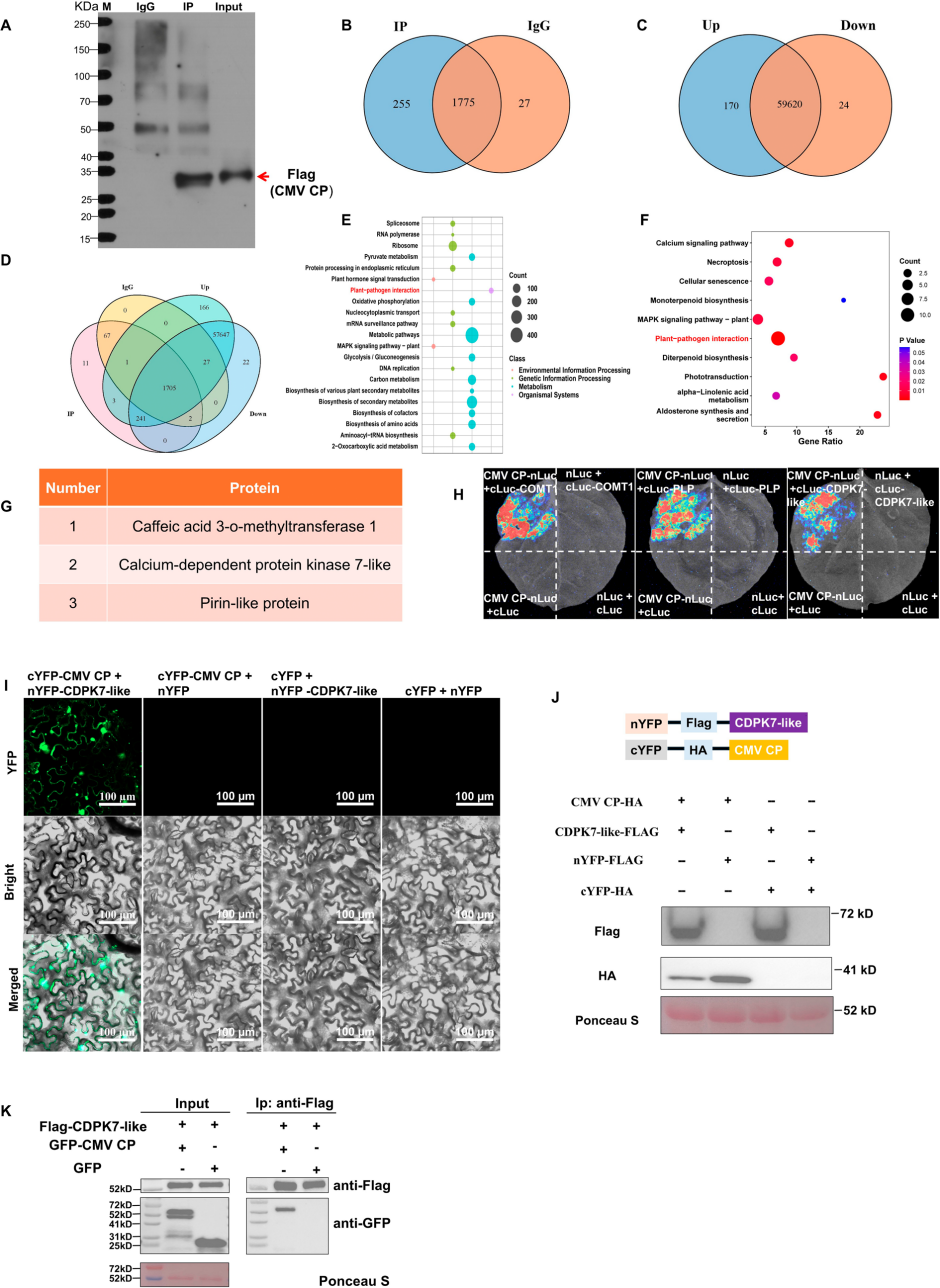
Identification and validation of the host factor CDPK7‐like interacting with cucumber mosaic virus (CMV) coat protein (CP) by co‐immunoprecipitation‐mass spectrometry (Co‐IP‐MS), transcriptomic analysis, luciferase complementation assay (LCA), bimolecular fluorescence complementation (BiFC), and reverse co‐immunoprecipitation (Co‐IP). (A) Immunoblot analysis showing enrichment of Flag‐tagged CMV CP in the IP fraction. Proteins from the IgG control, IP fraction, and input samples were detected using an anti‐Flag antibody. (B) Venn diagram showing the numbers of corresponding *Nicotiana benthamiana* genes identified in the IP and IgG groups from the Co‐IP‐MS analysis. (C) Venn diagram showing the numbers of upregulated and downregulated genes identified by transcriptomic analysis in *N. benthamiana* leaves expressing CMV CP at 3 days post‐infiltration (dpi). (D) Integrative analysis of overlapping genes/candidates from the Co‐IP‐MS and transcriptomic datasets. (E) KEGG pathway enrichment analysis of candidate genes identified from the IP fraction in the Co‐IP‐MS dataset. (F) KEGG pathway enrichment analysis of differentially expressed genes identified in *N. benthamiana* leaves expressing CMV CP. Significant DEGs were defined as log_2_(fold change) ≥ 1 and *p* ≤ 0.05. (G) List of candidate host proteins identified by the integrative analysis as potential CMV CP‐interacting proteins. (H) LCA showing the interactions between CMV CP and the host proteins CDPK7‐like, COMT1, and PLP in *N. benthamiana* leaves. At 60 h after agroinfiltration, the infiltrated leaves were treated with sodium luciferin, and luminescence signals were detected using a chemiluminescence imaging system. (I) BiFC assays showing the interaction between CMV CP and CDPK7‐like in *N. benthamiana* leaves. YFP fluorescence, bright‐field, and merged images are shown. No obvious fluorescence signal was detected in the corresponding empty‐vector control combinations. Scale bars, 100 μm. (J) Expression analysis of the individual fusion components used in the BiFC assays. The expression of CMV CP‐HA and CDPK7‐like‐Flag was detected by immunoblotting using anti‐Flag and anti‐HA antibodies. Ponceau S staining was used as the loading control. (K) Reverse Co‐IP assay confirming the interaction between CDPK7‐like and CMV CP. Flag‐CDPK7‐like was used as the bait protein for immunoprecipitation, and co‐immunoprecipitated GFP‐CMV CP was detected by anti‐GFP immunoblotting. GFP alone was used as the negative control. Ponceau S staining was used as the loading control.

To investigate the host factors potentially associated with CMV infection, we screened proteins interacting with CMV CP. To identify host proteins interacting with CMV CP, *N. benthamiana* leaves were infiltrated with pBWA(V)HS‐flag‐CMV CP, and co‐immunoprecipitation coupled with mass spectrometry (Co‐IP‐MS) was conducted at 3 days post‐infiltration (dpi) (Table S1). FLAG‐tagged CMV CP was enriched in the IP fraction of *N. benthamiana* leaves (Figure 1A). Based on the Co‐IP‐MS results, 2030 and 1802 corresponding *N. benthamiana* genes were identified in the IP and IgG groups, respectively, among which 255 genes were uniquely detected in the IP group (Figure 1B). Kyoto Encyclopedia of Genes and Genomes (KEGG) pathway analysis of these candidate genes revealed enrichment in pathways such as “Plant–pathogen interaction,” “Metabolic pathways,” and “Biosynthesis of secondary metabolites” (Figure 1E). Additionally, transcriptomic analysis was performed by comparing *N. benthamiana* leaves treated with pBWA(V)HS‐flag‐CMV CP and the corresponding empty‐vector control leaves at 3 dpi, identifying 170 upregulated and 24 downregulated differentially expressed genes (DEGs) (Figure 1C; Table S2). KEGG analysis of these DEGs showed enrichment in pathways such as “Plant–pathogen interaction,” “Calcium signaling pathway,” and “Monoterpenoid biosynthesis” (Figure 1F). Next, a comparative analysis of the Co‐IP‐MS and transcriptomic data identified three common genes (Figure 1D), corresponding to three candidate CMV CP‐interacting proteins (Figure 1G), namely *Caffeic acid 3‐O‐methyltransferase 1* (*COMT1*), *CDPK7‐like*, and *Pirin‐like protein* (*PLP*). Among these candidate genes, *CDPK7‐like* was annotated to the “Plant–pathogen interaction” pathway, which was one of the significantly enriched pathways identified by KEGG analysis (Figure 1E,F). CDPK family proteins are widely involved in calcium‐dependent signalling and plant defence‐related responses (Min et al. 2024). Therefore, CDPK7‐like was considered a candidate host factor potentially associated with CMV CP and host defence‐related signalling.

### 2.2 Validation of the Interaction Between CMV CP and CDPK7‐Like Using LCA, BiFC, and Co‐IP

To validate the interactions between CMV CP and host proteins, luciferase complementation assays (LCA) and bimolecular fluorescence complementation (BiFC) were performed. In the LCA (Figure 1H), co‐expression of CMV CP with CDPK7‐like, PLP, or COMT1 produced detectable luminescence in the leaves, indicating direct protein–protein interactions. Consistently, BiFC assays showed that co‐expression of CMV CP with these host proteins reconstituted YFP fluorescence, further confirming their interactions with the wild‐type CP (Figures 1I,J and S1). Notably, the BiFC assay revealed a distinct phenomenon: only the combination of cYFP‐CMV CP and nYFP‐CDPK7‐like produced abundant condensate‐like structures, whereas the other combinations failed to form such BMCs. When expressed individually, GFP‐CMV CP and mCherry‐CDPK7‐like did not show obvious condensate‐like structures, and their fluorescence signals were mainly distributed continuously in the cells (Figure S2A,B). In contrast, co‐expression of GFP‐CMV CP and mCherry‐CDPK7‐like resulted in the appearance of distinct punctate condensate‐like structures with overlapping fluorescence signals (Figure S2C), suggesting that their co‐expression is associated with condensate‐like structure formation. Further subcellular localisation analysis showed that these condensate‐like structures were observed in association with both the nucleus and the plasma membrane (Figure S3).

To further confirm the interaction between the host protein CDPK7‐like and CMV CP, a reverse Co‐IP assay was performed. Flag‐CDPK7‐like was used as the bait protein to immunoprecipitate interacting partners. The results showed that GFP‐CMV CP was successfully co‐immunoprecipitated with Flag‐CDPK7‐like, whereas GFP alone was not detected in the immunoprecipitated fraction (Figure 1K), further demonstrating a specific interaction between CDPK7‐like and CMV CP. In addition, we examined the expression pattern of the host gene *CDPK7‐like* under conditions of CMV CP overexpression and CMV infection. Reverse transcription‐quantitative PCR (RT‐qPCR) analysis showed that the transcript level of *CDPK7‐like* was significantly upregulated in both CMV CP‐overexpressing leaves and CMV‐infected *N. benthamiana* plants (Figure S4), suggesting that CDPK7‐like may participate in the host response to CMV infection. Taken together, these results demonstrate that CMV CP physically interacts with the host protein CDPK7‐like, and that CDPK7‐like expression is significantly induced during CMV infection or CP overexpression. These findings suggest that CDPK7‐like may play an important role in CMV–host interactions and provide a basis for further investigation of its function during CMV infection.

### 2.3 LLPS Drive the Formation of Aggregates

**FIGURE 2:**
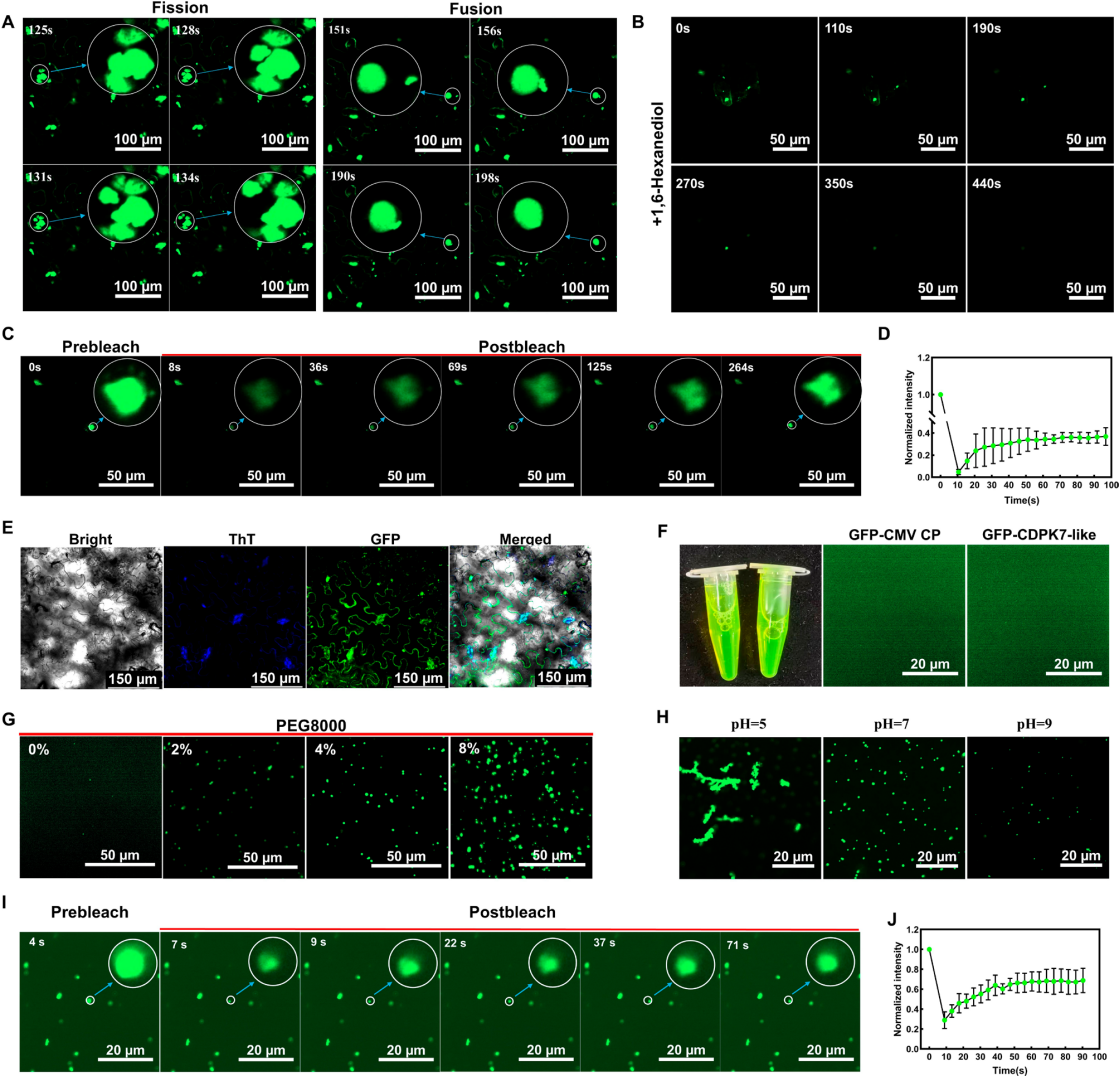
Dynamics and properties of cucumber mosaic virus (CMV) CP–*Nb*CDPK7‐like condensates. (A) Fission and fusion events of condensates observed by confocal microscopy following co‐expression of cYFP‐CMV CP and nYFP‐CDPK7‐like in *Nicotiana benthamiana* leaves at 60 h after agroinfiltration. Scale bars, 100 μm. (B) Representative confocal images showing the effect of 10% 1,6‐hexanediol on CMV CP–CDPK7‐like condensates in planta. *N. benthamiana* leaves co‐expressing cYFP‐CMV CP and nYFP‐CDPK7‐like for 60 h after agroinfiltration were treated with 10% 1,6‐hexanediol, and condensate number and size were reduced within 7 min under our experimental conditions. Dynamic changes are shown in Video S5. Scale bars, 50 μm. (C) Quantification of FRAP recovery of condensates in live cells. Scale bars, 20 μm. (D) Fluorescence recovery curves of live‐cell condensates after photobleaching. (E) ThT staining of CMV CP–CDPK7‐like condensates. Scale bars, 100 μm. (F) Representative confocal microscopy images of purified GFP‐CMV CP (2 mg/mL) and GFP‐CDPK7‐like (2 mg/mL) proteins imaged individually. Scale bars, 20 μm. (G) Effects of different PEG8000 concentrations on condensate formation by mixtures of GFP‐CMV CP (2 mg/mL) and GFP‐CDPK7‐like (2 mg/mL) in vitro. Scale bars, 20 μm. (H) Effects of different pH buffers on condensate formation by mixtures of GFP‐CMV CP (2 mg/mL) and GFP‐CDPK7‐like (2 mg/mL) in vitro. Scale bars, 20 μm. (I) Quantification of FRAP recovery of in vitro–formed condensates composed of purified GFP‐CMV CP (2 mg/mL) and GFP‐CDPK7‐like (2 mg/mL). Scale bars, 20 μm. (J) Fluorescence recovery curves of in vitro condensates after photobleaching.

LLPS has recently been recognised as a critical mechanism underlying the assembly and regulation of various functional membraneless organelles (Hyman et al. 2014). To examine whether the CMV CP‐associated condensates observed in our study were indeed formed via LLPS, we performed a comprehensive series of in vivo and in vitro validation assays. First, transient co‐expression of cYFP‐CMV CP and nYFP‐CDPK7‐like in *N. benthamiana* leaves revealed through dynamic imaging that the resulting condensates exhibited hallmark behaviours of liquid droplets. Under confocal microscopy, we clearly observed spontaneous fission events, in which a larger droplet split into two or more smaller ones, as well as frequent fusion events, where adjacent droplets rapidly coalesced into larger structures (Figure 2A, Videos S1 and S2). These liquid‐like properties are consistent with an LLPS‐associated process. Second, in vivo fluorescence recovery after photobleaching (FRAP) assays showed that the condensates underwent rapid fluorescence recovery within minutes after photobleaching, suggesting that these condensate‐like structures possess dynamic recovery behaviour (Figure 2C,D, Video S3) (Alberti et al. 2019). Such rapid recovery is consistent with the dynamic properties commonly associated with LLPS‐derived condensates. Moreover, at 60 h post‐transient co‐expression of cYFP‐CMV CP and nYFP‐CDPK7‐like in *N. benthamiana* leaves, treatment with 10% 1,6‐hexanediol led to a marked reduction in droplet number and a noticeable shrinkage of condensates within 7 min (Figure 2B, Video S5), suggesting that these condensate‐like structures show a degree of liquid‐like sensitivity under our experimental conditions. To further assess the molecular nature of these condensates, thioflavin T (ThT) staining was performed to evaluate the presence of amyloid‐like β‐sheet structures. ThT is a fluorescent dye that selectively binds to cross‐β sheet–rich amyloid assemblies, producing enhanced fluorescence upon binding (Biancalana and Koide 2010). Following ThT treatment, only a small subset of YFP‐positive structures exhibited detectable ThT‐positive fluorescence within 15 min, whereas the majority of condensate‐like structures remained unstained (Figure 2E). These results indicate that most CMV CP–CDPK7‐like condensates do not exhibit obvious amyloid‐like β‐sheet features and are therefore unlikely to represent classical amyloid‐like aggregates, which is consistent with their LLPS‐like properties.

In vitro reconstitution assays provided additional evidence. Neither GFP‐CMV CP nor GFP‐CDPK7‐like alone was able to produce condensates (Figure 2F); however, when mixed together, the two proteins readily and spontaneously formed numerous droplets. Increasing the PEG8000 concentration from 0% to 8% resulted in a progressive increase in both the number and size of droplets (Figure 2G), indicating a concentration‐dependent phase separation driven by macromolecular crowding. Furthermore, assays performed under different pH buffer conditions revealed that stable and reversible liquid‐like condensates were preferentially formed under near‐neutral pH, where droplet abundance and stability were highest (Figure 2H). In vitro FRAP analysis of these droplets (Figure 2I,J, Video S4) showed significant fluorescence recovery after photobleaching, confirming their dynamic, liquid‐like behaviour. Taken together, these results support the conclusion that CMV CP can recruit the host factor *Nb*CDPK7‐like into condensate‐like structures associated with LLPS. The combined evidence from live‐cell imaging, photobleaching recovery, chemical perturbation, amyloid staining, and in vitro reconstitution suggests that these structures possess dynamic biophysical properties and provides insight into how CMV may exploit host factors during infection.

### 2.4 *CDPK7‐like* Promotes CMV Proliferation in the Host

**FIGURE 3:**
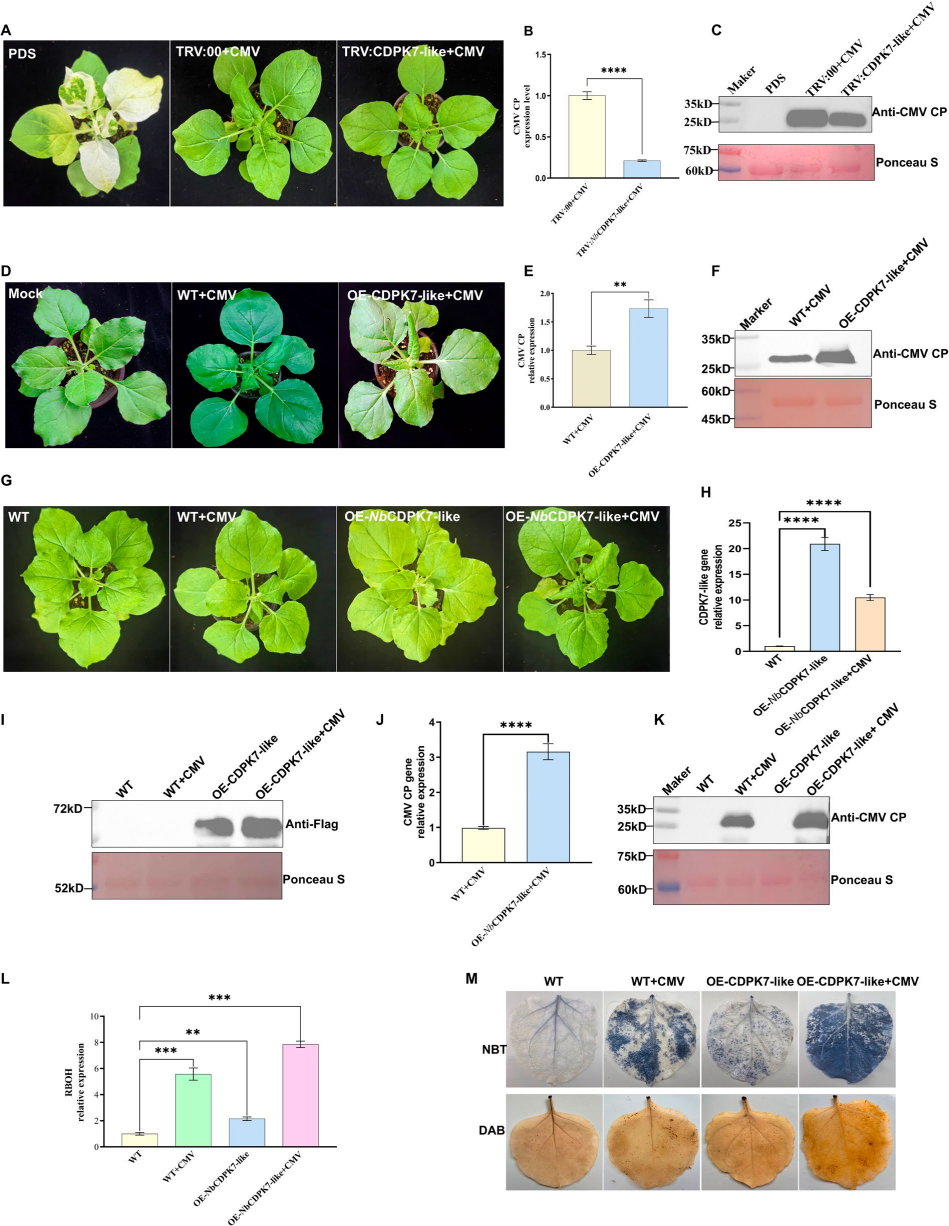
The effect of gene silencing and overexpression of *CDPK7‐like* on cucumber mosaic virus (CMV) proliferation. (A) Plant phenotypes 5 days after CMV inoculation following gene silencing of *CDPK7‐like*. (B) Reverse transcription‐quantitative PCR (RT‐qPCR) analysis of CMV CP transcript levels in the gene‐silencing group. (C) Western blot analysis of the CMV CP accumulation in the gene‐silencing group. (D) Plant phenotypes 5 days after CMV inoculation following overexpression of *CDPK7‐like*. WT, wild‐type. (E) RT‐qPCR analysis of CMV CP transcript levels in the overexpression group. (F) Western blot analysis of the CMV CP accumulation in the overexpression group. (G) Plant phenotypes of *Nicotiana benthamiana* 7 days after CMV inoculation across different treatment groups. (H) RT‐qPCR analysis confirming *NbCDPK7‐like* overexpression in transgenic plants. (I) Western blot analysis verifying CDPK7‐like protein accumulation in overexpression plants. (J) RT‐qPCR analysis of CMV CP transcript levels in different treatment groups. (K) Western blot analysis of CMV CP accumulation in different treatment groups. (L) RT‐qPCR analysis of *RBOH* transcript levels in different treatment groups. (M) Nitroblue tetrazolium (NBT) and 3,3′‐diaminobenzidine (DAB) staining of different treatment groups. Blue precipitates indicate superoxide anion accumulation (NBT staining), while brown precipitates indicate hydrogen peroxide accumulation (DAB staining). Data are presented as mean ± SD from three biological replicates. Statistical differences between two groups were evaluated by Student's *t* test. **p < 0.01, ***p < 0.001, ****p < 0.0001.

To further investigate the role of CDPK7‐like in CMV proliferation in *N. benthamiana*, we conducted gene silencing and overexpression experiments to assess the impact of CDPK7‐like on CMV protein accumulation in the host. In the gene silencing experiment, *N. benthamiana* plants were inoculated with TRV:CDPK7‐like to induce gene silencing. At 9 days after TRV inoculation, RT‐qPCR analysis confirmed that *CDPK7‐like* transcript levels were significantly reduced (Figure S5), and the plants were then inoculated with CMV. Symptoms were recorded for 5 days after CMV inoculation. Compared with the CMV‐infected TRV:00 plants, systemic leaves of CMV‐infected TRV:CDPK7‐like plants showed reduced leaf curling, indicating that silencing of *CDPK7‐like* alleviated CMV symptoms (Figure 3A). Western blot analysis further confirmed a marked reduction of CMV CP accumulation in *CDPK7‐like* silenced plants (Figure 3C). Consistently, RT‐qPCR analysis showed that CMV CP transcript levels were 8.06‐fold higher in TRV:00 control plants infected with CMV than in *CDPK7‐like*–silenced plants infected with CMV (Figure 3B). This suggests that silencing *CDPK7‐like* results in a marked reduction in CMV proliferation in *N. benthamiana*. In the transient overexpression experiment, *N. benthamiana* plants were first agroinfiltrated with pBWA(V)‐CDPK7‐like, followed by CMV inoculation 3 days later, and symptoms were recorded 5 days after CMV inoculation. CMV‐infected plants overexpressing *CDPK7‐like* exhibited more severe leaf curling than the corresponding CMV‐infected control plants (Figure 3D). Western blot analysis revealed higher CMV CP accumulation in CMV‐infected OE‐CDPK7‐like plants (Figure 3F). Consistently, RT‐qPCR showed that CMV CP transcript levels in the OE‐CDPK7‐like group were approximately 1.7‐fold higher than those in the control group (Figure 3E). These results demonstrate that *CDPK7‐like* overexpression promotes CMV proliferation in *N. benthamiana*, and that CMV accumulation is positively correlated with host *CDPK7‐like* expression.

To further investigate the effect of CDPK7‐like overexpression on CMV accumulation, we generated transgenic plants stably overexpressing *CDPK7‐like*. Overexpression was first confirmed by RT‐qPCR and Western blotting (Figures 3H,I and S6A), and the plants were subsequently inoculated with CMV. At 7 days post‐inoculation (dpi), the OE‐*Nb*CDPK7‐like plants infected with CMV exhibited the most severe systemic leaf curling (Figures 3G and S6A). Western blot analysis showed markedly higher CMV CP levels in CMV‐infected OE‐*Nb*CDPK7‐like plants compared with CMV‐infected wild‐type plants (Figure 3K). Consistently, RT‐qPCR analysis revealed that the CMV CP transcript level in CMV‐infected OE‐*Nb*CDPK7‐like plants was approximately 3.16‐fold higher than that in CMV‐infected wild‐type plants (Figure 3J). These findings further demonstrate that stable overexpression of *CDPK7‐like* enhances CMV accumulation in *N. benthamiana*. To assess the specificity of this phenomenon, *CDPK7‐like*‐overexpressing plants were also inoculated with PVY and PMMoV (Figure S6B,C). At 7 dpi, systemic leaves of OE‐*Nb*CDPK7‐like plants infected with PVY displayed markedly weaker green fluorescence compared to wild‐type plants infected with PVY. Western blot analysis showed reduced PVY protein accumulation in the overexpression plants. Consistently, RT‐qPCR analysis revealed that the PVY CP transcript level in PVY‐infected OE‐*Nb*CDPK7‐like plants was approximately 0.46‐fold that in PVY‐infected wild‐type plants (Figure S6B). Similarly, at 9 dpi with PMMoV, OE‐*Nb*CDPK7‐like plants infected with PMMoV exhibited weaker systemic leaf fluorescence compared with PMMoV‐infected WT plants, which was further supported by western blotting analysis. RT‐qPCR further confirmed that PMMoV CP transcript levels in PMMoV‐infected OE‐*Nb*CDPK7‐like plants were about 0.41‐fold of those in PMMoV‐infected WT plants (Figure S6C). Furthermore, BiFC assays (Figure S7) revealed that only CMV CP, but not PVY CP or PMMoV CP, was able to interact with CDPK7‐like and form visible condensates in planta. Collectively, these results indicate that, in contrast to CMV, overexpression of *CDPK7‐like* suppresses the accumulation of PVY and PMMoV. This suggests that CMV may specifically exploit CDPK7‐like to facilitate infection, whereas PVY and PMMoV do not appear to use this host factor in the same way.

Previous results together with transcriptome analysis indicated that *NbCDPK7‐like* was significantly associated with the plant–pathogen interaction pathway, that CMV CP interacts with CDPK7‐like and is associated with its upregulation, and that this correlates with increased expression of the downstream gene *RBOH*. RT‐qPCR analysis further confirmed this observation (Figure 3L): *RBOH* expression in CMV‐infected OE‐*Nb*CDPK7‐like plants was approximately 7.9‐fold higher than that in wild‐type plants, 1.4‐fold higher than that in CMV‐infected wild‐type plants, and 3.6‐fold higher than that in OE‐*Nb*CDPK7‐like plants without CMV infection. Although CMV infection in wild‐type plants was sufficient to induce *RBOH* expression, CMV‐infected OE‐*Nb*CDPK7‐like plants showed enhanced *RBOH* expression, which coincided with increased CMV CP–associated condensate formation. Consistent with this, nitroblue tetrazolium (NBT) and 3,3′‐diaminobenzidine (DAB) staining revealed a marked increase in reactive oxygen species (ROS) accumulation in CMV‐infected OE‐*Nb*CDPK7‐like plants (Figure 3M), in agreement with the observed trend in *RBOH* expression. Together, these results suggest that *CDPK7‐like* is associated with CMV CP‐related condensate formation and enhanced CMV infection, accompanied by altered *RBOH* expression and ROS accumulation.

### 2.5 Synthesis of Coumarin‐Based Derivatives D1–D34

**FIGURE 4:**
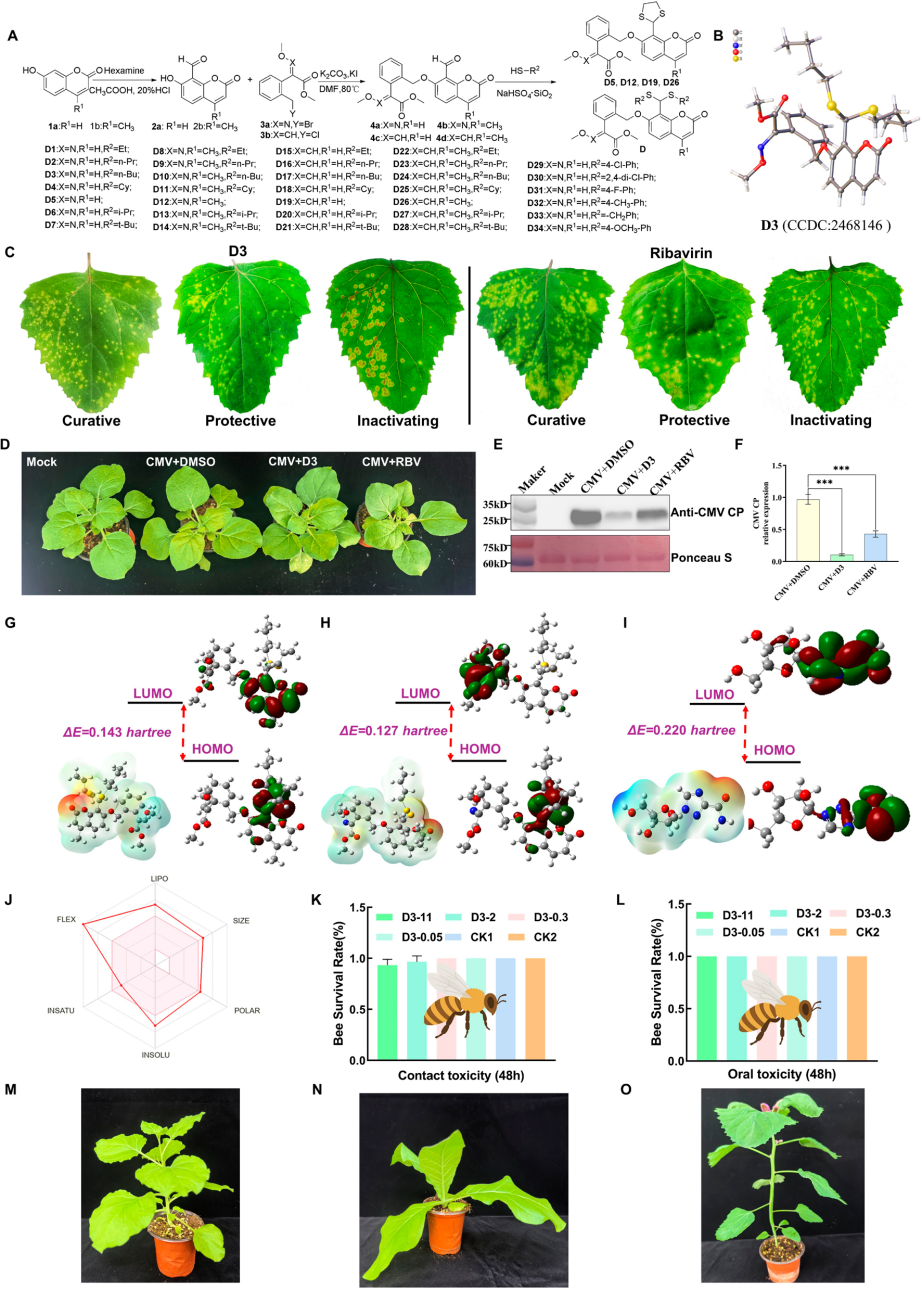
Design, characterisation, antiviral activity, and safety assessment of target compounds. (A) Design and synthesis of target compounds **D1**–**D34**. (B) Single‐crystal structure of **D3**. (C) Curative, protective, and inactivating activities of **D3** and ribavirin against cucumber mosaic virus (CMV) infection in *Chenopodium amaranticolor* leaves. (D) Phenotypes of *Nicotiana benthamiana* plants infected with CMV under different treatments. Plants were treated with dimethyl sulphoxide (DMSO), compound **D3**, or ribavirin. (E) Western blot analysis of CMV coat protein (CP) accumulation in plants under different treatments. (F) Reverse transcription‐quantitative PCR analysis of the relative RNA accumulation levels of CMV CP in plants under different treatments. (G) Molecular LUMO, HOMO, and ESP maps of **D24**. Blue areas in the molecule ESP represent positively charged regions, while red or yellow areas represent negatively charged regions. (H) Molecular LUMO, HOMO, and ESP maps of **D3**. (I) Molecular LUMO, HOMO, and ESP maps of ribavirin. (J) Predicted ADME properties of **D3**. (K) Acute contact toxicity of **D3** to honeybees. (L) Oral toxicity of compound **D3** to honeybees. (M) Growth phenotypes of *N. benthamiana* after treatment with **D3** (500 μg/mL) in the greenhouse for 1 week. (N) Growth phenotypes of *Nicotiana tabacum* ‘K326’ after treatment with **D3** (500 μg/mL) in the greenhouse for 1 week. (O) Growth phenotypes of *C. amaranticolor* after treatment with **D3** (500 μg/mL) treatment in the greenhouse for 1 week.

To identify antiviral compounds against CMV, we designed and synthesised a series of coumarin‐based derivatives by structurally optimizing the coumarin scaffold. The synthetic route (Figure 4A) employed Duff reaction for formylation at position C‐8, followed by nucleophilic substitution with strobilurin‐containing moieties, and concluded with thioacetalisation to generate target compounds **D1**–**D34**. To characterise the prepared molecules, we performed analytical techniques including NMR spectroscopy and HRMS (data available in Supporting Information). Additionally, a crystal structure of selected compound **D3** (CCDC: 2468146) was obtained by X‐ray diffraction analysis, which determined its precise chemical structure (Figure 4B).

### 2.6 Compound D3 Displays Promising Bioactivity Against CMV

Subsequent anti‐CMV evaluation was performed using the half‐leaf local‐lesion method to systematically assess curative, protective, and inactivating activities (Fry and Taylor 1954). As shown in Table S3, the majority of derivatives exhibited moderate to excellent antiviral performance across all three activity modes. Particularly noteworthy were compounds **D3** and **D23**, which demonstrated superior comprehensive efficacy compared to the positive control ribavirin. Specifically, **D3** showed respective activity rates of 58.7% (curative), 61.4% (protective), and 87.3% (inactivating), while **D23** achieved 54.8%, 56.7%, and 86.1% in the same categories, surpassing ribavirin's benchmark values of 41.2%, 43.7%, and 60.5% (Figure 4C). For quantitative comparison of antiviral potency, we further determined EC_50_ values of these compounds based on their inactivating effects. Derivative **D3** emerged as the most potent agent with an inactivation EC_50_ of 70.8 μg/mL, significantly outperforming ribavirin (EC_50_ = 195 μg/mL). To further validate the anti‐CMV activity of compound **D3**, antiviral assays were conducted in *N. benthamiana*. As shown in Figure 4D, compared with the CMV‐infected control plants, **D3** treatment markedly alleviated the systemic leaf curling symptoms caused by CMV infection. In contrast, CMV‐infected plants treated with ribavirin showed only slight symptom relief. Western blot analysis further confirmed this observation (Figure 4E). The accumulation level of CMV CP in CMV‐infected plants treated with **D3** was significantly reduced compared with that in the CMV‐infected control group. Although ribavirin treatment also reduced CP accumulation relative to the CMV‐infected control, the CP level remained noticeably higher than that in the **D3**‐treated group. Consistently, RT‐qPCR analysis showed that CMV CP RNA accumulation in **D3**‐treated CMV‐infected plants was approximately 0.10‐fold of that in CMV‐infected control plants, whereas the ribavirin‐treated CMV infection group exhibited a CP RNA level of about 0.42‐fold of the control (Figure 4F). These results were consistent with both the western blot data and the observed phenotypic changes. According to frontier molecular orbital theory, the distribution and ordering of a molecule's frontier orbitals inform its thermodynamic stability, chemical reactivity, and propensity for target engagement (Hu et al. 2025). The highest occupied molecular orbital (HOMO) typically donates electron density during bond formation, whereas the lowest unoccupied molecular orbital (LUMO) governs electron acceptance and charge‐transfer interactions (Zhang et al. 2022). A smaller HOMO–LUMO gap (ΔE) therefore indicates a lower excitation threshold and, in many ligand–receptor systems, correlates with heightened chemical reactivity and potential bioactivity. To elucidate structure–activity relationships at the quantum‐chemical level, we conducted density functional theory calculations on the fundamental electronic properties of the compounds (Figure 4G–I; Table S4). **D3** exhibited the smallest gap (ΔE = 0.127 Hartree) compared with **D24** (ΔE = 0.143 Hartree), consistent with a greater propensity for electronic interactions within the capsid pocket. Complementary electrostatic potential (ESP) analysis further showed that **D3** presents a more favourable donor–acceptor pattern over the pharmacophore region, supporting stronger electrostatic complementarity to the binding cavity. Taken together, the smaller ΔE and favourable ESP characteristics suggest that **D3** is the most bioactive among the tested compounds, whereas **D24** (and reference ribavirin under comparable protocols) display larger gaps and thus lower intrinsic electronic reactivity toward the target site. By combining the results of the bioassays and computational chemistry analyses, we found that the structural modifications enhanced the antiviral potential of the coumarin scaffold and led to the identification of **D3** as a promising candidate for further evaluation.

### 2.7 Small Molecule D3 Exhibits Favourable ADME Properties and Safety to Non‐Target Organisms

To evaluate the pharmacokinetic performance of compound **D3**, its absorption‐distribution‐metabolism‐excretion (ADME) properties were theoretically evaluated using the SwissADME web tool (http://www.swissadme.ch/index.php). As shown in the radar plot (Figure 4J), **D3** falls well within the optimal physicochemical space for drug‐like molecules in terms of lipophilicity, polarity, size, solubility and flexibility, indicating that it possesses favourable bioavailability and pharmacokinetic properties. To evaluate the ecotoxicological characteristics of compound **D3**, its acute toxicity to honeybees was assessed. After 48 h of oral and contact exposure, mortality remained ≤ 6.7% at the highest tested dose (11 μg a.i./bee) and was absent at lower concentrations (Figure 4K,L; Tables S5 and S6). According to the U.S. Environmental Protection Agency (EPA) guidelines, substances with an LD₅₀ > 11 μg a.i./bee are considered practically non‐toxic; therefore, **D3** can be classified as practically non‐toxic to honeybees, suggesting minimal ecological risk to this non‐target species. We further evaluated the phytotoxicity of **D3** in several plant species. The results showed that treatment of *N. benthamiana*, *Nicotiana tabacum* 'K326 and *Chenopodium amaranticolor* with 500 μg/mL **D3** for 1 week under controlled growth conditions did not induce noticeable leaf shrinkage, corrosion, or striping, particularly in newly emerged leaves (Figure 4M–O). These findings collectively suggest that **D3** possesses favourable ADME‐related properties and a satisfactory biosafety profile, supporting its potential as a promising antiviral lead compound.

### 2.8 Compound D3 Specifically Binds to the T52 Residue on CMV CP

**FIGURE 5:**
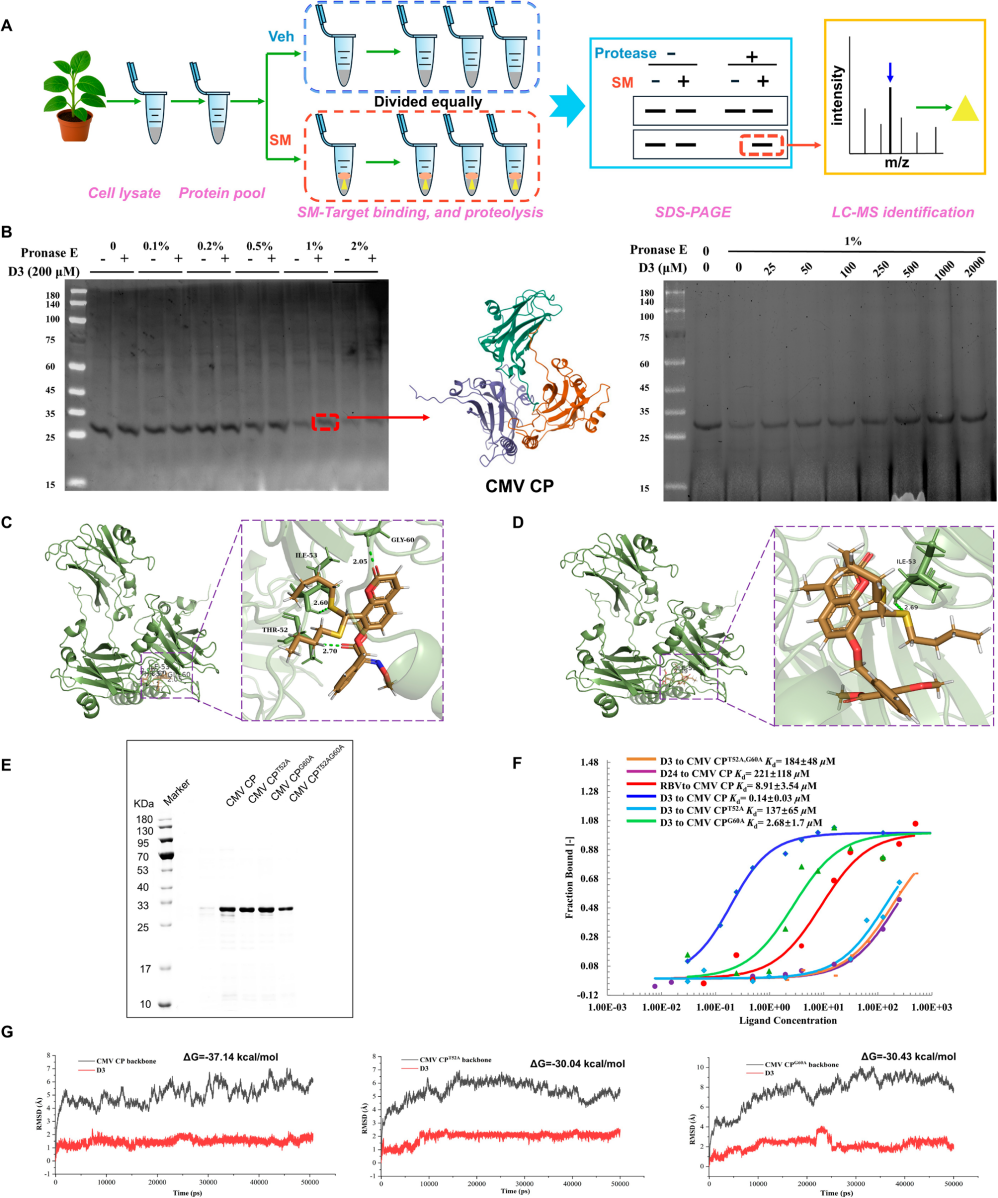
Identification and interaction analysis of small molecule targeting cucumber mosaic virus (CMV) coat protein (CP). (A) Schematic workflow of the DARTS assay. (B) DARTS analysis showing protection of CMV CP by compound **D3** under varying Pronase E concentrations (red arrow indicates protected band). (C) Molecular docking of **D3** with CMV CP. (D) Molecular docking of **D24** with CMV CP. (E) SDS‐PAGE analysis of purified CMV CP^WT^, CMV CP^T52A^, CMV CP^G60A^, and CMV CP^T52AG60A^ proteins. (F) Binding affinities of **D3**, **D24**, and ribavirin for wild‐type and mutant CMV CP proteins. (G) RMSD profiles of **D3** in complex with CMV CP, CMV CP^T52A^, and CMV CP^G60A^ during molecular dynamics (MD) simulation.

To elucidate the molecular target of compound **D3**, we employed the Drug Affinity Responsive Target Stability (DARTS) technique to capture viral target proteins in infected plants. Leaf lysates from CMV‐infected tissues were treated with either **D3** or dimethyl sulphoxide (DMSO) control, followed by partial proteolysis with low concentrations (1%) of Pronase E, and resulting differential protein bands were analysed via SDS‐PAGE (Figure 5A). A distinct, dose‐dependent band enhancement was observed exclusively in **D3**‐treated samples, suggesting specific ligand‐protein stabilisation. The specific band (25–30 kDa) was excised from the gel and subjected to LC–MS/MS analysis, which identified it as the CMV CP (Figure 5B and Table S7). These findings provide evidence that **D3** associates with and stabilises CMV CP against protease degradation.

In order to study the interaction sites of **D3** and CMV CP, molecular docking simulations were performed using Discovery Studio 4.5 software with the crystal structure of CMV CP (PDB ID: 1F15). As a result, **D3** established stable hydrogen bond interactions with three residues in the CP binding pocket: Gly60 (2.05 Å), Ile53 (2.60 Å), and Thr52 (2.70 Å) (Figure 5C). In contrast, **D24**, a derivative exhibiting inferior inactivating activity, demonstrated binding affinity solely with Ile53 (Figure 5D). Hydrogen‐bond interactions are often important contributors to antiviral ligand–target recognition (De Clercq 2023). Thus, after excluding the shared amino acid residue (Ile53), Gly60 and Thr52 were identified as important residues mediating the interaction between **D3** and CMV CP. To validate this, we employed microscale thermophoresis (MST) to investigate how alanine substitutions at these positions affect binding affinity. Wild‐type protein (CMV CP^WT^) along with its mutants CMV CP^T52A^, CMV CP^G60A^, and CMV CP^T52AG60A^ proteins, were successfully expressed in a prokaryotic system and purified for analysis (Figure 5E). The MST results (Figure 5F) revealed a dissociation constant (*K*_d_) of 0.14 μM for **D3** binding to CMV CP^WT^, which was significantly lower than those observed for **D24** (221 μM) and ribavirin (8.91 μM). This aligns with prior viral inactivation results, where stronger CP‐binding affinity correlated with enhanced antiviral activity. Strikingly, mutations at Thr52 profoundly disrupted **D3**–CP interactions: the *K*_d_ values increased to 137 μM for CP^T52A^ and 184 μM for the double mutant CP^T52AG60A^. In contrast, the G60A mutation caused only a modest reduction in affinity (*K*_d_ = 2.68 μM), suggesting that Thr52 plays a dominant role in stabilizing the **D3**–CMV CP complex.

As shown in Figure 5G, the RMSD of the complex gradually increased at the beginning of the molecular dynamics (MD) simulation, indicating that the interaction between **D3** and CMV CP was initially unstable. However, as the simulation progressed, the small molecule penetrated deeper into the protein pocket and formed additional hydrogen bonds, thereby stabilizing the interaction between the two. According to the binding mode obtained from the equilibrated MD simulation, **D3** mainly interacts with Thr52 and Gly60 of CMV CP through hydrogen bonds. To investigate which of these two amino acid residues is more crucial, we subsequently used Modeller to construct CMV CP^T52A^ and CMV CP^G60A^ and performed MD simulations of their binding modes with **D3** using Amber22. Compared with wild‐type CMV CP, both CMV CP^T52A^ and CMV CP^G60A^ exhibited larger fluctuations during the MD simulations. The binding energies of **D3** to CMV CP^T52A^ (−30.04 kcal/mol) and **D3** to CMV CP^G60A^ (−30.43 kcal/mol) were lower than that of **D3** to CMV CP (−37.14 kcal/mol). The weakest binding energy was observed between **D3** and CMV CP^T52A^, suggesting that Thr52 plays a more significant role in stabilizing the interaction. These results indicate that both Thr52 and Gly60 of CMV CP contribute to **D3** binding, with Thr52 likely playing the more important role. This inference was further evaluated in the subsequent infection assays.

### 2.9 Thr52 Is Critical for CMV Infectivity and Mediates the Antiviral Activity of D3

**FIGURE 6:**
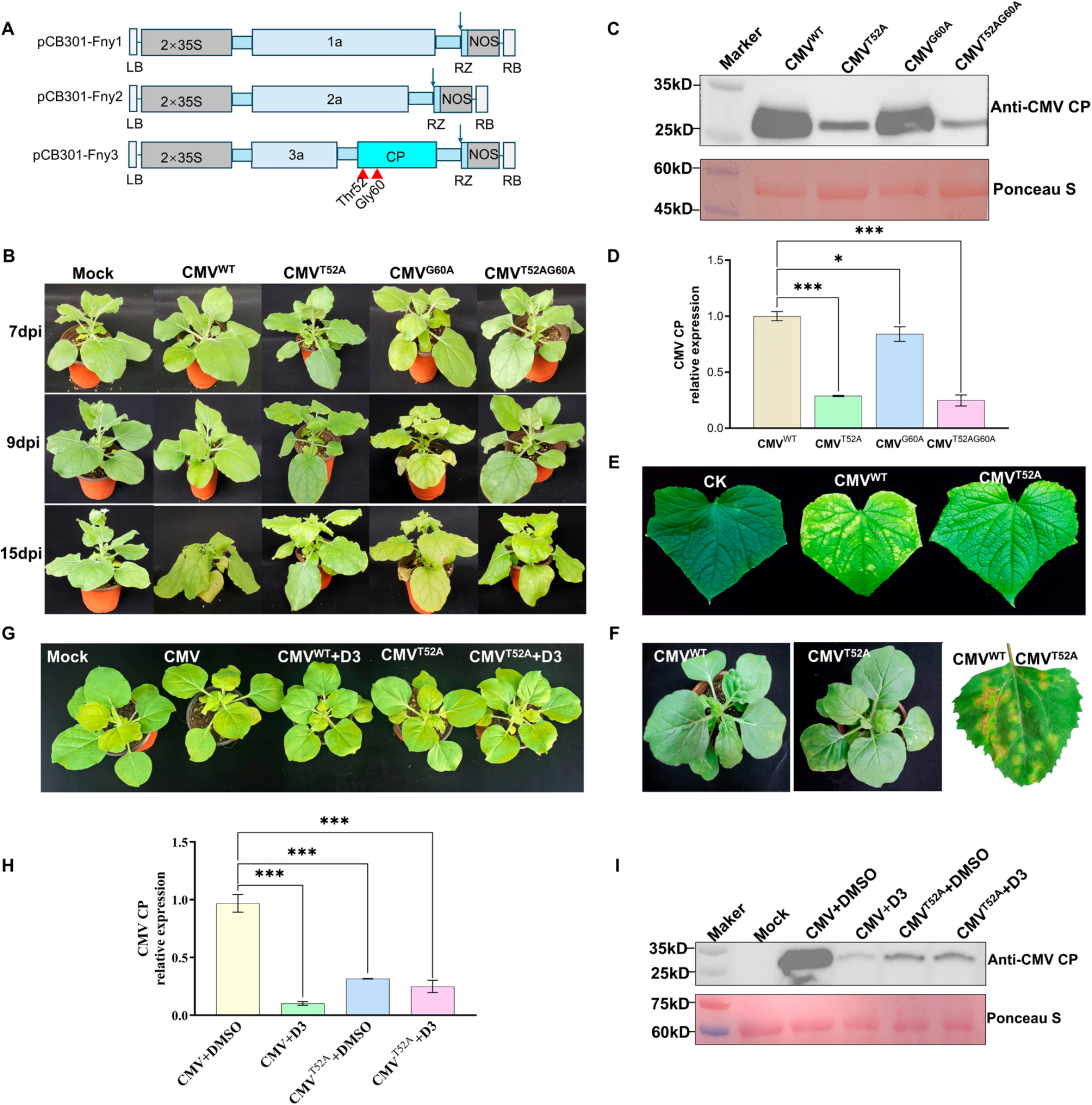
Effects of Thr52 mutation on cucumber mosaic virus (CMV) infectivity and the antiviral activity of compound **D3** in plants. (A) Schematic representation of the genomic structures of pCB301‐Fny1, pCB301‐Fny2, and pCB301‐Fny3 (including mutation sites). (B) Phenotypes of *Nicotiana benthamiana* infected with wild‐type and mutant CMV at 7, 9, and 15 days post‐inoculation (dpi). (C) Western blot analysis of CMV CP accumulation in systemic leaves at 7 days post‐inoculation. (D) Reverse transcription‐quantitative PCR (RT‐qPCR) analysis of viral RNA accumulation in systemic leaves of *N. benthamiana* infected with wild‐type and mutant CMV at 7 days post‐inoculation. (E) Leaf symptoms of cucumber plants infected with wild‐type CMV (WT) or CMV^T52A^ at 1 month post‐inoculation. (F) Phenotypes of *N. benthamiana* and *Chenopodium amaranticolor* infected with WT CMV or CMV^T52A^ at 5 dpi. (G) Phenotypes of *N. benthamiana* plants infected with WT CMV or CMV^T52A^ and treated with **D3**. (H) RT‐qPCR analysis of relative CMV CP RNA levels in plants under different treatments. (I) Western blot analysis of CMV CP accumulation in plants under different treatments. Data are presented as mean ± SD from three biological replicates. Statistical differences between two groups were evaluated by Student's *t* test. ***p < 0.001.

Following the identification of Thr52 as a candidate interaction site in vitro, we further evaluated its role in CMV infectivity in plants. Alanine substitutions were introduced into Thr52 and Gly60 of CMV CP to generate mutants pCB301‐Fny3‐CP^T52A^, pCB301‐Fny3‐CP^G60A^, and pCB301‐Fny3‐CP^T52AG60A^ (Figure 6A). These constructs were agroinfiltrated into *N. benthamiana* to assess viral proliferation through symptom development. As shown in Figure 6B, plants infected with CMV^WT^ or CMV^G60A^ showed severe symptoms, whereas CMV^T52A^ and CMV^T52AG60A^ mutants exhibited attenuated symptoms. This was confirmed by western blotting and RT‐qPCR: CP abundance and viral RNA levels in CMV^T52A^‐infected plants were significantly lower than in CMV^WT^ or CMV^G60A^ infections (Figure 6C,D) (Yao et al. 2011). RT‐qPCR showed that CMV^WT^ had 3.47‐fold higher CP RNA levels than CMV^T52A^, 1.19‐fold higher than CMV^G60A^, and 4.04‐fold higher than CMV^T52AG60A^. To evaluate Thr52's impact on virulence, we performed mechanical inoculation of *N. benthamiana*, *C. amaranticolor* , and cucumber plants with CMV or CMV‐CP^T52A^. In cucumber, CMV^WT^‐infected plants developed severe mosaic symptoms, whereas CMV^T52A^ infection resulted in less discolouration (Figure 6E). In *N. benthamiana*, CMV^WT^ caused severe apical curling, which was substantially reduced in CMV‐CP^T52A^ plants (Figure 6F). *C. amaranticolor* showed more necrotic lesions in CMV^WT^ infections compared to CMV^T52A^ (Figure 6F). Next, we treated CMV‐ and CMV^T52A^‐infected plants with **D3**. **D3** significantly alleviated symptoms in CMV‐infected plants (Figure 6G). However, **D3** treatment did not noticeably reduce symptoms in CMV^T52A^‐infected plants, suggesting that the antiviral effect of **D3** is closely associated with Thr52 (Figure 6G). RT‐qPCR showed a 0.10 relative accumulation of CMV CP RNA in **D3**‐treated CMV‐infected plants compared to untreated controls, but no significant difference in CMV^T52A^‐infected plants (Figure 6H). Western blotting analysis confirmed that **D3** reduced CMV CP accumulation in CMV‐infected plants, while no significant difference was observed in CMV^T52A^‐infected plants (Figure 6I). In conclusion, Thr52 is an important residue for CMV infectivity and pathogenicity. Mutation of Thr52 significantly reduced viral replication and symptoms across multiple hosts. Furthermore, the antiviral effect of **D3** was markedly reduced in the T52A mutant background, supporting an important role for Thr52 in both CMV infection and the antiviral action of **D3**.

### 2.10 The Residue Thr52 Affects the Interaction Between CMV CP and CDPK7‐Like and Is Associated With Condensate Formation

**FIGURE 7:**
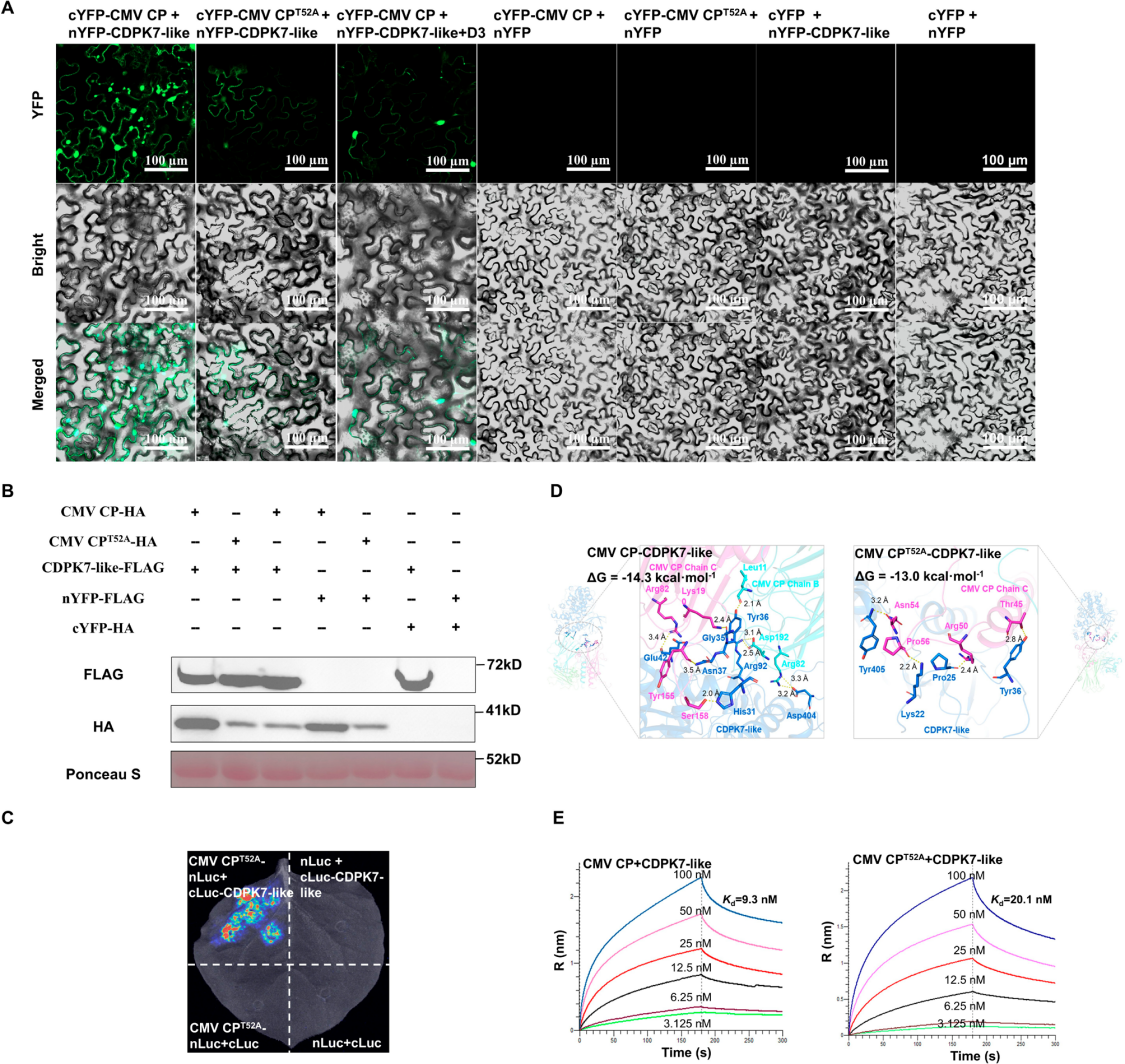
Effects of Thr52 mutation and **D3** treatment on the interaction between cucumber mosaic virus (CMV) coat protein (CP) and CDPK7‐like and on condensate formation. (A) Bimolecular fluorescence complementation (BiFC) analysis of the interactions of CMV CP and CMV CP^T52A^ with CDPK7‐like in *Nicotiana benthamiana* leaves. *Agrobacterium* strains carrying the indicated constructs were co‐infiltrated into *N. benthamiana* leaves, and YFP fluorescence was observed by confocal microscopy at 60 h after agroinfiltration. The **D3**‐treated sample and the corresponding negative control combinations are also shown. YFP, bright‐field, and merged images are presented. Scale bars, 100 μm. (B) Expression analysis of the individual fusion components used in the BiFC assays. The expression of CMV CP‐HA, CMV CP^T52A^‐HA, and CDPK7‐like‐FLAG was detected by immunoblotting using anti‐FLAG and anti‐HA antibodies. Ponceau S staining was used as the loading control. (C) Luciferase complementation analysis (LCA) of the interaction between CMV CP^T52A^ and CDPK7‐like. *Agrobacterium* strains carrying the indicated constructs were co‐infiltrated into *N. benthamiana* leaves. At 60 h after agroinfiltration, the infiltrated leaves were treated with sodium luciferin and luminescence signals were detected using a chemiluminescence imaging system. (D) Molecular docking analysis of CDPK7‐like with wild‐type CMV CP or the CMV CP^T52A^ mutant. Predicted binding modes and binding energies are shown. (E) Biolayer interferometry (BLI) analysis of the binding of CDPK7‐like to CMV CP and CMV CP^T52A^. Binding curves and the calculated dissociation constants (*K*_d_) are shown.

Our experimental data showed that the interaction between CMV CP and CDPK7‐like promotes the formation of BMCs. To determine the role of residue Thr52 in this interaction, LCA and BiFC assays demonstrated that the CMV CP^T52A^ mutant retained the ability to interact with CDPK7‐like (Figures 7A–C and S8). However, unlike the wild‐type protein, CMV CP^T52A^ failed to form BMCs with CDPK7‐like in *N. benthamiana* leaf cells (Figure 7A). Moreover, treatment of plants co‐expressing cYFP‐CMV CP and nYFP‐CDPK7‐like with compound **D3** markedly reduced the abundance of condensates (Figure 7A). These findings suggest that the interaction between CMV CP and CDPK7‐like contributes to BMC formation, and that either compound **D3** treatment or the T52A mutation effectively disrupts this process. To further investigate the role of Thr52 in BMC formation, molecular docking was performed using the ZDOCK online server (https://zdock.wenglab.org/) to assess the interactions between CDPK7‐like and either wild‐type or mutant CMV CP. Docking results (Figure 7D) indicated that CDPK7‐like binds to both the B and C chains of wild‐type CMV CP with a binding energy of −14.3 kcal mol^−1^, whereas it binds only to the C chain of CMV CP^T52A^ with weaker affinity (−13.0 kcal mol^−1^). This difference may account for their distinct capacities to form condensates. Consistently, Octet biolayer interferometry confirmed that CMV CP exhibited a stronger binding affinity to CDPK7‐like (*K*_d_ = 9.3 nM) compared with CMV CP^T52A^ (*K*_d_ = 20.1 nM) (Figure 7E), which correlated well with the predicted docking energies. Taken together, these results suggest that residue Thr52 of CMV CP may regulate its interaction with CDPK7‐like by altering the protein's conformation.

## 3 Discussion

**FIGURE 8:**
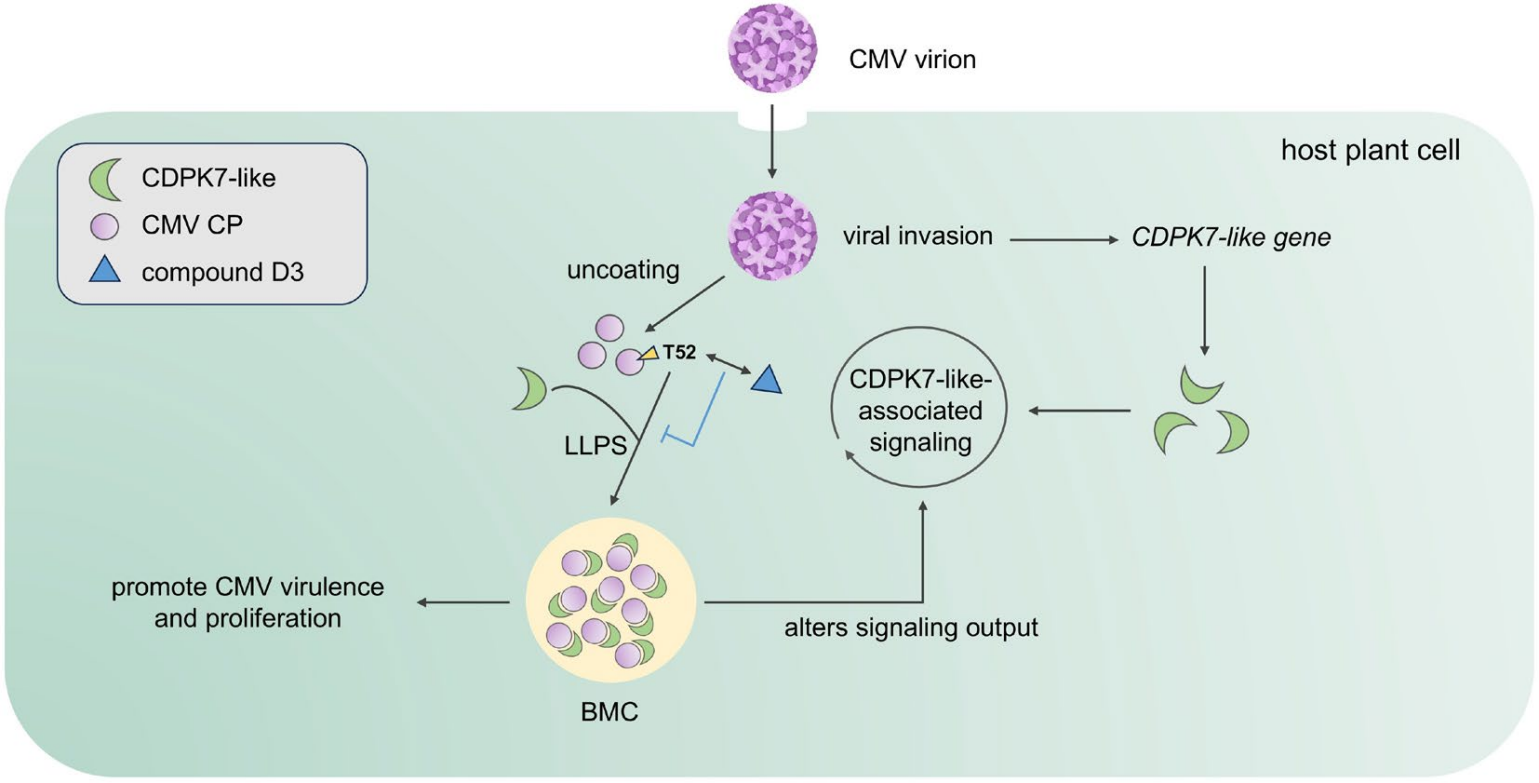
Proposed mechanistic model of compound D3 inhibiting cucumber mosaic virus (CMV) infection. Compound **D3** binds the Thr52‐associated region of CMV coat protein (CP), disrupts the CP–CDPK7‐like interaction and associated condensate‐like structure formation, and ultimately reduces CMV proliferation in the host.

Viruses have evolved diverse mechanisms to modulate host processes during infection, among which BMCs formed through the co‐assembly of viral proteins and host factors have emerged as an important phenomenon (Li, Ernst, et al. 2022). Notably, many, though not all, of these aggregates have been reported to be induced via phase separation processes (Liang et al. 2023; Lin and Nagy 2024). For example, the p27 protein of red clover necrotic mosaic virus (RCNMV) localises host *Nb*RBOHHB and *Nb*CDPKiso2 to intracellular aggregates to divert ROS‐generating machinery (Hyodo et al. 2017). Hyodo et al. demonstrated that *Nb*RACK1 is recruited to endoplasmic reticulum‐derived aggregates by p27, where it mediates ROS accumulation, providing a prerequisite for RCNMV replication (Hyodo et al. 2019). These cytoplasmic inclusions have been proposed to influence host responses during infection, which is broadly consistent with our findings. Recently, Zeng et al. showed that the P6 protein of southern rice black‐streaked dwarf virus forms stress granules with OsTSN1 through LLPS, mediating viral evasion of autophagy‐related defence pathways (Zeng et al. 2025). Our findings suggest that CMV CP associates with CDPK7‐like to form condensate‐like structures in an overexpression system, providing a possible basis for how CMV may manipulate host factors during infection. Although CDPK7‐like may participate in host defence‐related signalling during viral infection, the formation of these condensate‐like structures may alter its functional output and be associated with enhanced CMV infection and disease development. Furthermore, the Thr52 residue of CP likely plays an important role in this interaction and the associated condensate formation. Notably, we identified the coumarin derivative **D3**, which likely acts on CMV CP at this residue and exhibits clear antiviral activity against CMV (Figure 8). In particular, because the current FRAP analysis was performed in a BiFC‐based system, the fluorescence recovery observed here cannot distinguish between molecular exchange of pre‐existing complexes and local reconstitution of BiFC signals. Nevertheless, further studies using FLIM‐FRET, FCCS, or non‐BiFC‐based FRAP approaches will be needed to more directly characterise the in vivo interaction and dynamic behaviour of CMV CP–CDPK7‐like condensate‐like structures. It should also be noted that the condensate‐like structures described here were primarily observed in transient overexpression systems, and comparable structures have not yet been directly visualised under natural CMV infection conditions. In addition, although our data support CMV CP as a major target associated with the antiviral effect of **D3**, the in planta distribution of **D3** and the possible contribution of additional viral or host targets remain to be further investigated.

CMV stands as one of the most economically significant plant pathogens due to its broad host range, affecting over 1200 plant species, including key agricultural crops such as cucumbers, tomatoes, peppers, and legumes (Palukaitis and García‐Arenal 2003). First described in the early 20th century, CMV remains a pervasive threat to global food security, causing symptoms ranging from mosaic patterning and leaf distortion to severe stunting, necrosis, and yield losses exceeding 80% in susceptible cultivars (Roossinck 2001). Its capacity for rapid evolution, coupled with efficient transmission by over 80 aphid species in a non‐persistent manner, underscores its adaptability and challenges in disease management (Ng and Falk 2006).

CMV encodes multiple proteins involved in replication, movement, and pathogenesis, among which CP plays central roles in virion assembly, systemic movement, vector transmission, and host interactions. This multifunctional protein not only encapsulates the viral genome but also acts as a key determinant of CMV's ecological success and evolutionary adaptability. The CP is a 25–26 kDa protein that forms the icosahedral capsid, protecting the viral RNA from environmental degradation and host ribonuclease activity (El‐Borollosy and Waziri 2013). A defining feature of the CP is its role in aphid transmission. CMV is transmitted non‐persistently by over 80 aphid species, relying on the CP to bind directly to receptors in the aphid's stylet (Ng and Falk 2006). This interaction is facilitated by a conserved domain in the CP, which may coordinate with calcium ions to stabilise virion attachment (Smith et al. 2000). Mutations in these residues abolish transmission without affecting virion stability, underscoring the CP's specificity in vector interactions (Liu et al. 2002). Moreover, the CP enhances viral uptake into plant cells by working synergistically with the MP to mediate cell‐to‐cell movement (Delormel and Boudsocq 2019; Taliansky and Garcia‐Arenal 1995; Kaplan et al. 1998). Recent studies have demonstrated that CMV CP induces chlorotic symptoms in tobacco through interaction with host chloroplast ferredoxin I (Qiu et al. 2018). Given the functional diversity and importance of CP in maintaining viral particle stability, mediating vector–virus interactions, and facilitating virus–host interactions, targeting the inhibition of CP functions has emerged as a viable strategy for treating CMV.

In recent years, numerous small molecular chemicals targeting various plant viral CPs have been successively reported, most of which were obtained through optimisation of active natural products (Wu et al. 2022; Zan et al. 2021). The *cis*‐*trans* isomeric pair of indol‐3‐yl morpholino compounds was found to bind specifically to tobacco mosaic virus (TMV) CP and PVY CP, effectively inhibiting virion assembly (Sun et al. 2024). Several sulfonamide‐containing chromone derivatives demonstrated inhibitory effects on the CP of tomato chlorosis virus (Jiang et al. 2021). Dimers of ferulic acid exhibited antiviral activity through direct interaction with TMV particles, leading to viral inactivation (Tai et al. 2024). However, despite frequent observations of deformed or reduced virions via transmission electron microscopy and decreased viral accumulation measured by RT‐qPCR, the precise molecular mechanisms underlying these antiviral effects remain inadequately characterised. Specifically, the functional roles of amino acid residues targeted by these compounds on viral CPs require further elucidation. Structural characterisation of viral capsid proteins provides critical insights into potential modes of drug action (Kežar et al. 2019). The *H*‐chromene derivative C50 and a benzo[*b*]thiophene‐based analog C54 (derived from coffee bean constituents) were shown to inhibit viral RNA binding through specific interactions with Ser125 and Arg157 residues in PVY CP, respectively (Shi et al. 2025). Nevertheless, the complex tripartite interactions involving “agrochemicals–pathogens–hosts” remain poorly understood. A dithioacetal‐containing vanillin derivative not only targets Thr92 and Arg94 within the intrinsically disordered region of tomato spotted wilt virus nucleocapsid protein to prevent pathogenic aggregation, but also suppresses expression of the host factor GTP‐binding nuclear protein Ran‐like that promotes N condensation (Wang, Luo, et al. 2025). A recent study revealed that chiral benzimidazole derivatives competitively bind to Arg191 of PVY CP—a critical residue mediating interaction with host NtCPIP that facilitates cell‐to‐cell movement of viral particles (Wei et al. 2024).

Coumarin is a naturally occurring aromatic compound characterised by a benzopyrone structure and diverse bioactivities, serving as a privileged scaffold in drug discovery (Fry and Taylor 1954). Coumarin analogs have shown exciting antiviral activity against several human viral pathogens (Enterovirus 71, HIV, chikungunya virus, etc.) (Mishra et al. 2020; Jesumoroti et al. 2019; Li, Kong, et al. 2022). Mechanistically, certain derivatives target viral proteases or polymerases, critical for viral genome replication (Nichols et al. 2013). In addition, coumarin‐based compounds have shown potential in blocking the interaction between SARS‐CoV‐2 spike proteins and host ACE2 receptors (Singh et al. 2022). Our previous investigations revealed that coumarin derivatives modified with dithioacetal moieties exhibit targeted activity against phytoviral CPs (De Clercq 2023). Despite this, the mechanistic basis for how these interactions suppress viral pathogenicity, particularly whether they modulate dynamic virus–host interplay, remains unexplored.

In summary, our study demonstrates that the host kinase CDPK7‐like interacts with the CMV CP and is associated with the formation of condensate‐like structures with LLPS‐related properties. These findings suggest that CMV may engage host factors to modulate cellular processes during infection. Our results further indicate that the Thr52 residue of CMV CP plays an important role in this interaction and the associated condensate formation. In addition, we identified a coumarin‐derived small molecule, **D3**, whose antiviral effect is closely associated with the Thr52 residue and with interference in the CP–CDPK7‐like condensate‐related process. Together, these findings provide new insights into virus–host interactions and may contribute to the development of antiviral agrochemicals.

## 4 Experimental Procedures

### 4.1 Materials and Virus Sources

Seeds of *N. benthamiana* and *C. amaranticolor* were preserved in our laboratory prior to use. Plants were grown under controlled conditions in a growth chamber, with daytime settings of 25°C and 60% relative humidity for 16 h, followed by nighttime conditions of 16°C and 50% relative humidity for 8 h. The CMV infectious clone vector pCB301‐CMV, harbouring the complete viral genome, together with CMV virus materials, was generously provided by Prof. Xianbing Wang (China Agricultural University) to support plasmid construction.

### 4.2 Co‐Immunoprecipitation Mass Spectrometry

The coding sequence of CMV CP was amplified from the corresponding plasmid and cloned into the pBWA(V)HS‐Flag vector to generate the expression construct pBWA(V)HS‐Flag‐CMV CP. The resulting plasmid was transformed into *Agrobacterium tumefaciens* GV3101 and cultured overnight at 28°C with shaking. Bacterial cells were harvested by centrifugation and resuspended in infiltration buffer containing 10 mM MES (pH 5.6), 10 mM MgCl_2_, and 100 μM acetosyringone. The bacterial suspension was adjusted to an OD₆₀₀ of 1.0 and incubated at 28°C for 2 h before infiltration into *N. benthamiana* leaves.

Approximately 3 days post‐infiltration, leaf tissues were harvested, immediately frozen in liquid nitrogen, and ground into a fine powder. The samples were extracted with ice‐cold protein extraction buffer (50 mM Tris–HCl pH 7.4, 150 mM NaCl, 1% NP‐40, 1 mM EDTA, 1 mM PMSF, and a protease inhibitor cocktail), followed by gentle agitation at 4°C. The extracts were clarified by centrifugation at 12,000 *g* for 15 min at 4°C, and the supernatants were collected for Co‐IP assays.

For immunoprecipitation, cleared lysates were incubated with anti‐Flag magnetic beads at 4°C with gentle rotation for 4–6 h to enrich Flag‐CMV CP and its interacting proteins (IP group). In parallel, an IgG negative control group was included, in which equal amounts of lysate were incubated with non‐specific IgG‐conjugated magnetic beads under the same conditions to account for nonspecific binding. After incubation, the beads were collected using a magnetic separator and washed multiple times with wash buffer to remove nonspecifically bound proteins. Finally, the proteins bound to the beads were eluted and subjected to mass spectrometry analysis (IP‐MS) to identify host proteins specifically interacting with CMV CP.

### 4.3 Transcriptomic Experiment

The pBWA(V)HS‐Flag‐CMV CP expression construct and the corresponding empty‐vector control were transformed into *A. tumefaciens* GV3101 and cultured overnight at 28°C. Bacterial cells were collected by centrifugation, resuspended in infiltration buffer containing 10 mM MES, 10 mM MgCl_2_, and 100 μM acetosyringone, and adjusted to an OD_600_ of 1.0. After incubation at 28°C for 2 h, the suspensions were agroinfiltrated into *N. benthamiana* leaves. At approximately 60 h post‐infiltration, CMV CP‐expressing leaves and the corresponding empty‐vector control leaves were collected for comparative transcriptomic analysis.

### 4.4 LCA Experiment

Using the empty vectors available in our laboratory (Wang, Yang, et al. 2025), the coding sequences of PLP, COMT1, and CDPK7‐like were amplified from *N. benthamiana* cDNA and cloned into the JW772‐cLuc vector by homologous recombination, generating JW772‐cLuc‐PLP, JW772‐cLuc‐COMT1, and JW772‐cLuc‐CDPK7‐like. The coding sequences of CMV CP and CMV CP^T52A^ were cloned into JW771‐nLuc by homologous recombination, generating JW771‐CMV CP‐nLuc and JW771‐CMV CP^T52A^‐nLuc, respectively. *Agrobacterium* strains carrying the indicated constructs were individually adjusted to an OD_600_ of 1.0. For each assay, one *Agrobacterium* strain carrying an nLuc fusion construct and one strain carrying a cLuc fusion construct were mixed pairwise in equal volumes for co‐infiltration into *N. benthamiana* leaves. At 60 h after agroinfiltration, the infiltrated leaves were soaked in 1 mM sodium luciferin for 10 min. Luminescence signals were detected using a chemiluminescence imaging system, and the images were processed using Image Lab and Adobe Photoshop.

### 4.5 BiFC Experiment

Using the empty vectors available in our laboratory, the coding sequences of PLP, COMT1, and CDPK7‐like were amplified from *N. benthamiana* cDNA, while the coding sequences of CMV CP and CMV CP^T52A^ were amplified from the corresponding plasmids. These sequences were cloned into the BiFC vectors by homologous recombination (Li, Feng, et al. 2022), generating p1300‐CMV CP‐cYFP‐HA, p1300‐CMV CP^T52A^‐cYFP‐HA, and p1300‐CDPK7‐like‐nYFP‐Flag. The cYFP constructs carried an HA tag, whereas the nYFP constructs carried a FLAG tag. *Agrobacterium* strains carrying the indicated constructs were individually adjusted to an OD_600_ of 1.0. For each assay, one *Agrobacterium* strain carrying a cYFP fusion construct (or empty cYFP control) and one strain carrying an nYFP fusion construct (or empty nYFP control) were mixed pairwise in equal volumes for co‐infiltration into *N. benthamiana* leaves. At approximately 60 h after agroinfiltration, infiltrated leaves were collected, mounted in distilled water on glass slides, and observed using a Zeiss laser scanning confocal microscope. The p1300‐PLP‐nYFP‐Flag, p1300‐COMT1‐nYFP‐Flag, p1300‐PVY CP‐cYFP‐HA, and p1300‐PMMoV CP‐cYFP‐HA constructs were generated using the same cloning strategy, and the corresponding BiFC assays were performed as described above.

### 4.6 Virus‐Induced Gene Silencing Assays

*Agrobacterium* strains carrying pTRV2‐CDPK7‐like, pTRV2‐PDS, or pTRV2 were mixed with pTRV1 in equal volumes and adjusted to the same OD_600_ (OD_600_ = 0.5) before co‐infiltration into *N. benthamiana* plants at the 3–5 leaf stage. When the positive control (TRV:PDS) exhibited the characteristic bleaching phenotype at 9 dpi, RT‐qPCR was performed to evaluate the silencing efficiency of *CDPK7‐like*. The successfully silenced plants were then inoculated with CMV. At 5 dpi after CMV inoculation, symptoms were recorded, and 0.1 g leaf samples were collected for western blot and RT‐qPCR analyses to determine CMV CP accumulation and viral RNA levels, respectively.

### 4.7 Transient Overexpression of *CDPK7‐Like* Experiment

The CDPK7‐like coding sequence was amplified from *N. benthamiana* cDNA and cloned into the pBWA(V)HS vector by homologous recombination, generating pBWA(V)HS‐CDPK7‐like. The resulting construct was transformed into *A. tumefaciens* GV3101 and cultured overnight at 28°C. Bacterial cells were collected by centrifugation, resuspended in infiltration buffer containing 10 mM MES, 10 mM MgCl_2_, and 100 μM acetosyringone, and adjusted to an OD_600_ of 1.0. The suspension was then agroinfiltrated into *N. benthamiana* leaves. At 3 days after agroinfiltration, the plants were inoculated with CMV, and symptoms were recorded 5 days later. Leaf samples (0.1 g) were collected for western blot and RT‐qPCR analyses.

### 4.8 Synthesis of Intermediates and Target Compounds

The intermediates and target compounds were synthesised according to methods reported in the literature (Shaik et al. 2016; Chen et al. 2016; Wu et al. 2010; Zhao et al. 2020). Compounds **D1**–**D34** were synthesised via Duff reaction, nucleophilic substitution, and thioacetalisation. The structures of all target compounds were characterised using ^1^H NMR, ^13^C NMR, ^19^F NMR, and HRMS. Specific reaction details for the synthesis of intermediates for the title compounds are listed in the Supporting Information.

### 4.9 Antiviral Bioassay

Virus propagation, extraction, and activity testing were performed according to a previously reported method (Zhao et al. 2022). The virus was maintained in *N. tabacum* ‘K326’, and the protective, curative, and inactivating activities of **D1**–**D34** against CMV were tested using the half‐leaf local‐lesion method. *C. amaranticolor* was used as the test host, and all bioassays were repeated three times.

For whole‐plant antiviral validation in *N. benthamiana*, plants at the 4–5 leaf stage with uniform growth were used. The CMV infectious clone was first introduced into leaves by agroinfiltration. After 24 h, **D3** solution (500 μg mL^−1^) was infiltrated into the same leaves as a curative treatment. Control plants were infiltrated with an equal volume of DMSO aqueous solution. Symptoms were recorded at 7 dpi, and leaf samples were collected for subsequent western blot and RT‐qPCR analyses to determine viral protein accumulation and RNA levels.

### 4.10 Molecular Dynamics (MD) Simulations

Molecular dynamics (MD) simulations were performed in Amber22 to evaluate the stability of compound–CMV CP complexes, starting from docking poses. Systems were parameterised with ff14SB and GAFF force fields, solvated in a TIP3P water box, and neutralised with counterions. After equilibration, production runs were analysed by RMSD using Cpptraj (Jakalian et al. 2002; Maier et al. 2015; Price and Brooks 2004) (see Supporting Information for detailed protocols).

### 4.11 Determination of Bee Toxicity

Acute contact and oral toxicity of the compound were evaluated in *Apis mellifera* following standard procedures (Rasuli et al. 2017). For contact tests, 1 μL of compound solution was applied topically, while for oral tests, bees were fed compound in 5% honey solution. Mortality was recorded at 24 and 48 h (see Supporting Information for details).

### 4.12 DARTS Assay

The drug affinity responsive target stability (DARTS) assay was performed as described previously (Lomenick et al. 2009) with minor modifications. Briefly, protein extracts from CMV‐infected *N. tabacum* leaves were incubated with compound **D3** at different concentrations, followed by limited proteolysis with Pronase E. Protected proteins were analysed by SDS‐PAGE and immunoblotting, and potential targets were further identified by LC–MS/MS (see Supporting Information for details).

### 4.13 Molecular Docking

The molecular docking of compounds **D3** and **D24** with the CMV CP (PDB code: 1F15) was performed using Discovery Studio 4.5 software. The docking results were then visualised using PyMol.

### 4.14 Microscale Thermophoresis Assay

The binding affinities between the compounds and wild‐type or mutant CMV CP were determined using a Monolith NT.115 instrument (NanoTemper Technologies). Briefly, a range of ligand concentrations from 0 to 5 μM was incubated with 0.5 μM of purified wild‐type or mutant CMV CP for 5 min with NT‐647 dye (NanoTemper Technologies). After incubation, the mixed samples were loaded into the capillaries and analysed by the NanoTemper software to assess the equilibrium dissociation constants (Li et al. 2019).

### 4.15 Plant Growth and Virus Inoculation

A greenhouse was used to cultivate *N. tabacum*, *N. benthamiana*, and *C. amaranticolor* plants maintained at a 6/18‐h (dark/light) photoperiod and 25°C. The *Agrobacterium* strains containing CMV RNA1, RNA2, and RNA3 were cultured in Luria Bertani (LB) liquid medium with kanamycin (30 μg/mL) and rifampicin (30 μg/mL) at 28°C. Once the culture became turbid, the bacteria were harvested and resuspended in *Agrobacterium* buffer (10 mM MES, 10 mM MgCl_2_, 100 μM acetosyringone). The *Agrobacterium* suspensions were adjusted to an OD_600_ of 0.25. Equal volumes of RNA1, RNA2, and RNA3 were mixed together, incubated at 28°C for 2 h, and then used for agroinfiltration into *N. benthamiana* leaves (Zhang et al. 2017).

### 4.16 Purification of Proteins

Wild‐type and mutant CMV CP proteins were expressed in *Escherichia coli* BL21 (DE3) and induced with 1 mM IPTG at 16°C. Cells were harvested, lysed by sonication, and the supernatant was subjected to Ni‐NTA affinity chromatography on an AKTA system. Proteins were eluted with an imidazole gradient, desalted into phosphate buffer containing 300 mM NaCl and 10% glycerol, and concentrated. Protein purity and integrity were confirmed by SDS‐PAGE. GFP‐CMV CP, GFP‐CDPK7‐like, and CDPK7‐like proteins were purified using the same protocol (Zhang et al. 2016) (see Supporting Information for details).

### 4.17 Generation and Validation of *Nb*CDPK7‐Like Overexpression Transgenic Plants

The coding sequence of *NbCDPK7‐like* was cloned into a plant expression vector carrying a C‐terminal Flag tag and introduced into *A. tumefaciens* GV3101. Stable transgenic *N. benthamiana* plants were generated by an *Agrobacterium*‐mediated leaf disc transformation method. Leaf explants were infected with *Agrobacterium* (OD_600_ = 0.8–1.0) for 10 min, co‐cultivated in the dark for 2 days, and then transferred to selection medium containing kanamycin (100 mg/L) and timentin (300 mg/L). Regenerated shoots were rooted and transplanted after acclimation. Putative transgenic plants were initially screened by PCR based on the expected amplicon size. Positive lines were further validated by RT‐qPCR and anti‐Flag western blot, and stable overexpression lines were used for subsequent experiments.

### 4.18 RNA Extraction, RT‐PCR, and RT‐qPCR

RT‐qPCR analysis was performed as previously described (Shi et al. 2018). The leaves of the assayed *N. benthamiana* were used to extract total RNA by RNAiso Plus (TaKaRa). Reverse transcription was carried out using gene‐specific primers or random primers and a reverse transcriptase kit (TaKaRa) according to the instructions. The 2× Hieff PCR Master Mix (Yeasen) was used to execute PCR. qPCR was conducted using the TB Green Premix Ex Taq II (TaKaRa). All RT‐qPCR primers used in this study are listed in the Supporting Information (Table S8). All RT‐qPCR experiments were repeated three times to ensure the reliability and accuracy of the results.

### 4.19 Western Blot

Total proteins were extracted from *N. benthamiana* leaves as described previously (Li, Feng, et al. 2022). Extracts were separated by SDS‐PAGE and transferred onto PVDF membranes. After blocking, membranes were probed with specific primary antibodies and horseradish peroxidase‐conjugated secondary antibodies, and signals were detected using ECL reagents (see Supporting Information for detailed procedures).

### 4.20 Quantification and Statistical Analysis

The statistical analysis was conducted using GraphPad Prism 9.5, employing both Student's *t*‐test and one‐way ANOVA to assess significance. For Student's *t*‐test, *p*‐values greater than 0.05 were considered non‐significant and denoted as (ns), while *p*‐values less than 0.05 were considered statistically significant and denoted by (*), *p*‐values less than 0.01 by (**), *p*‐values less than 0.001 by (***), and *p*‐values less than 0.0001 by (****). Similarly, for one‐way ANOVA, *p*‐values less than 0.05 were also considered indicative of significant differences.

## Author Contributions

**Lu Yu:** writing – review and editing, supervision, conceptualization. **Lei Zhao:** writing – original draft, visualization, validation, resources, methodology, investigation, formal analysis, data curation. **Tangbing Yang:** methodology, investigation. **Qingwei Song:** formal analysis, software. **Xingjie Zhang:** formal analysis, software. **Deyu Hu:** writing – review and editing, supervision, funding acquisition, project administration. **Chunni Zhao:** formal analysis. **Runjiang Song:** writing – review and editing, supervision, project administration, conceptualization.

## Funding

This work was supported by the National Key Research and Development Program of China (2023YFD1700505), National Natural Science Foundation of China (32302388), Scientific Research Innovation Team of Guizhou University (202403).

## Conflicts of Interest

The authors declare no conflicts of interest.

## Supporting information


**Figure S1:** BiFC validation of the interactions of CMV CP with PLP and COMT1, and expression analysis of the corresponding fusion components. (A) Bimolecular fluorescence complementation (BiFC) assays showing the interactions of CMV CP with PLP and COMT1. YFP fluorescence, bright‐field, and merged images are shown. Clear YFP fluorescence signals were observed when CMV CP was co‐expressed with PLP or COMT1, whereas no obvious fluorescence signal was detected in the corresponding empty‐vector control combinations. Scale bars, 100 μm. (B) Expression analysis of the fusion components used in the PLP‐related BiFC assays. The expression of PLP‐FLAG, and CMV CP‐HA was detected by immunoblotting using anti‐FLAG and anti‐HA antibodies. Ponceau S staining was used as the loading control. (C) Expression analysis of the fusion components used in the COMT1‐related BiFC assays. The expression of COMT1‐FLAG, and CMV CP‐HA was detected by immunoblotting using anti‐FLAG and anti‐HA antibodies. Ponceau S staining was used as the loading control.

**Figure S2:** Confocal microscopy analysis of the subcellular localisation of CMV CP and CDPK7‐like and their co‐localisation in *N. benthamiana* epidermal cells. (A) Subcellular localisation of GFP‐CMV CP in *N. benthamiana* epidermal cells. (B) Subcellular localisation of mCherry‐CDPK7‐like in *N. benthamiana* epidermal cells. (C) Co‐expression of GFP‐CMV CP and mCherry‐CDPK7‐like in *N. benthamiana* epidermal cells revealed overlapping fluorescence signals and condensate‐like structures. GFP, mCherry, bright‐field, and merged images are shown. Scale bars, 100 μm in (A) and (B), and 50 μm in (C).

**Figure S3:** Subcellular localisation of CMV CP–CDPK7‐like condensates in *N. benthamiana*. (A) BiFC analysis showing the interaction between CMV CP and CDPK7‐like and the association of condensates with the nucleus. Nuclear localisation was indicated using a nuclear marker. (B) BiFC analysis showing the localisation of CMV CP–CDPK7‐like condensates at the plasma membrane. A plasma membrane marker was used to indicate membrane localisation. Scale bars, 100 μm.

**Figure S4:** CMV infection and CMV CP expression induce *NbCDPK7‐like* expression in *N. benthamiana*. (A) Phenotypes of *N. benthamiana* plants inoculated with CMV or mock‐inoculated at 7 dpi. (B) Western blot analysis of CMV CP accumulation in CMV‐infected and mock‐treated plants. (C) RT‐qPCR analysis of the relative expression levels of *NbCDPK7‐like* in CMV‐infected and mock‐treated plants. (D) Phenotypes of *N. benthamiana* plants agroinfiltrated with an empty vector (EV) or a CMV CP expression construct at 3 days after agroinfiltration. (E) Western blot analysis of CMV CP protein accumulation in EV‐ and CMV CP–expressing leaves. (F) RT‐qPCR analysis of relative *NbCDPK7‐like* transcript levels in EV‐ and CMV CP–expressing tissues.

**Figure S5:** Relative expression of *NbCDPK7‐like* in TRV:CDPK7‐like plants as determined by RT‐qPCR.

**Figure S6:** Virus accumulation in WT and OE‐*Nb*CDPK7‐like plants infected with different viruses. (A) CMV infection in WT and OE‐*Nb*CDPK7‐like plants. Representative phenotypes are shown. Overexpression of *NbCDPK7‐like* was confirmed by RT‐qPCR and anti‐FLAG immunoblotting, and CMV accumulation was analysed by anti‐CMV CP immunoblotting and RT‐qPCR of CMV CP RNA levels. (B) PVY infection in WT and OE‐*Nb*CDPK7‐like plants. Representative phenotypes (top) and fluorescence images (bottom) are shown. Overexpression of *NbCDPK7‐like* was confirmed by RT‐qPCR and anti‐FLAG immunoblotting, and PVY accumulation was analysed by anti‐PVY CP immunoblotting and RT‐qPCR of PVY CP RNA levels. (C) PMMoV infection in WT and *OE*‐NbCDPK7‐like plants. Representative phenotypes (top) and fluorescence images (bottom) are shown. Overexpression of *NbCDPK7‐like* was confirmed by RT‐qPCR and anti‐FLAG immunoblotting, and PMMoV accumulation was analysed by anti‐PMMoV CP immunoblotting and RT‐qPCR of PMMoV CP RNA levels.

**Figure S7:** BiFC analysis of the interactions of CMV CP, PVY CP, and PMMoV CP with CDPK7‐like, and expression analysis of the corresponding BiFC components. (A) Bimolecular fluorescence complementation (BiFC) assays showing the interactions of CMV CP, PVY CP, and PMMoV CP with CDPK7‐like. YFP fluorescence, bright‐field, and merged images are shown. Obvious YFP fluorescence signals and condensate‐like structures were observed only when CMV CP was co‐expressed with CDPK7‐like, whereas no obvious fluorescence signals were detected when PVY CP or PMMoV CP was co‐expressed with *CDPK7‐like*. No fluorescence signal was detected in the empty‐vector control combinations. Scale bars, 100 μm. (B) Expression analysis of the individual BiFC components in the CMV CP‐related BiFC assays. The expression of CMV CP‐HA, and CDPK7‐like‐FLAG was detected using anti‐FLAG and anti‐HA antibodies, respectively. Ponceau S staining was used as the loading control. (C) Expression analysis of the individual BiFC components in the PVY CP‐related BiFC assays. The expression of PVY CP‐HA, and CDPK7‐like‐FLAG was detected using anti‐FLAG and anti‐HA antibodies, respectively. Ponceau S staining was used as the loading control. (D) Expression analysis of the individual BiFC components in the PMMoV CP‐related BiFC assays. The expression of PMMoV CP‐HA, and CDPK7‐like‐FLAG was detected using anti‐FLAG and anti‐HA antibodies, respectively. Ponceau S staining was used as the loading control.

**Figure S8:** Luciferase complementation assay (LCA) validation of the interactions of CMV CP^T52A^ with CDPK7‐like, COMT1, and PLP in *N. benthamiana* leaves.

**Table S1:** Genes identified by CO‐IP of CMV CP.

**Table S2:** Differentially expressed genes in *N. benthamiana* leaves expressing CMV CP versus the empty‐vector control.

**Table S3:** Antiviral activities of target compounds **D1**–**D34** against CMV in vivo.

**Table S4:** Total energy, HOMO, LUMO, energy gap, dipole moment, TPSA of **D24**, **D3**, and ribavirin.

**Table S5:** Acute contact toxicity test results of compound **D3** on Italian honeybees.

**Table S6:** Acute oral toxicity of **D3** to Italian honeybees.

**Table S7:** Protein identification results from the DARTS assay.

**Table S8:** Primer sequence used for RT‐qPCR.

**Video S1:** Representative confocal time‐lapse imaging showing fission of CMV CP–CDPK7‐like condensates in *N. benthamiana* leaves following co‐expression of cYFP‐CMV CP and nYFP‐CDPK7‐like at 60 h after agroinfiltration.

**Video S2:** Representative confocal time‐lapse imaging showing fusion of CMV CP–CDPK7‐like condensates in *N. benthamiana* leaves following co‐expression of cYFP‐CMV CP and nYFP‐CDPK7‐like at 60 h after agroinfiltration.

**Video S3:** In vivo FRAP analysis of CMV CP–CDPK7‐like condensates in *N. benthamiana* leaves showing fluorescence recovery after photobleaching.

**Video S4:** In vitro FRAP analysis of condensates formed by purified GFP‐CMV CP and GFP‐CDPK7‐like proteins showing fluorescence recovery after photobleaching.

**Video S5:** Representative confocal time‐lapse imaging showing the effect of 10% 1,6‐hexanediol treatment on CMV CP–CDPK7‐like condensates in *N. benthamiana* leaves. Leaves co‐expressing cYFP‐CMV CP and nYFP‐CDPK7‐like for 60 h after agroinfiltration were treated with 10% 1,6‐hexanediol, resulting in a reduction in condensate number and size under our experimental conditions.

**Methods S1.** Synthesis of intermediates and target compounds.

**Methods S2.** Antiviral bioassay.

**Methods S3.** Molecular dynamics (MD) simulations.

**Methods S4.** Bee toxicity assays.

**Methods S5.** DARTS assay.

**Methods S6.** Purification of proteins.

**Methods S7.** Western blot.

**Methods S8.** Biolayer interferometry (BLI) assays.

**Text S1:** Characterisation data of intermediates and target compounds.

## Data Availability

All data that support the findings of this study are included in the published article and its Supporting Information.
