# Supplementary material for: Cucumber Mosaic Virus Coat Protein Sequesters Host CDPK7‐Like Into Phase‐Separated Condensates to Promote Viral Infection
Source: Mol Plant Pathol. 2026 May 18;27(5):e70270. doi: 10.1111/mpp.70270 (PMC13181337; doi:10.1111/mpp.70270)
Supplement: Supplementary file 1 — Figure S1: BiFC validation of the interactions of CMV CP with PLP and COMT1, and expression analysis of the corresponding fusion components. (A) Bimolecular fluorescence complementation (BiFC) assays showing the interactions of CMV CP with PLP and COMT1. YFP fluorescence, bright‐field, and merged images are shown. Clear YFP fluorescence signals were observed when CMV CP was co‐expressed with PLP or COMT1, whereas no obvious fluorescence signal was detected in the corresponding empty‐vector control combinations. Scale bars, 100 μm. (B) Expression analysis of the fusion components used in the PLP‐related BiFC assays. The expression of PLP‐FLAG, and CMV CP‐HA was detected by immunoblotting using anti‐FLAG and anti‐HA antibodies. Ponceau S staining was used as the loading control. (C) Expression analysis of the fusion components used in the COMT1‐related BiFC assays. The expression of COMT1‐FLAG, and CMV CP‐HA was detected by immunoblotting using anti‐FLAG and anti‐HA antibodies. Ponceau S staining was used as the loading control. [file MPP-27-e70270-s002.docx]

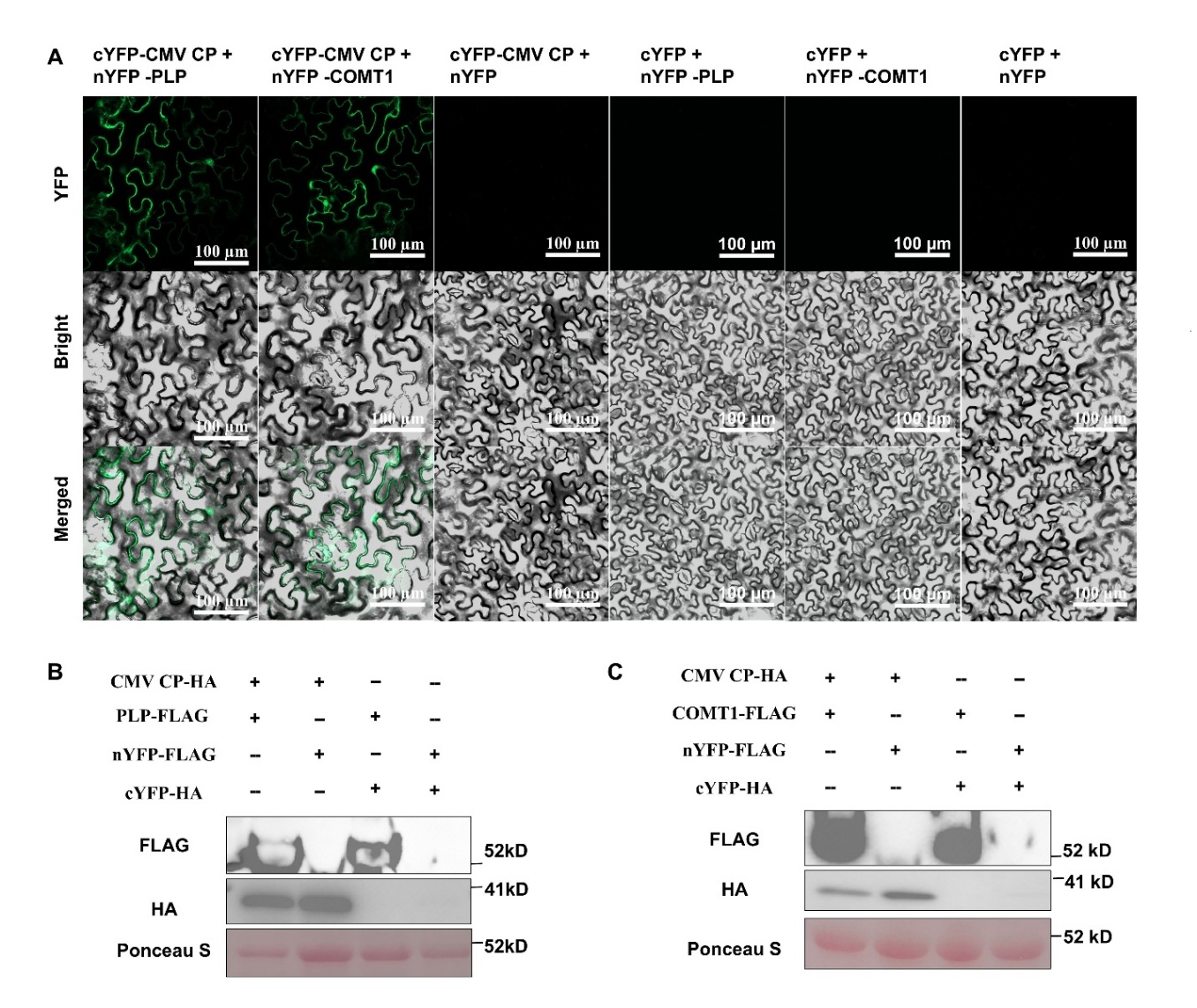


**FIGURE S1** | **BiFC validation of the interactions of CMV CP with PLP and COMT1, and expression analysis of the corresponding fusion components.** (A) Bimolecular fluorescence complementation (BiFC) assays showing the interactions of CMV CP with PLP and COMT1. YFP fluorescence, bright-field, and merged images are shown. Clear YFP fluorescence signals were observed when CMV CP was co-expressed with PLP or COMT1, whereas no obvious fluorescence signal was detected in the corresponding empty-vector control combinations. Scale bars, 100 μm. (B) Expression analysis of the fusion components used in the PLP-related BiFC assays. The expression of PLP-FLAG, and CMV CP-HA was detected by immunoblotting using anti-FLAG and anti-HA antibodies. Ponceau S staining was used as the loading control. (C) Expression analysis of the fusion components used in the COMT1-related BiFC assays. The expression of COMT1-FLAG, and CMV CP-HA was detected by immunoblotting using anti-FLAG and anti-HA antibodies. Ponceau S staining was used as the loading control.
