# Supplementary material for: Cucumber Mosaic Virus Coat Protein Sequesters Host CDPK7‐Like Into Phase‐Separated Condensates to Promote Viral Infection
Source: Mol Plant Pathol. 2026 May 18;27(5):e70270. doi: 10.1111/mpp.70270 (PMC13181337; doi:10.1111/mpp.70270)
Supplement: Supplementary file 2 — Figure S2: Confocal microscopy analysis of the subcellular localisation of CMV CP and CDPK7‐like and their co‐localisation in N. benthamiana epidermal cells. (A) Subcellular localisation of GFP‐CMV CP in N. benthamiana epidermal cells. (B) Subcellular localisation of mCherry‐CDPK7‐like in N. benthamiana epidermal cells. (C) Co‐expression of GFP‐CMV CP and mCherry‐CDPK7‐like in N. benthamiana epidermal cells revealed overlapping fluorescence signals and condensate‐like structures. GFP, mCherry, bright‐field, and merged images are shown. Scale bars, 100 μm in (A) and (B), and 50 μm in (C). [file MPP-27-e70270-s014.docx]

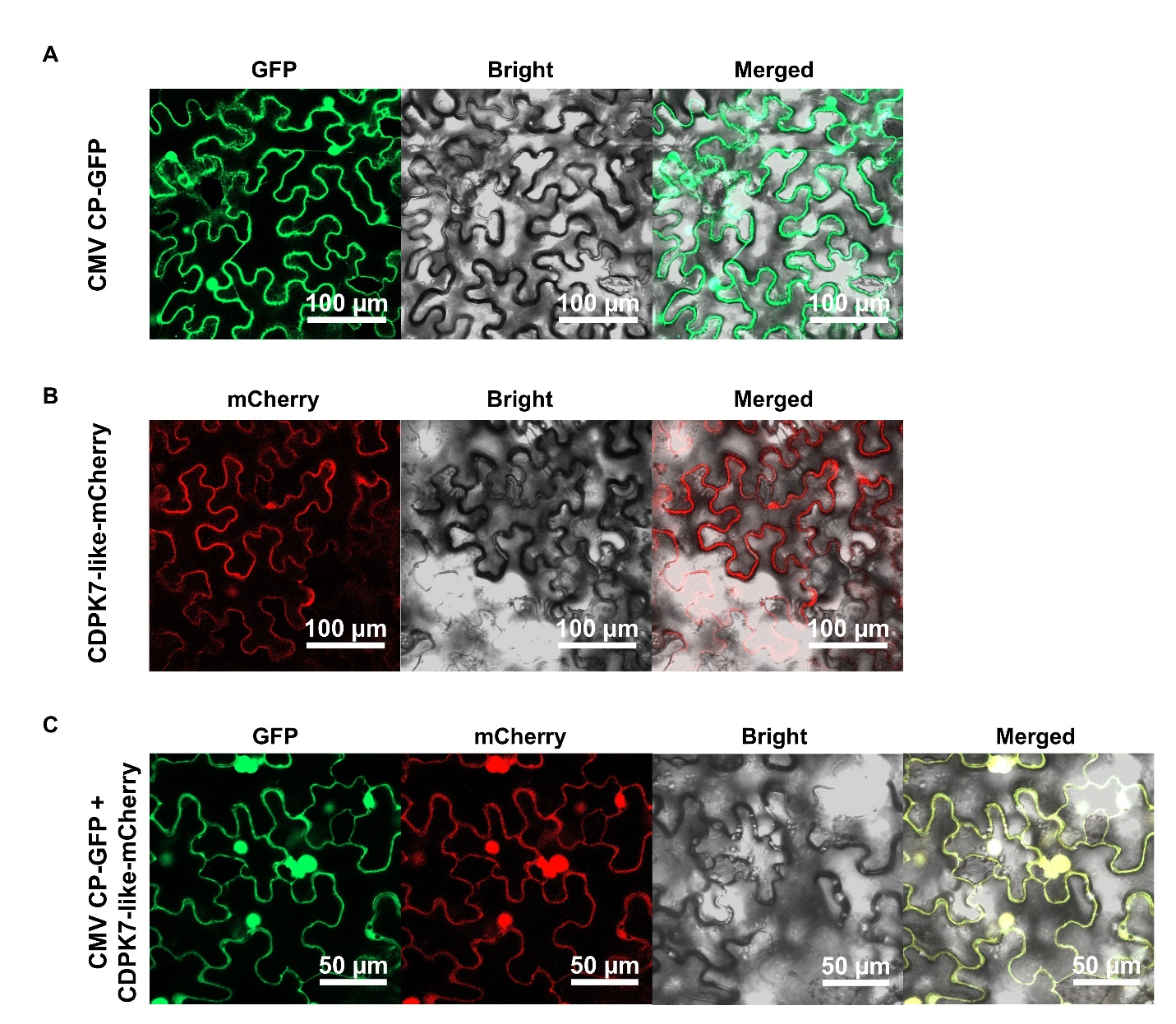


**FIGURE S2** | **Confocal microscopy analysis of the subcellular localization of CMV CP and CDPK7-like and their co-localization in *N. benthamiana* epidermal cells.**

(A) Subcellular localization of GFP-CMV CP in *N. benthamiana* epidermal cells.
(B) Subcellular localization of mCherry-CDPK7-like in *N. benthamiana* epidermal cells.
(C) Co-expression of GFP-CMV CP and mCherry-CDPK7-like in *N. benthamiana* epidermal cells revealed overlapping fluorescence signals and condensate-like structures. GFP, mCherry, bright-field, and merged images are shown. Scale bars, 100 μm in (A) and (B), and 50 μm in (C).
