# Supplementary material for: Cucumber Mosaic Virus Coat Protein Sequesters Host CDPK7‐Like Into Phase‐Separated Condensates to Promote Viral Infection
Source: Mol Plant Pathol. 2026 May 18;27(5):e70270. doi: 10.1111/mpp.70270 (PMC13181337; doi:10.1111/mpp.70270)
Supplement: Supplementary file 3 — Figure S3: Subcellular localisation of CMV CP–CDPK7‐like condensates in N. benthamiana. (A) BiFC analysis showing the interaction between CMV CP and CDPK7‐like and the association of condensates with the nucleus. Nuclear localisation was indicated using a nuclear marker. (B) BiFC analysis showing the localisation of CMV CP–CDPK7‐like condensates at the plasma membrane. A plasma membrane marker was used to indicate membrane localisation. Scale bars, 100 μm. [file MPP-27-e70270-s024.docx]

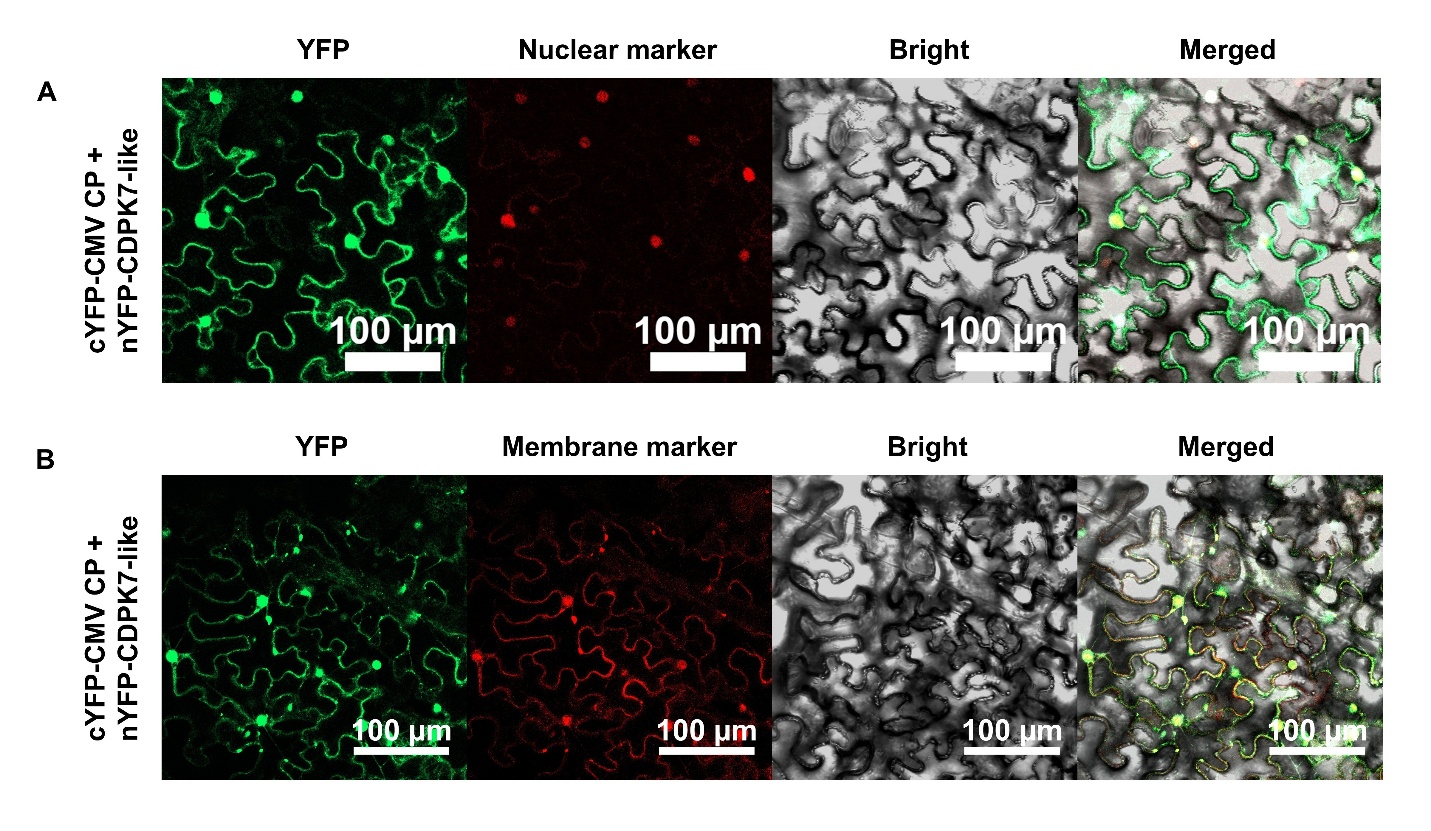


**FIGURE S3** | **Subcellular localization of CMV CP–CDPK7-like condensates in *N. benthamiana*.**(A) BiFC analysis showing the interaction between CMV CP and CDPK7-like and the association of condensates with the nucleus. Nuclear localization was indicated using a nuclear marker.

(B) BiFC analysis showing the localization of CMV CP–CDPK7-like condensates at the plasma membrane. A plasma membrane marker was used to indicate membrane localization. Scale bars, 100 μm.
