# Supplementary material for: Cucumber Mosaic Virus Coat Protein Sequesters Host CDPK7‐Like Into Phase‐Separated Condensates to Promote Viral Infection
Source: Mol Plant Pathol. 2026 May 18;27(5):e70270. doi: 10.1111/mpp.70270 (PMC13181337; doi:10.1111/mpp.70270)
Supplement: Supplementary file 4 — Figure S4: CMV infection and CMV CP expression induce NbCDPK7‐like expression in N. benthamiana. (A) Phenotypes of N. benthamiana plants inoculated with CMV or mock‐inoculated at 7 dpi. (B) Western blot analysis of CMV CP accumulation in CMV‐infected and mock‐treated plants. (C) RT‐qPCR analysis of the relative expression levels of NbCDPK7‐like in CMV‐infected and mock‐treated plants. (D) Phenotypes of N. benthamiana plants agroinfiltrated with an empty vector (EV) or a CMV CP expression construct at 3 days after agroinfiltration. (E) Western blot analysis of CMV CP protein accumulation in EV‐ and CMV CP–expressing leaves. (F) RT‐qPCR analysis of relative NbCDPK7‐like transcript levels in EV‐ and CMV CP–expressing tissues. [file MPP-27-e70270-s008.docx]

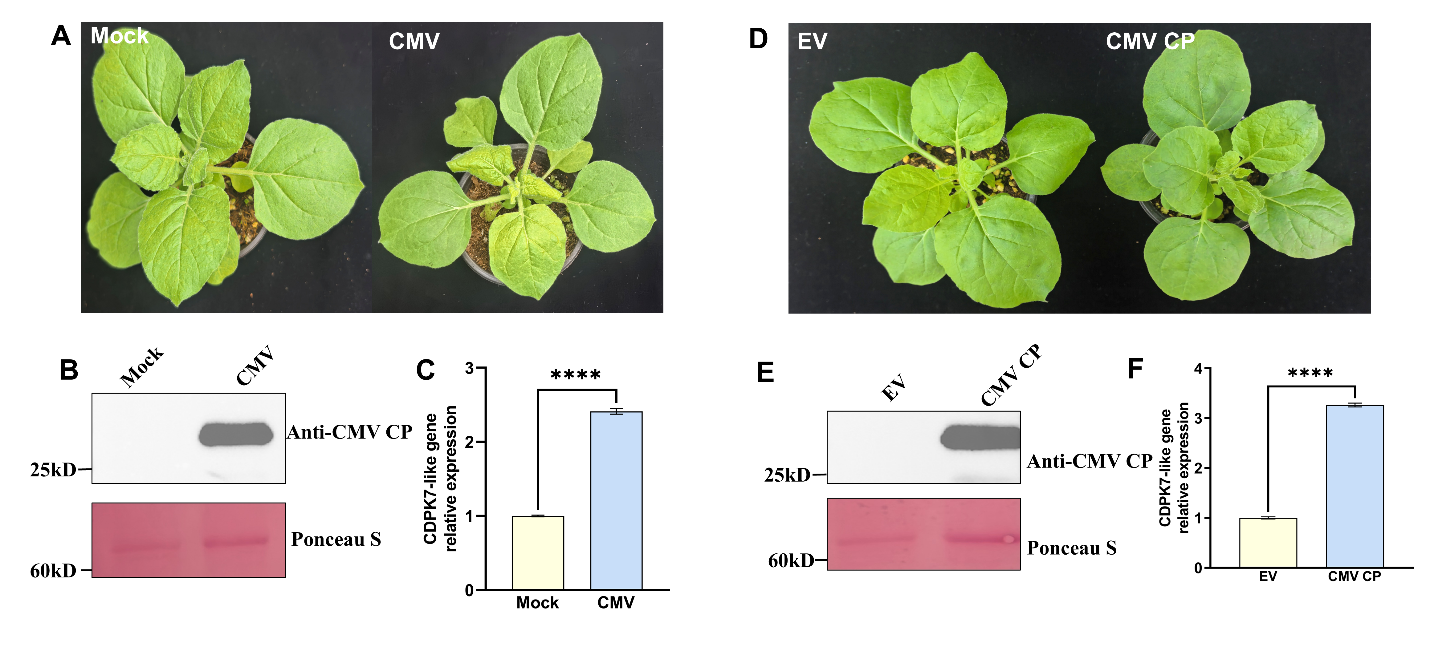


**FIGURE S4** | **CMV infection and CMV CP expression induce *NbCDPK7-like* expression in *N. benthamiana*.**

(A) Phenotypes of *N. benthamiana* plants inoculated with CMV or mock-inoculated at 7 dpi.
(B) Western blot analysis of CMV CP accumulation in CMV-infected and mock-treated plants. (C) RT-qPCR analysis of the relative expression levels of *NbCDPK7-like* in CMV-infected and mock-treated plants.

(D) Phenotypes of *N. benthamiana* plants agroinfiltrated with an empty vector (EV) or a CMV CP expression construct at 3 days after agroinfiltration.
(E) Western blot analysis of CMV CP protein accumulation in EV- and CMV CP–expressing leaves.
(F) RT-qPCR analysis of relative *NbCDPK7-like* transcript levels in EV- and CMV CP–expressing tissues.
