# Supplementary figures and images for: Cucumber Mosaic Virus Coat Protein Sequesters Host CDPK7‐Like Into Phase‐Separated Condensates to Promote Viral Infection
Source: Mol Plant Pathol. 2026 May 18;27(5):e70270. doi: 10.1111/mpp.70270 (PMC13181337; doi:10.1111/mpp.70270)

**
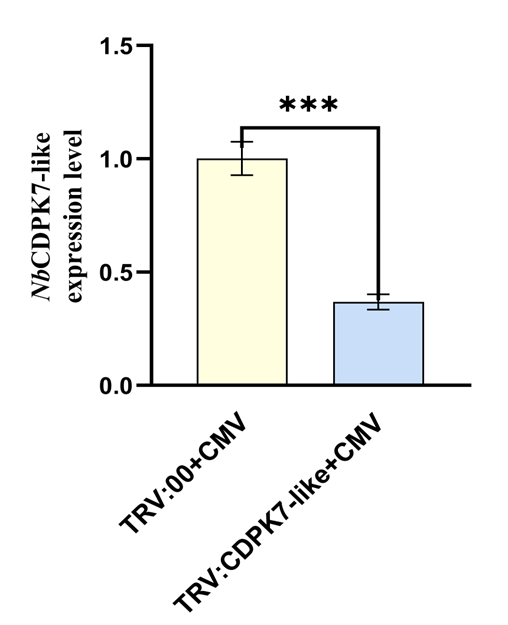
**

**FIGURE S5** | Relative expression of *NbCDPK7-like* in TRV:CDPK7-like plants as determined by RT-qPCR.

Supplement: Supplementary file 5 — Figure S5: Relative expression of NbCDPK7‐like in TRV:CDPK7‐like plants as determined by RT‐qPCR. [file MPP-27-e70270-s027.docx]
