# Supplementary material for: Cucumber Mosaic Virus Coat Protein Sequesters Host CDPK7‐Like Into Phase‐Separated Condensates to Promote Viral Infection
Source: Mol Plant Pathol. 2026 May 18;27(5):e70270. doi: 10.1111/mpp.70270 (PMC13181337; doi:10.1111/mpp.70270)
Supplement: Supplementary file 6 — Figure S6: Virus accumulation in WT and OE‐NbCDPK7‐like plants infected with different viruses. (A) CMV infection in WT and OE‐NbCDPK7‐like plants. Representative phenotypes are shown. Overexpression of NbCDPK7‐like was confirmed by RT‐qPCR and anti‐FLAG immunoblotting, and CMV accumulation was analysed by anti‐CMV CP immunoblotting and RT‐qPCR of CMV CP RNA levels. (B) PVY infection in WT and OE‐NbCDPK7‐like plants. Representative phenotypes (top) and fluorescence images (bottom) are shown. Overexpression of NbCDPK7‐like was confirmed by RT‐qPCR and anti‐FLAG immunoblotting, and PVY accumulation was analysed by anti‐PVY CP immunoblotting and RT‐qPCR of PVY CP RNA levels. (C) PMMoV infection in WT and OE‐NbCDPK7‐like plants. Representative phenotypes (top) and fluorescence images (bottom) are shown. Overexpression of NbCDPK7‐like was confirmed by RT‐qPCR and anti‐FLAG immunoblotting, and PMMoV accumulation was analysed by anti‐PMMoV CP immunoblotting and RT‐qPCR of PMMoV CP RNA levels. [file MPP-27-e70270-s013.docx]

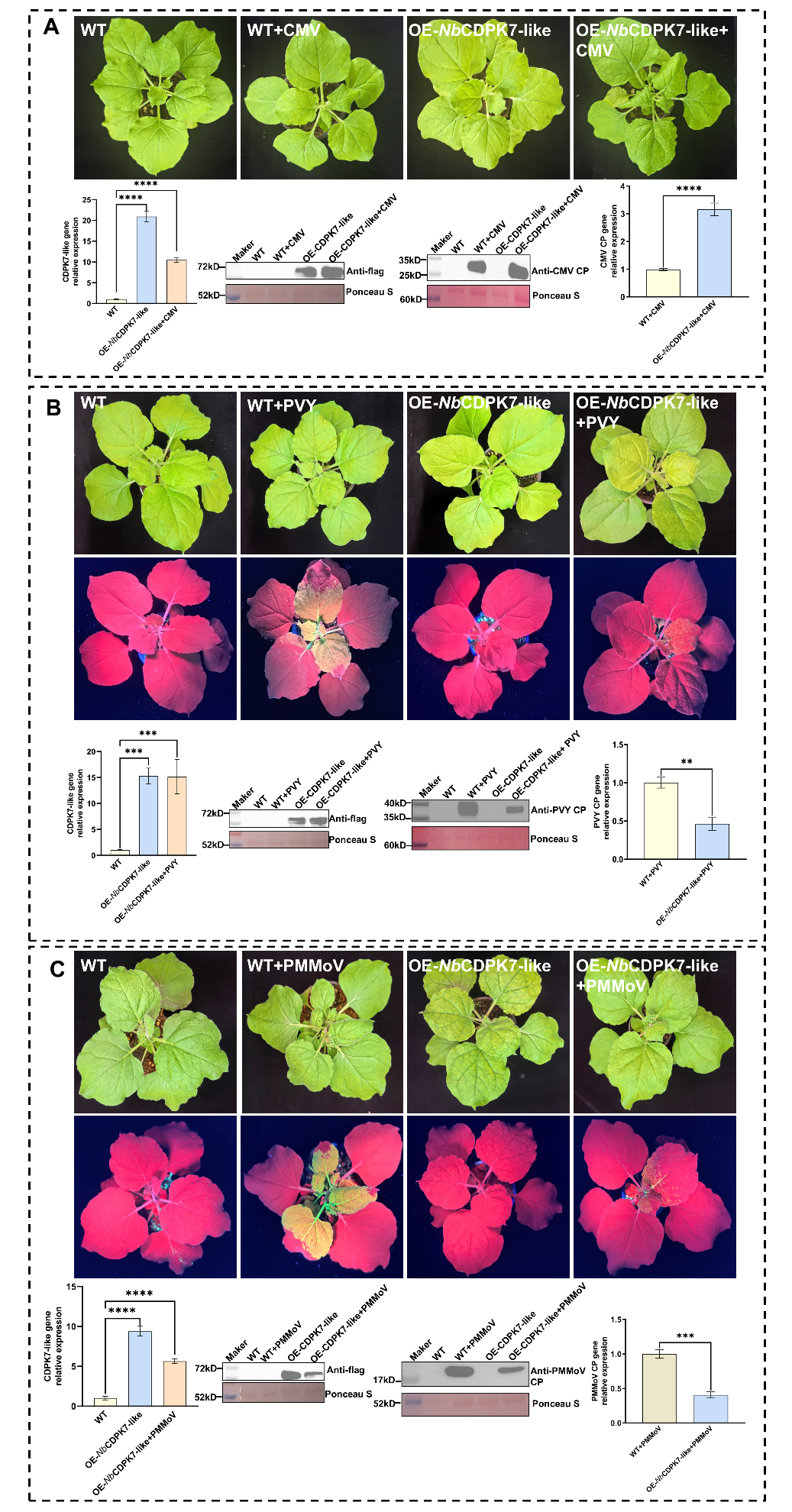


**FIGURE S6** | **Virus accumulation in WT and OE-*Nb*CDPK7-like plants infected with different viruses.** (A) CMV infection in WT and OE-NbCDPK7-like plants. Representative phenotypes are shown. Overexpression of *NbCDPK7-like* was confirmed by RT-qPCR and anti-FLAG immunoblotting, and CMV accumulation was analyzed by anti-CMV CP immunoblotting and RT-qPCR of CMV CP RNA levels. (B) PVY infection in WT and OE-*Nb*CDPK7-like plants. Representative phenotypes (top) and fluorescence images (bottom) are shown. Overexpression of *NbCDPK7-like* was confirmed by RT-qPCR and anti-FLAG immunoblotting, and PVY accumulation was analyzed by anti-PVY CP immunoblotting and RT-qPCR of PVY CP RNA levels. (C) PMMoV infection in WT and *OE*-NbCDPK7-like plants. Representative phenotypes (top) and fluorescence images (bottom) are shown. Overexpression of *NbCDPK7-like* was confirmed by RT-qPCR and anti-FLAG immunoblotting, and PMMoV accumulation was analyzed by anti-PMMoV CP immunoblotting and RT-qPCR of PMMoV CP RNA levels.
