# Supplementary material for: Cucumber Mosaic Virus Coat Protein Sequesters Host CDPK7‐Like Into Phase‐Separated Condensates to Promote Viral Infection
Source: Mol Plant Pathol. 2026 May 18;27(5):e70270. doi: 10.1111/mpp.70270 (PMC13181337; doi:10.1111/mpp.70270)
Supplement: Supplementary file 7 — Figure S7: BiFC analysis of the interactions of CMV CP, PVY CP, and PMMoV CP with CDPK7‐like, and expression analysis of the corresponding BiFC components. (A) Bimolecular fluorescence complementation (BiFC) assays showing the interactions of CMV CP, PVY CP, and PMMoV CP with CDPK7‐like. YFP fluorescence, bright‐field, and merged images are shown. Obvious YFP fluorescence signals and condensate‐like structures were observed only when CMV CP was co‐expressed with CDPK7‐like, whereas no obvious fluorescence signals were detected when PVY CP or PMMoV CP was co‐expressed with CDPK7‐like. No fluorescence signal was detected in the empty‐vector control combinations. Scale bars, 100 μm. (B) Expression analysis of the individual BiFC components in the CMV CP‐related BiFC assays. The expression of CMV CP‐HA, and CDPK7‐like‐FLAG was detected using anti‐FLAG and anti‐HA antibodies, respectively. Ponceau S staining was used as the loading control. (C) Expression analysis of the individual BiFC components in the PVY CP‐related BiFC assays. The expression of PVY CP‐HA, and CDPK7‐like‐FLAG was detected using anti‐FLAG and anti‐HA antibodies, respectively. Ponceau S staining was used as the loading control. (D) Expression analysis of the individual BiFC components in the PMMoV CP‐related BiFC assays. The expression of PMMoV CP‐HA, and CDPK7‐like‐FLAG was detected using anti‐FLAG and anti‐HA antibodies, respectively. Ponceau S staining was used as the loading control. [file MPP-27-e70270-s023.docx]

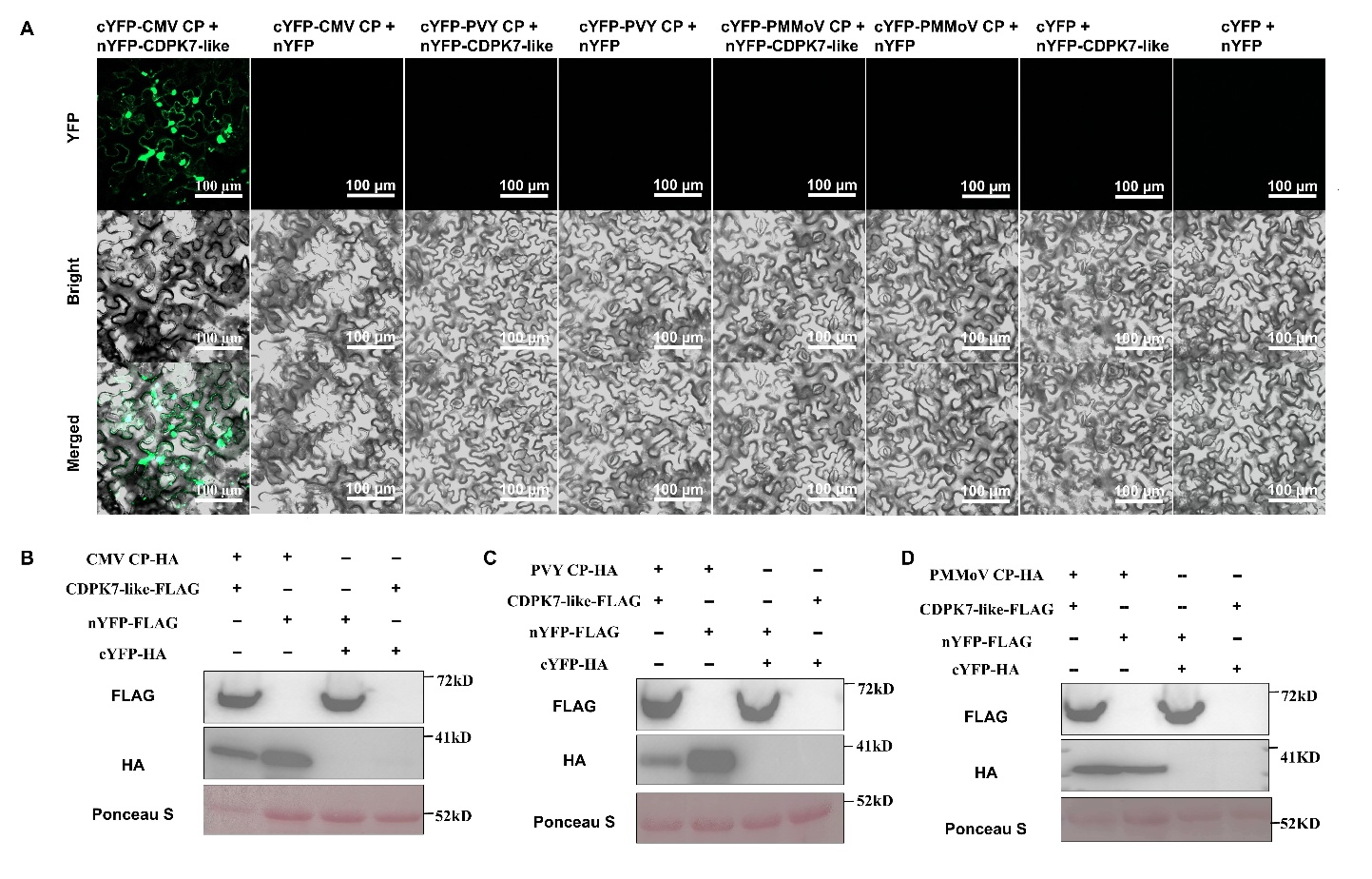


**FIGURE S7** | **BiFC analysis of the interactions of CMV CP, PVY CP, and PMMoV CP with CDPK7-like, and expression analysis of the corresponding BiFC components.** (A) Bimolecular fluorescence complementation (BiFC) assays showing the interactions of CMV CP, PVY CP, and PMMoV CP with CDPK7-like. YFP fluorescence, bright-field, and merged images are shown. Obvious YFP fluorescence signals and condensate-like structures were observed only when CMV CP was co-expressed with CDPK7-like, whereas no obvious fluorescence signals were detected when PVY CP or PMMoV CP was co-expressed with *CDPK7-like*. No fluorescence signal was detected in the empty-vector control combinations. Scale bars, 100 μm. (B) Expression analysis of the individual BiFC components in the CMV CP-related BiFC assays. The expression of CMV CP-HA, and CDPK7-like-FLAG was detected using anti-FLAG and anti-HA antibodies, respectively. Ponceau S staining was used as the loading control. (C) Expression analysis of the individual BiFC components in the PVY CP-related BiFC assays. The expression of PVY CP-HA, and CDPK7-like-FLAG was detected using anti-FLAG and anti-HA antibodies, respectively. Ponceau S staining was used as the loading control. (D) Expression analysis of the individual BiFC components in the PMMoV CP-related BiFC assays. The expression of PMMoV CP-HA, and CDPK7-like-FLAG was detected using anti-FLAG and anti-HA antibodies, respectively. Ponceau S staining was used as the loading control.
