# Supplementary material for: Cucumber Mosaic Virus Coat Protein Sequesters Host CDPK7‐Like Into Phase‐Separated Condensates to Promote Viral Infection
Source: Mol Plant Pathol. 2026 May 18;27(5):e70270. doi: 10.1111/mpp.70270 (PMC13181337; doi:10.1111/mpp.70270)
Supplement: Supplementary file 8 — Figure S8: Luciferase complementation assay (LCA) validation of the interactions of CMV CPT52A with CDPK7‐like, COMT1, and PLP in N. benthamiana leaves. [file MPP-27-e70270-s004.docx]

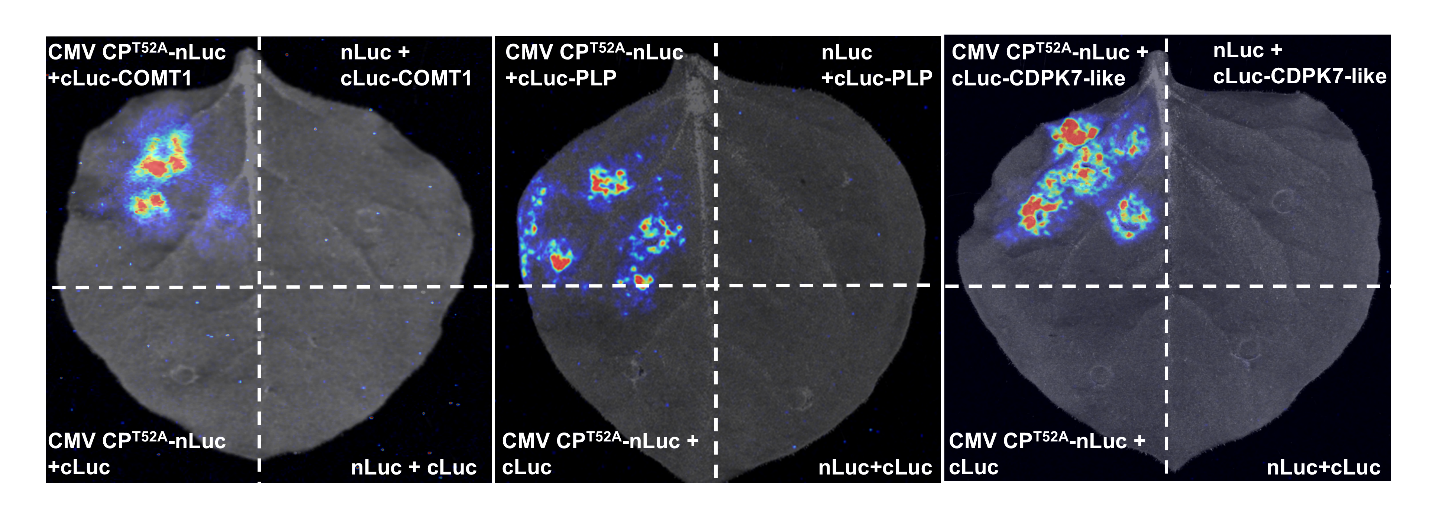


**FIGURE S8** | **Luciferase complementation assay (LCA) validation of the interactions of CMV CP^T52A^ with CDPK7-like, COMT1, and PLP in *N. benthamiana* leaves.**
