# Supplementary material for: Cucumber Mosaic Virus Coat Protein Sequesters Host CDPK7‐Like Into Phase‐Separated Condensates to Promote Viral Infection
Source: Mol Plant Pathol. 2026 May 18;27(5):e70270. doi: 10.1111/mpp.70270 (PMC13181337; doi:10.1111/mpp.70270)
Supplement: Supplementary file 9 — Table S1: Genes identified by CO‐IP of CMV CP. [file MPP-27-e70270-s018.docx]

**Table S1** Genes identified by CO-IP of CMV CP

| **Gene Accession** | **Protein Description** | **Gene ID** |
| --- | --- | --- |
| Niben101Scf02004g00031 | Aldo/keto reductase family | LGALDH |
| Niben101Scf00949g03003 | Protein TIC 100 | LOC107763893 |
| Niben101Scf13448g00015 | Belongs to the peptidase S10 family | LOC107813306 |
| Niben101Scf01795g01004 | Domain of unknown function (DUF296) | LOC107829884 |
| A0A2Z6IG95 | Geranylgeranyl pyrophosphate synthase 1 | NbGGPPS1 |
| Niben101Scf04558g02002 | molybdopterin biosynthesis | LOC107812843 |
| Niben101Scf06423g03031 | phosphatase 2 | PGLP2 |
| Niben101Scf09078g01011 | NifU-like domain | LOC107803163 |
| Niben101Scf06369g03006 | Belongs to the glutathione peroxidase family | GPX4 |
| Niben101Scf00779g06009 | 12-oxophytodienoate reductase | LOC107789205 |
| Niben101Scf06562g00009 | NAD-binding of NADP-dependent 3-hydroxyisobutyrate dehydrogenase | LOC107808859 |
| Niben101Scf17987g00001 | Photosystem I P700 chlorophyll a apoprotein | #N/A |
| Niben101Scf00854g01025 | This b-type cytochrome is tightly associated with the reaction center of photosystem II (PSII). PSII is a light-driven water plastoquinone oxidoreductase that uses light energy to abstract electrons from H(2)O, generating O(2) and a proton gradient subsequently used for ATP formation. It consists of a core antenna complex that captures photons, and an electron transfer chain that converts photonic excitation into a charge separation | #N/A |
| Niben101Scf00851g01013 | Adrenodoxin-like protein, mitochondrial | LOC107813305 |
| Niben101Scf01428g03001 | HR-like lesion-inducing | LOC107760737 |
| Niben101Scf08984g00007 | YGGT family | LOC107786710 |
| Niben101Scf05961g03018 | membrane depolarization during action potential | #N/A |
| Niben101Scf04475g00006 | CAAD domains of cyanobacterial aminoacyl-tRNA synthetase | CURT1D |
| Niben101Scf03283g04015 | Transmembrane proteins 14C | FAX1_0 |
| Niben101Scf03645g04018 | Removes the formyl group from the N-terminal Met of newly synthesized proteins | LOC104220094 |
| Niben101Scf00986g02003 | The proteasome is a multicatalytic proteinase complex which is characterized by its ability to cleave peptides with Arg, Phe, Tyr, Leu, and Glu adjacent to the leaving group at neutral or slightly basic pH | LOC107800869 |
| Niben101Scf04487g06016 | peptide chain release factor 1, mitochondrial | LOC107767663 |
| Niben101Scf05820g01009 | Ferredoxin | LOC107803171 |
| Niben101Scf05964g00023 | Sad1 / UNC-like C-terminal | SUN1 |
| Niben101Scf00953g06001 | GAD domain | A4A49_11819 |
| Niben101Scf02090g04020 | Oxidoreductase family, NAD-binding Rossmann fold | A4A49_21615 |
| Niben101Scf00646g09005 | Zinc knuckle | #N/A |
| Niben101Scf02090g07014 | programmed cell death protein 4 | LOC104220403 |
| Niben101Scf01281g01007 | Cytochrome b6 | #N/A |
| Niben101Scf06876g01028 | Rubredoxin | LOC107776709 |
| Niben101Scf02133g10010 | emp24/gp25L/p24 family/GOLD | LOC107803670 |
| Niben101Scf00713g05006 | Hydantoinase B/oxoprolinase | LOC107803589 |
| Niben101Scf06876g01037 | MafB19-like deaminase | LOC107776708 |
| Niben101Scf06848g05003 | Transferase family | BAHD1_6 |
| Niben101Scf00821g12007 | Membrane steroid-binding protein 2-like | #N/A |
| Niben101Scf07086g00019 | translocase of chloroplast 159, chloroplastic isoform X1 | LOC104230719 |
| Niben101Scf04325g01016 | Methylthioribose-1-phosphate isomerase | CIG2_2 |
| Niben101Scf07030g04004 | GHMP kinases C terminal | LOC104216953 |
| Niben101Scf00621g01007 | At4g15545-like | LOC107784131 |
| Niben101Scf03004g03025 | Protein of unknown function DUF89 | A4A49_55768 |
| Niben101Scf00647g02011 | Eukaryotic translation initiation factor 3 subunit G | LOC107829260 |
| Niben101Scf06958g02005 | Belongs to the GST superfamily | LOC104220805 |
| Niben101Scf06906g01008 | Myb-like protein X | LOC107804014 |
| Niben101Scf06757g00004 | Sucrose synthase | SUS7 |
| Niben101Scf18002g00001 | Nuclear transcription factor Y subunit C-1-like | CIP2a |
| Niben101Scf00849g01018 | Chalcone isomerase like | FAP1_1 |
| Niben101Scf03116g02005 | RNA recognition motif | RBG3_3 |
| Niben101Scf18106g01006 | Proteasome subunit beta | LOC107810770 |
| Niben101Scf06483g02003 | LOC104250150 isoform | LOC104250150 |
| Niben101Scf06280g05017 | Condensin complex subunit 2 | CAPH |
| Niben101Scf00700g09001 | Cation-independent O- methyltransferase 1 | HOMT1_2 |
| Niben101Scf01369g05022 | uncharacterized protein | LOC104234463 |
| Niben101Scf00856g00005 | K homology RNA-binding domain | LOC107822433 |
| Niben101Scf11078g01009 | Proteasome subunit alpha type | ES332_A06G170200v1 |
| Niben101Scf03766g03001 | Rubisco LSMT substrate-binding | LOC107767517 |
| Niben101Scf25021g01016 | Chlorophyll a-b binding protein, chloroplastic | LOC107775375 |
| Niben101Scf01314g00017 | uncharacterized protein | LOC107784071 |
| Niben101Scf01328g01013 | Regulator of chromosome condensation (RCC1) repeat | LOC107813665 |
| Niben101Scf02387g01003 | tetraacyldisaccharide 4'-kinase | LOC104215422 |
| Niben101Scf03714g05009 | Cytochrome c oxidase subunit VII | LOC107823918 |
| Niben101Scf06569g00001 | Embryo-specific protein 3, (ATS3) | LOC107793109 |
| Niben101Scf05158g01021 | peptidyl-prolyl cis-trans isomerase | CYP26-2 |
| Niben101Scf15752g00002 | calcium-dependent protein kinase 7-like | LOC107815101 |
| Niben101Scf03197g06004 | GRIM-19 protein | LOC107770117 |
| A0A1V1H6S6 | non-specific serine/threonine protein kinase | NbCDPKiso2 |
| Niben101Scf01269g04014 | RNA recognition motif. (a.k.a. RRM, RBD, or RNP domain) | RBP45_1 |
| Niben101Scf01267g00002 | Belongs to the class-IV pyridoxal-phosphate-dependent aminotransferase family | LOC107778190 |
| Niben101Scf15077g01009 | Serine-threonine kinase receptor-associated | TIF3I1_0 |
| Niben101Scf05431g01014 | AhpC/TSA antioxidant enzyme | LOC107780627 |
| Niben101Scf04555g00003 | Glutaredoxin | LOC107784219 |
| Niben101Scf03570g01013 | HR-like lesion-inducing | LOC107786901 |
| Niben101Scf04457g04017 | tRNA (adenine-N6-)-methyltransferase activity | LOC107820881 |
| Niben101Scf03494g11004 | Rhodanese Homology Domain | LOC107817113 |
| Niben101Scf10735g00016 | Pathogenesis-related protein STH-2-like | LOC104240635 |
| Niben101Scf03687g01001 | Nicotinate-nucleotide pyrophosphorylase carboxylating | QPT1a |
| Niben101Scf04709g00004 | Chloroplast import apparatus Tic20-like | #N/A |
| Niben101Scf04918g08002 | CYTH | LOC107785496 |
| Niben101Scf03514g00004 | Embryo-specific protein 3, (ATS3) | LOC107787366 |
| Niben101Scf08034g00002 | Protein of unknown function (DUF1279) | LOC107819445 |
| Niben101Scf03817g03033 | NADH dehydrogenase ubiquinone 1 alpha subcomplex assembly factor | LOC107829281 |
| Niben101Scf01066g04002 | 5' nucleotidase family | LOC107808313 |
| Niben101Scf03376g04013 | methionine sulfoxide reductase | MSRB2 |
| Niben101Scf05422g00019 | Tetratricopeptide repeat | #N/A |
| Niben101Scf01062g08013 | Belongs to the nonaspanin (TM9SF) (TC 9.A.2) family | LOC107790799 |
| Niben101Scf03326g01016 | VHS domain | LOC107817777 |
| Niben101Scf05438g02007 | Catalyzes the conversion of hemimercaptal, formed from methylglyoxal and glutathione, to S-lactoylglutathione | GLYI-11 |
| Niben101Scf09459g00003 | Belongs to the class-I pyridine nucleotide-disulfide oxidoreductase family | LOC107777121 |
| Niben101Scf01035g03002 | cytochrome P450 | C7A12_5 |
| Niben101Scf03038g06012 | Uncharacterised protein family (UPF0172) | A4A49_06643 |
| Niben101Scf01047g00001 | Belongs to the phosphohexose mutase family | DRT101 |
| Niben101Scf01033g03005 | non-haem dioxygenase in morphine synthesis N-terminal | A4A49_34165 |
| Niben101Scf01438g04009 | Replication protein A C terminal | RPA2A |
| Niben101Scf01781g01002 | Thaumatin family | OLP1 |
| Niben101Scf05029g00004 | UDP-sugar pyrophosphorylase | USP |
| Niben101Scf01237g13015 | Superoxide dismutase | LOC104249630 |
| Niben101Scf03357g00015 | Proline iminopeptidase | LOC104216368 |
| Niben101Scf03365g00008 | NmrA-like family | A4A49_16147 |
| Niben101Scf06008g00011 | Protein of unknown function (DUF1118) | LOC107823677 |
| Niben101Scf03374g09001 | Serine racemase | LOC107828409 |
| Niben101Scf10941g01004 | Bifunctional protein FolD 2-like | LOC104237570 |
| Niben101Scf05316g03010 | Cytochrome P450 | LOC107820237 |
| Niben101Scf01115g00003 | DNA photolyase | LOC107760080 |
| Niben101Scf13558g01003 | Protein of unknown function (DUF3148) | LOC107782810 |
| Niben101Scf13111g00006 | Uncharacterised protein family (UPF0160) | A4A49_13680 |
| Niben101Scf12987g00001 | Ribonuclease H protein | #N/A |
| Niben101Scf13393g00004 | Iron-binding zinc finger CDGSH type | LOC107803753 |
| Niben101Scf01578g04001 | probable calcium-binding protein CML50 | LOC107827669 |
| Niben101Scf12159g00013 | heat shock protein | LOC107775218 |
| Niben101Scf02608g01016 | Synechocystis YCF37 | A4A49_37286 |
| Niben101Scf00185g01001 | ABA/WDS induced protein | LOC107760194 |
| Niben101Scf02442g00015 | FAM10 family protein At4g22670-like | A4A49_56894 |
| Niben101Scf05009g00002 | Belongs to the CDP-alcohol phosphatidyltransferase class-I family | PGPS1_1 |
| Niben101Scf01623g14008 | Domain of unknown function (DUF4149) | LOC107788056 |
| Q2QFR2 | Cysteine proteinase glycinain type | #N/A |
| Niben101Scf02625g02001 | Indole-3-glycerol phosphate synthase, chloroplastic-like | IGPS_1 |
| Niben101Scf11756g01002 | RNA binding (RRM RBD RNP motifs) family protein | A4A49_10847 |
| Niben101Scf08341g05018 | leucine-rich repeat extensin-like protein | LOC107822178 |
| Niben101Scf11078g01010 | proteasome subunit alpha type-5-like | LOC107817032 |
| Niben101Scf04083g01027 | Transcription factor Pcc1 | LOC104232662 |
| Niben101Scf00294g00014 | trypsin inhibitor | #N/A |
| B2D077 | Methyltransferase | #N/A |
| Niben101Scf00606g02018 | Inorganic pyrophosphatase | #N/A |
| Niben101Scf00215g01001 | Replication protein A 14 kDa subunit | RPA3B_1 |
| Q20KN2 | Metacaspase type II | #N/A |
| Niben101Scf01376g04027 | pirin-like protein | LOC107763309 |
| Niben101Scf05395g03009 | Uncharacterized protein isoform X2 | LOC107826601 |
| Niben101Scf07648g01017 | LOC107776739 | LOC107776739 |
| Niben101Scf00259g00001 | Transmembrane proteins 14C | FAX1_0 |
| Niben101Scf00085g05010 | Profilin | LOC107820001 |
| Niben101Scf07231g02010 | Vesicle transport V-snare | LOC107774800 |
| Niben101Ctg01107g00003 | Calmodulin binding protein-like | #N/A |
| Niben101Scf02477g00013 | Serine threonine protein phosphatase 2A 55 kDa regulatory subunit B beta isoform-like isoform X1 | PP2AB2_0 |
| Niben101Scf03712g00010 | Proteasome subunit beta | LOC107831212 |
| Niben101Scf15836g00013 | Acyl-CoA dehydrogenase, C-terminal domain | IVD |
| Niben101Scf03944g02014 | DNA-dependent RNA polymerase catalyzes the transcription of DNA into RNA using the four ribonucleoside triphosphates as substrates | #N/A |
| Niben101Ctg11328g00002 | phosphatase 2c | LOC104212909 |
| Niben101Ctg12072g00001 | fructokinase activity | LOC107817420 |
| Niben101Ctg13092g00001 | Sn-1 protein | NtSN1b |
| Niben101Ctg14992g00001 | Protein of unknown function (DUF3007) | LOC107822436 |
| Niben101Ctg15035g00004 | Belongs to the Casparian strip membrane proteins (CASP) family | LOC107799862 |
| Niben101Scf10055g05004 | Endonuclease/Exonuclease/phosphatase family | LOC107794486 |
| Niben101Scf00119g01002 | Thaumatin family | LOC107824127 |
| Niben101Scf16582g00005 | Cytochrome c oxidase subunit | LOC107805558 |
| Niben101Scf00123g06014 | Glutaredoxin | LOC104236961 |
| Niben101Scf00127g01005 | The coatomer is a cytosolic protein complex that binds to dilysine motifs and reversibly associates with Golgi non- clathrin-coated vesicles, which further mediate biosynthetic protein transport from the ER, via the Golgi up to the trans Golgi network. Coatomer complex is required for budding from Golgi membranes, and is essential for the retrograde Golgi-to-ER transport of dilysine-tagged proteins | #N/A |
| Niben101Scf03907g00006 | Clp amino terminal domain, pathogenicity island component | CLPT1_1 |
| Niben101Scf03978g09005 | RNA recognition motif | BPA1_0 |
| Niben101Scf18121g00007 | Pleckstrin homology domain | LOC107787436 |
| Niben101Scf03937g00009 | Plastocyanin-like domain | BABL_1 |
| Niben101Scf17760g00015 | Isocitrate/isopropylmalate dehydrogenase | LOC107781243 |
| Niben101Scf14955g00012 | Soluble starch synthase 3, chloroplastic amyloplastic isoform X1 | SS3 |
| Niben101Ctg07933g00004 | Methionine gamma-lyase | #N/A |
| Niben101Scf07123g00003 | Uncharacterized protein | A4A49_04019 |
| Niben101Scf00525g04009 | Pyridine nucleotide-disulphide oxidoreductase | LOC107776152 |
| Niben101Scf04737g00007 | ATP synthase subunit d, mitochondrial | LOC107761950 |
| Niben101Scf08039g00007 | Endoplasmic reticulum transmembrane protein | A4A49_39120 |
| Niben101Scf06171g00001 | Belongs to the bacterial ribosomal protein bL31 family | LOC107777917 |
| Niben101Scf06112g02015 | Minor allergen Alt a | LOC104236231 |
| Niben101Scf00491g04045 | Eukaryotic aspartyl protease | ASPG1_5 |
| Niben101Scf00466g03018 | Aldo/keto reductase family | LOC107767965 |
| Niben101Scf01521g16018 | Bacterial PH domain | A4A49_02661 |
| Niben101Scf08926g08029 | Acetyltransferase (GNAT) domain | LOC107817186 |
| Niben101Scf00472g03019 | Osmotic stress-activated protein kinase | SnRK2.4 |
| Niben101Scf10213g00017 | Transmembrane 9 superfamily member | TMN7_2 |
| Q6VUQ2 | Protein-serine/threonine phosphatase | NPP5-1 |
| Niben101Scf07231g07005 | Flavin containing amine oxidoreductase | LOC107787313 |
| Niben101Scf03584g06021 | Belongs to the UDP-glycosyltransferase family | - |
| Niben101Scf03277g02015 | Arginine biosynthesis bifunctional protein ArgJ, chloroplastic | LOC104219972 |
| Niben101Scf07457g00004 | Leucine Rich repeat | #N/A |
| Q076B0 | RPN8 | #N/A |
| Niben101Scf01469g01006 | Methionine aminopeptidase | LOC104245859 |
| Niben101Scf00541g05002 | Redoxin | LOC107768657 |
| Niben101Scf06088g00005 | K homology RNA-binding domain | LOC104246626 |
| Niben101Scf00542g03004 | NADH-cytochrome b5 reductase | CBR2 |
| Niben101Scf02230g02002 | GrpE protein homolog | LOC107812441 |
| Niben101Scf10007g01011 | Protein YELLOW LEAF 1, choloroplastic-like | LOC107804991 |
| Niben101Scf05901g03008 | Acyl CoA binding protein | ACBP4_4 |
| Niben101Scf20669g00001 | LOC104240752 isoform X | LOC104240752 |
| Niben101Scf04375g08024 | first, biotin carboxylase catalyzes the carboxylation of the carrier protein and then the transcarboxylase transfers the carboxyl group to form malonyl-CoA | BCCP1 |
| Niben101Scf10616g00008 | Protein CHLORORESPIRATORY REDUCTION 7 | LOC107787887 |
| Niben101Scf00354g01012 | Acyl-transferase | LOC107778215 |
| Niben101Scf10283g01011 | Endoplasmic reticulum vesicle transporter | LOC107824863 |
| Niben101Scf08812g02011 | ATPase LOC104222995 | LOC104222995 |
| Niben101Scf00315g00020 | Import inner membrane translocase subunit tim21 | TIM21 |
| Niben101Scf02349g03001 | Belongs to the peroxidase family. Classical plant (class III) peroxidase subfamily | LOC104210110 |
| Niben101Scf00397g01008 | Uncharacterized protein | A4A49_21971 |
| Niben101Scf08874g01015 | DUF1338 | LOC104243986 |
| Niben101Scf06596g03014 | Peptidase family M20/M25/M40 | A4A49_34740 |
| Niben101Scf07353g02009 | TENA/THI-4/PQQC family | LOC107819674 |
| Niben101Scf11178g01004 | Metal transporter | LOC107792619 |
| Niben101Scf00430g01013 | Uncharacterized protein | A4A49_65669 |
| Niben101Scf00437g00015 | pentatricopeptide repeat-containing protein At4g16390, chloroplastic | LOC104242943 |
| Niben101Scf01722g03009 | Ureidoglycolate hydrolase | LOC107785953 |
| Niben101Scf04216g02006 | PsbP | LOC107819052 |
| Niben101Scf02405g03003 | trypsin inhibitor | #N/A |
| Niben101Scf07981g01018 | DEAD-box ATP-dependent RNA helicase | RH38_1 |
| Niben101Scf02622g10012 | Uncharacterized protein | LOC107787934 |
| Niben101Scf02821g04003 | tocopherol O-methyltransferase, chloroplastic isoform X1 | LOC104247874 |
| Niben101Scf02709g04001 | dehydratase shikimate | LOC107791190 |
| A0A2Z4WTD4 | 4a-hydroxytetrahydrobiopterin dehydratase | #N/A |
| Niben101Scf02526g00002 | Belongs to the glutathione peroxidase family | LOC107795121 |
| J7EYL4 | GRX1 | #N/A |
| Niben101Scf02562g01011 | eukaryotic translation initiation factor | A4A49_13943 |
| Niben101Ctg05133g00002 | Belongs to the PdxS SNZ family | Pdx1-B |
| Niben101Scf03375g02028 | Catalyzes the ferrous insertion into protoporphyrin IX | LOC107779891 |
| Niben101Scf09071g00010 | Photosystem II reaction center Psb28 protein | LOC107768014 |
| Niben101Scf02026g11001 | Proton-conducting membrane transporter | #N/A |
| Niben101Scf14144g00027 | Belongs to the nonaspanin (TM9SF) (TC 9.A.2) family | LOC107773219 |
| Niben101Scf06737g00032 | MA3 domain | A4A49_15301 |
| Niben101Scf02791g01003 | phosphatase 2c | LOC111489768 |
| Niben101Scf02269g00005 | Rhodanese-like domain | LOC107781654 |
| Niben101Scf00389g01002 | Thylakoid lumenal 15 kDa protein | LOC104245014 |
| Niben101Scf00409g03001 | V-type proton ATPase subunit G | VATG1 |
| Niben101Scf11341g00010 | Enolase, N-terminal domain | LOC107832416 |
| Niben101Scf07352g00001 | phosphoenolpyruvate | COCNU_14G003850 |
| Niben101Scf36540g00003 | peptidyl-prolyl cis-trans isomerase | PNSL4 |
| Niben101Scf00057g00027 | Plastocyanin | LOC107792758 |
| Niben101Scf07255g01023 | NADH dehydrogenase ubiquinone iron-sulfur protein 8 | #N/A |
| Niben101Scf21459g00012 | Universal stress protein A-like protein | PHOS34_1 |
| Niben101Scf00126g03005 | prohibitin homologues | LOC104216651 |
| Niben101Scf00022g06009 | DUF7880 domain-containing protein | LOC107805847 |
| Niben101Scf28836g00001 | Prolyl oligopeptidase family | LOC104224688 |
| Niben101Scf13569g00001 | Belongs to the enoyl-CoA hydratase isomerase family | LOC107787165 |
| Niben101Scf11795g00012 | DUF1338 | A4A49_36685 |
| Niben101Scf15156g00001 | Dihydrodipicolinate reductase, C-terminus | LOC107766908 |
| Niben101Scf12848g00007 | Macrophage migration inhibitory factor (MIF) | LOC107788402 |
| Niben101Scf01451g05003 | Eukaryotic porin | LOC107777563 |
| Niben101Scf04727g07004 | Rubisco LSMT substrate-binding | LOC107787484 |
| Niben101Scf04205g00005 | BPI/LBP/CETP C-terminal domain | LOC107814007 |
| Niben101Scf08446g00014 | 6-phosphogluconolactonase 4, chloroplastic | LOC107827206 |
| Niben101Scf04339g04002 | Tryptophan synthase | LOC104239837 |
| Niben101Scf04398g01005 | Protein of unknown function (DUF760) | A4A49_25861 |
| Niben101Scf05712g02002 | Ribulose-1,5 bisphosphate carboxylase oxygenase large subunit N-methyltransferase, chloroplastic | RBCMT_0 |
| Niben101Scf15752g00007 | Elongation factor G C-terminus | LOC107805650 |
| Niben101Scf05405g08004 | Thioredoxin-like | PDIL1-3 |
| Niben101Scf05669g02028 | Protein of unknown function (DUF760) | LOC107816208 |
| Niben101Scf01153g00016 | ATP-dependent 6-phosphofructokinase | PFK3_1 |
| B7XH28 | GDP-mannose 4,6-dehydratase | GMD |
| Niben101Scf03076g01025 | Scavenger mRNA decapping enzyme C-term binding | LOC107811311 |
| Niben101Scf04287g06007 | ATP-citrate synthase alpha chain protein | LOC107799450 |
| Niben101Scf01696g06053 | Calcium-binding EF hand family protein | LOC104217073 |
| Niben101Scf00557g00008 | LOC104227702 isoform X | LOC104227702 |
| Niben101Scf02804g00003 | DNA-damage-repair toleration protein DRT102 | LOC107830916 |
| Niben101Scf00698g02019 | Aldo/keto reductase family | LOC104210408 |
| Niben101Scf06506g00001 | Peroxisomal fatty acid beta-oxidation multifunctional protein AIM1-like | LOC104235287 |
| Niben101Scf00822g01007 | Enoyl-(Acyl carrier protein) reductase | A4A49_42256 |
| Niben101Scf05649g06001 | Small subunit of acetolactate synthase | LOC104231515 |
| Niben101Scf01402g00005 | Signal recognition particle | LOC107768293 |
| Niben101Scf01899g07007 | 3-isopropylmalate dehydrogenase | LOC107760060 |
| Niben101Scf03954g01001 | Thioredoxin Y1, chloroplastic | A4A49_05391 |
| Niben101Scf07252g00007 | Eukaryotic translation initiation factor 3 subunit E | LOC107801330 |
| Q58IU5 | Non-intrinsic ABC protein | #N/A |
| Niben101Scf04765g00002 | Glutaredoxin | GRXS17 |
| Niben101Scf04292g11008 | Pyruvate kinase, cytosolic | LOC104210658 |
| Niben101Scf05486g00009 | Belongs to the class-I aminoacyl-tRNA synthetase family | LOC104230233 |
