# Supplementary material for: Cucumber Mosaic Virus Coat Protein Sequesters Host CDPK7‐Like Into Phase‐Separated Condensates to Promote Viral Infection
Source: Mol Plant Pathol. 2026 May 18;27(5):e70270. doi: 10.1111/mpp.70270 (PMC13181337; doi:10.1111/mpp.70270)
Supplement: Supplementary file 10 — Table S2: Differentially expressed genes in N. benthamiana leaves expressing CMV CP versus the empty‐vector control. [file MPP-27-e70270-s012.docx]

**Table S2** Differentially expressed genes in *N. benthamiana* leaves expressing CMV CP versus the empty-vector control.

| Gene Accession | Protein Description | Gene Id | Result |
| --- | --- | --- | --- |
| Niben101Scf05694g00009 | Belongs to the syntaxin family | SYP121_1 | up |
| Niben101Scf02069g00017 | WRKY transcription | LOC107813611 | up |
| Niben101Scf15752g00002 | calcium-dependent protein kinase 7-like | LOC107815101 | up |
| Niben101Scf01212g03005 | Pathogenesis-related genes transcriptional activator | LOC107807573 | up |
| Niben101Scf01395g00017 | UDP-glucoronosyl and UDP-glucosyl transferase | LOC107768655 | up |
| Niben101Scf06976g06002 | WRKY transcription factor | LOC107801001 | up |
| Niben101Scf00700g09001 | Cation-independent O- methyltransferase family 1 | HOMT1_2 | up |
| Niben101Scf03555g01001 | Eukaryotic aspartyl protease | LOC107825731 | up |
| Niben101Scf07042g01006 | Short calmodulin-binding motif containing conserved Ile and Gln residues. | LOC107789694 | up |
| Niben101Scf01958g01006 | Belongs to the GRAS family | LOC107764158 | up |
| Niben101Scf08321g01005 | WRKY transcription factor | LOC107761907 | up |
| Niben101Scf01649g04003 | Uncharacterized protein | A4A49_19781 | up |
| Niben101Scf03877g00018 | A Receptor for Ubiquitination Targets | LOC104243453 | up |
| Niben101Scf04133g02009 | EF-hand domain | LOC107778507 | up |
| Niben101Scf01125g02022 | C1 domain | LOC107830887 | up |
| Niben101Scf02825g00007 | ATP synthase delta (OSCP) subunit | ATPD_0 | up |
| Niben101Scf01022g02010 | Huntingtin-interacting protein | LOC107831008 | up |
| Niben101Scf00817g04005 | WRKY transcription factor | LOC107761907 | up |
| Niben101Scf01252g03001 | zinc-finger of the FCS-type, C2-C2 | LOC107764254 | up |
| Niben101Scf07311g00005 | Transcription factor DIVARICATA-like | LOC107819472 | up |
| Niben101Scf03839g12003 | Cytochrome b5-like Heme/Steroid binding domain | SLD2 | down |
| Niben101Scf00439g10056 | Leucine-rich repeat | A4A49_12627 | up |
| Niben101Scf02069g00025 | Belongs to the WD repeat SEC13 family | NbSeh1b | up |
| Niben101Scf03114g03011 | Heat shock protein | HSP83A_2 | up |
| Niben101Scf07527g03002 | GH3 auxin-responsive promoter | GH3.5_1 | up |
| Niben101Scf00206g00034 | RWD | A4A49_35267 | up |
| Niben101Scf07063g00001 | WRKY transcription | WRKY53_2 | up |
| Niben101Scf01143g11007 | Exo70 exocyst complex subunit | LOC104212371 | up |
| Niben101Scf04098g00003 | Sigma factor binding protein | LOC104233345 | up |
| Niben101Scf06819g02006 | 40S ribosomal protein S24 | LOC104237075 | up |
| Niben101Scf01481g01006 | F-box domain | LOC107764020 | up |
| Niben101Scf13254g00003 | Serine hydrolase | A4A49_27001 | up |
| Niben101Scf08873g01022 | Ferric reduction oxidase 8 | A4A49_39556 | up |
| Niben101Scf08873g01026 | U-box domain-containing protein | PUB52_1 | up |
| Niben101Scf02694g06025 | uncharacterized protein | LOC104246539 | up |
| Niben101Ctg16115g00003 | WRKY transcription factor | wizz | up |
| Niben101Scf01696g05014 | Prefoldin subunit 4 | AIP3 | up |
| Niben101Scf01476g00023 | 60S ribosomal protein L31-like | LOC107780457 | up |
| Niben101Scf09577g01001 | Lipase (class 3) | LOC107828088 | up |
| Niben101Scf06650g03009 | Acetyltransferase (GNAT) domain | LOC104223027 | up |
| Niben101Scf01660g02014 | UDP-glucoronosyl and UDP-glucosyl transferase | LOC107767710 | up |
| Niben101Scf03600g01010 | calcium-binding protein CML19 | LOC107805895 | up |
| Niben101Scf04988g02019 | domain-containing protein | LOC104234225 | up |
| Niben101Scf02816g01009 | Regulator of Vps4 activity in the MVB pathway | A4A49_30386 | up |
| Niben101Scf00063g12003 | - | #N/A | up |
| Niben101Scf04252g01008 | Auxin-responsive protein | AUX22D_0 | up |
| Niben101Scf04053g01008 | Auxin-responsive protein | LOC107809429 | up |
| Niben101Scf02411g00007 | Receptor-like serine/threonine-protein kinase | A4A49_06488 | up |
| Niben101Scf02562g01010 | shikimate | LOC104244757 | up |
| Niben101Scf06245g01017 | synthase | LOC107778627 | up |
| Niben101Scf00996g02002 | Protodermal factor | LOC107795275 | down |
| Niben101Scf00577g01002 | Carboxylesterase family | LOC107767338 | up |
| Niben101Scf12954g01001 | Probable xyloglucan endotransglucosylase/hydrolase protein | XTH | down |
| Niben101Scf01013g01003 | Myb-like DNA-binding domain | LOC104216616 | up |
| Niben101Scf01574g00002 | 9-divinyl ether synthase-like | LOC107829877 | down |
| Niben101Scf06447g00005 | 40S ribosomal protein | - | up |
| Niben101Scf01942g04001 | WRKY transcription factor | LOC107807916 | up |
| Niben101Scf00370g03012 | phototropism protein | LOC104220252 | up |
| Niben101Scf04122g03021 | Myb-like DNA-binding domain | LOC107788569 | up |
| Niben101Scf10053g00011 | Belongs to the mitochondrial carrier (TC 2.A.29) family | PNC1_0 | up |
| Niben101Scf10306g00003 | WRKY transcription factor | WRKY50 | up |
| Niben101Scf00573g00010 | protein phosphatase 2C 25 | A4A49_39253 | up |
| Niben101Scf01817g02017 | Belongs to the glucose-6-phosphate 1-epimerase family | LOC107777417 | up |
| Niben101Scf00669g00008 | UDP-glucoronosyl and UDP-glucosyl transferase | UGT73C3_3 | up |
| Niben101Scf01412g00018 | DnaJ molecular chaperone homology domain | LOC107775089 | up |
| Niben101Scf00175g02011 | Belongs to the UDP-glycosyltransferase family | #N/A | up |
| Niben101Scf02698g00008 | Glutamate decarboxylase | LOC107789472 | down |
| Niben101Scf01241g02001 | defensin J1-2-like | LOC107772553 | down |
| Niben101Scf04490g00001 | Heat shock cognate 70 kDa | Ahy_B06g081994 | up |
| Niben101Scf05938g01050 | uncharacterized protein | LOC107780506 | up |
| Niben101Scf07462g01001 | Cytochrome P450 | LOC107811594 | down |
| Niben101Scf13823g03007 | Xyloglucan endotransglucosylase/hydrolase | LOC107817239 | down |
| Niben101Scf01942g05021 | MRN-interacting protein | LOC104234576 | up |
| Niben101Scf09282g00004 | Heavy-metal-associated domain | LOC107793358 | up |
| Niben101Scf08130g00014 | Belongs to the GST superfamily | LOC107813571 | up |
| Niben101Scf00352g02001 | MYB-CC type transfactor, LHEQLE motif | LOC107812672 | up |
| Niben101Scf05749g02030 | RING-like zinc finger | RHY1A_1 | up |
| Niben101Scf01128g00004 | Domain of unknown function (DUF4408) | LOC107803683 | up |
| Niben101Scf02597g03006 | Glutathione S-transferase, C-terminal domain | TCHQD | up |
| Niben101Scf07070g01015 | Glycosyl transferase family 21 | CSLC4 | up |
| Niben101Scf00682g04013 | Belongs to the serine threonine dehydratase family | TD | down |
| Niben101Scf02322g07001 | Belongs to the cytochrome P450 family | LOC104230322 | up |
| Niben101Scf02023g01005 | zinc-finger of the FCS-type, C2-C2 | LOC107764254 | up |
| Niben101Scf02002g01004 | ubiquitin | UPL5_0 | down |
| Niben101Scf01982g03006 | Homocysteine S-methyltransferase | LOC104245742 | up |
| Niben101Scf03706g00007 | late embryogenesis abundant protein | LOC104245543 | up |
| Niben101Scf00501g00004 | Myb-like DNA-binding domain | lbm4 | up |
| Niben101Scf06358g00012 | Belongs to the peptidase S10 family | SCPL42_2 | up |
| Niben101Scf04424g00001 | Belongs to the iron ascorbate-dependent oxidoreductase family | GA3ox2 | down |
| Niben101Scf02514g01004 | Heavy-metal-associated domain | LOC107824923 | up |
| Niben101Scf00297g00013 | Myelodysplasia-myeloid leukemia factor 1-interacting protein | LOC107802727 | up |
| Niben101Scf01478g05006 | POT family | LOC107829632 | up |
| Niben101Scf09523g02006 | Protodermal factor | LOC107795275 | down |
| Niben101Scf03321g04008 | ankyrin repeat-containing protein | LOC107765492 | up |
| Niben101Scf02494g01018 | 21 kDa protein-like isoform X1 | LOC107812028 | down |
| Niben101Scf08178g00008 | 21 kDa protein-like | LOC107801092 | up |
| Niben101Scf03739g06015 | C2C2 Zinc finger | LOC104211715 | up |
| Niben101Scf01897g03007 | KR domain | SDR2A_0 | up |
| Niben101Scf01453g06001 | Belongs to the peroxidase family | LOC107813838 | down |
| Niben101Scf06603g03002 | WRKY transcription factor | LOC107762610 | up |
| Niben101Scf04234g02011 | 3-ketoacyl-CoA synthase | LOC107807886 | up |
| Niben101Scf02124g01026 | Belongs to the protein kinase superfamily | LOC104232367 | up |
| Niben101Scf00715g09001 | WRKY transcription factor | wizz | up |
| Niben101Scf00127g03002 | trihelix transcription factor ASIL1-like | LOC107804172 | up |
| Niben101Scf01440g02013 | KR domain | LOC104219566 | down |
| Niben101Scf09116g04018 | Belongs to the SNF7 family | LOC104210691 | up |
| Niben101Scf02755g06016 | Belongs to the heat shock protein 70 family | BIP4_1 | up |
| Niben101Scf05191g00013 | DHHC palmitoyltransferase | PAT19 | up |
| Niben101Scf03923g03003 | heat shock factor | HSFA6B_3 | up |
| Niben101Scf02430g03006 | DNA binding domain | NbWRKY7 | up |
| Niben101Scf03860g03001 | Transcription factor myb1r1 | MY1R1_18 | up |
| Niben101Scf01685g09002 | EF-hand domain pair | LOC107784779 | up |
| Niben101Scf03885g10002 | RING-type zinc-finger | A4A49_00774 | up |
| Niben101Scf02228g02003 | metal ion transport | A4A49_37711 | up |
| Niben101Scf03238g00012 | dentin sialophosphoprotein-like | LOC107762228 | up |
| Niben101Scf00661g03005 | RNA recognition motif | A4A49_31759 | up |
| Niben101Scf06493g00024 | Pollen proteins Ole e I like | LOC107767187 | up |
| Niben101Scf10938g00009 | Belongs to the short-chain dehydrogenases reductases (SDR) family | SDR1_8 | up |
| Niben101Scf08008g00006 | Metallothionein-like protein type 2 | mlp2 | down |
| Niben101Scf00927g08010 | Modified RING finger domain | - | up |
| Niben101Scf06017g02002 | Myb-like DNA-binding domain | lbm4 | up |
| Niben101Scf01589g00002 | Protein of unknown function (DUF563) | LOC107786439 | up |
| Niben101Scf03422g01002 | Belongs to the MIP aquaporin (TC 1.A.8) family | LOC104234650 | down |
| Niben101Scf18955g00004 | MYB-CC type transfactor, LHEQLE motif | LOC104210771 | up |
| Niben101Scf11689g03003 | domain-containing protein | A4A49_27272 | up |
| Niben101Scf04911g00004 | NUDIX domain | LOC107792661 | up |
| Niben101Scf02050g01029 | receptor-like kinase | LOC107817279 | up |
| Niben101Scf00522g01017 | Myb-like DNA-binding domain | LOC107827213 | up |
| Niben101Scf03231g00007 | Dehydrin | ECPP44 | up |
| Niben101Scf00175g02019 | Belongs to the UDP-glycosyltransferase family | NTGT1a | up |
| Niben101Scf00970g02011 | Protein kinase domain | LOC104236717 | up |
| Niben101Scf06280g01016 | Belongs to the glycosyltransferase 8 family | LOC104221990 | up |
| Niben101Scf06562g01010 | Neprosin | LOC104217006 | up |
| Niben101Scf01998g03009 | Short calmodulin-binding motif containing conserved Ile and Gln residues. | LOC107805112 | up |
| Niben101Scf01110g00002 | Protein radialis-like 1 | RL1_2 | up |
| Niben101Scf02300g00025 | paramyosin-like | LOC104215852 | up |
| Niben101Scf01376g04027 | pirin-like protein | LOC107763309 | up |
| Niben101Scf39295g00002 | Leucine rich repeat N-terminal domain | LRR1 | down |
| Niben101Scf01642g03014 | rRNA processing | LOC107806786 | up |
| Niben101Scf00307g01019 | Wall-associated receptor kinase C-terminal | LOC107828977 | up |
| Niben101Scf01870g03002 | Low affinity sulfate transporter 3-like | LOC107766288 | up |
| Niben101Scf15817g01009 | Belongs to the UDP-glycosyltransferase family | UGT73A25 | up |
| Niben101Scf00905g01002 | Transferase family | LOC107782061 | down |
| Niben101Scf00107g02002 | Belongs to the cyclin family | CYCD3-1_2 | up |
| Niben101Scf05060g07008 | EF-hand domain | LOC107778507 | up |
| Niben101Scf07508g08003 | ubiquitin-protein transferase activity | A4A49_39077 | up |
| Niben101Scf05855g06014 | Ethylene-responsive transcription factor ERF039-like | LOC107822488 | down |
| Niben101Scf00069g14014 | Protein kinase C conserved region 2 (CalB) | CAR3 | up |
| Niben101Scf04036g04007 | Domain associated at C-terminal with AAA | LOC104232292 | up |
| Niben101Scf00616g02010 | Phosphoglycerate mutase family | LOC107766036 | up |
| Niben101Scf12672g00005 | ubiquitin-protein transferase activity | LOC107776374 | up |
| Niben101Scf12308g00011 | Sarcoplasmic reticulum histidine-rich calcium-binding protein | LOC107792799 | up |
| Niben101Scf03341g01002 | uncharacterized protein LOC107797315 | LOC107797315 | up |
| Niben101Scf01777g02001 | Heat shock factor protein HSF30 | LOC104245420 | up |
| Niben101Scf00883g02011 | Conserved gene of | LOC107785472 | up |
| Niben101Scf08111g02011 | PLAC8 family | LOC107806230 | up |
| Niben101Scf00750g00005 | Potato inhibitor I family | LOC107827891 | down |
| Niben101Scf08804g02014 | NUDIX domain | LOC107792661 | up |
| Niben101Scf00984g09007 | Modified RING finger domain | - | up |
| Niben101Scf01297g04006 | DNA binding domain | NbWRKY7 | up |
| Niben101Scf01635g00005 | Zinc finger ccch domain-containing protein 40 | A4A49_15347 | up |
| Niben101Scf09928g01023 | Belongs to the protein kinase superfamily | LOC104232367 | up |
| Niben101Scf03779g06005 | Transcription factor DIVARICATA-like | LOC107819472 | up |
| Niben101Scf02904g03003 | Guanylate kinase | LOC107776379 | up |
| Niben101Scf00953g04021 | Uncharacterized protein | A4A49_07662 | up |
| Niben101Scf01977g00001 | WRKY transcription factor | LOC104245013 | up |
| Niben101Scf00369g21005 | Phloem protein 2 | PP2B15_2 | up |
| Niben101Scf00448g18001 | glycine-rich cell wall structural protein | LOC104214809 | down |
| Niben101Scf05298g00004 | GDP-fucose protein O-fucosyltransferase | LOC107818021 | up |
| Niben101Scf06078g02011 | Lycopene cyclase protein | LOC107823278 | up |
| Niben101Scf05787g01001 | heat shock factor | HSFA4B | up |
| Niben101Scf00841g02021 | Belongs to the MIP aquaporin (TC 1.A.8) family | LOC104246595 | down |
| Niben101Scf02362g02014 | DNA binding domain | NbWRKY8 | up |
| Niben101Scf02537g18002 | Protein of unknown function (DUF740) | #N/A | up |
| Niben101Scf07309g00004 | Allene oxide synthase | LOC107832778 | down |
| Niben101Scf13773g00014 | UDP-galactose UDP-glucose transporter 4-like | UTR4_1 | up |
| Niben101Scf03830g02010 | - | #N/A | up |
| Niben101Scf00016g03004 | transcription, DNA-templated | LOC107789148 | up |
| Niben101Scf07953g01009 | Belongs to the mitochondrial carrier (TC 2.A.29) family | AAC3_0 | up |
| Niben101Scf03985g02025 | - | #N/A | up |
| Niben101Scf05133g06002 | epoxide hydrolase | EH1.1 | up |
| Niben101Scf07201g01009 | probable mitochondrial chaperone BCS1-B | LOC104224261 | up |
| Niben101Scf00215g02002 | Uncharacterized protein | A4A49_15415 | up |
| Niben101Scf06344g00005 | Belongs to the UDP-glycosyltransferase family | UGT73A25 | up |
| Niben101Scf00735g06023 | Stress-associated protein | LOC107799902 | up |
| Niben101Scf01297g04003 | Domain of unknown function (DUF588) | A4A49_10131 | up |
| Niben101Scf09445g04018 | Ankyrin repeat-containing protein | LOC107790749 | up |
| Niben101Scf17398g00012 | Nucleotide binding leucine rich repeat protein | ZAR1 | up |
| Niben101Scf01920g00006 | #N/A | #N/A | up |
| Niben101Scf00109g07005 | Polcalcin Jun o | LOC102601867 | up |
| Niben101Scf07194g00014 | Septum-promoting GTP-binding protein 1-like | LOC104233556 | up |
| Niben101Scf01922g10003 | Golgin subfamily A member 6-like protein | LOC107805530 | up |
| Niben101Scf03985g04006 | WRKY transcription | LOC107813611 | up |
| Niben101Scf02264g03012 | Protein of unknown function (DUF3741) | LOC104245709 | up |
