# Supplementary material for: Cucumber Mosaic Virus Coat Protein Sequesters Host CDPK7‐Like Into Phase‐Separated Condensates to Promote Viral Infection
Source: Mol Plant Pathol. 2026 May 18;27(5):e70270. doi: 10.1111/mpp.70270 (PMC13181337; doi:10.1111/mpp.70270)
Supplement: Supplementary file 11 — Table S3: Antiviral activities of target compounds D1–D34 against CMV in vivo. [file MPP-27-e70270-s022.docx]

**Table S3** Antiviral activities of target compounds **D1** − **D34** against CMV *in vivo****^a^*.**

| Compd. | Curative activity (%) | Protective activity (%) | Inactivating activity (%) | EC_50_ of Inactivating activity (*μ*g/mL) |
| --- | --- | --- | --- | --- |
| **D1** | 47.6±4.5 | 44.0±1.4 | 77.4±2.9 | 95.8±3.4 |
| **D2** | 35.0±1.6 | 21.3±1.3 | 66.2±1.9 | 172±7.7 |
| **D3** | 58.7±3.6 | 61.4±1.0 | 87.3±2.3 | 70.8±2.2 |
| **D4** | 68.5±1.8 | 31.9±2.9 | 74.7±1.5 | 106±2.8 |
| **D5** | 47.9±2.4 | 62.0±3.3 | 71.7±3.2 | 126±4.3 |
| **D6** | 48.9±4.5 | 33±2.7 | 59.4±1.1 | 232±6.8 |
| **D7** | 55.5±4.6 | 53.0±3.6 | 80.8±2.4 | 96.6±3.3 |
| **D8** | 42.4±3.9 | 35.2±3.3 | 34.6±1.5 | 1860±80 |
| **D9** | 43.5±4.5 | 35.6±1.9 | 82.3±1.4 | 85.1±2.1 |
| **D10** | 54.8±4.9 | 56.3±3.2 | 83.2±2.5 | 85.2±1.9 |
| **D11** | 59.0±4.2 | 46.2±2.8 | 42.1±1.6 | 1020±11 |
| **D12** | 57.3±4.3 | 35.2±4.9 | 53.1±2.2 | 386±18 |
| **D13** | 53.3±4.9 | 58.2±2.2 | 75.1±1.2 | 105±4.8 |
| **D14** | 48.5±4.8 | 30.3±4.3 | 71.7±1.1 | 127±5.8 |
| **D15** | 62.3±4.8 | 42.3±3.3 | 79.6±1.4 | 96.2±4.4 |
| **D16** | 68.3±4.3 | 54.7±4.4 | 43.7±0.8 | 1030±46 |
| **D17** | 50.1±0.9 | 50.8±2.5 | 55.1±1.5 | 450±8.2 |
| **D18** | 34.3±3.0 | 64.1±3.2 | 80.2±2.1 | 97.3±4.3 |
| **D19** | 53.2±5.0 | 56.3±2.9 | 63.1±1.6 | 194±6.0 |
| **D20** | 50.3±4.5 | 41.7±1.9 | 52.3±2.4 | 397±16.4 |
| **D21** | 46.9±2.6 | 62.0±2.3 | 52.7±1.2 | 406±5.1 |
| **D22** | 55.7±4.8 | 55.9±3.4 | 62.3±2.5 | 192±9.6 |
| **D23** | 54.8±2.1 | 56.7±4.9 | 86.1±1.4 | 73.7±5.3 |
| **D24** | 10.8±1.1 | 25.5±4.6 | 37.3±1.8 | 1700±80 |
| **D25** | 52.8±1.7 | 48.3±4.4 | 55.5±2.2 | 363±14 |
| **D26** | 33.5±4.5 | 31.9±3.1 | 63.2±1.5 | 183±6.2 |
| **D27** | 60.4±3.9 | 62.8±4.4 | 49.2±2.4 | 511±15 |
| **D28** | 43.7±4.0 | 18.6±5.0 | 62.4±1.3 | 194±5.2 |
| **D29** | 52.4±4.4 | 42.2±4.7 | 63.4±4.3 | 181.7±7.5 |
| **D30** | 22.6±4.8 | 52.2±4.7 | 50.1±4.2 | 408±10 |
| **D31** | 26.9±4.2 | 51.7±4.6 | 51.1±4.9 | 401±8.2 |
| **D32** | 45.1±4.8 | 58.1±2.1 | 60.7±4.5 | 204±11.2 |
| **D33** | 48.1±3.4 | 49.3±4.5 | 55.2±4.0 | 360±8.7 |
| **D34** | 38.1±3.2 | 61.5±1.2 | 59.5±3.5 | 210±9.2 |
| **Ribavirin***^b^* | 41.2±2.5 | 43.7±4.2 | 60.5±3.8 | 195±8.6 |
| *^a^*Average of three replicates. *^b^*Ribavirin was used as a control. | | | | |

Table S3 shows the results of the bioactivity assays for treatment, protection, and neutralization of all compounds. Most of the target compounds exhibited excellent assay results. Structure-Activity Relationships (SARs). SAR analysis indicated that the anti-CMV activities of coumarin derivatives were influenced by the nature of different mercaptan substituents. In terms of curative activity, when X = N, R^2^ was n-Bu, the activities of the compounds were better than that of Et and n-Pr, for example, D3 > D1, D2; D10 > D8, D9. When X = CH, R^2^ was n-Bu, the activities of the compounds were lower than that of Et and n-Pr, for example, D15, D16 > D17; D22, D23 > D24. In terms of protective activity, when X = N, R^2^ was n-Bu, the compounds activities were better than Et and n-Pr, for example, D3 > D1, D2; D10 > D8, D9. When X = CH, R^2^ was n-Bu, and the activities of the compounds were lower than that of Et and n-Pr, for example, D22, D23 > D24 (except D17). In terms of inactivating activity, when X = N and R^2^ were n-Bu, the activities of the compounds were better than that of Et and n-Pr, for example, D3 > D1, D2; D10 > D8, D9. When X = CH, R^2^ was n-Bu, and the activities of the compounds were lower than that of Et and n-Pr, for example, D22, D23 > D24 (except D17). It is worth noting that when X = N and R^2^ were n-Bu, the activities of the compounds were generally higher than that of short chain Et and n-Pr. When X = CH and R^2^ were n-Bu, the activities of most compounds were lower than that of short chain Et and n-Pr.
