# Supplementary material for: Cucumber Mosaic Virus Coat Protein Sequesters Host CDPK7‐Like Into Phase‐Separated Condensates to Promote Viral Infection
Source: Mol Plant Pathol. 2026 May 18;27(5):e70270. doi: 10.1111/mpp.70270 (PMC13181337; doi:10.1111/mpp.70270)
Supplement: Supplementary file 12 — Table S4: Total energy, HOMO, LUMO, energy gap, dipole moment, TPSA of D24, D3, and ribavirin. [file MPP-27-e70270-s009.docx]

**Table S4** Total Energy, HOMO, LUMO, energy gap, dipole moment, TPSA of **D24**, **D3**, and ribavirin.

| **Parameter** | **D24** | **D3** | **RBV** |
| --- | --- | --- | --- |
| *E*total/hartree | -2451.952 | –2415.725 | –907.195 |
| *E*_HOMO_/hartree | –0.207 | –0.204 | –0.266 |
| *E*_LUMO_/hartree | –0.064 | –0.077 | –0.046 |
| △E/hartree | 0.143 | 0.127 | 0.220 |
| μ(Debye) | 9.893 | 9.863 | 7.206 |
| TPSA/Å² | 71.060 | 83.420 | 140.970 |
