# Supplementary material for: Cucumber Mosaic Virus Coat Protein Sequesters Host CDPK7‐Like Into Phase‐Separated Condensates to Promote Viral Infection
Source: Mol Plant Pathol. 2026 May 18;27(5):e70270. doi: 10.1111/mpp.70270 (PMC13181337; doi:10.1111/mpp.70270)
Supplement: Supplementary file 14 — Table S6: Acute oral toxicity of D3 to Italian honeybees. [file MPP-27-e70270-s019.docx]

**Table S6** Acute oral toxicity of **D3** to Italian honeybees.

| Drug Processing | Set Concentration (*µ*g a.i./bee) | Total Number of Bees | Number of Deaths | | Mortality Rate (48h) |
| --- | --- | --- | --- | --- | --- |
|  |  |  | 24h | 48h |  |
| **D3** | 11 | 30 | 0 | 0 | 0 |
|  | 2 | 30 | 0 | 0 | 0 |
|  | 0.3 | 30 | 0 | 0 | 0 |
|  | 0.05 | 30 | 0 | 0 | 0 |
| **CK_1_(DMSO)** | 0 | 30 | 0 | 0 | 0 |
| **CK_2_(Water)** | 0 | 30 | 0 | 0 | 0 |
