# Supplementary material for: Cucumber Mosaic Virus Coat Protein Sequesters Host CDPK7‐Like Into Phase‐Separated Condensates to Promote Viral Infection
Source: Mol Plant Pathol. 2026 May 18;27(5):e70270. doi: 10.1111/mpp.70270 (PMC13181337; doi:10.1111/mpp.70270)
Supplement: Supplementary file 15 — Table S7: Protein identification results from the DARTS assay. [file MPP-27-e70270-s015.docx]

**Table S7** Protein identification results from the DARTS assay

| **Protein accession** | **Protein description** | **Organism** | **Length** |
| --- | --- | --- | --- |
| A0A067XHD6 | Capsid protein (CP) | Cucumber mosaic virus | 218 |
| Q9YJS3 | Capsid protein (CP) | Cucumber mosaic virus | 218 |
| Q66132 | Capsid protein (CP) | Cucumber mosaic virus | 173 |
| A0A0U3T3E9 | Capsid protein (CP) | Cucumber mosaic virus | 218 |
| Q8JPX1 | Capsid protein (CP) | Cucumber mosaic virus | 218 |
| Q9MB25 | Capsid protein (CP) | Cucumber mosaic virus | 100 |
| X5I0N3 | Genome polyprotein | Zucchini yellow mosaic virus | 3080 |
| A0A0K0QQ16 | Capsid protein (CP) | Cucumber mosaic virus | 218 |
| A0A140H4X3 | Capsid protein (CP) | Cucumber mosaic virus | 218 |
| A9EES5 | Capsid protein (CP) | Cucumber mosaic virus | 218 |
| A0A067XHD6 | Capsid protein (CP) | Cucumber mosaic virus | 218 |
