# Supplementary material for: Cucumber Mosaic Virus Coat Protein Sequesters Host CDPK7‐Like Into Phase‐Separated Condensates to Promote Viral Infection
Source: Mol Plant Pathol. 2026 May 18;27(5):e70270. doi: 10.1111/mpp.70270 (PMC13181337; doi:10.1111/mpp.70270)
Supplement: Supplementary file 16 — Table S8: Primer sequence used for RT‐qPCR. [file MPP-27-e70270-s029.docx]

**Table S8** Primer sequence used for RT-qPCR.

| **Primer** | **Sequence** |
| --- | --- |
| CMV CP-F | GTCTTGTCGCAGCAGCTTTC |
| CMV CP-R | GTGTACCCAGGTCTACAGCG |
| PVV CP-F | TGGCGAGGTTCCATTTTCA |
| PVV CP-R | CATAGGAGAAACTGAGATGCCAACT |
| PMMoV CP-F | GACGAGGCGGGTAGATGATG |
| PMMoV CP-R | AGTTGTAGCCCAGGTGAGTC |
| RBOH-F | AGCTCAAAGAGTTCTGGGAT |
| RBOH-R | TTCCATTTTCCATTACTTCG |
| CDPK7-like-F | AGGAGGTGGCTGGAATAATGG |
| CDPK7-like-R | ACTTGATGTCCGAGCTTCTGTA |
| Actin-F | ATCGGAATGGAAGCTGCTGG |
| Actin-R | TCATCCTATCAGCAATGCCCG |
