# Supplementary material for: Cucumber Mosaic Virus Coat Protein Sequesters Host CDPK7‐Like Into Phase‐Separated Condensates to Promote Viral Infection
Source: Mol Plant Pathol. 2026 May 18;27(5):e70270. doi: 10.1111/mpp.70270 (PMC13181337; doi:10.1111/mpp.70270)
Supplement: Supplementary file 22 — Methods S1. Synthesis of intermediates and target compounds. [file MPP-27-e70270-s007.docx]

**Methods S1** Synthesis of intermediates and target compounds.

General synthetic procedure of intermediates **2a** – **2b**. According to literature reports, the intermediates **2a** – **2b** were prepared (Shaik *et al.,* 2016). The initial raw materials **1a** – **1b** (12mmol) and hexamine (50 mmol) were added into acetic acid (30 mL), heated to 90°C, reflux for 6h, and then 20% hydrochloric acid (30 mL) was added, the temperature was kept at 75 °C for 1h, and the reaction was stopped and cooled to room temperature. The mixture was extracted with ethyl acetate and then dried over anhydrous sodium sulfate. The intermediates **2a** – **2b** were subsequently purified by column chromatography.

General synthetic procedure of intermediates **3a** – **3b**, **4a** – **4d**. Intermediates **3a** – **3b** were prepared according to literature reports (Chen *et al.,* 2016; Wu *et al.,* 2010). K_2_CO_3_ (11 mmol) was added to a DMF (35 mL) solution of **2a** – **2b** (5.5 mmol), heated at 80°C for 1.5 hours, and then cooled to room temperature. Subsequently, **3a** – **3b** (5.5 mmol) and KI (5.5 mmol) were added, and the mixture was reheated to 80 °C. Thin layer chromatography (TLC) followed the reaction, the raw material completely disappeared and stopped the reaction and cooled to room temperature. Pour into ice water, adjust pH to acidity with dilute hydrochloric acid, filter cake, purify by column chromatography to obtain intermediates **4a** – **4d**.

Synthetic procedure of title compounds **D1** – **D34**. The target compounds were synthesized from intermediates **4a** – **4d** and various thiols following a previously reported method (Zhao *et al.,* 2020). Intermediates **4a** – **4d** (1 mmol) were dissolved in dichloromethane (30 mL), followed by the addition of thiols (2.5 mmol) and NaHSO_4_·SiO_2_ catalyst (5 mmol). The progress of the reaction was monitored using TLC. Once the starting materials were completely consumed, the reaction was terminated, and the target compounds were isolated and purified by column chromatography.

**References**

Chen, Y., H. Lu, H. Dai, W. S. Yu, and X. H. Pan. 2016. “New Manufacturing Route to Picoxystrobin.” *Organic Process Research & Development* 20: 195-198.

Shaik, J. B., B. K. Palaka, M. Penumala, et al. 2016. “Synthesis, Biological Evaluation, and Molecular Docking of 8‐imino‐2‐oxo‐2H,8H‐pyrano[2,3‐f]chromene Analogs: New Dual AChE Inhibitors as Potential Drugs for the Treatment of Alzheimer's Disease” *Chemical Biology & Drug Design* 88: 43-53.

Wu, Q. Y., G. D. Wang, S. W. Huang, L. Lin, and G. F. Yang. 2010. “Synthesis and Biological Activity of Novel Phenyltriazolinone Derivatives.” *Molecules* 15: 9024-9034.

Zhao, L., J. Zhang, T. Liu, et al. 2020. “Design, Synthesis, and Antiviral Activities of Coumarin Derivatives Containing Dithioacetal Structures.” *Journal of Agricultural and Food Chemistry* 68: 975-981.
