# Supplementary material for: Cucumber Mosaic Virus Coat Protein Sequesters Host CDPK7‐Like Into Phase‐Separated Condensates to Promote Viral Infection
Source: Mol Plant Pathol. 2026 May 18;27(5):e70270. doi: 10.1111/mpp.70270 (PMC13181337; doi:10.1111/mpp.70270)
Supplement: Supplementary file 23 — Methods S2. Antiviral bioassay. [file MPP-27-e70270-s026.docx]

**Methods S2** Antiviral bioassay.

The virus was extracted and its activity tested according to previously reported methods (Zhao *et al.,* 2022). Viruses were propagated in *N. tabacum* cv. K326, ground in 0.5M phosphate buffer (0.01M EDTA, 0.1% *β*-ME, 2% Triton X-100) and filtered with a double layer pledget. The extract was centrifuged for 5 min at 10000×g, and the supernatant was used as the crude extract of the virus. The extraction process was carried out at 4°C.

Curative activities of target compounds against CMV *in vivo*. The uniform growth of *C. amaranticolor* at the 5 - 6 leaves stage was selected to test its anti - CMV activity. Sprinkle silicon carbide on the leaves and inoculate CMV virus. After 0.5h-1h, rinse with water and dry naturally. The water was spread on the left side of the leaves of *C. amaranticolor* as a negative control, the liquid medicine was evenly spread on the right side of the leaves. After the *C. amaranticolor* were cultivated in a phytotron (illumination of 10 000 lx, 28 ± 1 °C). After 3-5 days, the number of dead spots on leaves was counted, and the measurement was repeated 3 times.

Protective activities of target compounds against CMV *in vivo*. The uniform growth of *C. amaranticolor* at the 5-6 leaves stage was selected to test its anti-CMV activity. The water was spread on the left side of the leaves of *C. amaranticolor* as a negative control, the liquid medicine was evenly spread on the right side of the leaves. After 24h, evenly sprinkle silicon carbide on the leaves and inoculate CMV virus. After 0.5h-1h, rinse with water and dry naturally. After the *C. amaranticolor* were cultivated in a phytotron (illumination of 10 000 lx, 28 ± 1 °C). After 3-5 days, the number of dead spots on leaves was counted, and the measurement was repeated 3 times.

Inactive activities of target compounds against CMV *in vivo*. The uniform growth of *C. amaranticolor* at the 5-6 leaves stage was selected to test its anti - CMV activity. Add the compound solution to the same volume of CMV (6 × 10^−3^ mg/mL) virus solution and mix for 30 minutes. Sprinkle silicon carbide on the leaves, inoculate CMV virus (1.2 × 10^−2^ mg/mL) on the left side of the leaves as a control, and inoculate the mixture on the right side. After 0.5h-1h, rinse with water and dry naturally. After the plants were cultivated in a phytotron (illumination of 10 000 lx, 28 ± 1 °C). After 3-5 days, the number of dead spots on leaves was counted, and the measurement was repeated 3 times.

**Reference**

Zhao, L., D. Y. Hu, Z. X. Wu, C. L. Wei, S. Wu, and B. A. Song. 2022. “Coumarin Derivatives Containing Sulfonamide and Dithioacetal Moieties: Design, Synthesis, Antiviral Activity, and Mechanism.” *Journal of Agricultural and Food Chemistry* 70: 5773-5783.
