# Supplementary material for: Cucumber Mosaic Virus Coat Protein Sequesters Host CDPK7‐Like Into Phase‐Separated Condensates to Promote Viral Infection
Source: Mol Plant Pathol. 2026 May 18;27(5):e70270. doi: 10.1111/mpp.70270 (PMC13181337; doi:10.1111/mpp.70270)
Supplement: Supplementary file 24 — Methods S3. Molecular dynamics (MD) simulations. [file MPP-27-e70270-s030.docx]

**Methods S3** Molecular dynamics (MD) simulations.

To investigate the stable binding conformation of compounds with CMV CP, molecular dynamics (MD) simulations were conducted using the docking results of ligands and proteins as starting structures. Ligand atomic charges were assigned using the AM1-BCC method within the Antechamber module of Amber22 (Jakalian *et al.,* 2002). The complex’s topology and coordinate files were generated through the Leap module. Amino acid residues were described by the AMBER ff14SB force field, while ligands and neutralizing ions (Cl⁻ or Na⁺) employed the general AMBER force field (GAFF) (Maier *et al.,* 2015). The entire system was solvated in a rectangular TIP3P water box, extending at least 10 Å from the solute in all directions (Price *et al.,* 2004). To assess the stability of the complex throughout the simulation, root mean square deviation (RMSD) analyses was performed using the Cpptraj module in Amber22.

**References**

Jakalian, A., D. B. Jack, and CI. Bayly. 2002. “Fast, Efficient generation of High-Quality Atomic Charges. AM1-BCC Model: II. Parameterization and Validation.” *Journal of Computational Chemistry* 23: 1623-1641.

Maier, J. A., C. Martinez, K. Kasavajhala, L. Wickstrom, K. E. Hauser, and C. Simmerling. 2015. “ff14SB: Improving the Accuracy of Protein Side Chain and Backbone Parameters from ff14SB.” *Journal of Chemical Theory and Computation* 11: 3696-3713.

Price, D. J., and C. L. Brooks. 2004. “A Modified tip3p Water Potential for Simulation with Ewald Summation.” *The Journal of Chemical Physics* 121: 10096-10103.
