# Supplementary material for: Cucumber Mosaic Virus Coat Protein Sequesters Host CDPK7‐Like Into Phase‐Separated Condensates to Promote Viral Infection
Source: Mol Plant Pathol. 2026 May 18;27(5):e70270. doi: 10.1111/mpp.70270 (PMC13181337; doi:10.1111/mpp.70270)
Supplement: Supplementary file 25 — Methods S4. Bee toxicity assays. [file MPP-27-e70270-s016.docx]

**Methods S4** Bee toxicity assays.

In this study, Italian honeybees (*Apis mellifera* L.) were used to assess acute contact and oral toxicity (Rasuli *et al.,* 2017). For the contact toxicity test, different concentrations of the test compound were dissolved in DMSO, and 1 μL was applied to each bee. Blank (CK1) and solvent (CK2) controls were included, with 10 bees per group and three replicates. Mortality was recorded at 24 and 48 hours. In the oral toxicity test, the compound was dissolved in DMSO and diluted with 5% honey solution to four dose levels. Bees, fasted for two hours, were fed 200 μL of the solution. Control groups were similarly included, and survival was recorded at 24 and 48 hours.

**Reference**

Rasuli, F., J. N. Rafie, and A. Sadeghi. 2017. “Acute Contact Toxicity of Six Pesticides in Honeybees (Apis Mellifera Meda) in Iran.” *Journal of Apicultural Science* 61: 29-36.
