# Supplementary material for: Cucumber Mosaic Virus Coat Protein Sequesters Host CDPK7‐Like Into Phase‐Separated Condensates to Promote Viral Infection
Source: Mol Plant Pathol. 2026 May 18;27(5):e70270. doi: 10.1111/mpp.70270 (PMC13181337; doi:10.1111/mpp.70270)
Supplement: Supplementary file 26 — Methods S5. DARTS assay. [file MPP-27-e70270-s010.docx]

**Methods S5** DARTS assay.

To verify whether compound **D3** directly binds to CMV viral proteins, we employed the DARTS technique to identify potential targets (Lomenick *et al.,* 2009).

Protein extraction. CMV-infected Nicotiana tabacum leaves were homogenized on ice in 0.5 M phosphate buffer (pH 7.4). After centrifugation at 12,000 × g for 15 min at 4 °C, the supernatant was collected and further lysed using a non-denaturing lysis buffer. The lysate was concentrated, and total protein concentration was determined using the BCA method, then adjusted to 1 mg/mL.

Compound treatment. A total of 47 μL of protein solution was mixed with 1 μL of compound **D3** at final concentrations of 25, 50, 100, 250, 500, 1000, and 2000 μM. The control group received an equal volume of DMSO. Samples were incubated at 25 °C for 1 hour.

Limited proteolysis. After incubation, 2 μL of Pronase E was added to each sample, with final enzyme-to-protein ratios ranging from 0% to 2% (w/w). Proteolysis was performed at 37 °C for 20 min. The reaction was terminated by adding SDS-PAGE loading buffer and boiling at 95 °C for 10 min.

Detection and target identification. Digested samples were resolved by SDS-PAGE. Portions of the gels were transferred to PVDF membranes for immunoblotting using anti-CMV CP antibodies, while others were stained with Coomassie Brilliant Blue. Differential bands enhanced by **D3** treatment were excised, subjected to in-gel trypsin digestion, and analyzed via LC-MS/MS. The identified target was confirmed as CMV capsid protein (CP).

**Reference**

Lomenick, B., R. Hao, N. Jonai, et al. 2009. “Target Identification Using Drug Affinity Responsive Target Stability (DARTS).” *Proceedings of the National Academy of Sciences of the United States of America* 106: 21984-21989.
