# Supplementary material for: Cucumber Mosaic Virus Coat Protein Sequesters Host CDPK7‐Like Into Phase‐Separated Condensates to Promote Viral Infection
Source: Mol Plant Pathol. 2026 May 18;27(5):e70270. doi: 10.1111/mpp.70270 (PMC13181337; doi:10.1111/mpp.70270)
Supplement: Supplementary file 27 — Methods S6. Purification of proteins. [file MPP-27-e70270-s005.docx]

**Methods S6** Purification of proteins.

The wild-type and mutant CMV CP proteins were purified following the procedures described in the literature (Zhang *et al.,* 2016). The target plasmid was introduced into *E. coli* BL21 (DE3) cells and cultured in LB medium supplemented with 30 μg/mL kanamycin at 37°C until the OD_600_ reached 0.65. Protein expression was initiated by adding 1 mM Isopropyl *β*-D-thiogalactoside (IPTG) and incubating at 16°C for 12–16 hours. The bacterial cells were then harvested by centrifugation at 6,000 rpm for 10 minutes at 4°C. The cell pellet was resuspended in lysis buffer (30 mM PBS, 300 mM NaCl, 1 mM *β*-mercaptoethanol, pH 7.4) and disrupted using ultrasonic treatment. The resulting lysate was centrifuged at 12,000 rpm for 20 minutes at 4°C to obtain the supernatant. This protein-containing solution was loaded onto an Ni-NTA column for affinity purification, and the target protein was purified using an AKTA protein purification system with the following buffers: Buffer A (300 mM NaCl,30 mM PBS, 20 mM imidazole, 10% glycerol, pH 7.4) and Buffer B (300 mM NaCl, 30 mM PBS, 400 mM imidazole, 10% glycerol, pH 7.4). The purified protein was concentrated and desalted using SEC buffer (300 mM NaCl, 30 mM PBS, 10% glycerol, pH 7.4). Its concentration was measured using the Coomassie Brilliant Blue assay. Finally, the purified protein was analyzed and verified by 12% SDS-PAGE (sodium dodecyl sulfate-polyacrylamide gel electrophoresis). GFP-CMV CP, GFP-CDPK7-like, and CDPK7-like proteins were purified using the same procedures.

**Reference**

Zhang, G. P., G. F. Hao, J. K. Pan, J. Zhang, D. Y. Hu, and B. A. Song. 2016. “Asymmetric Synthesis and Bioselective Activities of α-amino-phosphonates Based on the Dufulin Motif.” *Journal of Agricultural and Food Chemistry* 64: 4207-4213.
