# Supplementary material for: Cucumber Mosaic Virus Coat Protein Sequesters Host CDPK7‐Like Into Phase‐Separated Condensates to Promote Viral Infection
Source: Mol Plant Pathol. 2026 May 18;27(5):e70270. doi: 10.1111/mpp.70270 (PMC13181337; doi:10.1111/mpp.70270)
Supplement: Supplementary file 28 — Methods S7. Western blot. [file MPP-27-e70270-s028.docx]

**Methods S7** Western blot.

The leaves of the assayed *N. benthamiana* were used to extract total protein according to previously described (Li *et al.,* 2022). Approximately 0.1 g of tobacco leaf tissue was weighed into a 1.5 mL RNase-free centrifuge tube, rapidly frozen in liquid nitrogen, and fully ground using a tissue oscillator to ensure complete disruption. A volume of 0.35 mL protein extraction buffer (Yuanye Biotech, Cat#R21233) was then added, and the mixture was vortexed thoroughly. The homogenate was centrifuged at 12,000 rpm for 20 minutes at 4°C. The supernatant was transferred to a new tube and centrifuged again under the same conditions. The resulting supernatant, containing the total plant proteins, was collected for subsequent analysis. Protein samples were separated on 12% TGX Stain-Free FastCast Acrylamide gels (Bio-Rad, Cat#1610185) and electrotransferred onto PVDF membranes. Membranes were blocked in Tris-buffered saline containing 0.1% (v/v) Tween 20 (TBST) and 5% (w/v) non-fat milk for 1 hour at room temperature. After blocking, membranes were incubated with primary antibodies diluted in 5% (w/v) BSA in TBST for 3 hours at room temperature, followed by three washes with TBST. Subsequently, membranes were incubated with either anti-mouse IgG-HRP (Sangon Biotech, Cat#D110098) or anti-rabbit IgG-HRP (Sangon Biotech, Cat#D110058), diluted in TBST containing 1% (w/v) non-fat milk, for 1 hour. After three additional washes with TBST, protein bands were detected using the Clarity Western ECL Substrate (Bio-Rad, Cat#170-5060) and visualized with the Bio-Rad ChemiDoc MP Imaging System (Bio-Rad, RRID:SCR_019037).

**Reference**

Li, J., H. Feng, S. Liu, et al. 2022. “Phosphorylated Viral Protein Evades Plant Immunity Through Interfering the Function of RNA-binding Protein.” *Plos Pathogens* 18: e1010412.
