# Supplementary material for: Cucumber Mosaic Virus Coat Protein Sequesters Host CDPK7‐Like Into Phase‐Separated Condensates to Promote Viral Infection
Source: Mol Plant Pathol. 2026 May 18;27(5):e70270. doi: 10.1111/mpp.70270 (PMC13181337; doi:10.1111/mpp.70270)
Supplement: Supplementary file 29 — Methods S8. Biolayer interferometry (BLI) assays. [file MPP-27-e70270-s006.docx]

**Methods S8** Biolayer interferometry (BLI) assays.

Biolayer interferometry (BLI) detection. The purified His-tagged CDPK7-like protein was loaded onto Ni-NTA biosensors, and the binding signal values were measured by Octet RED 96 (FortéBio, USA) using the biolayer interferometry method. All CMV CP and CMV CP^T52A^ were diluted with PBST. The binding affinity (KD) between CDPK7-like and wild-type and mutant proteins (100 nM, 50 nM, 25 nM, 12.5 nM, 6.25 nM, and 3.125 nM) was determined using Octet BLI Discovery 12.2 (FortéBio, USA). The experimental binding curves were globally fitted and analyzed by Octet Analysis Studio 12.2 (FortéBio, USA) to determine the binding constants of CDPK7-like with wild and mutant CP proteins. The binding constant (KD) values were calculated by Octet Red software.
