# Supplementary material for: Cucumber Mosaic Virus Coat Protein Sequesters Host CDPK7‐Like Into Phase‐Separated Condensates to Promote Viral Infection
Source: Mol Plant Pathol. 2026 May 18;27(5):e70270. doi: 10.1111/mpp.70270 (PMC13181337; doi:10.1111/mpp.70270)
Supplement: Supplementary file 30 — Text S1: Characterisation data of intermediates and target compounds. [file MPP-27-e70270-s025.docx]

**Text S1** Characterization data of intermediates and target compounds.

Methyl(*E*)-2-(2-(((8-formyl-2-oxo-2H-chromen-7-yl)oxy)methyl)phenyl)-2-(methoxyimino)acetate (4a). Yellow solid; yield 62 %; m.p. 213 − 214 ℃; ^1^H NMR (500 MHz, CDCl_3_) *δ* 10.64 (s, 1H, -CHO), 7.66 − 7.62 (m, 2H, Ar-H), 7.57 (d, *J* = 8.7 Hz, 1H, Ar-H), 7.49 − 7.46 (m, *J* = 7.6, 1.4 Hz, 1H, Ar-H ), 7.44 − 7.40 (m, *J* = 7.6, 1.4 Hz, 1H, Ar-H), 7.22 (dd, *J* = 7.5, 1.4 Hz, 1H, Ar-H), 6.89 (d, *J* = 8.8 Hz, 1H, Ar-H), 6.33 (d, *J* = 9.5 Hz, 1H, Ar-H), 5.16 (s, 2H, -OCH_2_-), 4.04 (s, 3H, =N-OCH_3_), 3.88 (s, 3H, -CO-OCH_3_). ^13^C NMR (126 MHz, CDCl_3_) *δ* 186.76, 163.33, 162.14, 159.54, 155.81, 149.09, 143.05, 134.06, 133.76, 130.06, 129.00, 128.68, 128.34, 127.57, 114.35, 113.18, 112.98, 109.52, 69.50, 64.07, 53.34. HRMS (ESI): calcd for C_21_H_17_O_7_N ([M+Na]^+^), 418.09009; found, 418.08972.

Methyl(*E*)-2-(2-(((8-formyl-4-methyl-2-oxo-2H-chromen-7-yl)oxy)methyl)phenyl)-2-(methoxyimino)acetate (4b). Yellow solid; yield 61.5 %; m.p. 126 − 127℃. ^1^H NMR (400 MHz, CDCl_3_) *δ* 10.67 (s, 1H, -CHO), 7.67 (d, *J* = 9.1 Hz, 2H, Ar-H), 7.49 − 7.45 (m, 1H, Ar-H), 7.44 − 7.39 (m, 1H, Ar-H), 7.21 (dd, *J* = 7.5, 1.5 Hz, 1H, Ar-H), 6.89 (d, *J* = 9.0 Hz, 1H, Ar-H), 6.21 (s, 1H, Ar-H), 5.17 (s, 2H, -OCH_2_-), 4.05 (s, 3H, =N-OCH_3_), 3.88 (s, 3H, -CO-OCH_3_), 2.40 (s, 3H, -CH_3_). ^13^C NMR (101 MHz, CDCl_3_) *δ* 186.98, 163.25, 161.63, 159.51, 155.37, 151.89, 148.98, 133.77, 130.67, 129.95, 128.78, 128.51, 128.15, 127.35, 113.94, 113.04, 112.88, 109.08, 69.24, 63.96, 53.24, 18.91. HRMS (ESI): calcd for C_22_H_19_O_7_N ([M+Na]^+^), 432.10574; found, 432.10537.

Methyl(*E*)-2-(2-(((8-formyl-2-oxo-2H-chromen-7-yl)oxy)methyl)phenyl)-3-methoxyacrylate (4c). Yellow solid; yield 67 %; m.p. 154 − 155 ℃. ^1^H NMR (500 MHz, CDCl_3_) *δ* 10.68 (s, 1H, -CHO), 7.63 (s, 1H, -C=CH-O-), 7.61 (d, *J* = 10.0 Hz, 1H, Ar-H), 7.58 − 7.56 (m, 1H, Ar-H), 7.52 (d, *J* = 10.0 Hz, 1H, Ar-H), 7.36 − 7.34 (m, 2H, Ar-H), 7.20 − 7.18 (m, 1H, Ar-H), 6.86 (d, *J* = 10.0Hz, 1H, Ar-H), 6.31 (d, *J* = 10.0 Hz, Ar-H), 5.20 (s, 2H, -OCH_2_-), 3.85 (s, 3H, =N-OCH_3_), 3.72 (s, 3H, -CO-OCH_3_). ^13^C NMR (126 MHz, CDCl_3_) *δ* 186.94, 167.63, 162.60, 160.52, 159.56, 155.57, 143.00, 134.37, 133.92, 131.24, 130.86, 128.36, 127.98, 127.02, 114.05, 113.04, 112.60, 109.71, 109.63, 69.38, 62.19, 51.82. HRMS (ESI): calcd for C_22_H_18_O_7_ ([M+Na]^+^), 417.09476; found, 417.09447.

Methyl(*E*)-2-(2-(((8-formyl-4-methyl-2-oxo-2H-chromen-7-yl)oxy)methyl)phenyl)-3-methoxyacrylate (4d). Yellow solid; yield 64 %; m.p. 170 − 171 ℃. ^1^H NMR (500 MHz, CDCl_3_) *δ* 10.70 (s, 1H, -CHO), 7.65-7.63 (d, *J* = 10.0 Hz, 1H, Ar-H), 7.64 (s, 1H, -C=CH-O-), 7.58 − 7.56 (m, 1H, Ar-H), 7.36 − 7.34 (m, 2H, Ar-H), 7.20 − 7.18 (m, 1H, Ar-H), 6.86 (d, *J* =10.0 Hz, 1H, Ar-H), 6.20 (s, 1H, Ar-H), 5.21 (s, 2H, -OCH_2_-), 3.86 (s, 3H, =N-OCH_3_), 3.73 (s, 3H, -CO-OCH_3_), 2.39 (s, 3H, -CH_3_). ^13^C NMR (126 MHz, CDCl_3_) *δ* 187.28, 167.67, 162.14, 160.55, 159.66, 155.26, 151.98, 134.44, 131.17, 130.95, 130.68, 128.35, 127.90, 126.86, 113.63, 112.93, 112.70, 109.54, 109.36, 69.17, 62.20, 51.85, 18.93. HRMS (ESI): calcd for C_23_H_20_O_7_ ([M+Na]^+^), 431.11029; found, 431.11012.

Methyl(*E*)-2-(2-(((8-(bis(ethylthio)methyl)-2-oxo-2H-chromen-7-yl)oxy)methyl)phenyl)-2-(methoxyimino)acetate (D1). White solid; yield 83 %; m.p. 103 − 104 ℃. ^1^H NMR (500 MHz, CDCl_3_) *δ* 7.92 (d, *J* = 7.7 Hz, 1H, Ar-H), 7.61 (d, *J* = 9.5 Hz, 1H, Ar-H), 7.46 − 7.43 (m, 1H, Ar-H), 7.38 − 7.35 (m, 1H, Ar-H), 7.25 (d, *J* = 8.8 Hz, 1H, Ar-H), 7.17 (dd, *J* = 7.5, 1.3 Hz, 1H, Ar-H), 6.72 (d, *J* = 8.7 Hz, 1H, Ar-H), 6.25 (d, *J* = 9.4 Hz, 1H, Ar-H), 5.68 (s, 1H, -SCHS-), 5.21 (s, 2H, -OCH_2_-), 4.10 (s, 3H, =N-OCH_3_), 3.91 (s, 3H, -CO-OCH_3_), 2.76 − 2.69 (m, 4H, -SCH_2_-), 1.32 (t, *J* = 7.4 Hz, 6H, -CH_3_). ^13^C NMR (126 MHz, CDCl_3_) *δ* 163.45, 160.51, 159.64, 150.28, 149.09, 143.99, 134.77, 130.03, 127.93, 127.84, 127.56, 127.06, 118.32, 113.29, 112.66, 110.07, 68.38, 64.11, 53.34, 43.23, 28.32, 14.82. HRMS (ESI): calcd for C_25_H_27_O_6_NS_2_ ([M+Na]^+^), 524.11737; found, 524.11720.

Methyl(*E*)-2-(2-(((8-(bis(propylthio)methyl)-2-oxo-2H-chromen-7-yl)oxy)methyl)phenyl)-2-(methoxyimino)acetate (D2). Yellow liquid; yield 76%; m.p. 71 − 72 ℃. ^1^H NMR (400 MHz, CDCl_3_) *δ* 7.95 (d, *J* = 7.8 Hz, 1H, Ar-H), 7.61 (d, *J* = 9.5 Hz, 1H, Ar-H), 7.46 − 7.42 (m, 1H, Ar-H), 7.36 (t, *J* = 6.9 Hz, 1H, Ar-H), 7.24 (d, *J* = 8.6 Hz, 1H, Ar-H), 7.17 (d, *J* = 7.7 Hz, 1H, Ar-H), 6.72 (d, *J* = 8.6 Hz, 1H, Ar-H), 6.25 (d, *J* = 9.4 Hz, 1H, Ar-H), 5.64 (s, 1H, -SCHS-), 5.21 (s, 2H, -OCH_2_-), 4.10 (s, 3H, =N-OCH_3_), 3.91 (s, 3H, -CO-OCH_3_), 2.68 (m, 4H, -SCH_2_-), 1.69 (m, 4H, -SCH_2_CH_2_-), 0.98 (t, *J* = 7.4 Hz, 6H, -SCH_2_CH_2_CH_3_). ^13^C NMR (101 MHz, CDCl_3_) *δ* 163.46, 160.52, 159.66, 150.30, 149.10, 144.00, 134.80, 130.02, 127.91, 127.83, 127.54, 127.10, 118.35, 113.27, 112.63, 110.07, 68.36, 64.11, 53.35, 43.82, 36.38, 23.01, 13.69. HRMS (ESI): calcd for C_27_H_31_O_6_NS_2_ ([M+Na]^+^), 552.14880; found, 552.14850.

Methyl(*E*)-2-(2-(((8-(bis(butylthio)methyl)-2-oxo-2H-chromen-7-yl)oxy)methyl)phenyl)-2-(methoxyimino)acetate (D3). Yellow liquid; yield 75 %; m.p. 55 − 56 ℃. ^1^H NMR (400 MHz, CDCl_3_) *δ* 7.95 (dd, *J* = 7.9, 1.2 Hz, 1H, Ar-H), 7.61 (d, *J* = 9.5 Hz, 1H, Ar-H), 7.46 − 7.41 (m, *J* = 7.7, 1.5 Hz, 1H, Ar-H), 7.38 − 7.34 (m, *J* = 7.5, 1.3 Hz, 1H, Ar-H), 7.24 (d, *J* = 8.7 Hz, 1H, Ar-H), 7.17 (dd, *J* = 7.7, 1.3 Hz, 1H, Ar-H), 6.72 (d, *J* = 8.7 Hz, 1H, Ar-H), 6.25 (d, *J* = 9.5 Hz, 1H, Ar-H), 5.65 (s, 1H, -SCHS-), 5.21 (s, 2H, -OCH_2_-), 4.10 (s, 3H, =N-OCH_3_), 3.91 (s, 3H, -CO-OCH_3_), 2.73 − 2.68 (m, *J* = 7.0 Hz, 4H, -SCH_2_-), 1.66 − 1.59 (m, 4H, -SCH_2_CH_2_-), 1.42 − 1.37 (m, 4H, -SCH_2_CH_2_CH_2_-), 0.89 (t, *J* = 7.4 Hz, 6H, -SCH_2_CH_2_CH_2_CH_3_)*.* ^13^C NMR (101 MHz, CDCl_3_) *δ* 163.33, 160.35, 159.53, 150.20, 148.99, 143.86, 134.69, 129.89, 129.83, 127.79, 127.71, 127.41, 126.97, 118.24, 113.15, 112.52, 109.93, 68.24, 63.99, 53.22, 43.82, 33.96, 31.56, 22.03, 13.68. HRMS (ESI): calcd for C_29_H_35_O_4_NS_2_ ([M+Na]^+^), 580.17981; found, 580.17980.

Methyl(*E*)-2-(2-(((8-(bis(cyclohexylthio)methyl)-2-oxo-2H-chromen-7-yl)oxy)methyl)phenyl)-2-(methoxyimino)acetate (D4). White solid; yield 91 %; m.p. 63 − 64 ℃. ^1^H NMR (500 MHz, CDCl_3_) *δ* 7.97 (d, *J* =10.0 Hz, 1H, Ar-H), 7.60 (d, *J* = 10.0 Hz, 1H, Ar-H), 7.42 (t, *J* =10.0 Hz, 1H, Ar-H), 7.35 (t, *J* =10.0 Hz, 1H, Ar-H), 7.25 − 7.1 (m, 2H, Ar-H), 6.69 (d, *J* = 10 Hz, 1H, Ar-H), 6.24 (d, *J* =10 Hz, 1H, Ar-H), 5.81 (s, 1H, -SCHS-), 5.22 (s, 2H, -OCH_2_-), 4.10 (s, 3H, =N-OCH_3_), 3.91 (s, 3H, -CO-OCH_3_), 2.81 − 2.76 (m, 2H, Cyclohexyl-H), 2.20 − 1.24 (m, 20H, Cyclohexyl-H). ^13^C NMR (126 MHz, CDCl_3_) *δ* 163.37, 160.31, 159.57, 149.89, 149.02, 143.92, 134.82, 129.86, 127.68, 127.66, 127.60, 127.36, 127.04, 119.16, 113.07, 112.47, 110.00, 68.15, 64.00, 53.23, 45.71, 33.72, 33.67, 26.06, 25.81. HRMS (ESI): calcd for C_33_H_39_O_6_NS_2_ ([M+Na]^+^), 632.21161; found, 632.21110.

Methyl(*E*)-2-(2-(((8-(1,3-dithiolan-2-yl)-2-oxo-2H-chromen-7-yl)oxy)methyl)phenyl)-2-(methoxyimino)acetate (D5). Yellow solid; yield 82 %; m.p. 150 − 151 ℃. ^1^H NMR (400 MHz, CDCl_3_) *δ* 7.59 (dd, *J* = 9.3, 2.4 Hz, 2H, Ar-H), 7.49 − 7.40 (m, 2H, Ar-H), 7.28 − 7.21 (m, 1H, Ar-H), 6.76 (d, *J* = 8.7 Hz, 1H, Ar-H), 6.54 (s, 1H, Ar-H), 6.26 (d, *J* = 9.5 Hz, 1H, Ar-H), 5.30 (s, 1H, -SCHS-), 5.07 (s, 2H, -OCH_2_-), 4.08 (s, 3H, =N-OCH_3_), 3.88 (s, 3H, -CO-OCH_3_), 3.83 − 3.77 (m, *J* = 6.6, 3.7 Hz, 2H, -SCH2-), 3.41-3.33 (m, 2H, -SCH2-). ^13^C NMR (101 MHz, CDCl_3_) *δ* 163.37, 160.45, 158.73, 153.18, 149.06, 143.74, 134.32, 130.02, 129.12, 128.65, 128.23, 128.20, 127.98, 118.05, 113.63, 113.58, 109.21, 69.34, 64.15, 53.38, 42.50, 41.57. HRMS (ESI): calcd for C_23_H_21_O_6_NS_2_ ([M+Na]^+^), 494.07053; found, 494.07025.

Methyl(*E*)-2-(2-(((8-(bis(isopropylthio)methyl)-2-oxo-2H-chromen-7-yl)oxy)methyl)phenyl)-2-(methoxyimino)acetate (D6). Yellow solid, yield 84 %, m.p.112 − 113 ℃; ^1^H NMR (500 MHz, CDCl_3_) *δ* 7.95 (d, *J* = 10.0 Hz, 1H, Ar-H), 7.60 (d, *J* = 10.0 Hz, 1H, Ar-H), 7.45 − 7.41 (m, 1H, Ar-H), 7.35 (t, *J* = 10.0 Hz, 1H, Ar-H), 7.23 (d, *J* =10.0Hz, 1H, Ar-H), 7.17 (dd, *J* = 10.0, 5.0 Hz, 1H, Ar-H), 6.70 (d, *J* = 10.0 Hz, 1H, Ar-H), 6.25 (d, *J* = 10.0 Hz, 1H, Ar-H), 5.75 (s, 1H, -SCHS-), 5.23 (s, 2H, -OCH_2_-), 4.10 (s, 3H, =N-OCH_3_), 3.92 (s, 3H, -CO-OCH_3_), 3.09 − 3.01 (m, 2H, -SCH-), 1.39 (d, *J* = 5.0 Hz, 6H, -CH_3_), 1.29 (d, *J* = 10.0 Hz, 6H, -CH_3_).^13^C NMR (500 MHz, CDCl_3_) *δ* 163.34, 160.29, 159.46, 149.81, 148.98, 143.91, 134.75, 129.87, 127.67, 127.38, 126.94, 118.91, 113.11, 112.48, 109.96, 68.14, 64.00, 53.24, 40.66, 37.32, 23.57, 23.45. HRMS (ESI): calcd for C_27_H_31_O_6_NS_2_ ([M+Na]^+^), 552.14905; found, 552.14850.

Methyl(*E*)-2-(2-(((8-(bis(tert-butylthio)methyl)-2-oxo-2H-chromen-7-yl)oxy)methyl)phenyl)-2-(methoxyimino)acetate (D7). White solid, yield 86 %, m.p. 116 − 117 ℃; ^1^H NMR NMR (500 MHz, CDCl_3_) *δ* 8.05 (d, *J* = 10.0 Hz, 1H, Ar-H), 7.60 (d, *J* = 10.0 Hz, 1H, Ar-H), 7.46 − 7.43 (m, 1H, Ar-H), 7.36 (t, *J* = 8.0 Hz, 1H, Ar-H), 7.21 (d, *J* = 10.0 Hz, 1H, Ar-H), 7.17 (d, *J* = 10.0 Hz, 1H, Ar-H), 6.69 (d, *J* = 10.0 Hz, 1H, Ar-H), 6.25 (d, *J* = 10.0 Hz, 1H, Ar-H), 5.86 (s, 1H, -SCHS-), 5.23 (s, 2H, -OCH_2_-), 4.11 (s, 3H, =N-OCH_3_), 3.92 (s, 3H, -CO-OCH_3_), 1.36 (s, 18H, -CH_3_). ^13^C NMR (125 MHz, CDCl_3_) *δ* 163.35, 160.30, 159.39, 149.01, 148.91, 144.01, 134.87, 129.87, 127.62, 127.59, 127.35, 127.14, 127.11, 121.46, 112.92, 112.44, 110.07, 68.17, 64.01, 53.26, 45.71, 36.30, 31.00. HRMS (ESI): calcd for C_29_H_36_O_6_NS_2_ ([M+Na]^+^), 580.18030; found, 58017980.

Methyl(*E*)-2-(2-(((8-(bis(ethylthio)methyl)-4-methyl-2-oxo-2H-chromen-7-yl)oxy)methyl)phenyl)-2-(methoxyimino)acetate (D8). Yellow solid; yield 81 %; m.p. 125 − 126 ℃. ^1^H NMR (400 MHz, CDCl_3_) *δ* 7.92 (d, *J* = 8.0 Hz, 1H, Ar-H), 7.44 (t, *J* = 8.0 Hz, 1H, Ar-H), 7.37 (d, *J* = 8.0 Hz, 1H, Ar-H), 7.18 (d, *J* = 4.0 Hz, 1H, Ar-H), 6.73 (d, *J* = 8.0 Hz, 1H, Ar-H), 6.15 (s, 1H, Ar-H), 5.71 (s, 1H, -SCHS-), 5.21 (s, 2H, -OCH_2_-), 4.10 (s, 3H, =N-OCH_3_), 3.92 (s, 3H, -CO-OCH_3_), 2.79 − 2.65 (m, 4H, -SCH2-), 2.36 (s, 3H, -CH_3_), 1.31 (t, *J* = 7.4 Hz, 6H, -SCH_2_CH_3_). ^13^C NMR (101 MHz, CDCl_3_) *δ* 163.40, 160.42, 159.26, 152.83, 149.64, 148.98, 134.77, 129.91, 127.71, 127.41, 126.91, 124.44, 118.18, 113.57, 112.10, 109.58, 68.17, 64.01, 53.25, 43.37, 28.23, 18.86, 14.73. HRMS (ESI): calcd for C_26_H_29_O_6_NS_2_ ([M+Na]^+^), 538.13330; found, 538.13285.

Methyl(*E*)-2-(2-(((8-(bis(propylthio)methyl)-4-methyl-2-oxo-2H-chromen-7-yl)oxy)methyl)phenyl)-2-(methoxyimino)acetate (D9). Yellow solid; yield 86 %; m.p. 105 − 106℃. ^1^H NMR (500 MHz, CDCl_3_) *δ* 7.94 (d, *J* = 7.8 Hz, 1H, Ar-H), 7.45 − 7.40 (m, 1H, Ar-H), 7.37 (d, *J* = 9.0 Hz, 2H, Ar-H), 7.17 (d, *J* = 7.8 Hz, 1H, Ar-H), 6.73 (d, *J* = 8.8 Hz, 1H, Ar-H), 6.14 (s, 1H, -CH-), 5.67 (s, 1H, -SCHS-), 5.21 (s, 2H, -OCH_2_-), 4.10 (s, 3H, =N-OCH_3_), 3.92 (s, 3H, -CO-OCH_3_), 2.72 − 2.62 (m, 4H, -SCH_2_-), 2.36 (s, 3H, -CH_3_), 1.72 − 1.65 (m, 4H, -SCH_2_CH_2_-), 0.98 (t, *J* = 7.4 Hz, 6H, -SCH_2_CH_2_CH_3_). ^13^C NMR (126 MHz, CDCl_3_) *δ* 163.42, 160.42, 159.30, 152.83, 149.69, 149.01, 134.81, 129.91, 127.98, 127.71, 127.39, 126.96, 124.43, 118.23, 113.56, 112.10, 109.58, 68.16, 64.01, 53.25, 43.96, 36.29, 22.92, 18.86, 13.58. HRMS (ESI): calcd for C_28_H_33_O_6_NS_2_ ([M+Na]^+^), 566.16437; found, 566.16415.

Methyl(*E*)-2-(2-(((8-(bis(butylthio)methyl)-4-methyl-2-oxo-2H-chromen-7-yl)oxy)methyl)phenyl)-2-(methoxyimino)acetate (D10). Yellow solid; yield 87 %; m.p. 51 − 52 ℃. ^1^H NMR (400 MHz, CDCl_3_) *δ* 7.94 (dd, *J* = 7.8, 1.2 Hz, 1H, Ar-H), 7.45 − 7.41 (m, 1H, Ar-H), 7.38 (d, *J* = 1.7 Hz, 1H, Ar-H), 7.35 (d, *J* = 1.2 Hz, 1H, Ar-H), 7.17 (dd, *J* = 7.6, 1.4 Hz, 1H, Ar-H), 6.72 (d, *J* = 8.9 Hz, 1H, Ar-H), 6.14 (s, 1H, Ar-H), 5.68 (s, 1H, -SCHS-), 5.21 (s, 2H, -OCH_2_-), 4.10 (s, 3H, , =N-OCH_3_), 3.92 (s, 3H,, -CO-OCH_3_), 2.76 − 2.64 (m, 4H, -SCH_2_-), 2.36 (s, 3H, -CH_3_), 1.65 − 1.54 (m, 4H, -SCH_2_CH_2_-), 1.42 − 1.37 (m, 4H, -SCH_2_CH_2_CH_2_-), 0.89 (t, *J* = 7.4 Hz, 6H, -SCH_2_CH_2_CH_2_CH_3_). ^13^C NMR (101 MHz, CDCl_3_) *δ* 163.53, 160.50, 159.40, 152.91, 149.81, 149.12, 134.93, 130.03, 127.81, 127.50, 127.06, 124.53, 118.33, 113.67, 112.22, 109.67, 68.26, 64.13, 53.38, 44.19, 34.11, 31.70, 22.17, 18.99, 13.83. HRMS (ESI): calcd for C_30_H_37_O_6_NS_2_ ([M+Na]^+^), 594.19550; found, 594.19545.

Methyl(*E*)-2-(2-(((8-(bis(cyclohexylthio)methyl)-4-methyl-2-oxo-2H-chromen-7-yl)oxy)methyl)phenyl)-2-(methoxyimino)acetate (D11). Yellow solid, yield 83 %, m.p. 54 − 55 ℃; ^1^H NMR (500 MHz, CDCl_3_) *δ* 7.94 (d, *J* = 7.7 Hz, 1H, Ar-H), 7.40 − 7.36 (m, 1H, Ar-H), 7.33 − 7.30 (m, 2H, Ar-H), 7.14 (d, *J* = 7.6, 1.3 Hz, 1H, Ar-H), 6.67 (d, *J* = 9.0 Hz, 1H, Ar-H), 6.10 (s, 1H, Ar-H), 5.81 (s, 1H, -SCHS-), 5.20 (s, 2H, -OCH_2_-), 4.07(s, 3H, =N-OCH_3_), 3.89 (s, 3H, -CO-OCH_3_), 3.16 (s, 3H, -CH_3_), 2.80 − 2.72 (m, 2H, Cyclohexyl-H), 1.92 − 1.18 (m, 20H, Cyclohexyl-H). ^13^C NMR (126 MHz, CDCl_3_) *δ* 163.41, 160.29, 159.29, 152.82, 149.36, 149.01, 134.92, 129.84, 127.67, 127.64, 127.32, 126.99, 124.22, 119.12, 113.48, 112.02, 109.60, 68.05, 63.99, 53.23, 45.83, 40.14, 33.72, 36.69, 26.06, 25.82, 18.86. HRMS (ESI): calcd for C_34_H_41_O_6_NS_2_ ([M+Na]^+^), 646.22687; found, 646.22675.

Methyl(*E*)-2-(2-(((8-(1,3-dithiolan-2-yl)-4-methyl-2-oxo-2H-chromen-7-yl)oxy)methyl)phenyl)-2-(methoxyimino)acetate (D12). Yellow solid; yield 87 %; m.p. 123 − 124 ℃. ^1^H NMR (400 MHz, CDCl_3_) *δ* 7.59 (dd, *J* = 7.4, 1.0 Hz, 1H, Ar-H), 7.60-7.40 (m, 3H, Ar-H), 7.22 (dd, *J* = 7.5, 1.4 Hz, 1H, Ar-H), 6.77 (d, *J* = 8.9 Hz, 1H, Ar-H), 6.57 (s, 1H, Ar-H), 6.16 (d, *J* = 1.3 Hz, 1H, -SCHS-), 5.08 (s, 2H, -OCH_2_-), 4.08 (s, 3H, =N-OCH_3_), 3.89 (s, 3H, -CO-OCH_3_), 3.85 − 3.78 (m, 2H, -SCH_2_-), 3.40 − 3.33 (m, 2H, -SCH_2_-), 2.36 (s, 3H, -CH_3_). ^13^C NMR (101 MHz, CDCl_3_) *δ* 163.29, 160.40, 158.45, 152.53, 152.43, 148.95, 134.30, 129.89, 128.94, 128.50, 128.06, 127.79, 124.71, 117.78, 114.55, 112.38, 108.75, 69.12, 64.03, 53.27, 42.55, 41.45, 18.88. HRMS (ESI): calcd for C_24_H_23_O_6_NS_2_ ([M+Na]^+^), 506.08618; found, 508.08590.

Methyl(*E*)-2-(2-(((8-(bis(isopropylthio)methyl)-4-methyl-2-oxo-2H-chromen-7-yl)oxy)methyl)phenyl)-2-(methoxyimino)acetate (D13). Yellow solid; yield 81 %; m.p. 155 − 156 ℃. ^1^H NMR (500 MHz, CDCl_3_) *δ* 7.94 (d, *J* = 7.7 Hz, 1H, Ar-H), 7.44 − 7.41 (m, 1H, Ar-H), 7.34 (d, *J* =5.0 Hz, 2H, Ar-H), 7.17 (d, *J* = 10.0, 1H, Ar-H), 6.70 (d, *J* = 5.0 Hz, 1H, Ar-H), 6.14 (s, 1H, Ar-H ), 5.78 (s, 1H, -SCHS-), 5.23 (s, 2H, -OCH_2_-), 4.11 (s, 3H, =N-OCH_3_), 3.93 (s, 3H, -CO-OCH_3_), 3.08-3.00 (m, 2H, -SCH-), 2.36 (s, 3H, -CH_3_), 1.39 (d, *J* = 10.0 Hz, 6H, -SCHCH_3_), 1.28 (d, *J* = 5.0 Hz, 6H, -CH_3_). ^13^C NMR (500 MHz, CDCl_3_) *δ* 163.42, 160.32, 159.19, 152.83, 149.29, 148.98, 134.87, 129.87, 127.65, 127.35, 126.91, 124.28, 118.91, 113.51, 112.06, 109.58, 68.04, 64.02, 53.27, 40.94, 37.36, 23.61, 23.48, 18.88. HRMS (ESI): calcd for C_28_H_33_O_6_NS_2_ ([M+Na]^+^), 566.16455; found, 566.16415.

Methyl(*E*)-2-(2-(((8-(bis(tert-butylthio)methyl)-4-methyl-2-oxo-2H-chromen-7-yl)oxy)methyl)phenyl)-2-(methoxyimino)acetate (D14). White solid; yield 78 %; m.p. 160 − 161 ℃. ^1^H NMR (500 MHz, CDCl_3_) *δ* 8.05 (d, *J* = 10.0 Hz, 1H, Ar-H), 7.44 (t, *J* = 5.0 Hz, 1H, Ar-H), 7.35 (dd, *J* = 10.0, 5.0 Hz, 1H, Ar-H), 7.17 (d, *J* = 10.0 Hz, 1H, Ar-H), 6.69 (d, *J* = 10.0 Hz, 1H, Ar-H), 6.14 (s, 1H, Ar-H ), 5.89 (s, 1H, -SCHS-), 5.24 (s, 2H, -OCH_2_-), 4.11 (s, 3H, =N-OCH_3_), 3.93 (s, 3H, -CO-OCH_3_), 2.36 (s, 3H, -CH_3_), 1.35 (s, 18H, -SCCH_3_). ^13^C NMR (500 MHz, CDCl_3_) *δ* 163.52, 160.41, 159.22, 153.05, 149.12, 148.49, 135.09, 129.98, 127.71, 127.43, 127.22, 124.06, 121.54, 113.57, 112.02, 109.78, 68.18, 64.13, 53.40, 45.83, 36.65, 31.12, 31.04, 19.04. HRMS (ESI): calcd for C_30_H_37_O_6_NS_2_ ([M+Na]^+^), 594.19568; found, 594.19545.

Methyl(*E*)-2-(2-(((8-(bis(ethylthio)methyl)-2-oxo-2H-chromen-7-yl)oxy)methyl)phenyl)-3-methoxyacrylate (D15). Yellow solid; yield 91 %; m.p. 84 − 85 ℃. ^1^H NMR (500 MHz, CDCl_3_) *δ* 7.81 (d, *J* = 10.0 Hz, 1H, Ar-H), 7.65 (s, 1H, -C=CH-O-), 7.59 (d, *J* = 10.0 Hz, 1H, Ar-H), 7.35 − 7.29 (m, 2H, Ar-H), 7.18 (dd, *J* = 10.0, 5.0 Hz, 2H, Ar-H), 6.67 (d, *J* = 5.0Hz, 1H, Ar-H), 6.23 (d, *J* = 10.0 Hz, 1H, Ar-H), 5.69 (s, 1H, -SCHS-), 5.26 (s, 2H, -OCH_2_-), 3.88 (s, 3H, =N-OCH_3_), 3.75 (s, 3H, -CO-OCH_3_), 2.79 − 2.67 (m, 4H, -SCH_2_-), 1.32 (t, *J* =7.4 Hz, 6H, -SCH_2_CH_3_); ^13^C NMR (126 MHz, CDCl_3_) *δ* 167.74, 160.54, 160.48, 159.84, 150.17, 143.95, 135.32, 130.65, 129.78, 128.26 128.26, 127.77, 127.21, 126.53, 117.97, 112.93, 112.30, 110.07, 109.59, 68.52, 62.14, 51.82, 43.23, 28.21, 14.71. HRMS (ESI): calcd for C_26_H_28_O_6_S_2_ ([M+Na]^+^), 523.12250; found, 523.12195.

Methyl(*E*)-2-(2-(((8-(bis(propylthio)methyl)-2-oxo-2H-chromen-7-yl)oxy)methyl)phenyl)-3-methoxyacrylate (D16). Yellow solid; yield 85 %; m.p. 83 − 85 ℃. ^1^H NMR (500 MHz, CDCl_3_) *δ* 7.83 (d, *J* = 5.0 Hz, 1H, Ar-H), 7.66 (s, 1H, -C=CH-O-), 7.59 (d, *J* = 5.0 Hz, 1H, Ar-H), 7.35 − 7.28 (m, 2H, Ar-H), 7.20 − 7.15 (m, 2H, Ar-H), 6.68 (d, *J* = 5.0 Hz, 1H, Ar-H), 6.23 (d, *J* = 10.0 Hz, 1H, Ar-H), 5.65 (s, 1H, -SCHS-), 5.27 (s, 2H, -OCH_2_-), 3.88 (s, 3H, =N-OCH_3_), 3.75 (s, 3H, -CO-OCH_3_), 2.73 − 2.64 (m, 4H, -SCH_2_-), 1.72 − 1.65 (m, *J* = 7.4 Hz, 4H, -SCH_2_CH_2_-), 0.98 (t, *J* = 10.0 Hz, 6H, -SCH_2_CH_2_CH_3_). ^13^C NMR (126 MHz, CDCl_3_) *δ* 167.82, 160.60, 160.57, 159.95, 150.28, 144.05, 135.44, 130.72, 129.86, 128.33, 127.84, 127.28, 126.65, 118.09, 113.00, 112.36, 110.15, 109.68, 68.59, 62.23, 51.91, 43.89, 36.36, 23.00, 13.68. HRMS (ESI): calcd for C_28_H_32_O_6_S ([M+Na]^+^), 551.15381; found, 551.15325.

Methyl(*E*)-2-(2-(((8-(bis(butylthio)methyl)-2-oxo-2H-chromen-7-yl)oxy)methyl)phenyl)-3-methoxyacrylate (D17): Yellow solid; yield 84 %; m.p. 94 − 95 ℃. ^1^H NMR (500 MHz, CDCl_3_) *δ* 7.83 (d, *J* = 10.0 Hz, 1H, Ar-H), 7.65 (s, 1H, -C=CH-O-), 7.58 (d, *J* =5.0Hz, 1H, Ar-H), 7.35 − 7.28 (m, 2H, Ar-H), 7.20 − 7.15 (m, 2H, Ar-H), 6.67 (d, *J* =10.0Hz, 1H, Ar-H), 6.23 (d, *J* =10.0Hz, 1H, Ar-H), 5.65 (s, 1H, -SCHS-), 5.26 (s, 2H, -OCH_2_-), 3.88 (s, 3H, =N-OCH_3_), 3.75 (s, 3H, -CO-OCH_3_), 2.76 − 2.66 (m, 4H, -SCH_2_-), 1.68 − 1.56 (m, 4H, -SCH_2_CH_2_-), 1.44 − 1.38 (m, 4H, -SCH_2_CH_2_CH_2_-), 0.89 (t, *J* = 10.0 Hz, 6H, -SCH_2_CH_2_CH_2_CH_3_). ^13^C NMR (126 MHz, CDCl_3_) *δ* 167.73, 160.47, 159.86, 150.21, 143.92, 135.36, 130.63, 129.76, 128.24, 127.73, 127.18, 126.56, 118.02, 112.91, 112.27, 110.05, 109.60, 68.50, 62.14, 51.82, 43.93, 33.98, 31.59, 22.06, 13.69. HRMS (ESI): calcd for C_30_H_36_O_6_S_2_ ([M+Na]^+^), 579.18494; found, 579.18455.

Methyl(*E*)-2-(2-(((8-(bis(cyclohexylthio)methyl)-2-oxo-2H-chromen-7-yl)oxy)methyl)phenyl)-3-methoxyacrylate (D18). White solid; yield 76 %; m.p. 71 − 72 ℃. ^1^H NMR (500 MHz, CDCl_3_) *δ* 7.85 (dd, *J* = 10.0, 5.0 Hz, 1H, Ar-H), 7.65 (s, 1H, -C=CH-O-), 7.58 (d, *J* =10.0 Hz, 1H, Ar-H), 7.34 − 7.28 (m, 2H, Ar-H), 7.22 − 7.15 (m, 2H, Ar-H), 6.64 (d, *J* =10.0Hz, 1H, Ar-H), 6.22 (d, *J* =10.0 Hz, 1H, Ar-H), 5.81 (s, 1H, -SCHS-), 5.28 (s, 2H, -OCH_2_-), 3.88 (s, 3H, =N-OCH_3_), 3.75 (s, 3H, -CO-OCH_3_), 2.83 − 2.76 (m, 2H, Cyclohexyl-H), 1.96 − 1.22 (m, 20H, Cyclohexyl-H). ^13^C NMR (126 MHz, CDCl_3_) *δ* 167.75, 160.47, 160.44, 159.84, 149.87, 143.98, 135.44, 130.56, 129.70, 128.20, 127.54, 127.12, 126.61, 118.85, 112.81, 112.19, 110.07, 109.58, 68.37, 62.15, 51.83, 45.68, 33.72, 33.68, 26.08, 25.82. HRMS (ESI): calcd for C_34_H_40_O_6_S_2_ ([M+Na]^+^), 631.21619; found, 631.21585.

Methyl(*E*)-2-(2-(((8-(1,3-dithiolan-2-yl)-2-oxo-2H-chromen-7-yl)oxy)methyl)phenyl)-3-methoxyacrylate (D19): Light pink solid; yield 74 %; m.p. 58 − 59 ℃. ^1^H NMR (500 MHz, CDCl_3_) *δ* 7.65 (s, 1H, -C=CH-O-), 7.57 (d, *J* = 10.0 Hz, 1H, Ar-H), 7.54 − 7.52 (m, 1H, Ar-H), 7.36 − 7.34 (m, 2H, Ar-H), 7.24 (d, *J* = 10.0 Hz, 1H, Ar-H), 7.21 − 7.19 (m, 1H, Ar-H), 6.74 (d, *J* = 10.0 Hz, 1H, Ar-H), 6.59 (s, 1H, -SCHS-), 6.24 (d, *J* = 10.0 Hz, 1H, Ar-H), 5.12 (s, 2H, -OCH_2_-), 3.86 (s, 3H, =N-OCH_3_), 3.86-3.83 (m, 2H, -SCH_2_-), 3.73 (s, 3H, -CO-OCH_3_), 3.40 − 3.36 (m, 2H, -SCH_2_-).^13^C NMR (126 MHz, CDCl_3_) *δ* 167.66, 160.46, 158.90, 153.05, 143.71, 134.93, 131.20, 131.00, 128.28, 128.06, 127.86, 127.49, 117.71, 113.24, 113.19, 109.72, 109.15, 69.20, 62.21, 51.83, 42.42, 41.46. HRMS (ESI): calcd for C_24_H_22_O_6_S_2_ ([M+Na]^+^), 493.07541; found, 493.07500.

Methyl(*E*)-2-(2-(((8-(bis(isopropylthio)methyl)-2-oxo-2H-chromen-7-yl)oxy)methyl)phenyl)-3-methoxyacrylate (D20). Yellow solid; yield 81 %; m.p. 85 − 87 ℃. ^1^H NMR (400 MHz, CDCl_3_) *δ* 7.84 (dd, *J* = 8.0, 4.0 Hz, 1H, Ar-H), 7.66 (s, 1H, -C=CH-O-), 7.58 (d, *J* = 12.0 Hz, 1H, Ar-H), 7.35 − 7.28 (m, 2H, Ar-H), 7.23 − 7.15 (m, 2H, Ar-H), 6.65 (d, *J* = 8.0 Hz, 1H, Ar-H), 6.22 (d, *J* =12.0 Hz, 1H, Ar-H), 5.76 (s, 1H, -SCHS-), 5.28 (s, 2H, -OCH_2_-), 3.88 (s, 3H, =N-OCH_3_), 3.75 (s, 3H, -CO-OCH_3_), 3.09-2.99 (m, 2H, -SCH-), 1.40 (d, *J* = 4.0Hz, 6H, -SCHCH_3_), 1.29 (d, *J* = 4.0Hz, 6H, -SCHCH_3_). ^13^C NMR (100 MHz, CDCl_3_) *δ* 167.69, 160.47, 160.39, 159.74, 149.79, 143.97, 135.37, 130.57, 129.71, 128.17, 127.60, 127.13, 126.52, 118.61, 112.83, 112.20, 110.04, 109.52, 68.37, 62.15, 51.81, 40.71, 37.29, 23.58, 23.46. HRMS (ESI): calcd for C_28_H_32_O_6_S_2_ ([M+Na]^+^), 551.15363; found, 551.15325.

Methyl(*E*)-2-(2-(((8-(bis(tert-butylthio)methyl)-2-oxo-2H-chromen-7-yl)oxy)methyl)phenyl)-3-methoxyacrylate (D21). White solid; yield 83 %; m.p. 126 − 127 ℃. ^1^H NMR (500 MHz, CDCl_3_) *δ* 7.94 (d, *J* = 5.0 Hz, 1H, Ar-H), 7.66 (s, 1H, -C=CH-O-), 7.58 (d, *J* = 10.0 Hz, 1H, Ar-H), 7.35 − 7.29 (m, 2H, Ar-H), 7.17 (d, *J* = 5.0 Hz, 1H, Ar-H), 7.15 (d, *J* = 5.0 Hz, 1H, Ar-H), 6.64 (d, *J* = 5.0 Hz, 1H, Ar-H), 6.23 (d, *J* = 10.0 Hz, 1H, Ar-H), 5.87 (s, 1H, -SCHS-), 5.29 (s, 2H, -OCH_2_-), 3.89 (s, 3H, =N-OCH_3_), 3.75 (s, 3H, -CO-OCH_3_), 1.36 (s, 18H, -SCCH_3_). ^13^C NMR (126 MHz, CDCl_3_) *δ* 167.88, 160.60, 160.53, 159.78, 149.02, 144.18, 135.63, 130.63, 129.77, 128.29, 127.39, 127.23, 126.89, 121.28, 112.77, 112.28, 110.24, 109.67, 68.52, 62.27, 51.95, 45.76, 36.46, 31.12. HRMS (ESI): calcd for C_30_H_36_O_6_S_2_ ([M+Na]^+^), 579.18500; found, 579.18455.

Methyl(*E*)-2-(2-(((8-(bis(ethylthio)methyl)-4-methyl-2-oxo-2H-chromen-7-yl)oxy)methyl)phenyl)-3-methoxyacrylate (D22). Yellow solid; yield 76 %; m.p. 105 − 106 ℃. ^1^H NMR (500 MHz, CDCl_3_) *δ* 7.80 (dd, *J* = 10.0, 5.0 Hz, 1H, Ar-H), 7.65 (s, 1H, -C=CH-O-), 7.35 − 7.27 (m, 3H, Ar-H), 7.16 (d, *J* = 5.0 Hz, 1H, Ar-H), 6.68 (d, *J* = 9.0 Hz, 1H, Ar-H), 6.13 (s, 1H, Ar-H), 5.72 (s, 1H, -SCHS-), 5.27 (s, 2H, -OCH_2_-), 3.88 (s, 3H, =N-OCH_3_), 3.76 (s, 3H, -CO-OCH_3_), 2.79 − 2.67 (m, 4H, -SCH_2_-), 2.34 (s, 3H, -CH_3_), 1.32 (t, *J* = 10.0 Hz, 6H, -SCH_2_CH_3_). ^13^C NMR (126 MHz, CDCl_3_) *δ* 167.74, 160.52, 160.47, 159.57, 152.88, 149.64, 135.40, 130.63, 129.74, 128.24, 127.16, 126.48, 124.37, 117.93, 113.30, 111.90, 109.68, 109.59, 68.42, 62.15, 51.82, 43.47, 28.23, 18.84, 14.73. HRMS (ESI): calcd for C_27_H_30_O_6_S_2_ ([M+Na]^+^), 537.13800; found, 537.13760.

Methyl(*E*)-2-(2-(((8-(bis(propylthio)methyl)-4-methyl-2-oxo-2H-chromen-7-yl)oxy)methyl)phenyl)-3-methoxyacrylate (D23): Yellow solid; yield 79%; m.p. 78 − 79 ℃; ^1^H NMR (500 MHz, CDCl_3_) *δ* 7.83 (dd, *J* = 7.4, 1.7 Hz, 1H, Ar-H), 7.66 (s, 1H, Ar-H), 7.35 − 7.33 (m, 1H, Ar-H), 7.31 (d, *J* = 2.0 Hz, 1H, Ar-H), 7.30 − 7.27 (m, 1H, Ar-H), 7.16 (dd, *J* = 7.3, 1.7 Hz, 1H, Ar-H), 6.72 (dd, *J* = 8.9 Hz, 1H, Ar-H), 6.12 (d, *J* = 1.3 Hz, 1H, Ar-H), 5.68 (s, 1H, -SCHS-), 5.28 (s, 2H, -OCH_2_-), 3.88 (s, 3H, =N-OCH_3_), 3.76 (s, 3H, -CO-OCH_3_), 2.73 − 2.62 (m, 4H, -SCH_2_-), 2.35 (s, 3H, -CH_3_), 1.73 − 1.62 (m, 4H, -SCH_2_CH_2_-), 0.98 (t, *J* = 7.3 Hz, 6H, -SCH_2_CH_2_CH_3_). ^13^C NMR (126 MHz, CDCl_3_) *δ* 167.77, 160.55, 160.51, 159.57, 152.91, 149.66, 135.42, 130.62, 129.72, 128.25, 127.15, 126.50, 124.38, 117.91, 113.26, 111.88, 109.66, 109.55, 68.37, 62.16, 51.84, 43.99, 36.27, 22.91, 18.86, 13.60. HRMS (ESI): calcd for C_29_H_34_O_6_S_2_ ([M+Na]^+^), 565.16943; found, 565.16890.

Methyl(*E*)-2-(2-(((8-(bis(butylthio)methyl)-4-methyl-2-oxo-2H-chromen-7-yl)oxy)methyl)phenyl)-3-methoxyacrylate (D24). Yellow solid; yield 78 %; m.p. 56 − 57 ℃. ^1^H NMR (500 MHz, CDCl_3_) *δ* 7.80 (dd, *J* = 10.0, 5.0 Hz, 1H, Ar-H), 7.64 (s, 1H, -C=CH-O-), 7.35 − 7.26 (m, 3H, Ar-H), 7.14 (dd, *J* = 10.0, 5.0 Hz, 1H, Ar-H), 6.66 (d, *J* = 10.0 Hz, 1H, Ar-H), 6.10 (s, 1H, Ar-H), 5.66 (s, 1H, -SCHS-), 5.25 (s, 2H, -OCH_2_-), 3.86 (s, 3H, =N-OCH_3_), 3.74 (s, 3H, -CO-OCH_3_), 2.74-2.63 (m, 4H, -SCH_2_-), 2.33 (s, 3H, -CH_3_), 1.65 − 1.56 (m, 4H, -SCH_2_CH_2_-), 1.39 (m, 4H, -SCH_2_CH_2_CH_2_-), 0.87 (t, *J* = 10.0 Hz, 6H, -SCH_2_CH_2_CH_2_CH_3_). ^13^C NMR (126 MHz, CDCl_3_) *δ* 167.75, 160.47, 159.58, 152.83, 149.67, 135.44, 130.61, 129.72, 128.23, 127.14, 126.50, 124.34, 117.96, 113.27, 111.89, 109.65, 109.59, 68.39, 62.14, 51.82, 44.15, 33.98, 31.59, 22.07, 18.84, 13.69. HRMS (ESI): calcd for C_31_H_38_O_6_S_2_ ([M+Na]^+^), 593.20050; found, 593.20020.

Methyl(*E*)-2-(2-(((8-(bis(cyclohexylthio)methyl)-4-methyl-2-oxo-2H-chromen-7-yl)oxy)methyl)phenyl)-3-methoxyacrylate (D25). Yellow solid; yield 83 %; m.p. 153 − 154 ℃. ^1^H NMR (500 MHz, CDCl_3_) *δ* 7.85 − 7.84 (m, 1H, Ar-H), 7.65 (s, 1H, -C=CH-O-), 7.35 − 7.32 (m, 1H, Ar-H), 7.30 − 7.27 (m, 2H, Ar-H), 7.16 − 7.15 (m, 1H, Ar-H), 6.65 (d, *J* = 10.0 Hz, 1H, Ar-H), 6.12 (s, 1H, Ar-H), 5.84 (s, 1H, -SCHS-), 5.28 (s, 2H, -OCH_2_-), 3.88 (s, 3H, =N-OCH_3_), 3.76 (s, 3H, -CO-OCH_3_), 3.09 (s, 3H, -CH_3_), 2.81 − 2.75 (m, 2H, Cyclohexyl-H), 2.34 − 1.23 (m, 20H, Cyclohexyl-H). ^13^C NMR (126 MHz, CDCl_3_) *δ* 167.76, 160.46, 160.43, 159.58, 152.87, 149.35, 135.54, 130.56, 129.69, 128.18, 127.08, 126.58, 124.15, 118.84, 113.20, 111.81, 109.68, 109.60, 68.29, 62.15, 51.83, 45.70, 40.19, 33.72, 26.08, 25.84, 18.85. HRMS (ESI): calcd for C_34_H_40_O_6_S_2_ ([M+Na]^+^), 645.23151; found, 645.23150.

Methyl(*E*)-2-(2-(((8-(1,3-dithiolan-2-yl)-4-methyl-2-oxo-2H-chromen-7-yl)oxy)methyl)phenyl)-3-methoxyacrylate (D26). White solid; yield 79 %; m.p. 150 − 151 ℃. ^1^H NMR (500 MHz, CDCl_3_) *δ* 7.65 (s, 1H, -C=CH-O-), 7.54 − 7.52 (m, 1H, Ar-H), 7.38 − 7.33 (m, 3H, Ar-H), 7.21 − 7.19 (m, 1H, Ar-H), 6.75 (d, *J* = 10.0 Hz, 1H, Ar-H), 6.62 (s, 1H, Ar-H), 6.14 (s, 1H, -SCHS-), 5.13 (s, 2H, -OCH_2_-), 3.86 (s, 3H, =N-OCH_3_), 3.86 − 3.81 (m, 2H, -SCH_2_-), 3.73 (s, 3H, -CO-OCH_3_), 3.40-3.34 (m, 2H, -SCH_2_-), 2.34 (s, 3H, -CH_3_).^13^C NMR (126 MHz, CDCl_3_) *δ* 167.76, 160.60, 160.55, 158.84, 152.61, 135.12, 131.29, 131.06, 128.36, 127.91, 127.54, 124.77, 117.67, 114.36, 112.25, 109.84, 108.92, 69.23, 62.31, 51.92, 42.70, 41.56, 18.95. HRMS (ESI): calcd for C_25_H_24_O_6_S_2_ ([M+Na]^+^), 507.09125; found, 507.09065.

Methyl(*E*)-2-(2-(((8-(bis(isopropylthio)methyl)-4-methyl-2-oxo-2H-chromen-7-yl)oxy)methyl)phenyl)-3-methoxyacrylate (D27). Yellow solid; yield 75 %; m.p. 144 − 145 ℃. ^1^H NMR (400 MHz, CDCl_3_) *δ* 7.82 (dd, *J* = 8.0, 4.0 Hz, 1H, Ar-H), 7.66 (s, 1H, -C=CH-O-), 7.36 − 7.27 (m, 3H, Ar-H), 7.17 − 7.15 (m, 1H, Ar-H), 6.66 (d, *J* = 8.0 Hz, 1H, Ar-H), 6.12 (s, 1H, Ar-H), 5.78 (s, 1H, -SCHS-), 5.29 (s, 2H, -OCH_2_-), 3.89 (s, 3H, =N-OCH_3_), 3.76 (s, 3H, -CO-OCH_3_), 3.09-2.99 (m, 2H, -SCH-), 2.34 (s, 3H, -CH_3_), 1.39 (d, *J* = 8.0 Hz, 6H, -SCHCH_3_), 1.29 (d, *J* = 8.0 Hz, 6H, -SCHCH_3_). ^13^C NMR (100 MHz, CDCl_3_) *δ* 167.76, 160.49, 159.46, 152.89, 149.26, 135.46, 130.56, 129.66, 128.19, 127.11, 126.48, 124.21, 118.60, 113.21, 111.84, 109.65, 109.53, 68.27, 62.17, 51.85, 40.98, 37.34, 23.61, 23.49, 18.87. HRMS (ESI): calcd for C_29_H_34_O_6_S_2_ ([M+Na]^+^), 565.16937; found, 565.16890.

Methyl(*E*)-2-(2-(((8-(bis(tert-butylthio)methyl)-4-methyl-2-oxo-2H-chromen-7-yl)oxy)methyl)phenyl)-3-methoxyacrylate (D28). Yellow solid; yield 81 %; m.p. 70 − 72 ℃. ^1^H NMR (500 MHz, CDCl_3_) *δ* 7.92 (d, *J* =10.0 Hz, 1H, Ar-H), 7.64 (s, 1H, -C=CH-O-), 7.32 − 7.14 (m, 3H, Ar-H), 7.14 (d, *J* = 5.0 Hz, 1H, Ar-H), 6.62 (d, *J* = 10.0 Hz, 1H, Ar-H), 6.10 (s, 1H, Ar-H), 5.87 (s, 1H, -SCHS-), 5.27 (s, 2H, -OCH_2_-), 3.87 (s, 3H, =N-OCH_3_), 3.74 (s, 3H, -CO-OCH_3_), 2.32 (s, 3H, -CH_3_), 1.34 (s, 18H, -SCCH_3_). ^13^C NMR (126 MHz, CDCl_3_) *δ* 167.78, 160.48, 159.37, 152.97, 148.36, 135.60, 130.49, 129.62, 128.16, 127.07, 126.74, 123.87, 121.12, 113.15, 111.68, 109.72, 109.55, 68.30, 62.16, 51.84, 45.65, 36.58, 31.02, 30.94, 18.90. HRMS (ESI): calcd for C_31_H_38_O_6_S ([M+Na]^+^), 593.20050; found, 593.20020.

Methyl(*E*)-2-(2-(((8-(bis((4-chlorophenyl)thio)methyl)-2-oxo-2*H*-chromen-7-yl)oxy)methyl)phenyl)-2-(methoxyimino)acetate (D29): White solid; yield 87%; m.p. 48 − 49 ℃. ^1^H NMR (400 MHz, CDCl_3_) *δ* 7.99 (dd, *J* = 7.8, 1.2 Hz, 1H, Ar-H), 7.53 − 7.12 (m, 13H, Ar-H), 6.77 (d, *J* = 8.8 Hz, 1H, Ar-H), 6.17 (s, 1H, -SCHS-), 6.14 (d, *J* =10 Hz, 1H, Ar-H), 5.25 (s, 2H, -OCH_2_-), 4.13 (s, 3H, =N-OCH_3_), 3.93 (s, 3H, -CO-OCH_3_). ^13^C NMR (101 MHz, CDCl_3_) *δ* 163.37, 159.62, 159.24, 156.10, 150.25, 148.94, 143.40, 134.46, 134.38, 133.69, 129.98, 129.03, 128.92, 128.49, 128.02, 127.77, 127.12, 116.42, 113.22, 112.45, 109.64, 68.62, 64.09, 53.31, 53.01. HRMS (ESI): calcd for C_33_H_25_O_6_NCl_2_S_2_ ([M+Na]^+^), 688.03986; found, 688.03926.

Methyl(*E*)-2-(2-(((8-(bis((2,4-dichlorophenyl)thio)methyl)-2-oxo-2*H*-chromen-7-yl)oxy)methyl)phenyl)-2-(methoxyimino)acetate (D30). White solid; yield 89 %; m.p. 64 − 65℃. ^1^H NMR (400 MHz, CDCl_3_) *δ* 8.08 (dd, *J* = 7.9, 1.2 Hz, 1H, Ar-H), 7.59 − 7.36 (m, 5H, Ar-H), 7.34 (dd, *J* = 11.7, 2.2 Hz, 2H, Ar-H), 7.27 (d, *J* = 7.8 Hz, 1H, Ar-H), 7.23 (dd, *J* = 7.6, 1.3 Hz, 1H, Ar-H), 7.11 (dd, *J* = 8.4, 2.2 Hz, 2H, Ar-H), 6.78 (d, *J* = 8.8 Hz, 1H, Ar-H), 6.36 (s, 1H, -SCHS-), 6.17 (d, *J* = 9.2 Hz, 1H, Ar-H), 5.27 (s, 2H, -OCH_2_-), 4.13 (s, 3H, =N-OCH_3_), 3.93 (s, 3H, -CO-OCH_3_). ^13^C NMR (101 MHz, CDCl_3_) *δ* 163.38, 159.55, 159.34, 143.29, 138.11, 136.59, 135.48, 134.80, 134.35, 132.40, 130.01, 129.64, 129.48, 128.76, 127.98, 127.78, 127.40, 127.37, 127.29, 115.46, 113.27, 112.43, 109.56, 68.73, 64.09, 53.32, 50.27. HRMS (ESI): calcd for C_33_H_23_O_6_NCl_4_S_2_ ([M+H]^+^), 733.98157; found, 733.97937.

Methyl(*E*)-2-(2-(((8-(bis((4-fluorophenyl)thio)methyl)-2-oxo-2H-chromen-7-yl)oxy)methyl)phenyl)-2-(methoxyimino)acetate (D31): White solid; yield 85 %; m.p. 88 − 89 ℃. ^1^H NMR (400 MHz, CDCl_3_) *δ* 8.03 (dd, *J* = 7.9, 1.2 Hz, 1H, Ar-H), 7.52 − 7.44 (m, 2H, Ar-H), 7.42 − 7.26 (m, 6H, Ar-H), 7.23 − 7.10 (m, 1H, Ar-H), 6.98 − 6.81 (m, 4H, Ar-H), 6.77 (d, *J* = 8.7 Hz, 1H, Ar-H), 6.12 (d, *J* = 9.8 Hz, 1H, Ar-H), 6.06 (s, 1H, -SCHS-), 5.26 (s, 2H, -OCH_2_-), 4.13 (s, 3H, =N-OCH_3_), 3.93 (s, 3H, -CO-OCH_3_). ^13^C NMR (101 MHz, CDCl_3_) *δ* 164.06, 163.38, 161.59, 159.69, 159.20, 150.30, 148.96, 143.39, 135.84, 135.76, 134.54, 129.98, 128.34, 128.00, 127.75, 127.16, 116.05, 115.83, 113.15, 112.39, 109.62, 68.60, 64.07, 53.98, 53.30. ^19^F NMR (376 MHz, CDCl_3_) *δ* -112.62. HRMS (ESI): calcd for C_33_H_25_O_6_NF_2_S_2_ ([M+Na]^+^), 656.09851; found, 656.09836.

Methyl(*E*)-2-(2-(((8-(bis(p-tolylthio)methyl)-2-oxo-2H-chromen-7-yl)oxy)methyl)phenyl)-2-(methoxyimino)acetate (D32). White solid; yield 79 %; m.p. 52 − 53 ℃. ^1^H NMR (400 MHz, CDCl_3_) *δ* 8.08 (dd, *J* = 7.6, 1.6 Hz, 1H, Ar-H), 7.47 (d, *J* = 9.4 Hz, 1H, Ar-H), 7.42 − 7.37 (m, 1H, Ar-H), 7.32-7.28 (m, 3H, Ar-H), 7.27 − 7.25 (m, 2H, Ar-H), 7.21 (dd, *J* = 9.0, 1.7 Hz, 2H, Ar-H), 7.02 − 6.98 (m, 4H, Ar-H), 6.75 (d, *J* = 8.8 Hz, 1H, Ar-H), 6.11 (s, 1H, -SCHS-), 6.18 (d, *J* = 9.6 Hz, 1H, Ar-H), 5.27 (s, 2H, -OCH_2_-), 4.13 (s, 3H, =N-OCH_3_), 3.93 (s, 3H, -CO-OCH_3_), 2.28 (s, 6H, -CH_3_). ^13^C NMR (101 MHz, CDCl_3_) *δ* 163.39, 159.81, 159.26, 150.35, 143.34, 138.11, 134.70, 133.41, 133.32, 131.91, 130.00, 129.55, 129.46, 128.00, 127.85, 127.60, 127.22, 117.39, 112.94, 112.33, 109.60, 68.47, 64.06, 53.29, 53.23, 21.12. HRMS (ESI): calcd for C_35_H_31_O_6_NS_2_ ([M+Na]^+^), 648.14722; found, 648.14850.

Methyl(*E*)-2-(2-(((8-(bis(benzylthio)methyl)-2-oxo-2H-chromen-7-yl)oxy)methyl)phenyl)-2-(methoxyimino)acetate (D33). White solid; yield 85 %; m.p. 52 − 53 ℃. ^1^H NMR (400 MHz, CDCl_3_) *δ* 7.90 (d, *J* = 7.7 Hz, 1H, Ar-H), 7.53 − 7.30 (m, 3H, Ar-H), 7.26 − 7.12 (m, 9H, Ar-H), 7.13 − 6.92 (m, 3H, Ar-H), 6.65 (d, *J* = 8.8 Hz, 1H, Ar-H), 6.20 (d, *J* = 9.6 Hz, 1H, Ar-H), 5.44 (s, 1H, -SCHS-), 5.15 (s, 2H, -OCH_2_-), 4.09 (s, 3H, =N-OCH_3_), 3.91 (s, 3H, -CO-OCH_3_), 3.80 − 3.63 (m, 4H, -SCH_2_-). ^13^C NMR (101 MHz, CDCl_3_) *δ* 163.32, 159.81, 159.50, 150.02, 148.94, 143.40, 136.15, 134.57, 132.76, 130.23, 129.89, 128.46, 128.35, 127.93, 127.87, 127.58, 126.95, 117.38, 113.29, 112.45, 109.55, 68.36, 64.03, 53.27, 42.62, 37.56. HRMS (ESI): calcd for C_35_H_31_O_6_NS_2_ ([M+Na]^+^), 648.14850; found, 648.14850.

Methyl(*E*)-2-(2-(((8-(bis((4-methoxyphenyl)thio)methyl)-2-oxo-2H-chromen-7-yl)oxy)methyl)phenyl)-2-(methoxyimino)acetate (D34). White solid; yield 80 %; m.p. 53 − 54 ℃. ^1^H NMR (400 MHz, CDCl_3_) *δ* 8.08 (dd, *J* = 7.9, 1.2 Hz, 1H, Ar-H), 7.53 − 7.40 (m, 2H, Ar-H), 7.40 − 7.26 (m, 5H, Ar-H), 7.21 (dd, *J* = 8.2, 2.1 Hz, 2H, Ar-H), 6.80 − 6.72 (m, 4H, Ar-H), 6.71 (d, *J* = 2.2 Hz, 1H, Ar-H), 6.08 (d, *J* = 9.2 Hz, 1H, Ar-H), 5.95 (s, 1H, -SCHS-), 5.27 (s, 2H, -OCH_2_-), 4.13 (s, 3H, =N-OCH_3_), 3.93 (s, 3H, -CO-OCH_3_), 3.77 (s, 6H, -CH_3_). ^13^C NMR (101 MHz, CDCl_3_) *δ* 163.40, 159.92, 159.86, 159.20, 150.45, 149.02, 143.35, 135.89, 135.79, 134.74, 129.99, 127.96, 127.87, 127.62, 127.22, 126.46, 126.18, 117.39, 114.32, 114.21, 112.97, 112.34, 109.59, 68.46, 64.06, 55.33, 54.72, 53.30. HRMS (ESI): calcd for C_35_H_31_O_8_NS_2_ ([M+Na]^+^), 680.13849; found, 680.13833.

^1^H NMR, ^13^C NMR, ^19^F NMR, and HRMS spectra


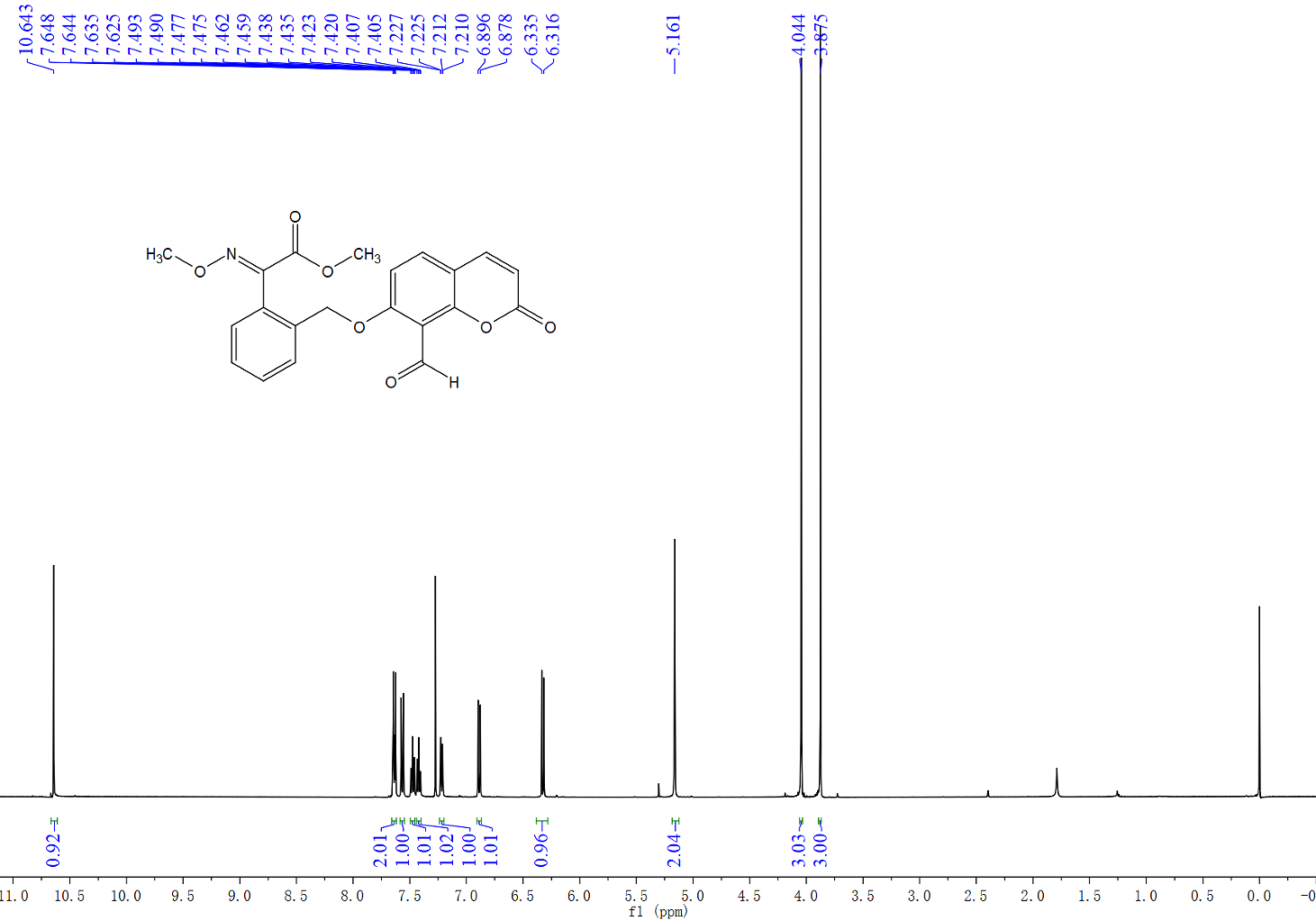


^1^H NMR of compound **4a**


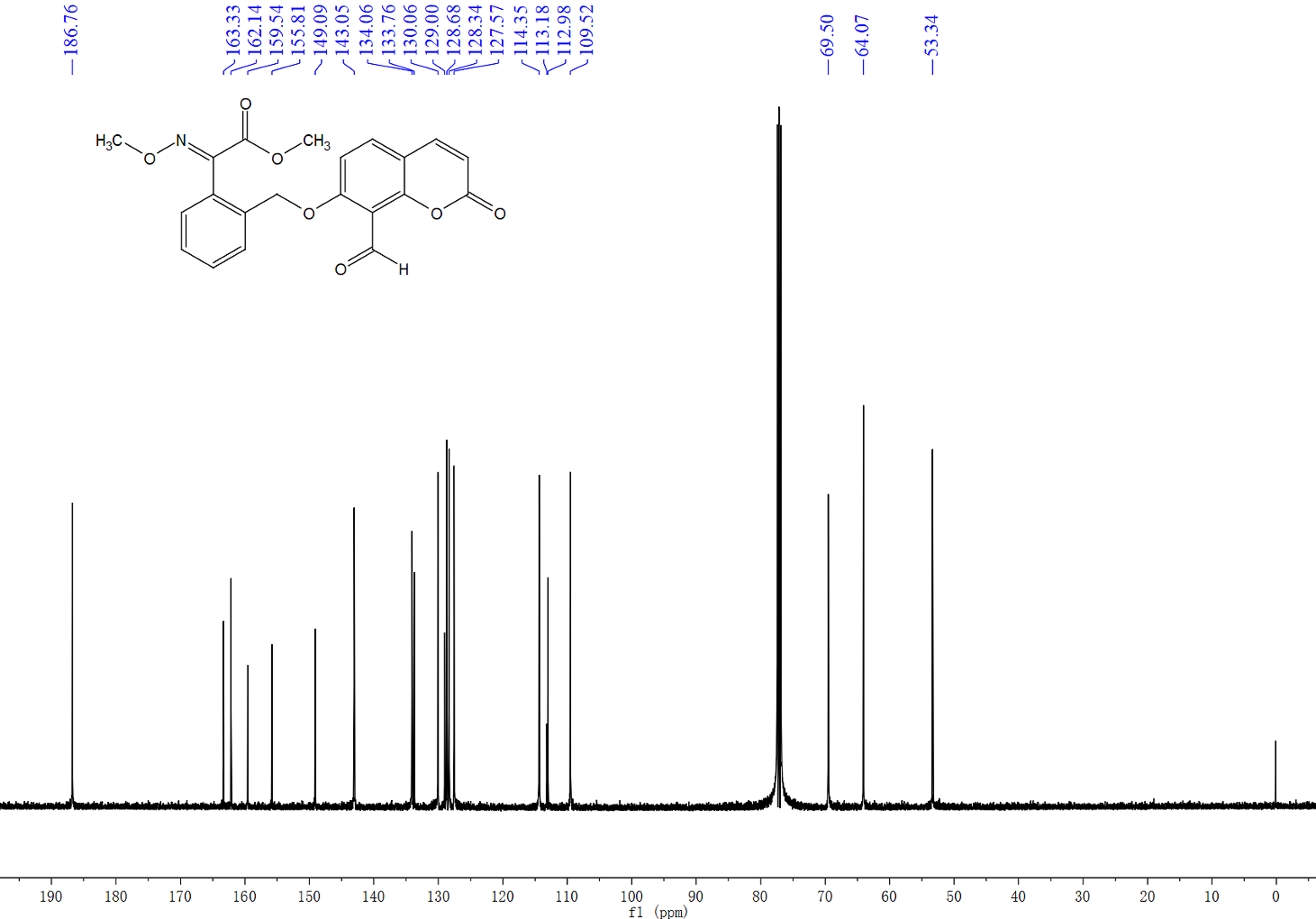


^13^C NMR of compound **4a**


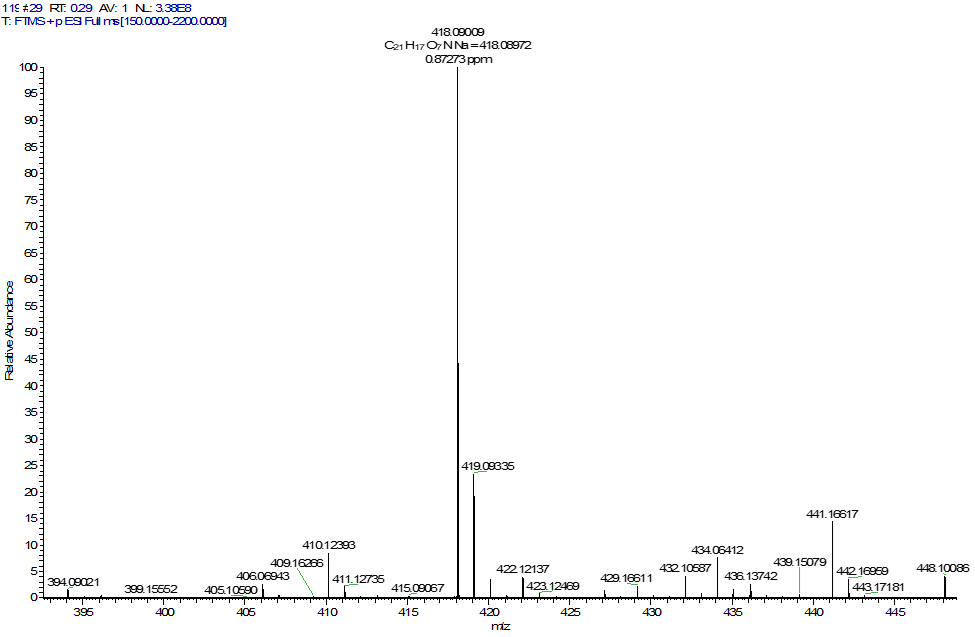


HRMS of compound **4a**


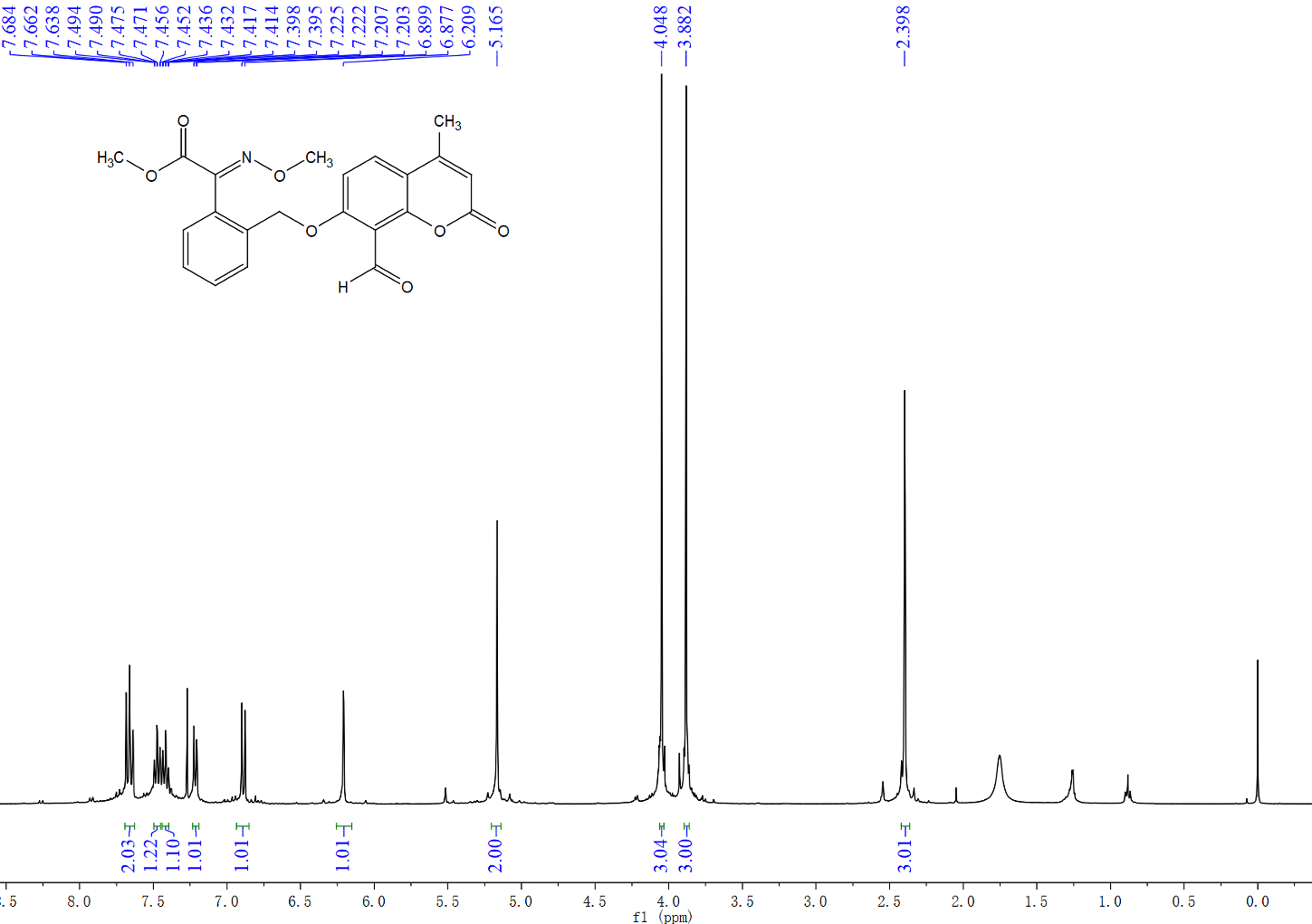


^1^H NMR of compound **4b**


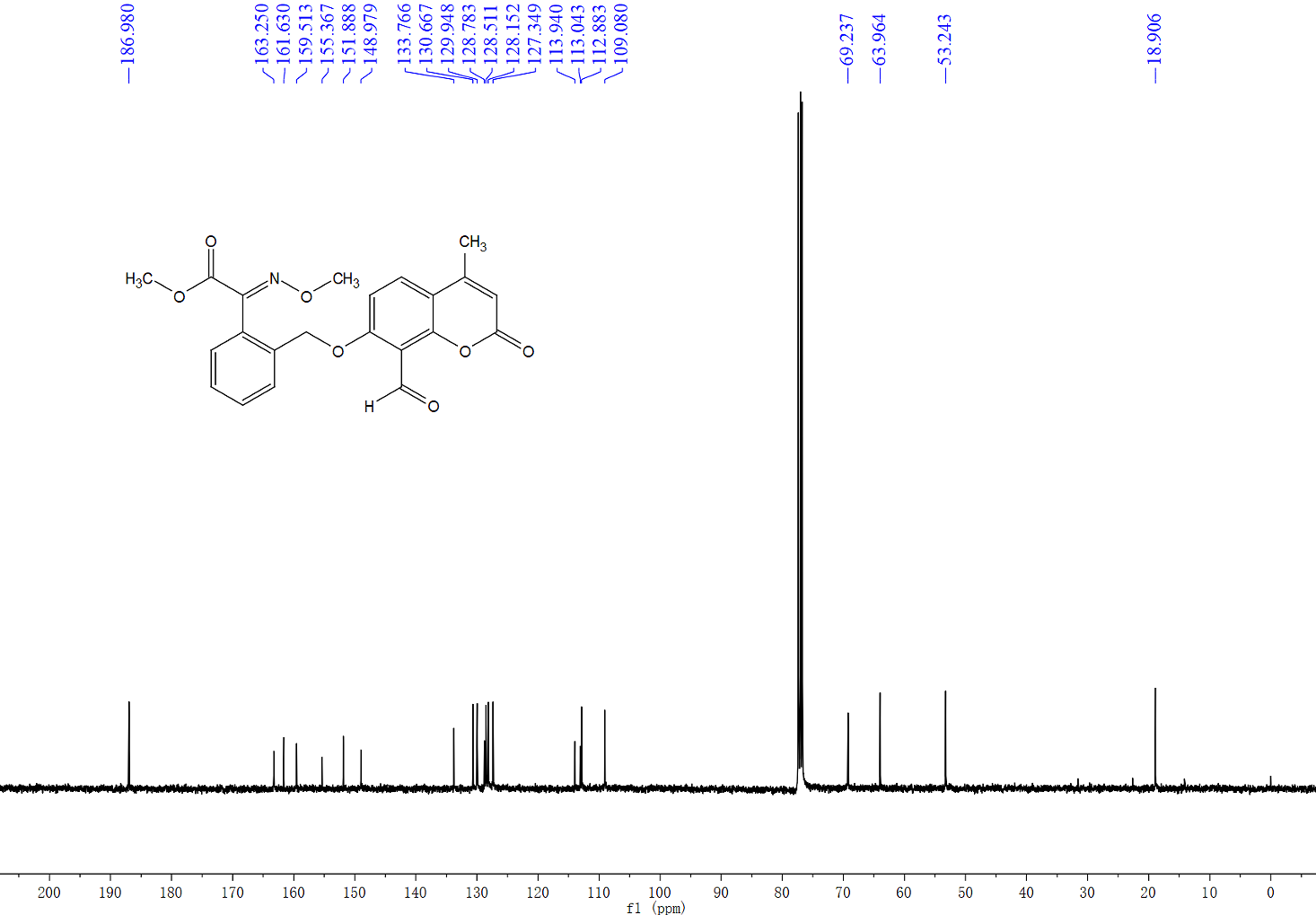


^13^C NMR of compound **4b**


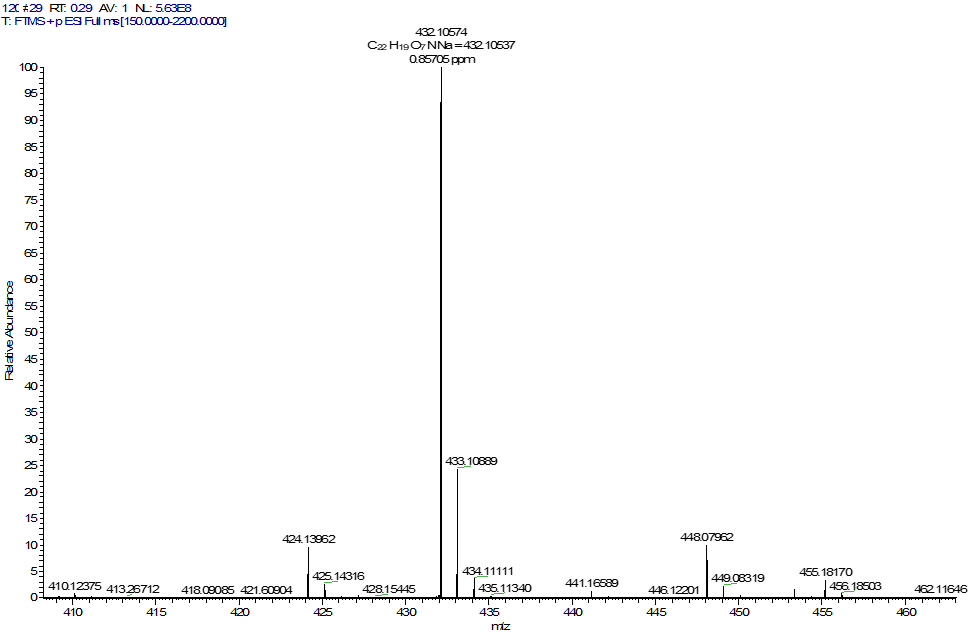


HRMS of compound **4b**


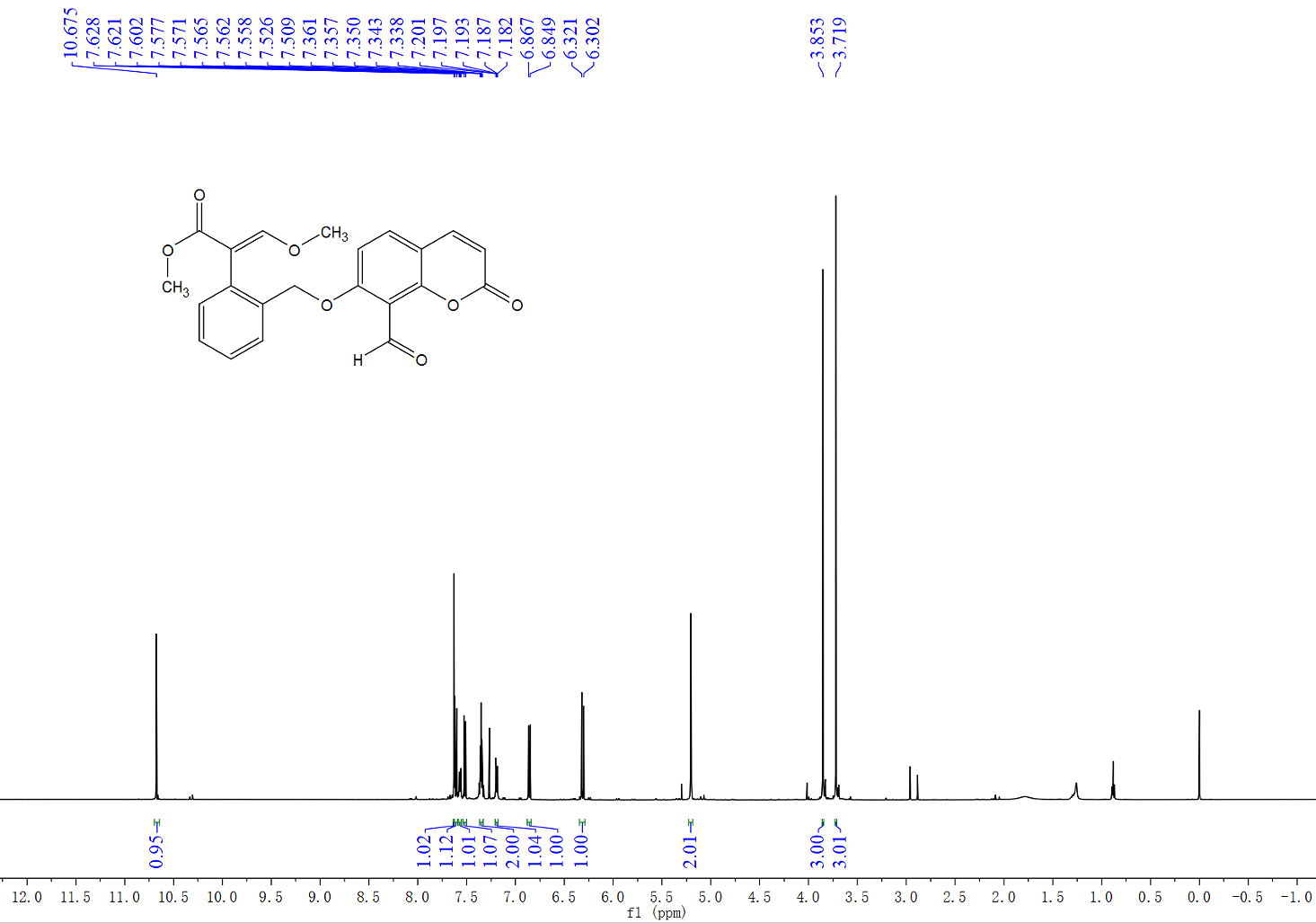


^1^H NMR of compound **4c**


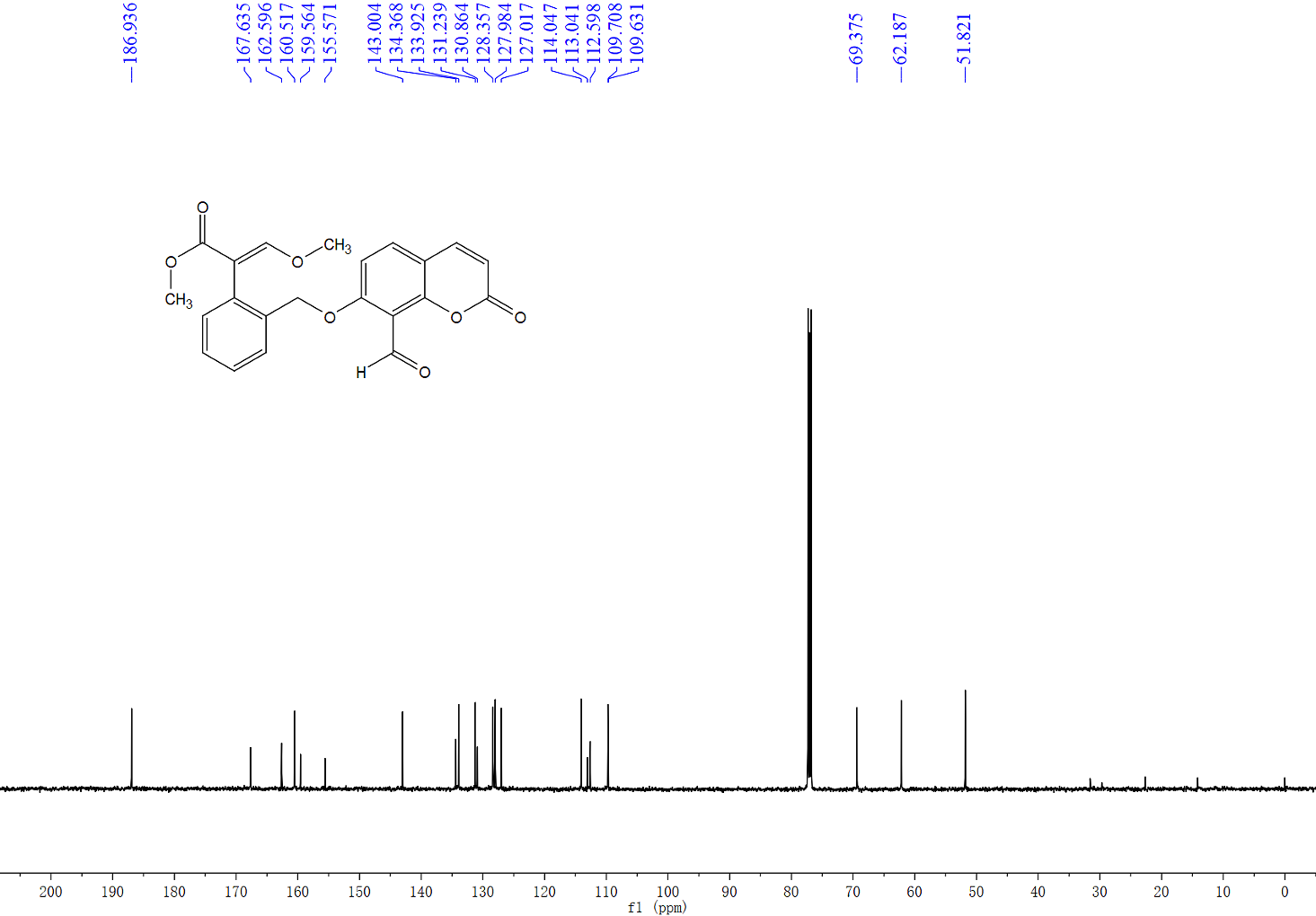


^13^C NMR of compound **4c**


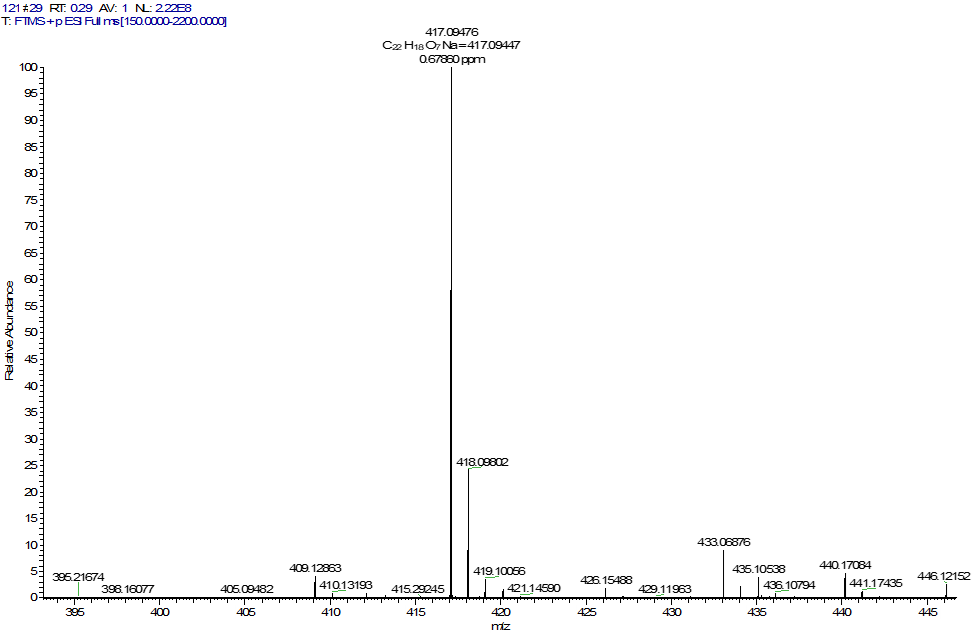


HRMS of compound **4c**


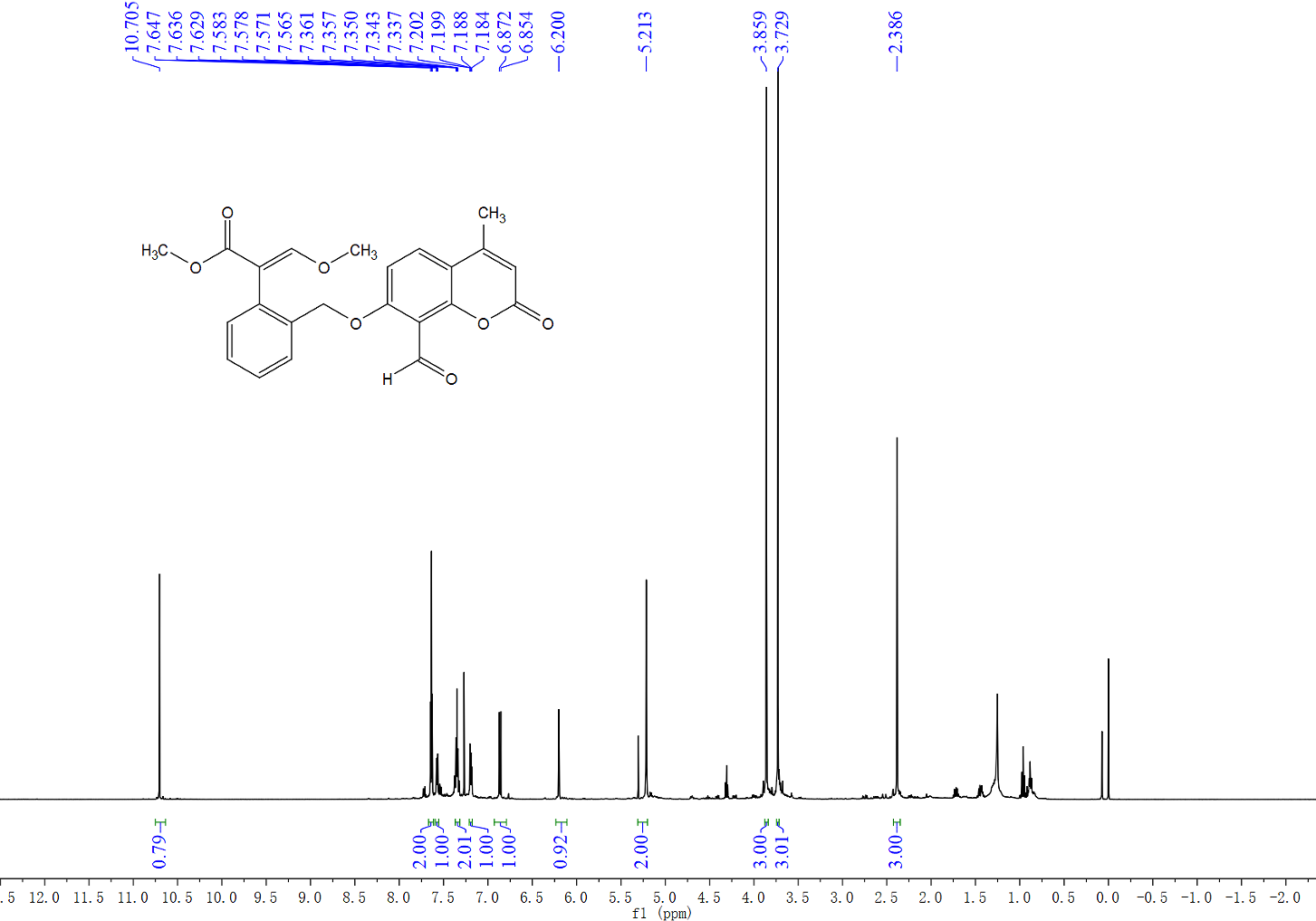


^1^H NMR of compound **4d**


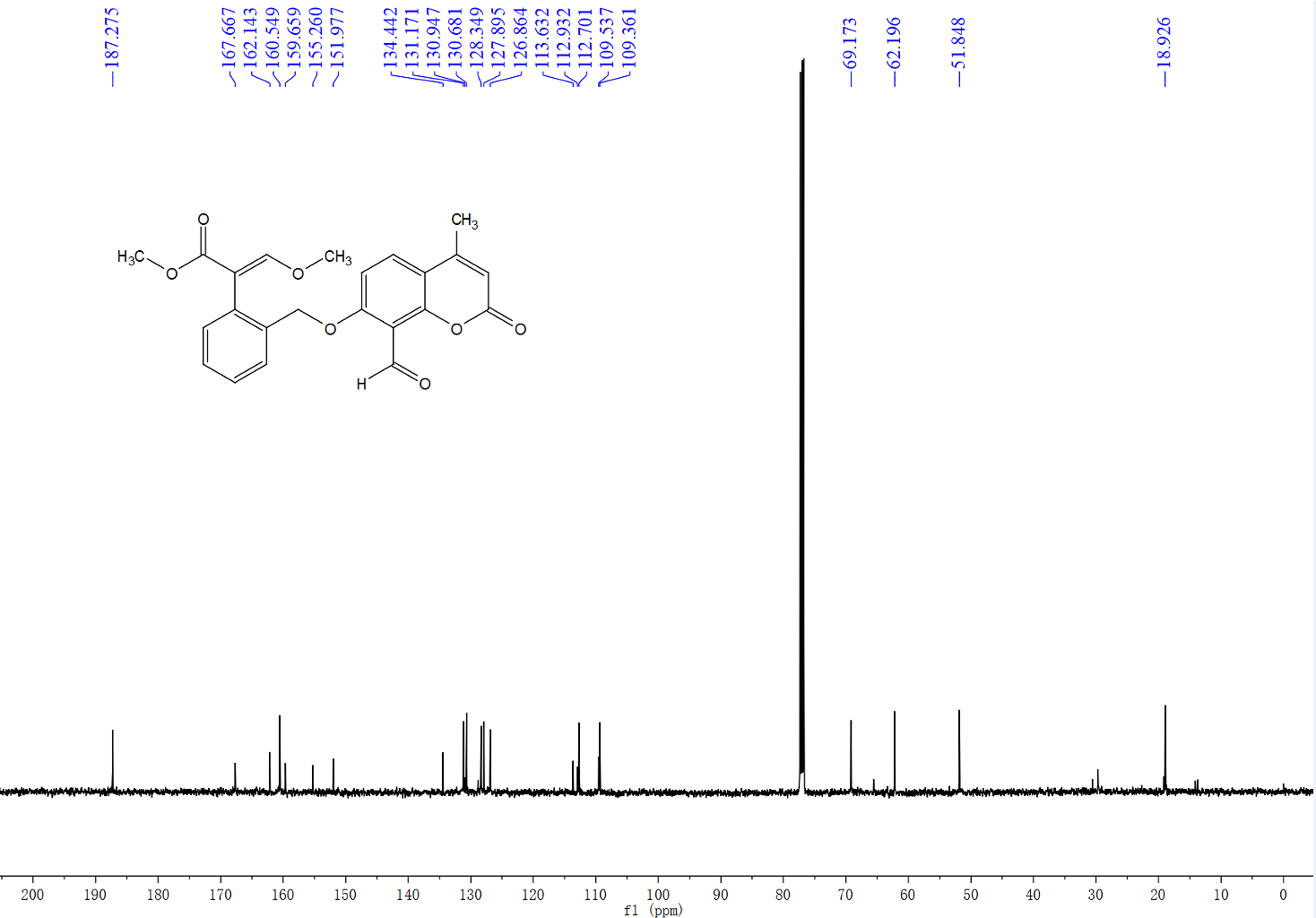


^13^C NMR of compound **4d**


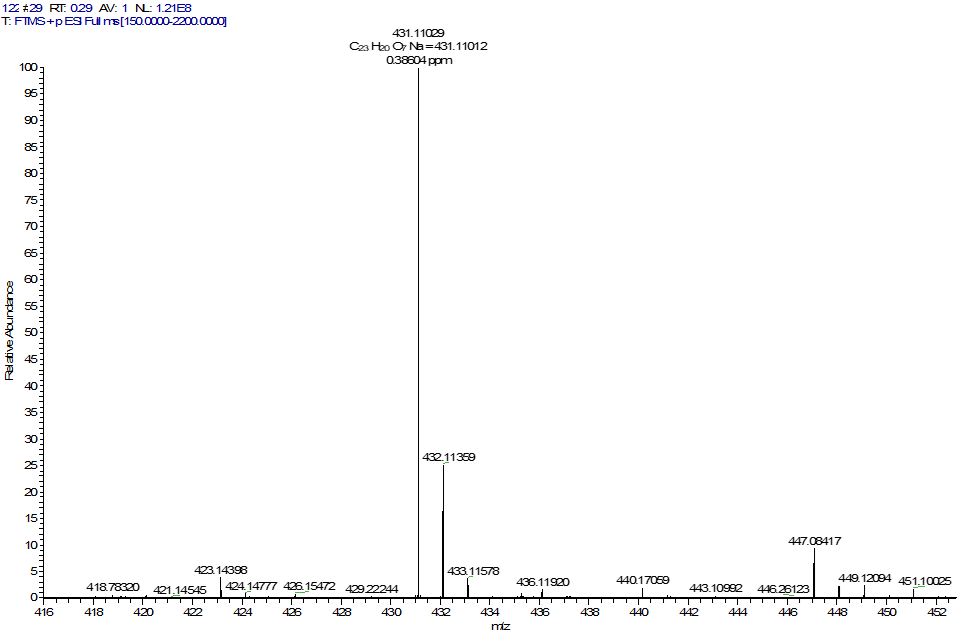


HRMS of compound **4d**


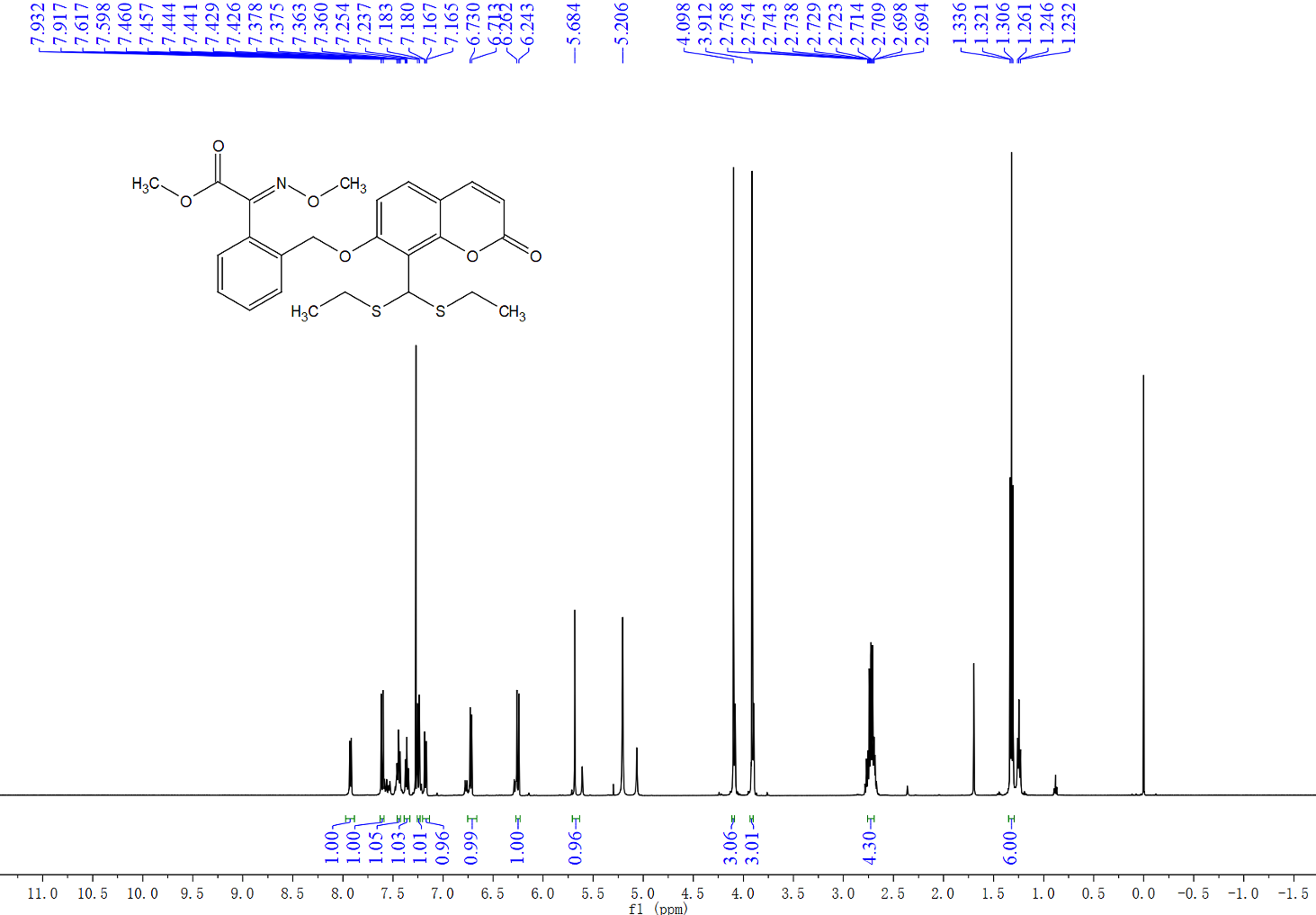


^1^H NMR of compound **D1**


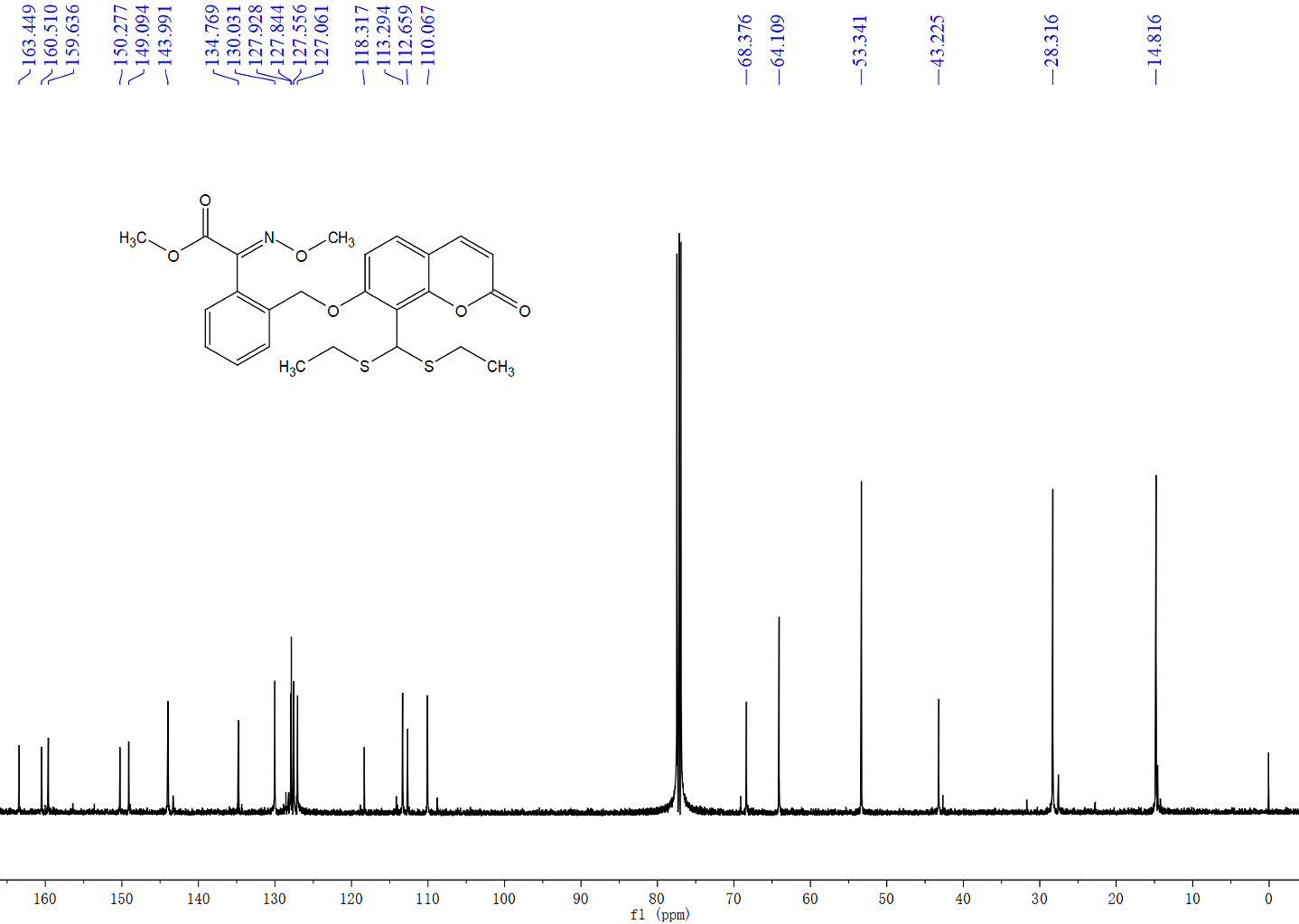


^13^C NMR of compound **D1**


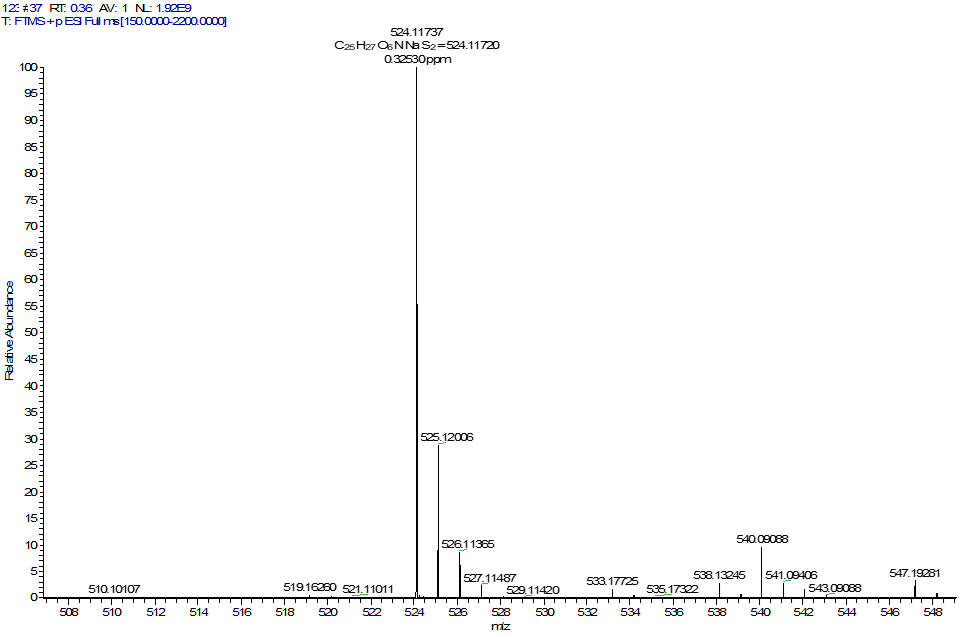


HRMS of compound **D1**


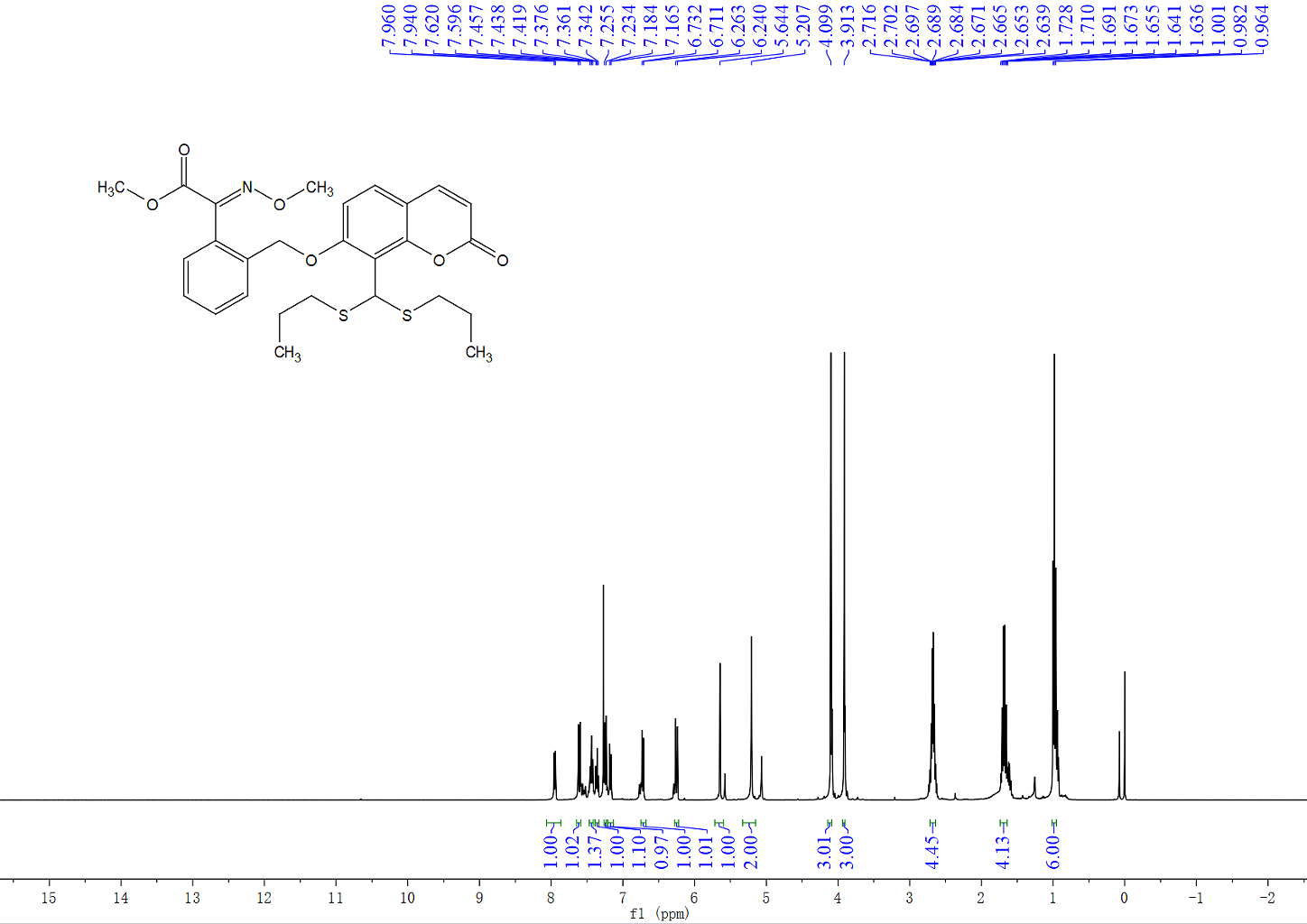


^1^H NMR of compound **D2**


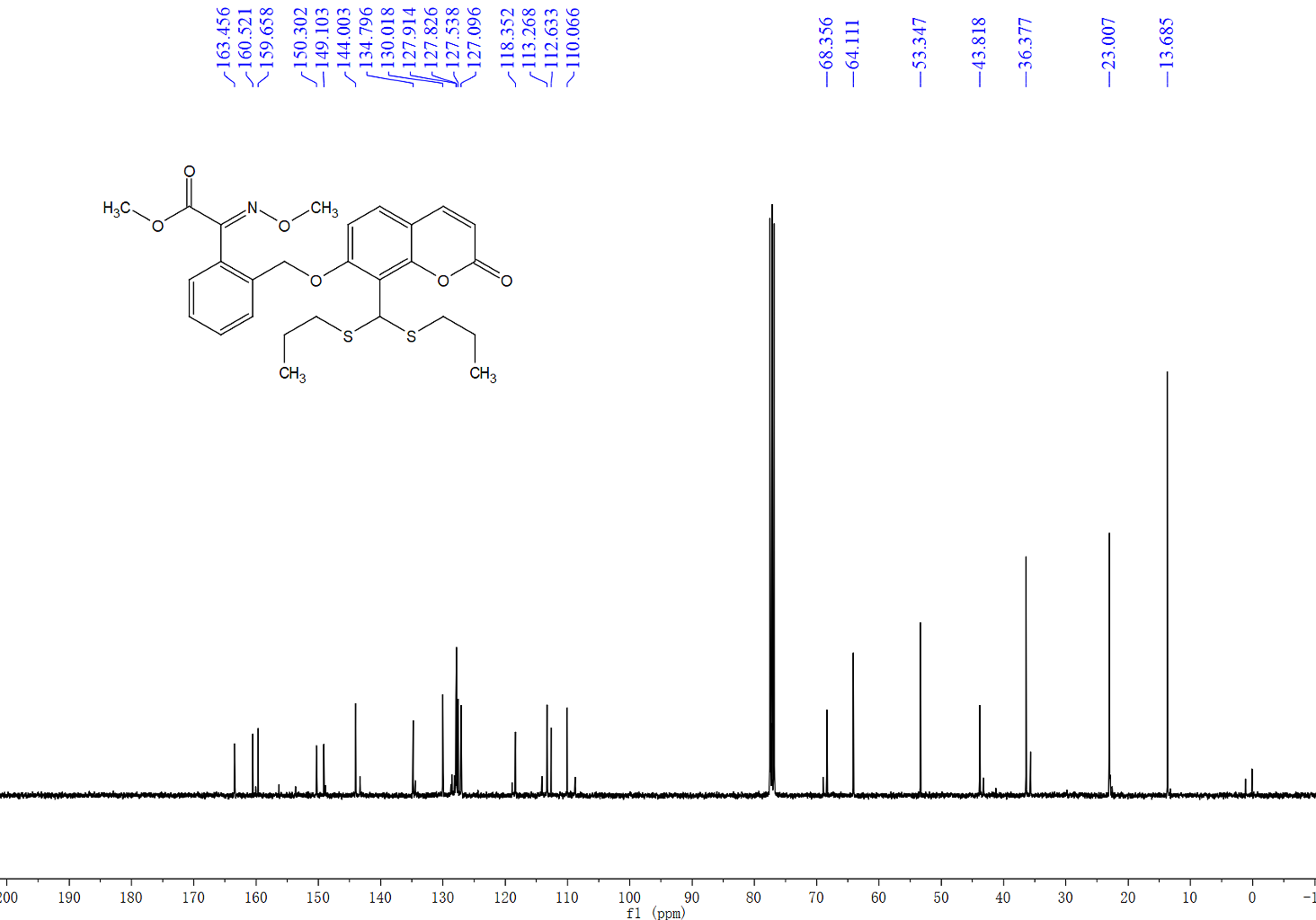


^13^C NMR of compound **D2**


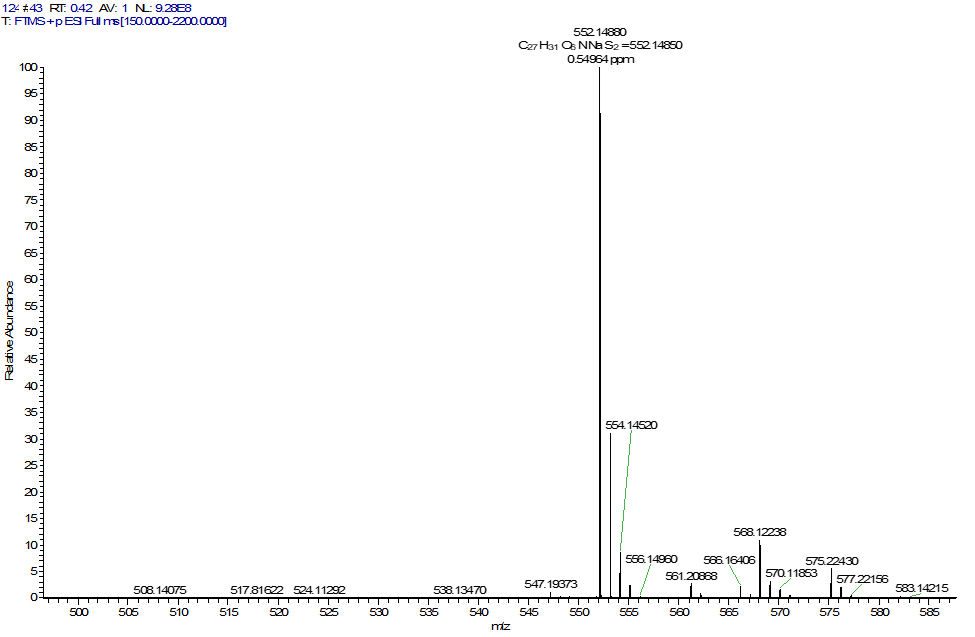


HRMS of compound **D2**


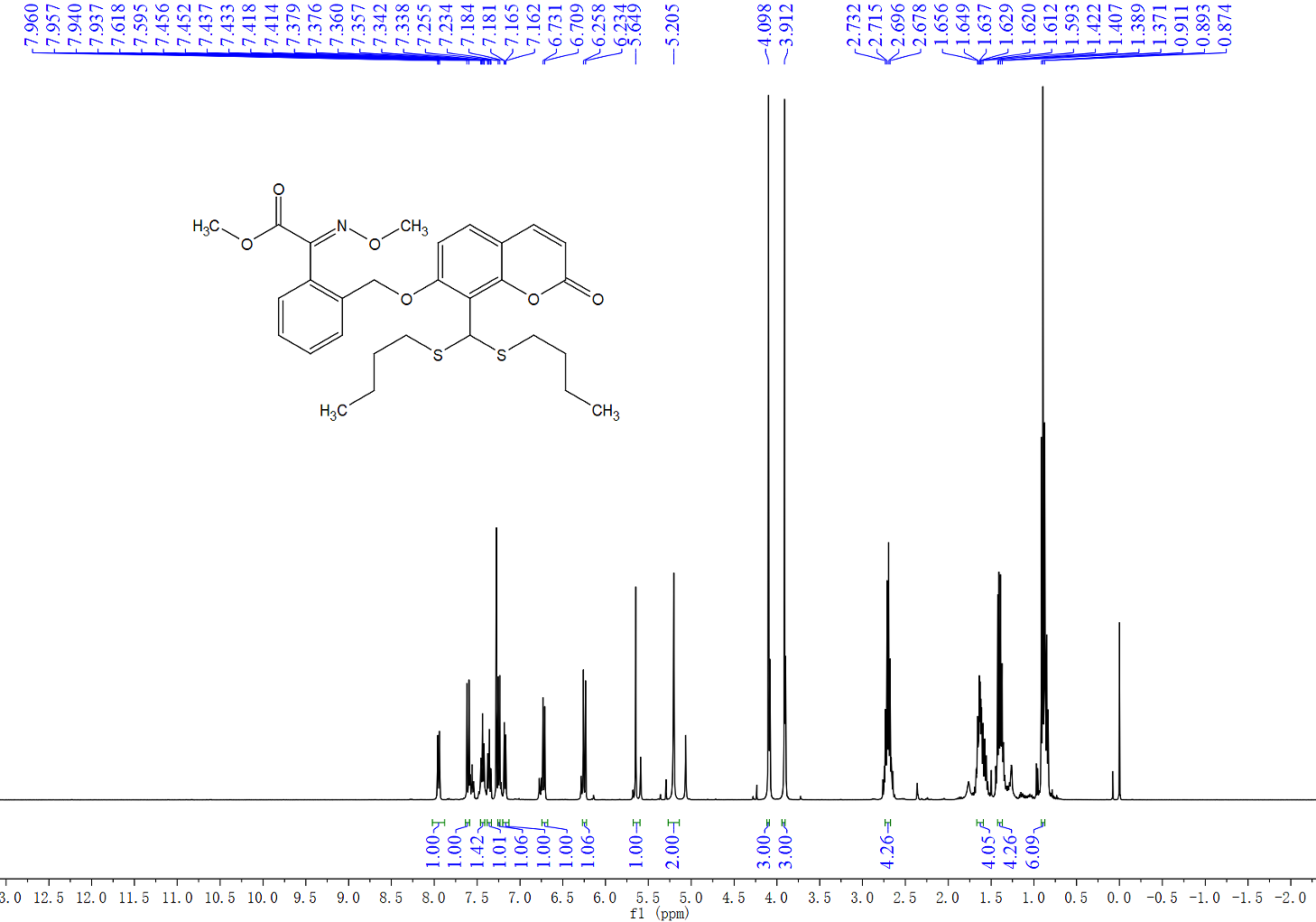


^1^H NMR of compound **D3**


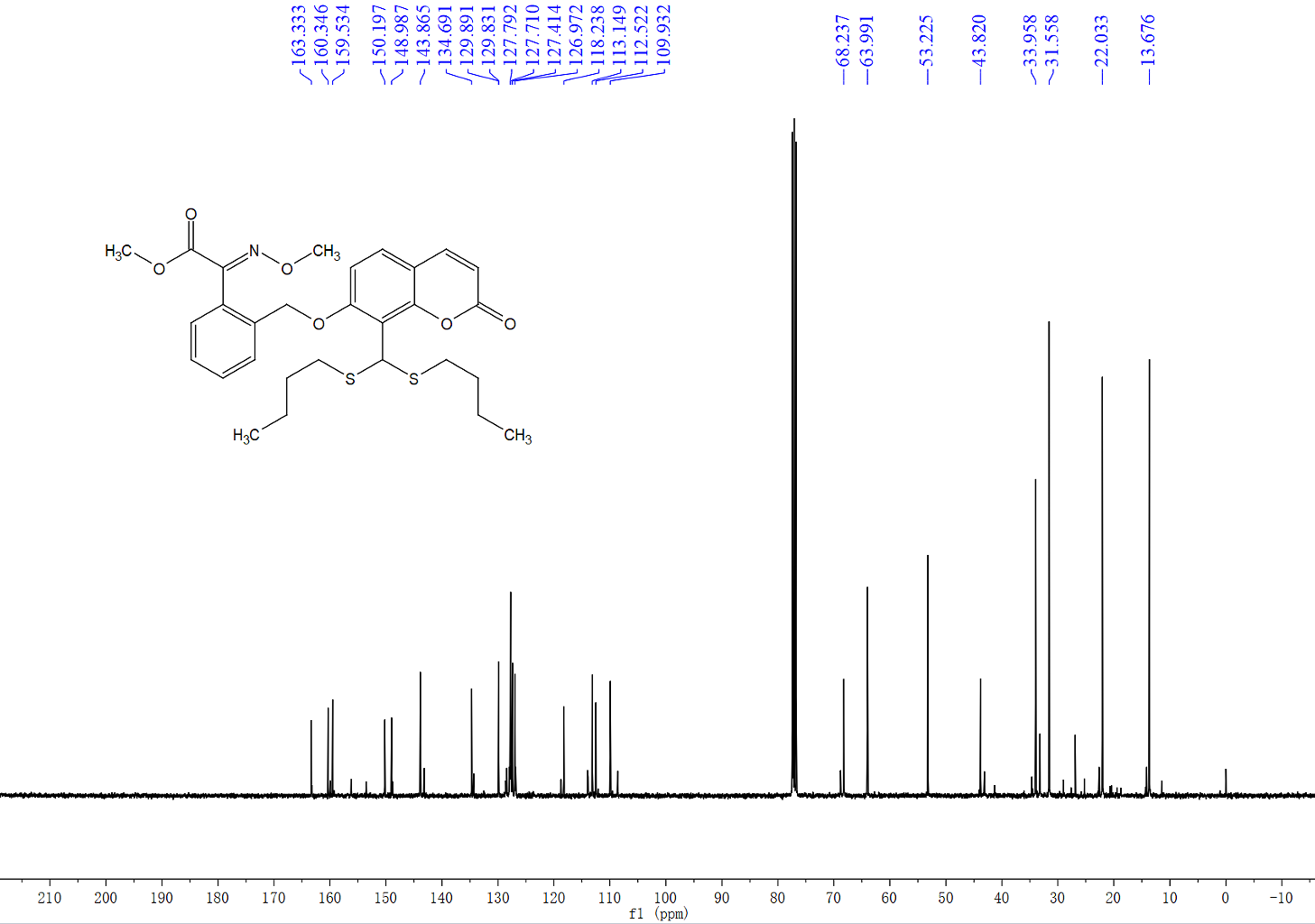


^13^C NMR of compound **D3**


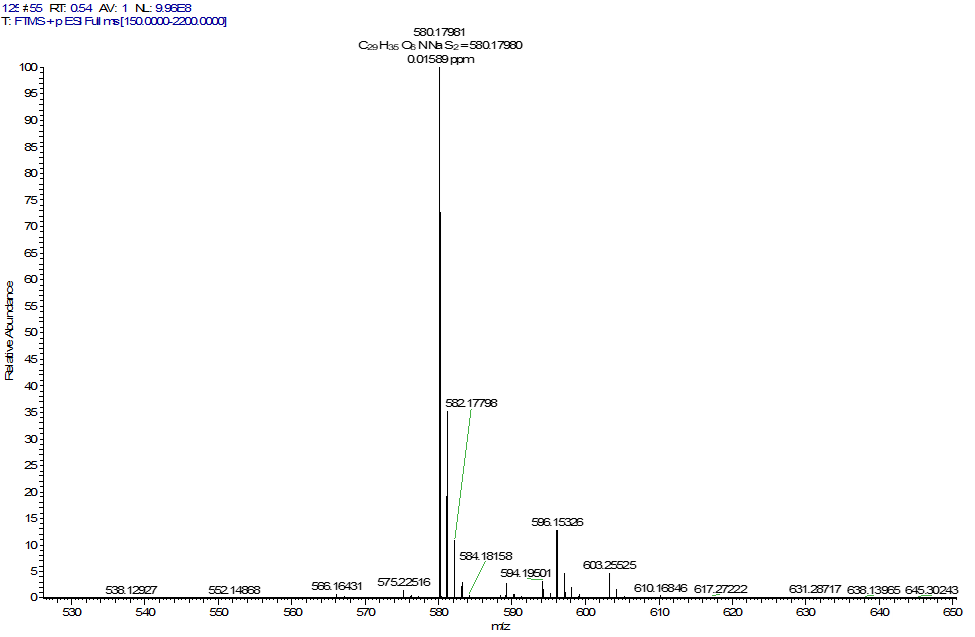


HRMS of compound **D3**


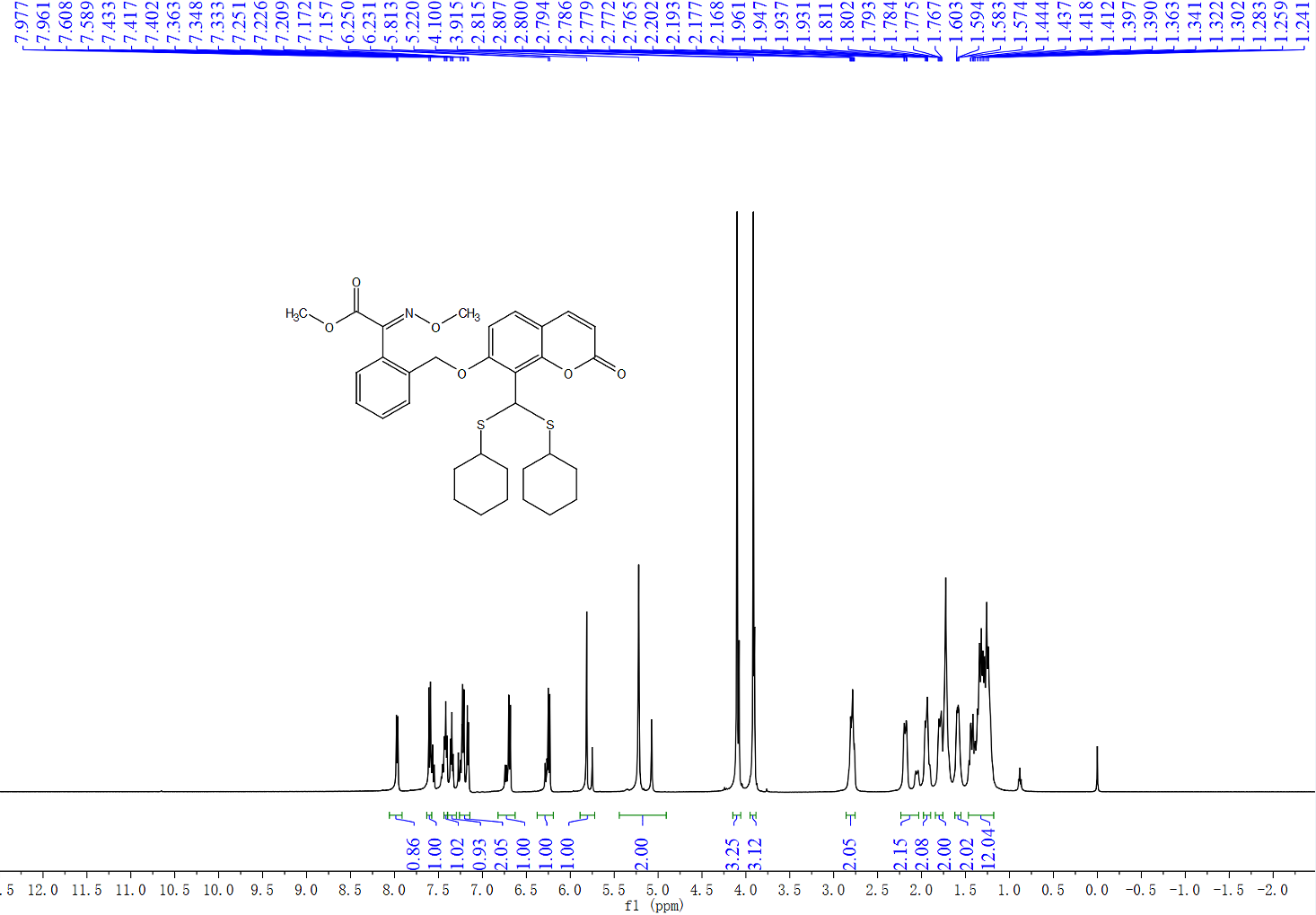


^1^H NMR of compound **D4**


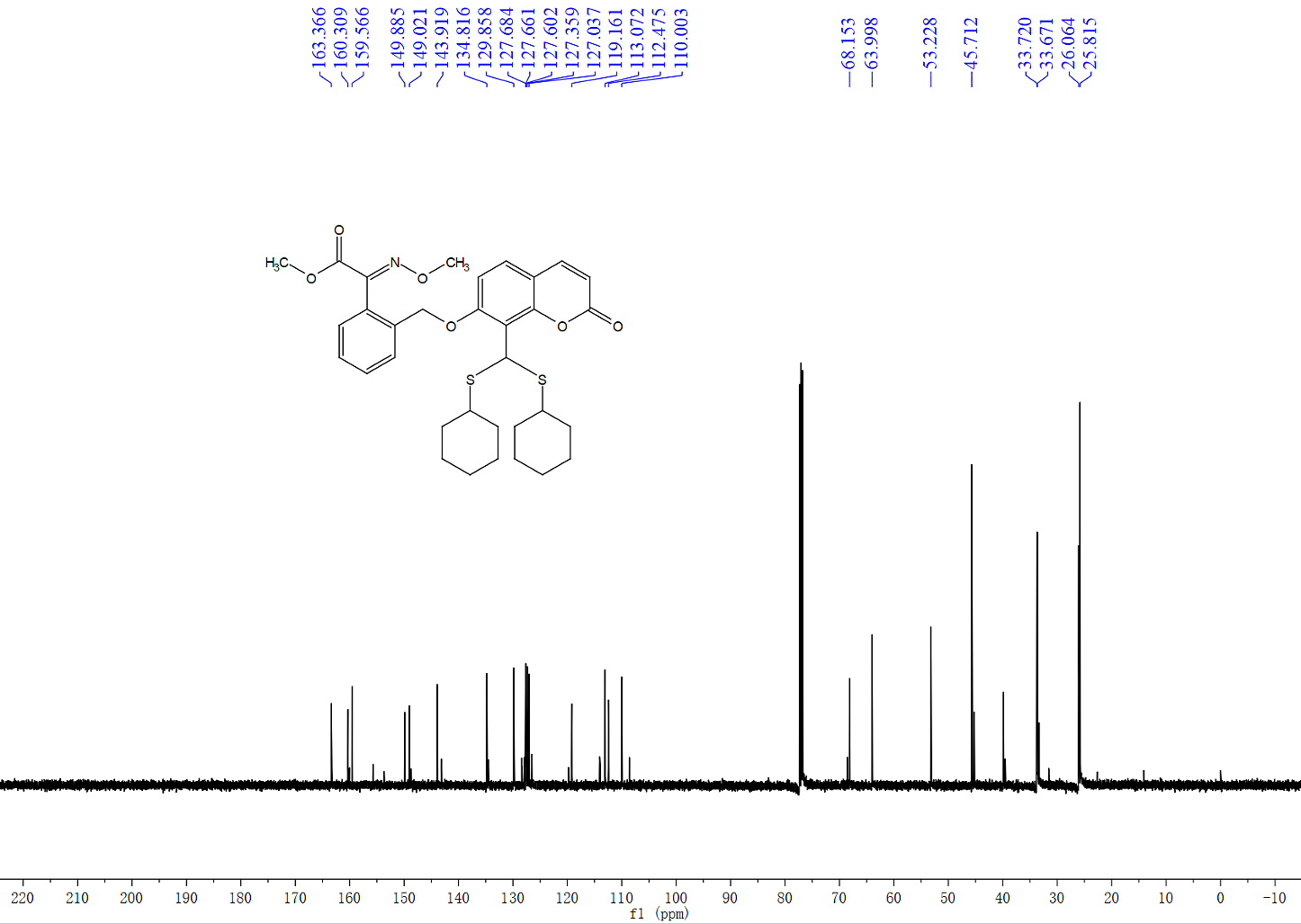


^13^C NMR of compound **D4**


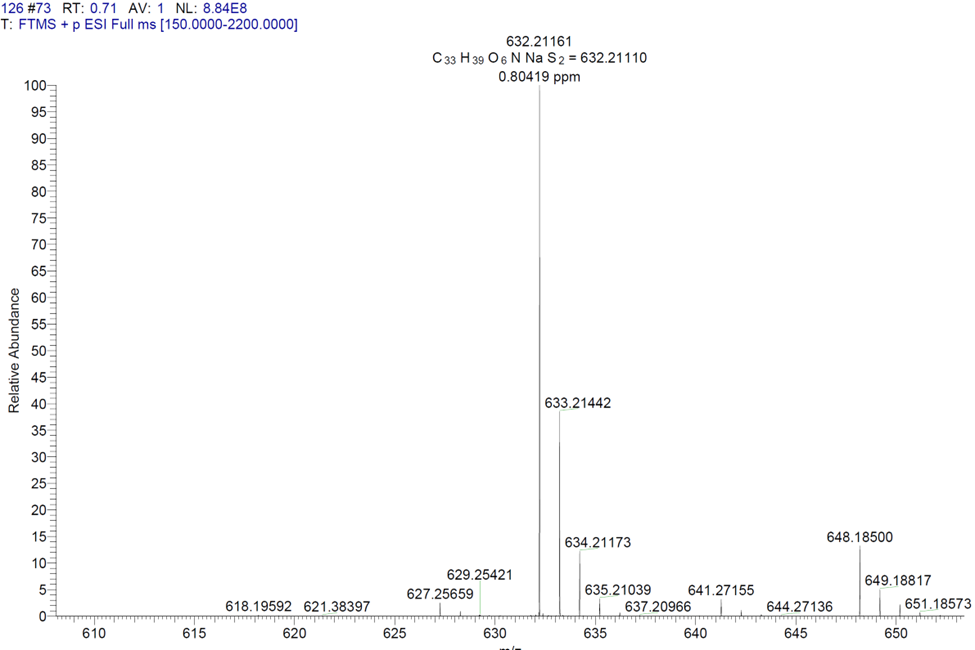


HRMS of compound **D4**


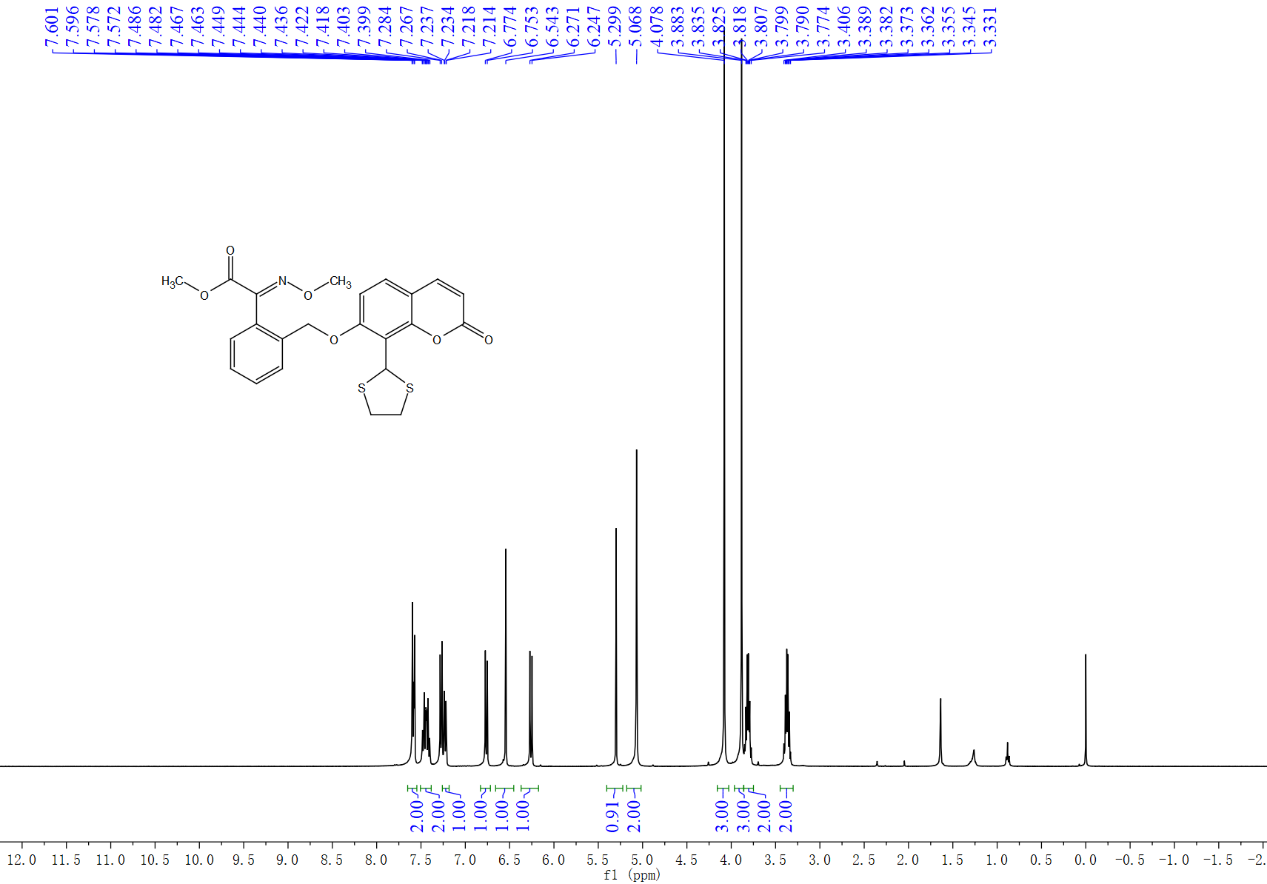


^1^H NMR of compound **D5**


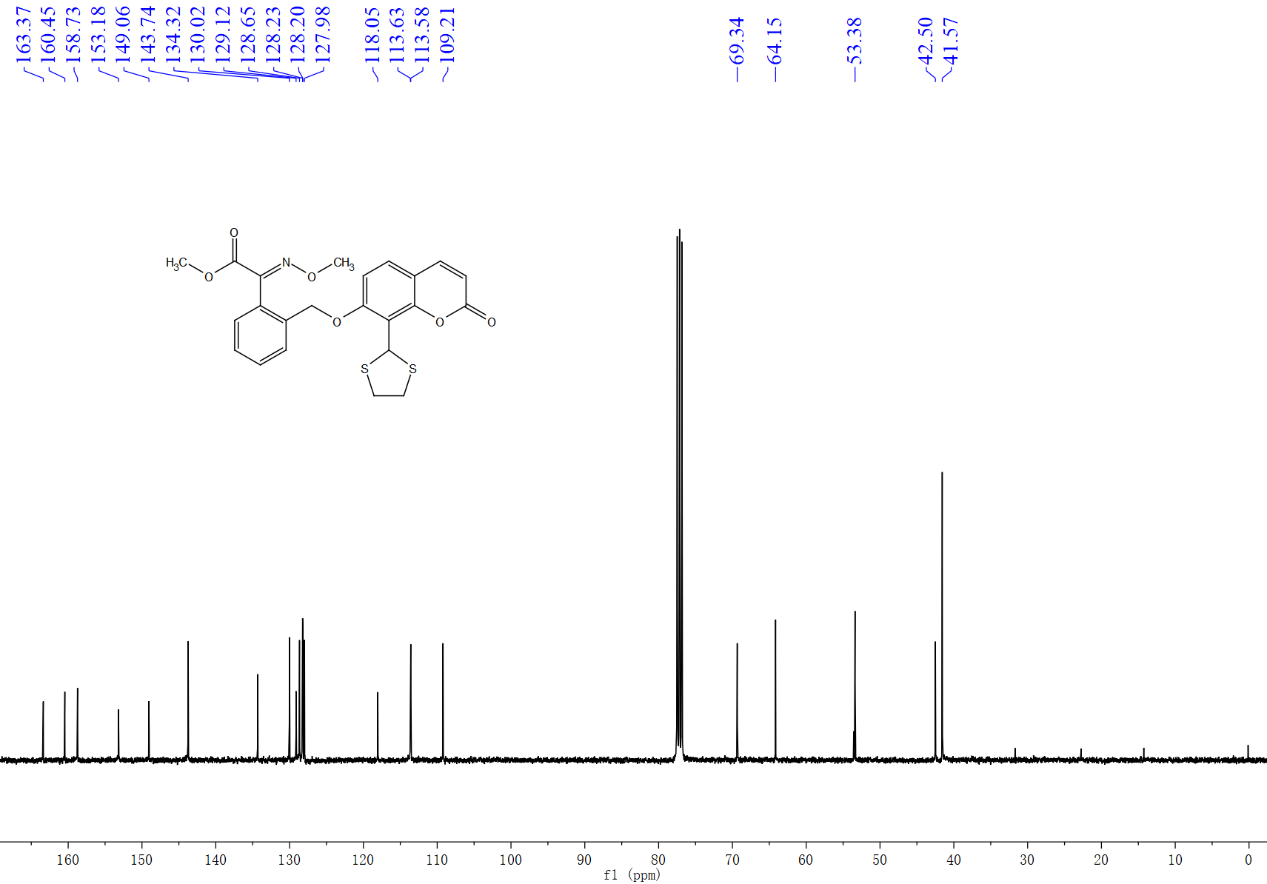


^13^C NMR of compound **D5**


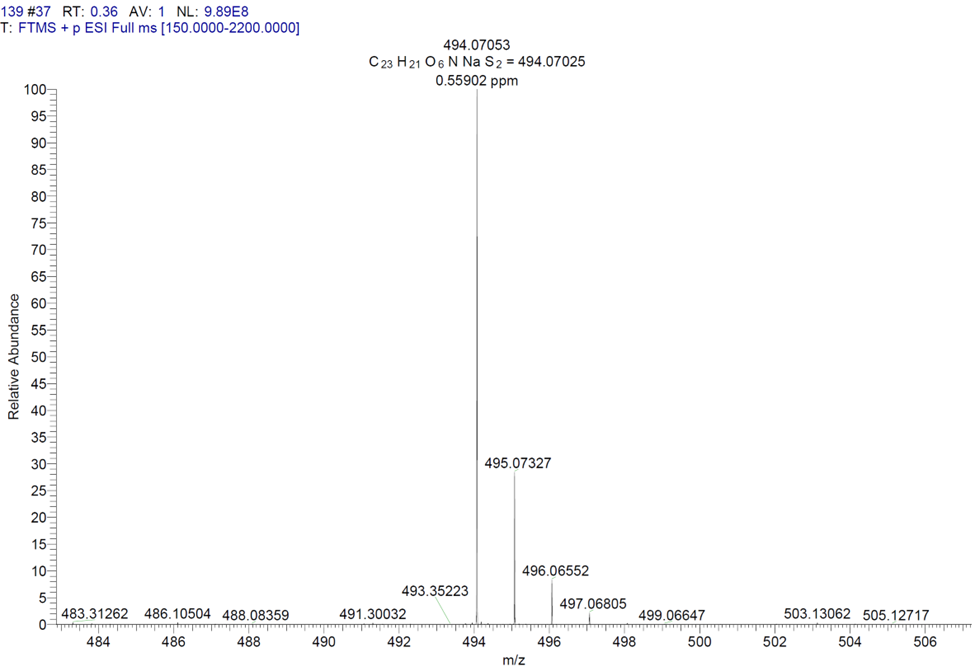


HRMS of compound **D5**


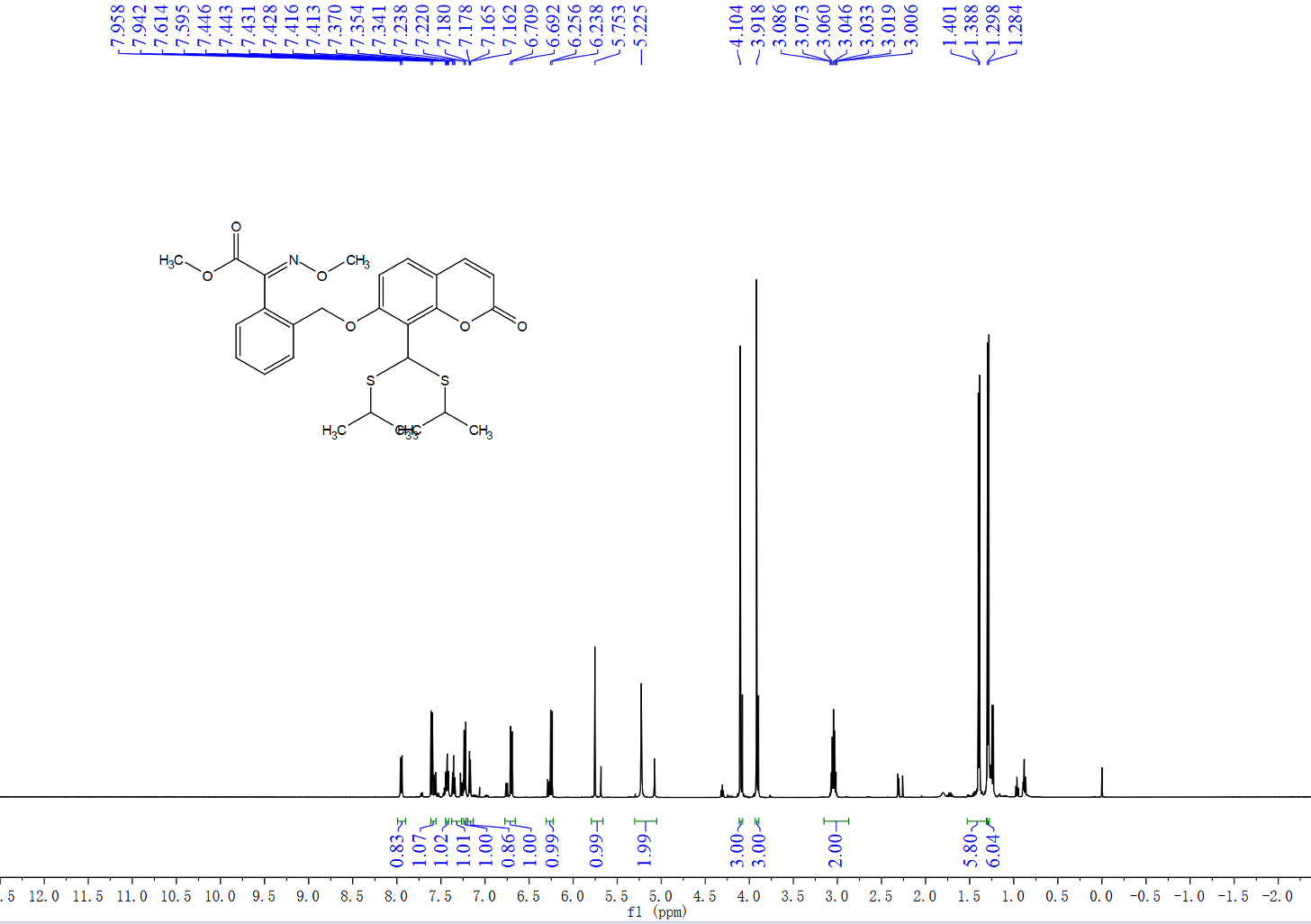


^1^H NMR of compound **D6**


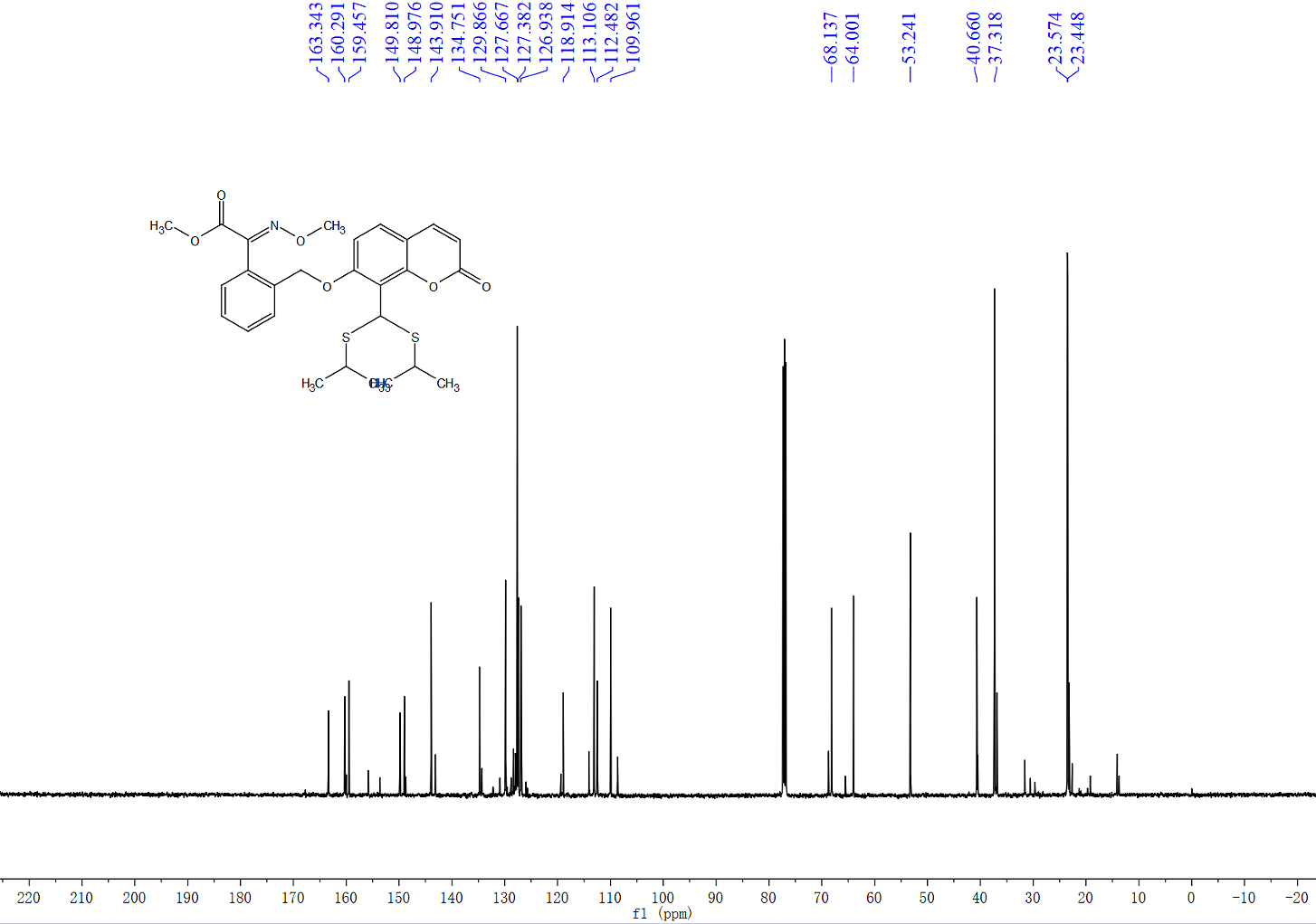


^13^C NMR of compound **D6**


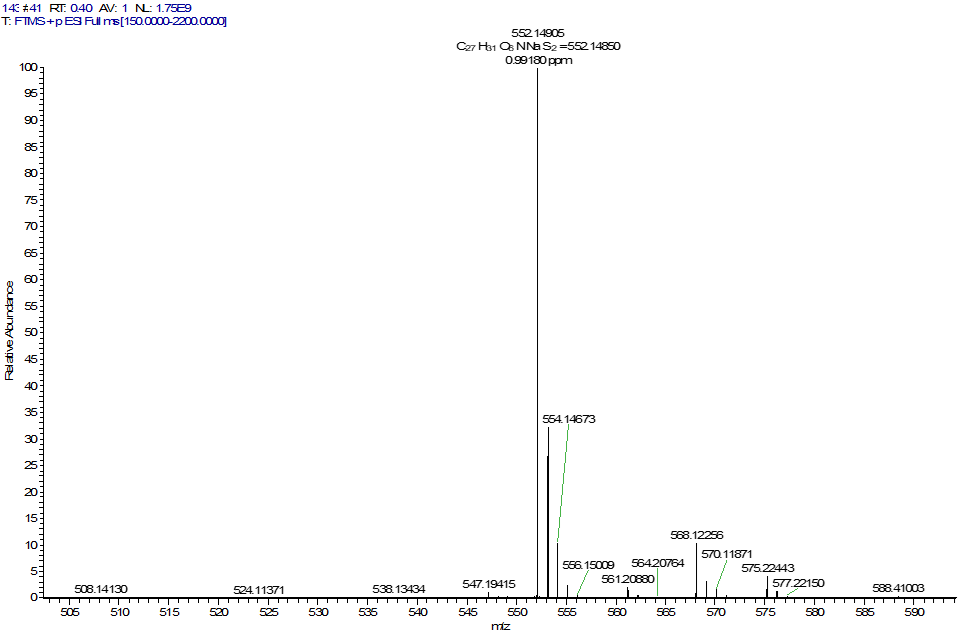


HRMS of compound **D6**


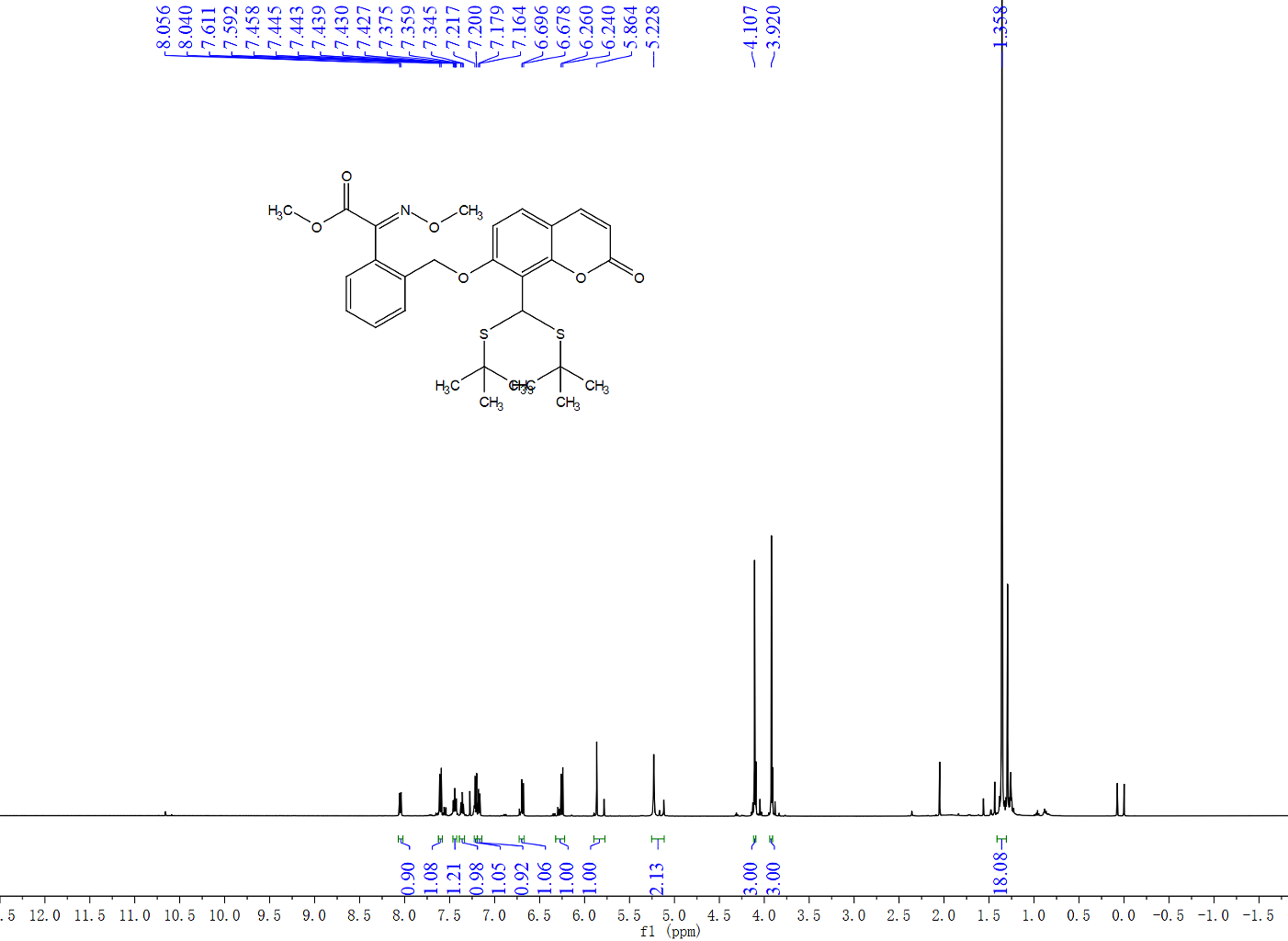


^1^H NMR of compound **D7**


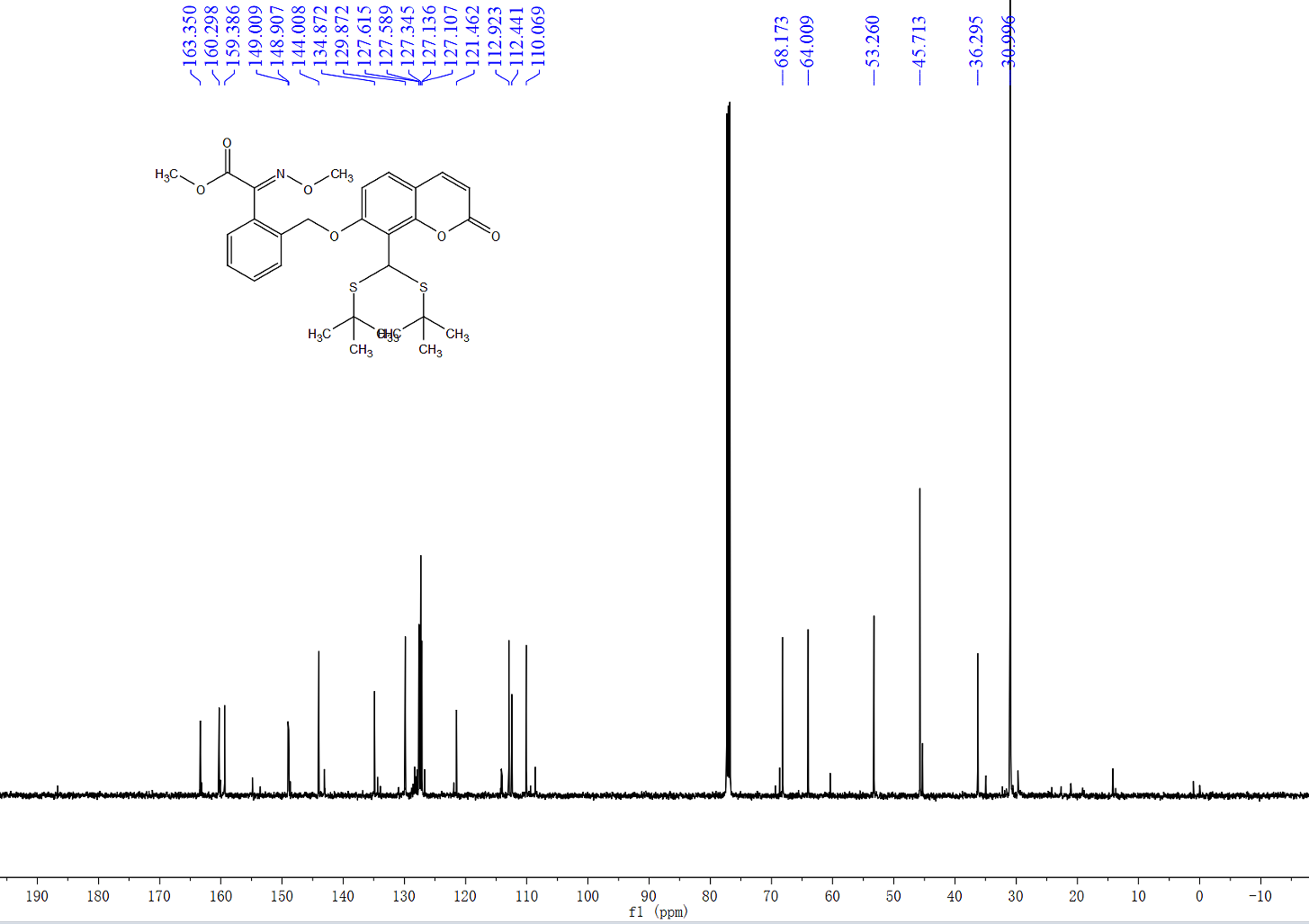


^13^C NMR of compound **D7**


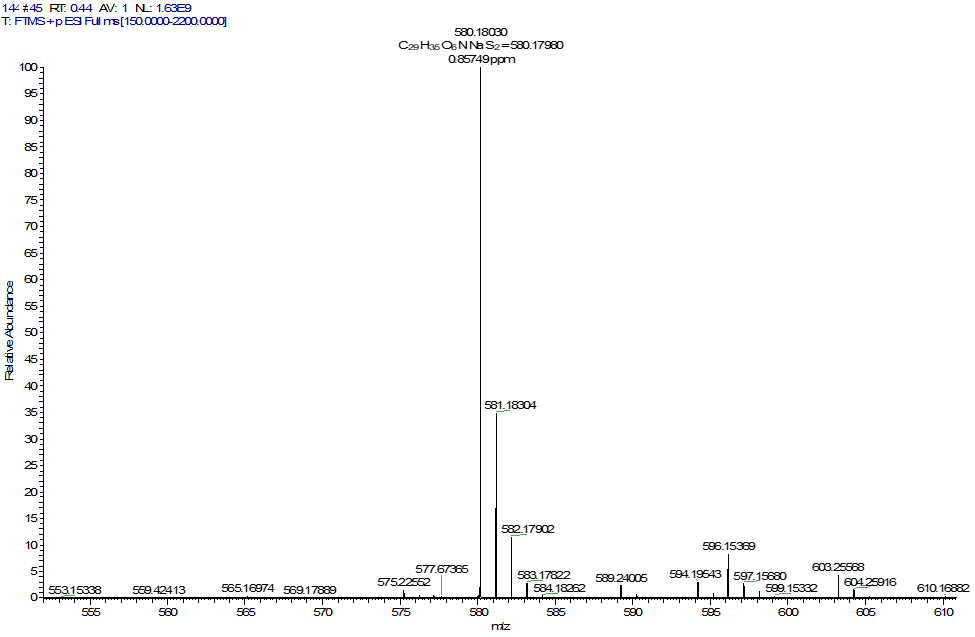


HRMS of compound **D7**


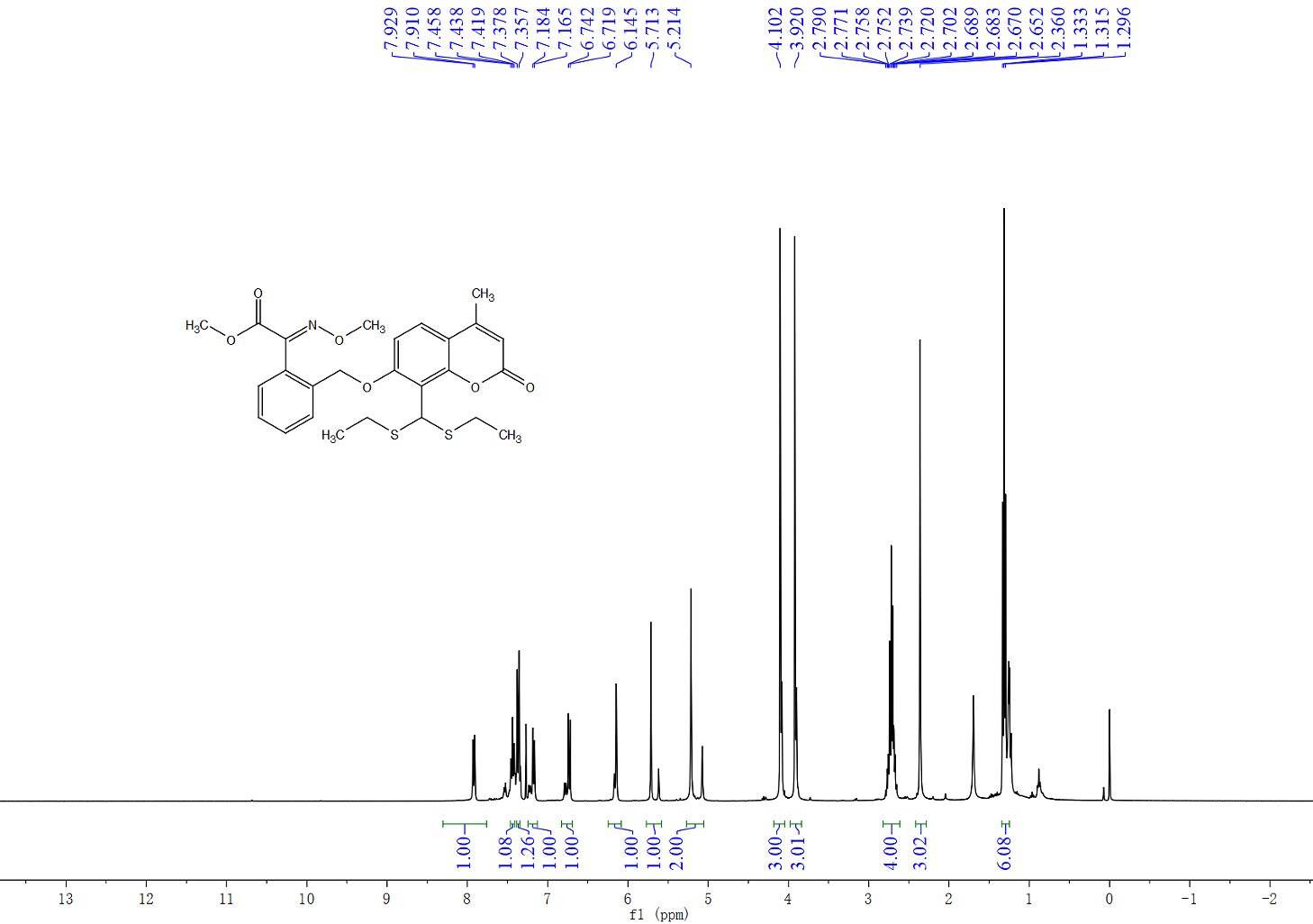


^1^H NMR of compound **D8**


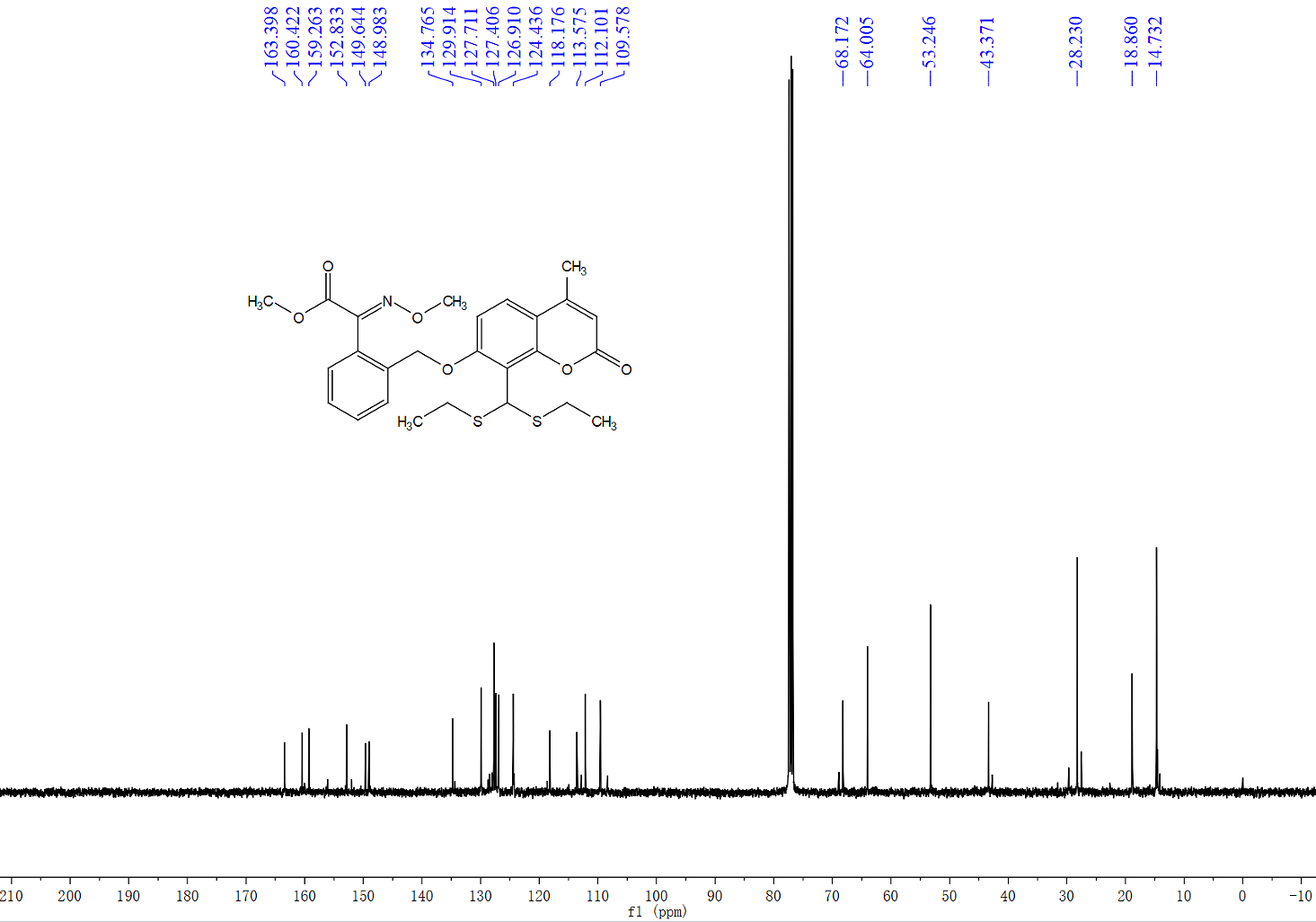


^13^C NMR of compound **D8**


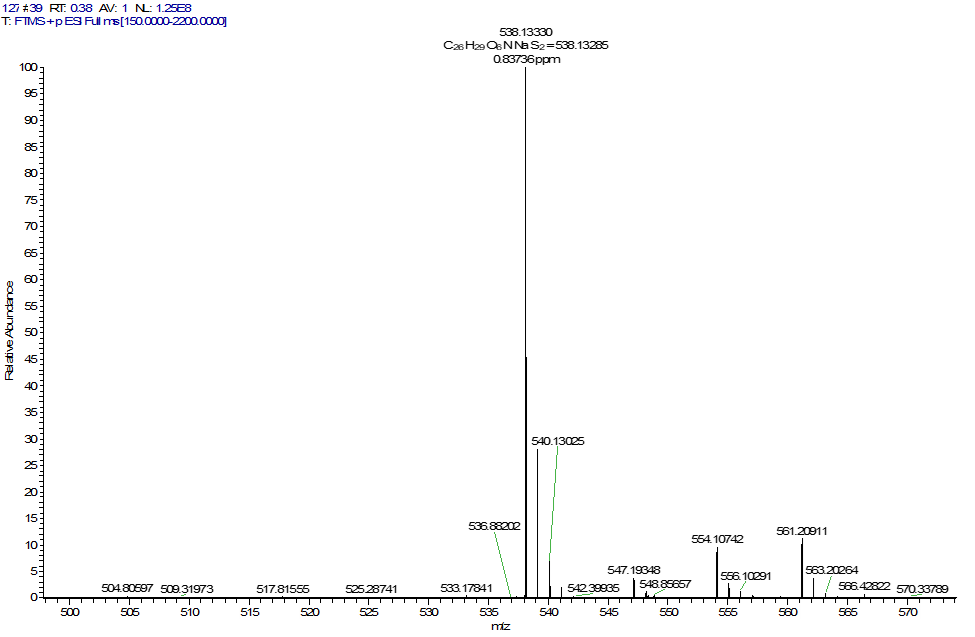


HRMS of compound **D8**


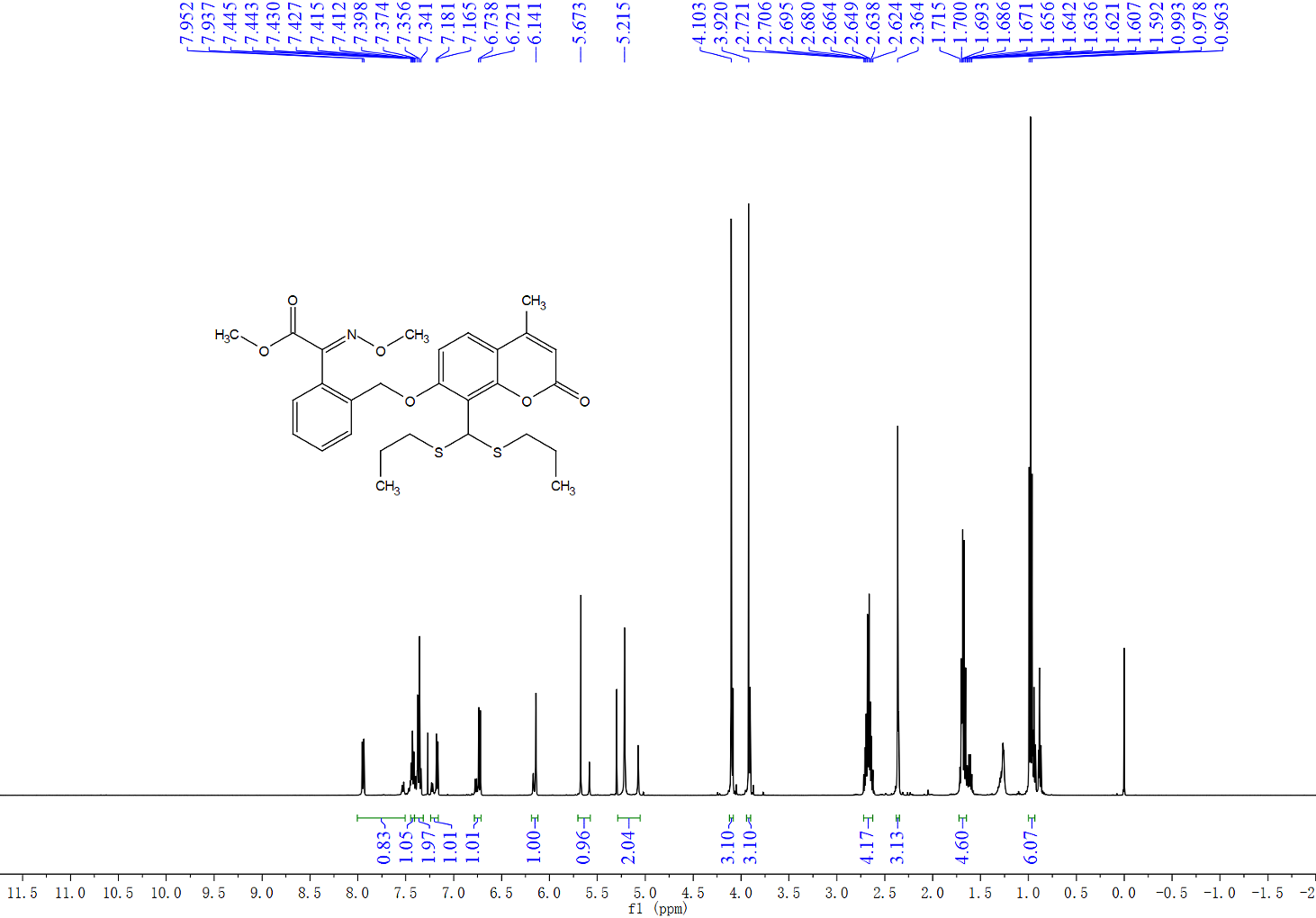


^1^H NMR of compound **D9**


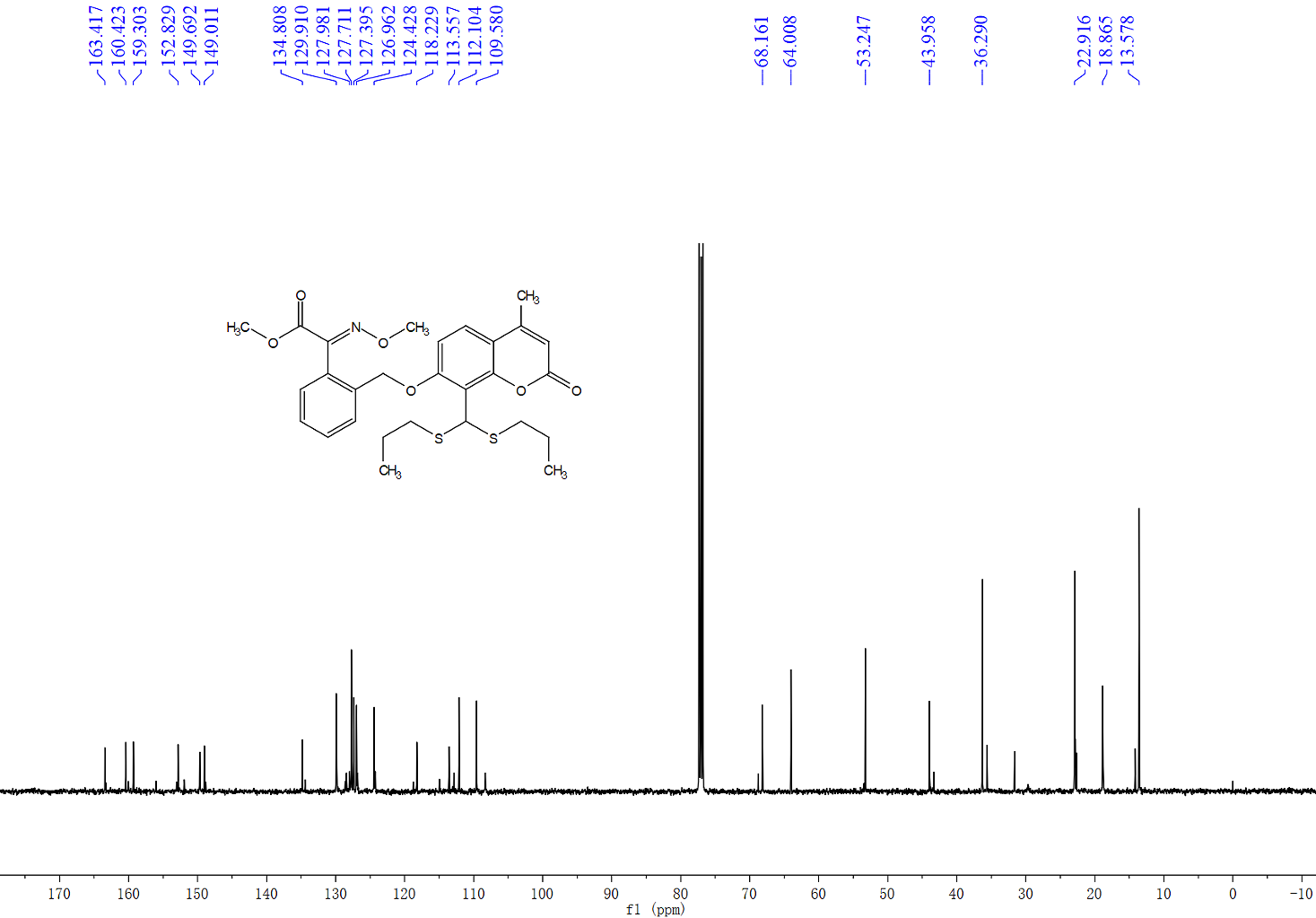


^13^C NMR of compound **D9**


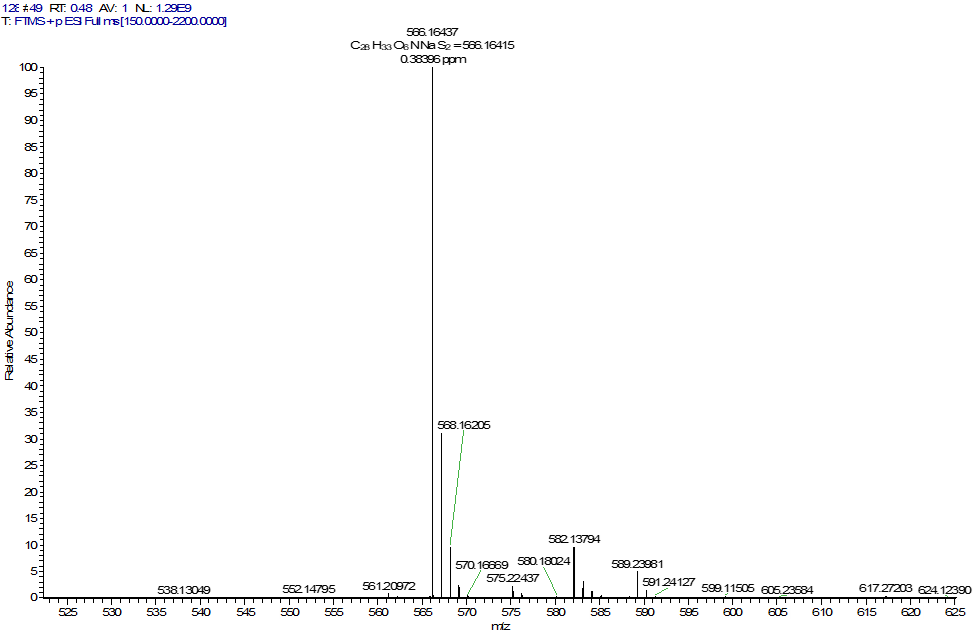


HRMS of compound **D9**


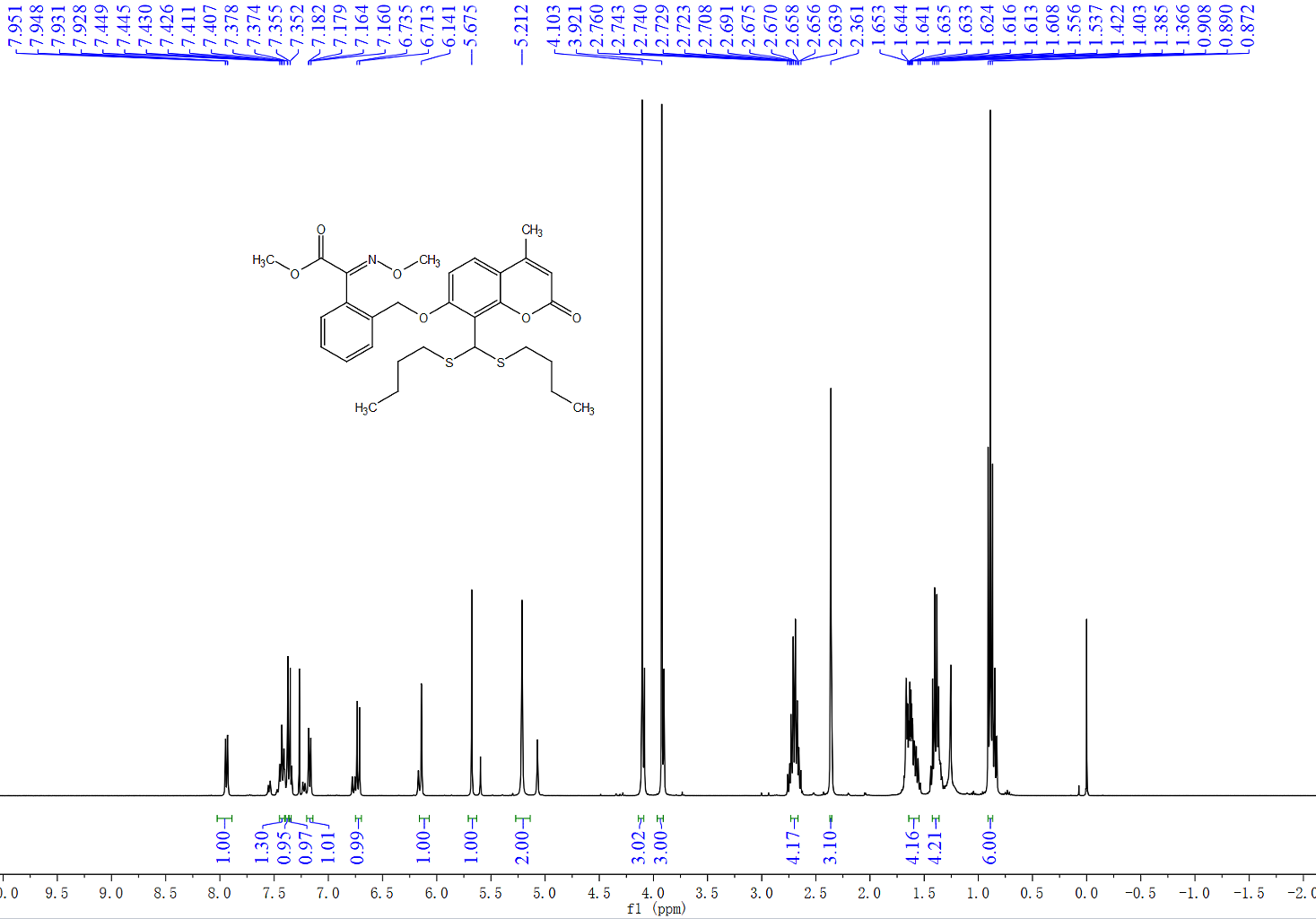


^1^H NMR of compound **D10**


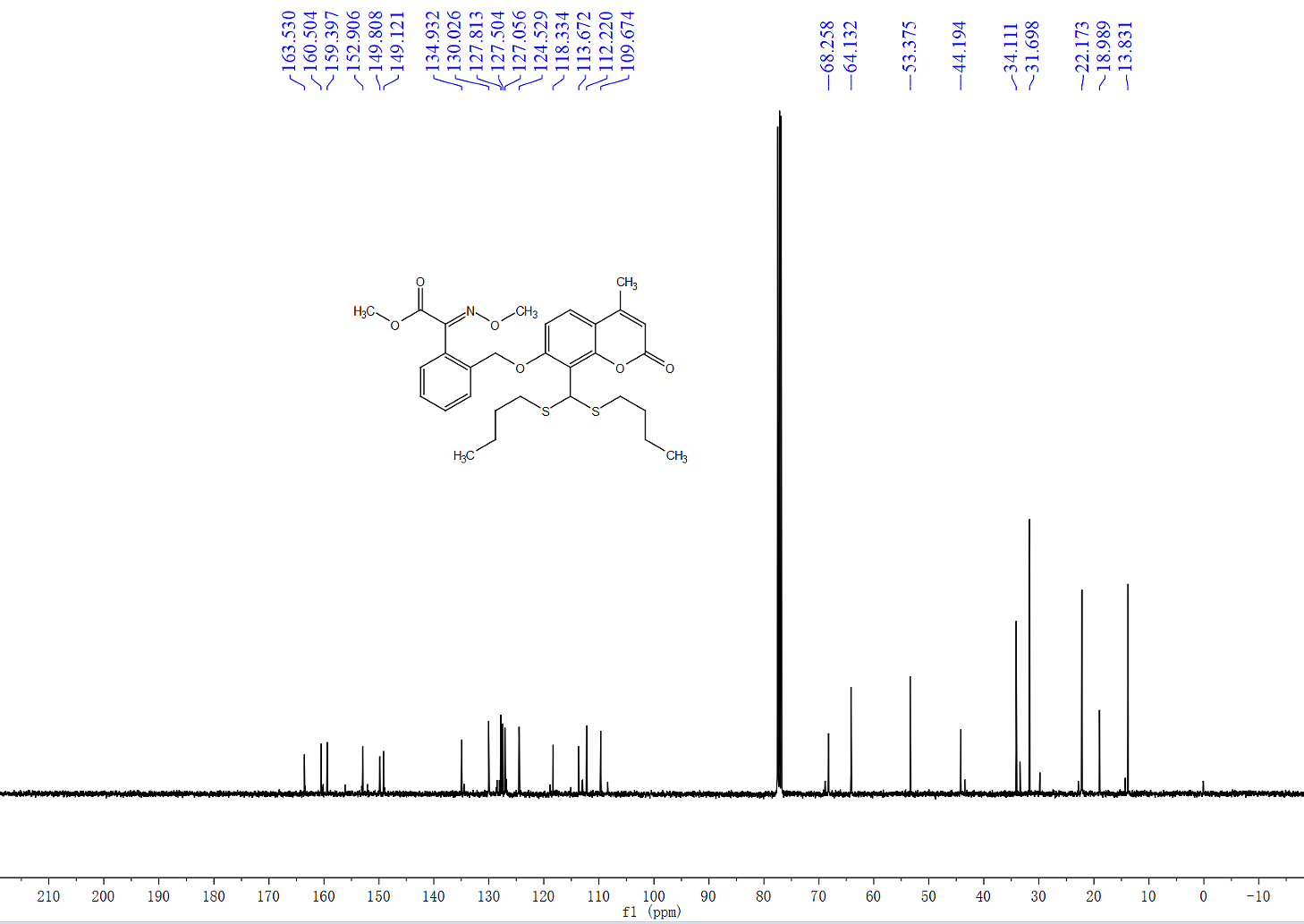


^13^C NMR of compound **D10**


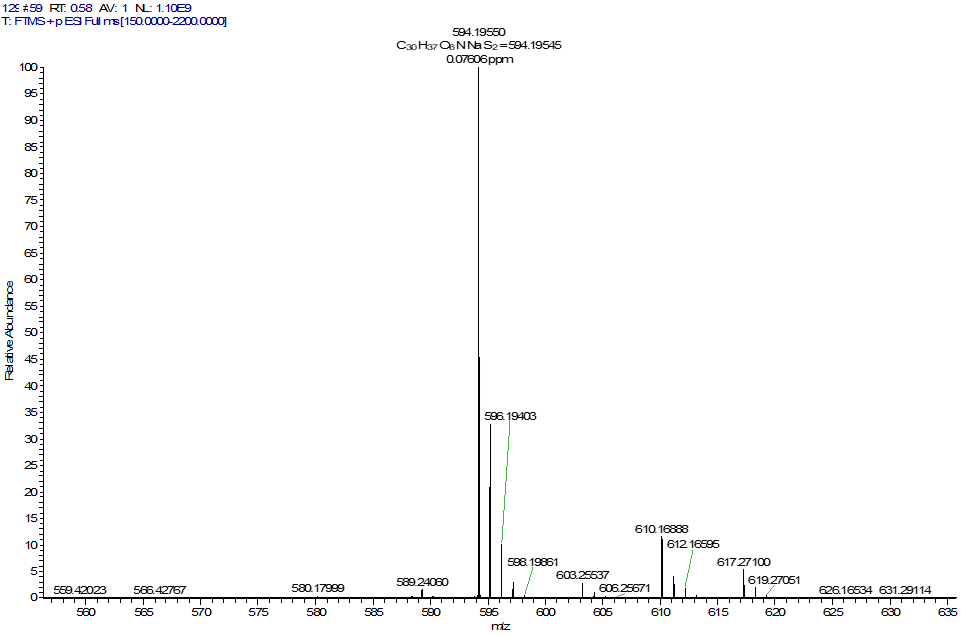


HRMS of compound **D10**


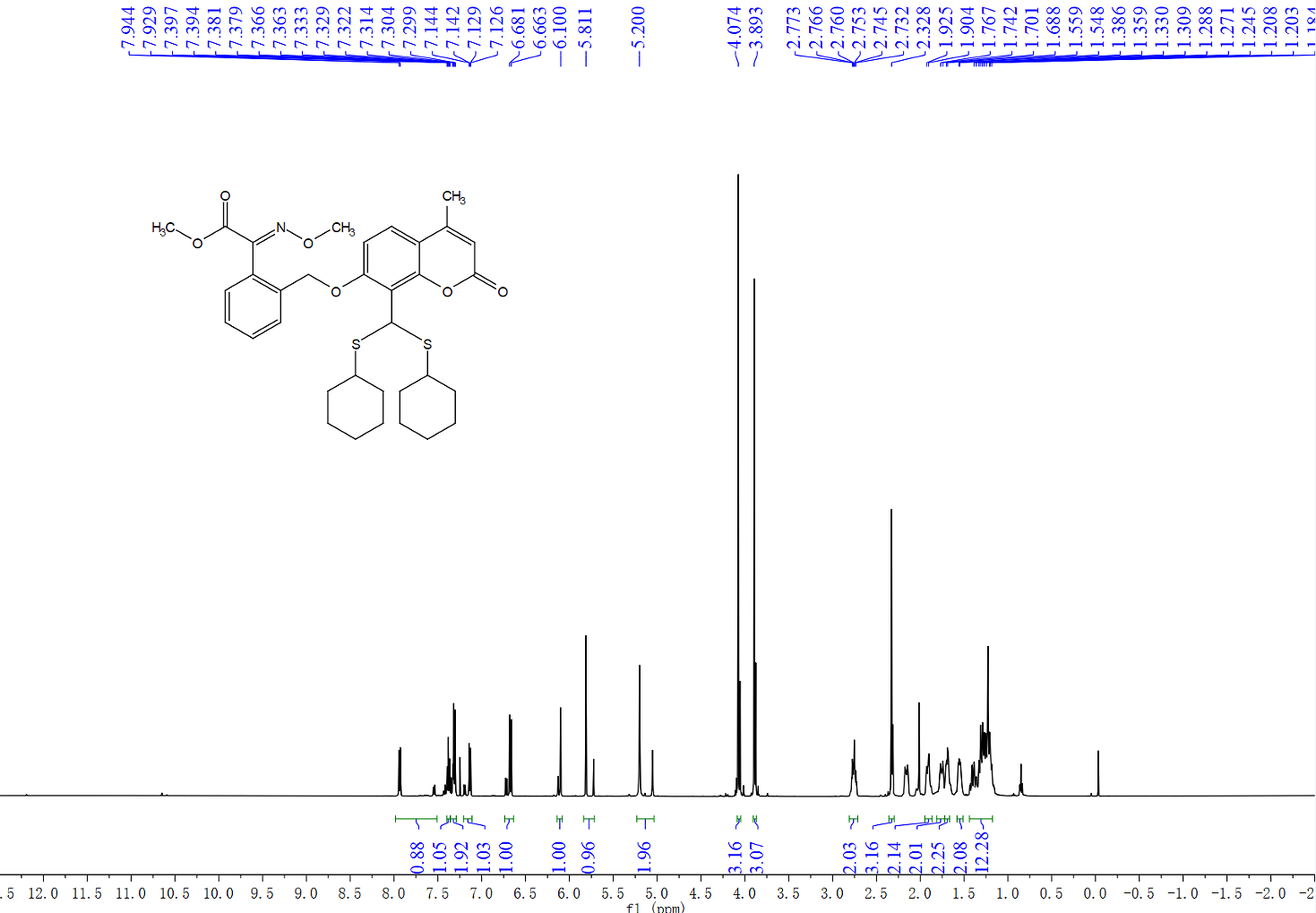


^1^H NMR of compound **D11**


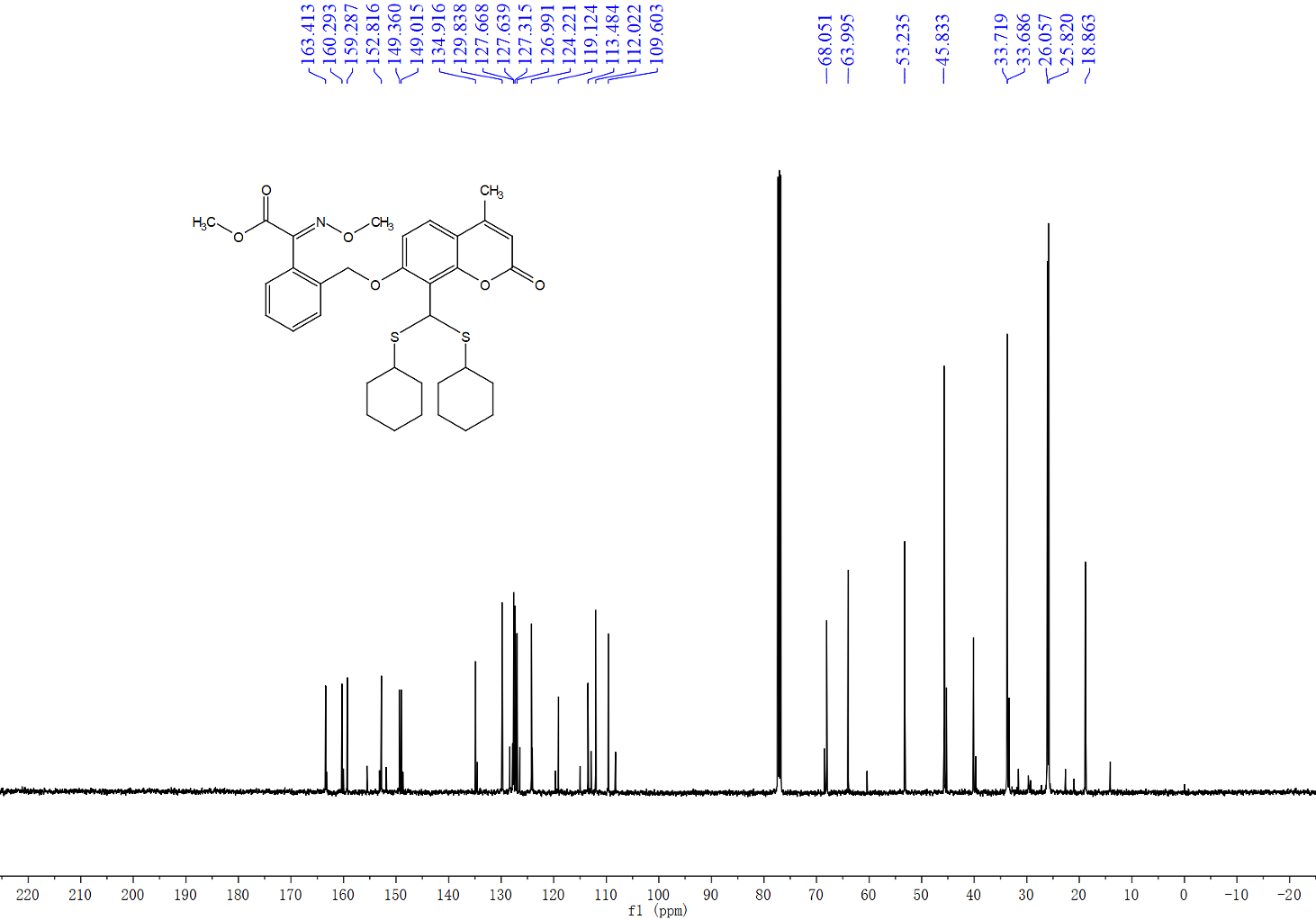


^13^C NMR of compound **D11**


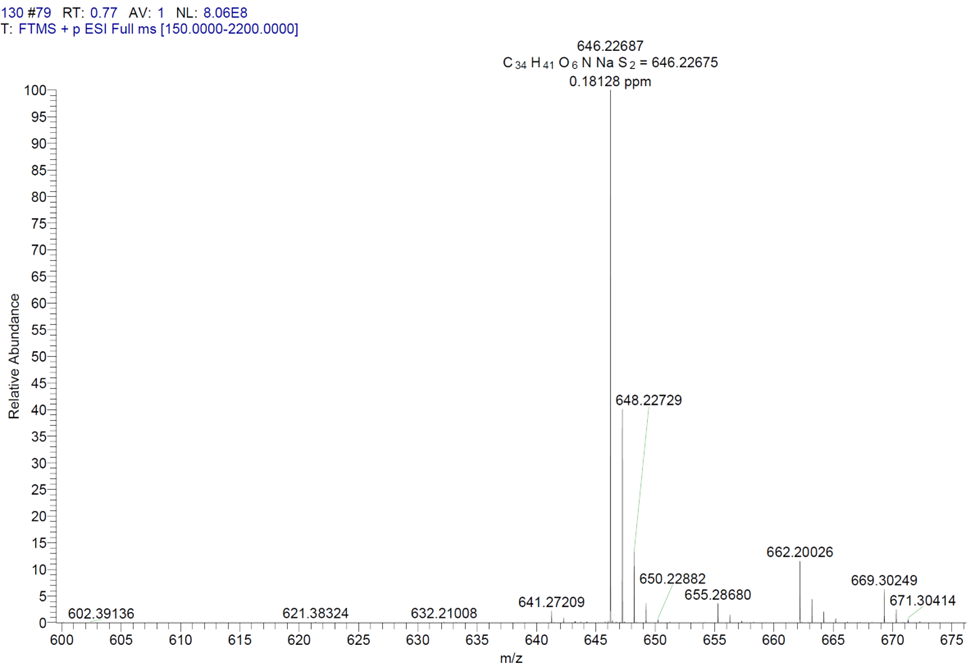


HRMS of compound **D11**


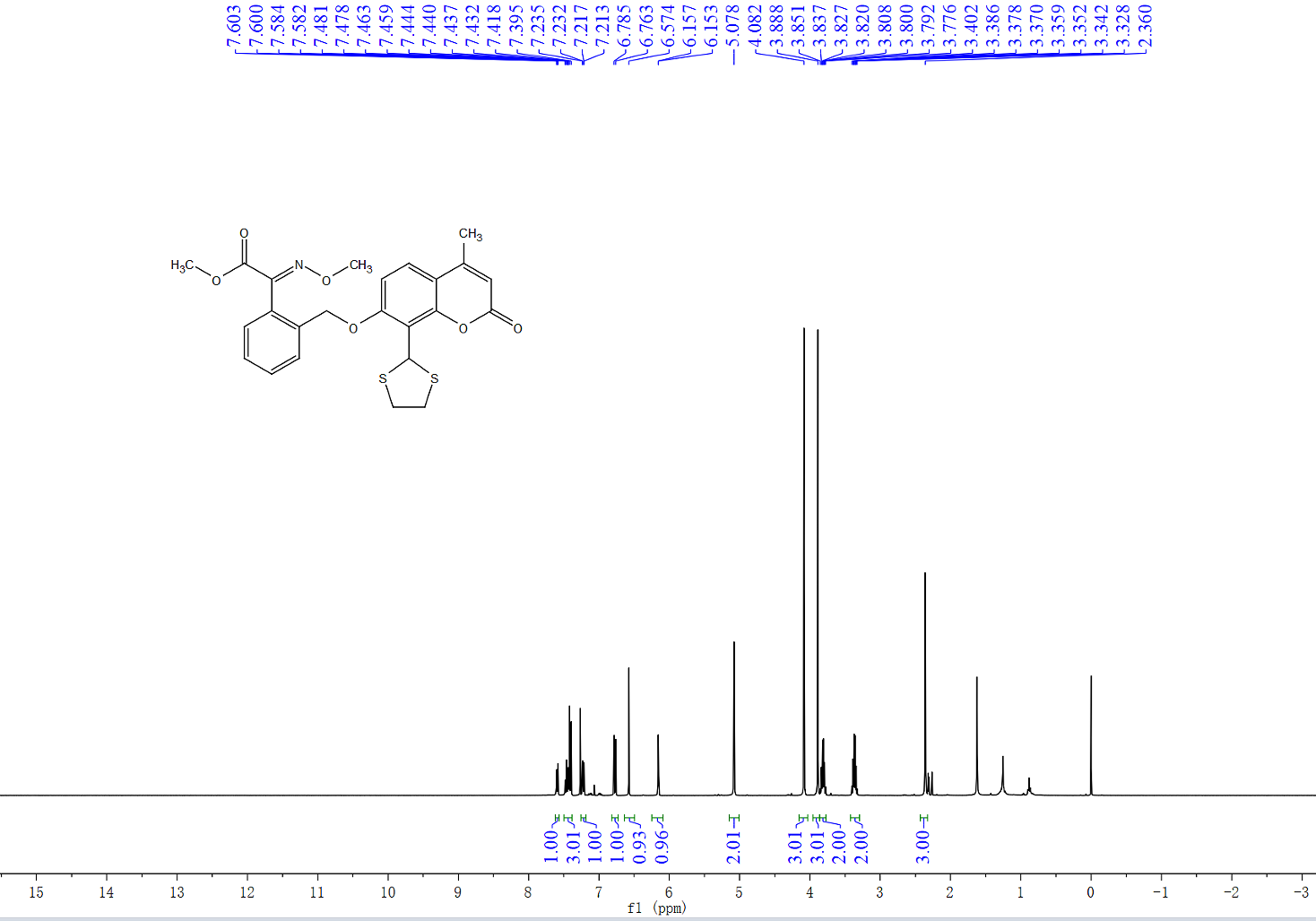


^1^H NMR of compound **D12**


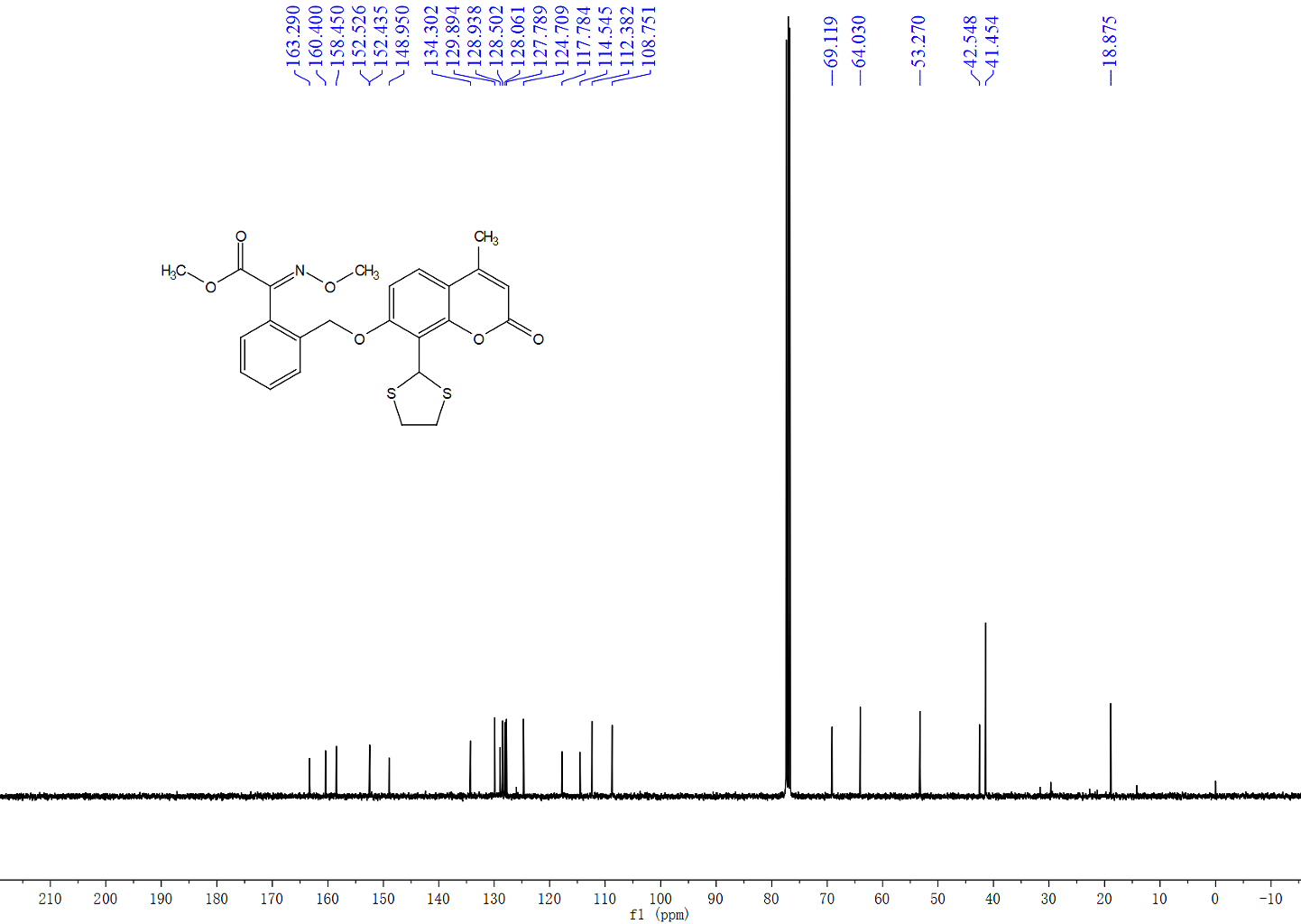


^13^C NMR of compound **D12**


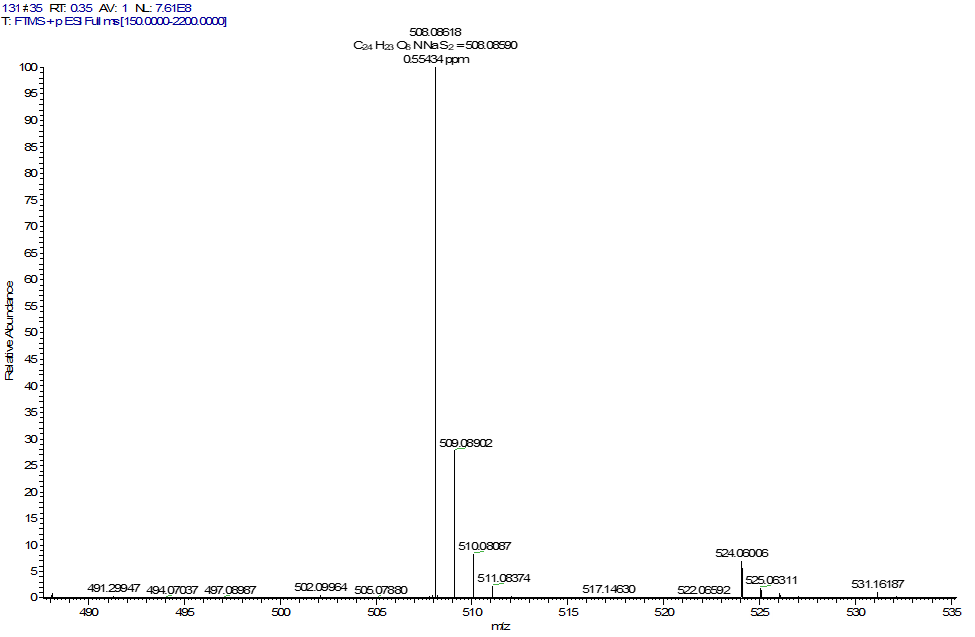


HRMS of compound **D12**


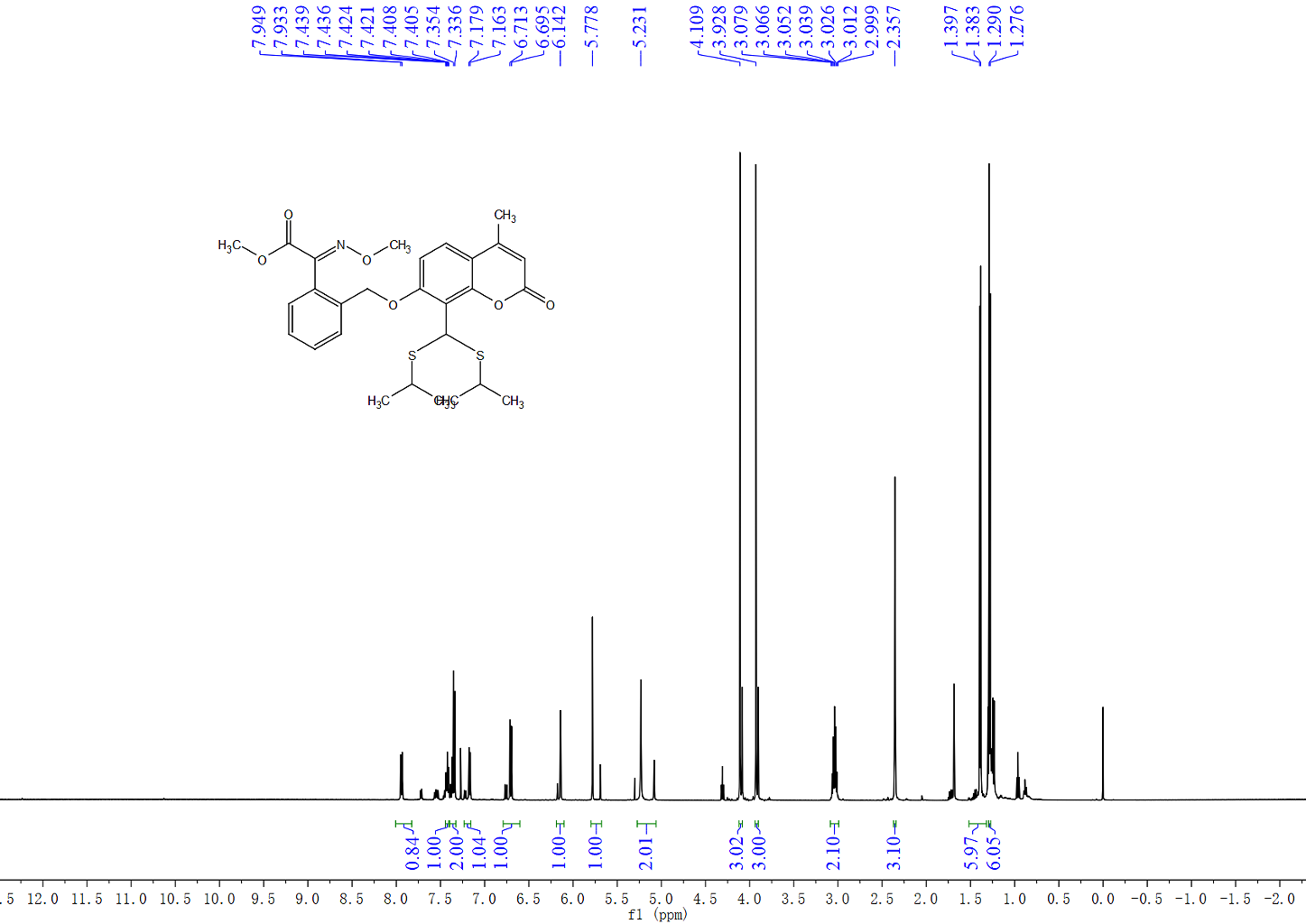


^1^H NMR of compound **D13**


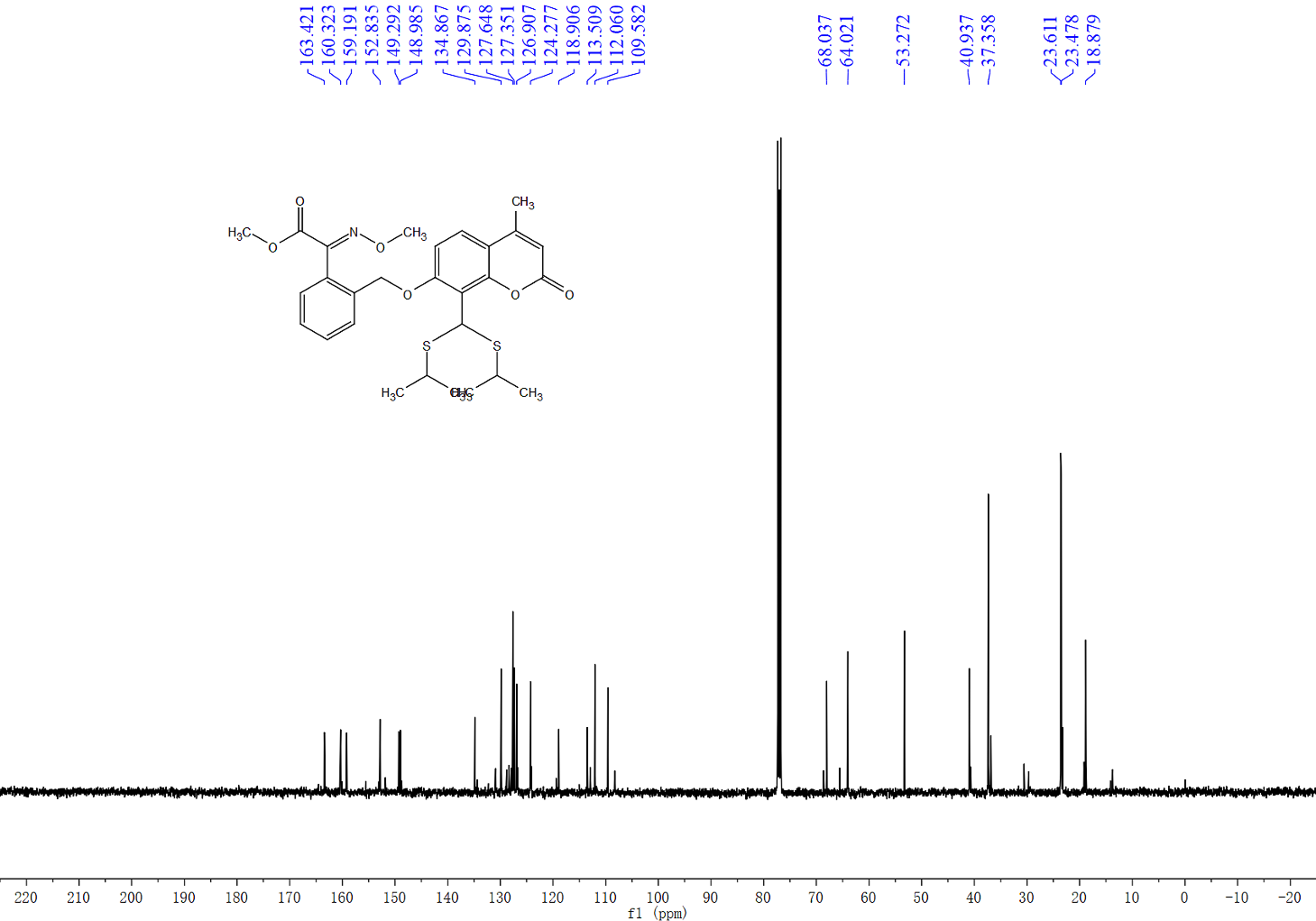


^13^C NMR of compound **D13**


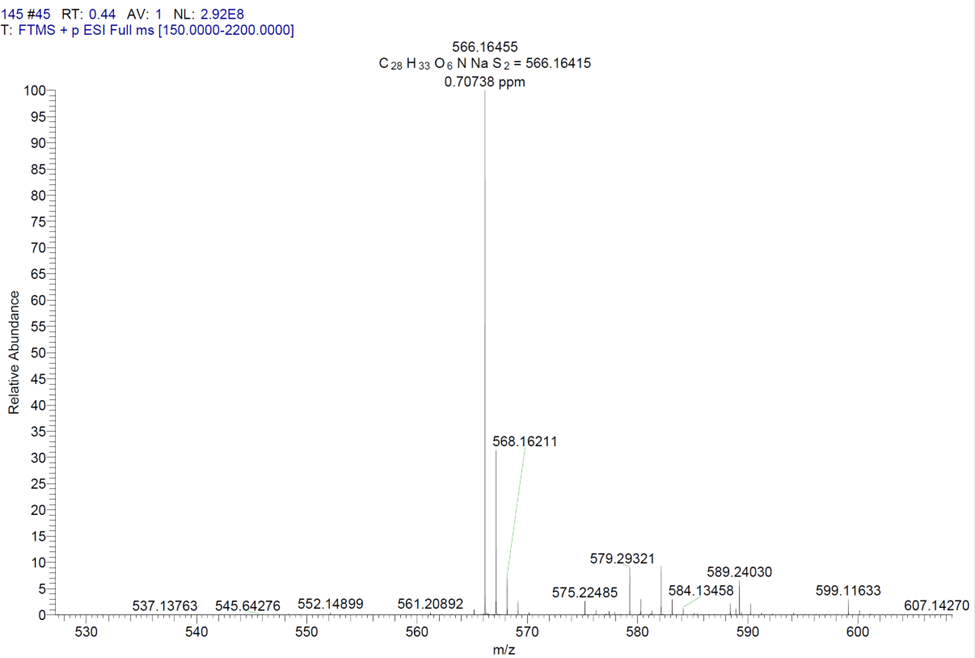


HRMS of compound **D13**


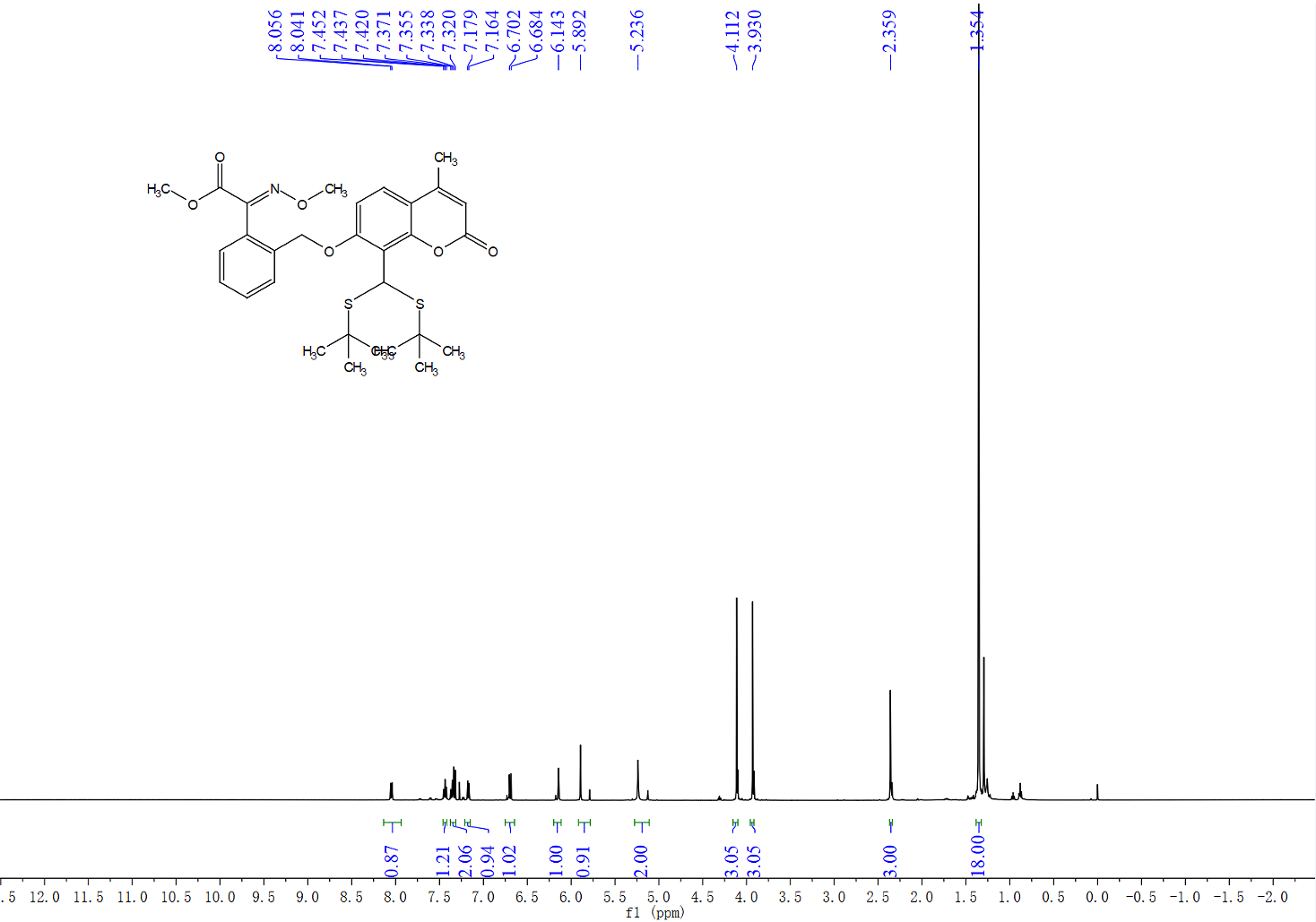


^1^H NMR of compound **D14**


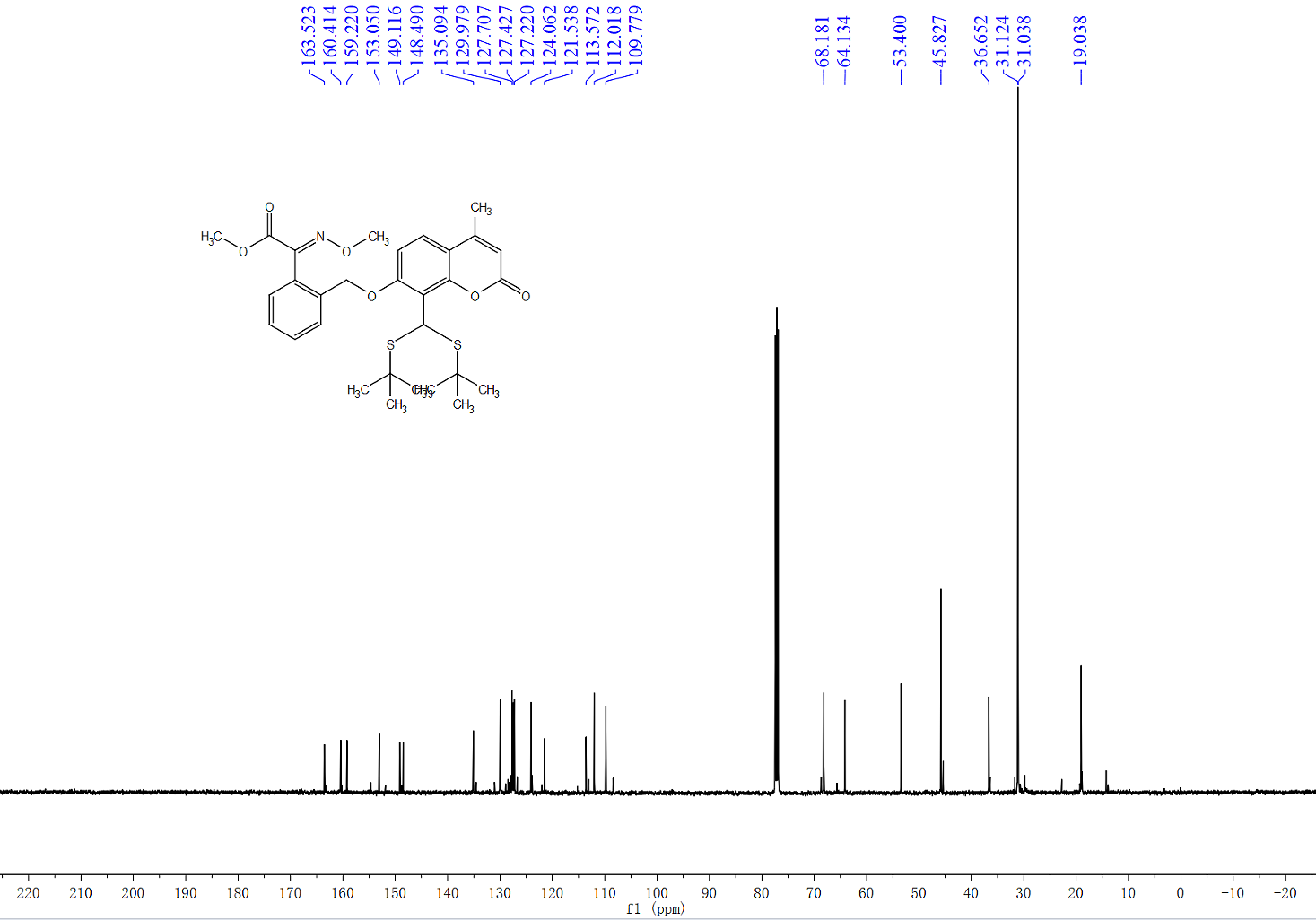


^13^C NMR of compound **D14**


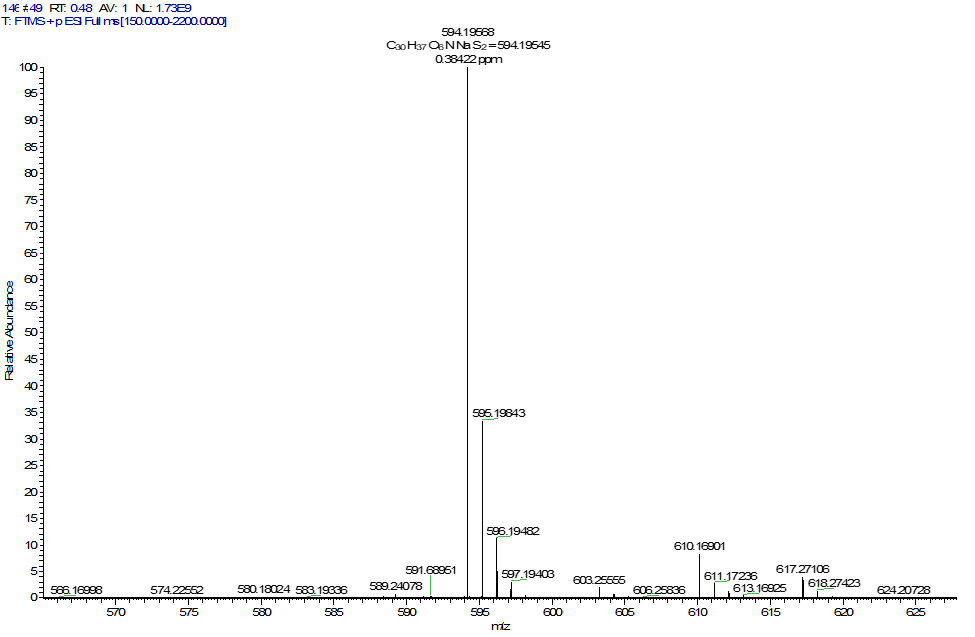


HRMS of compound **D14**


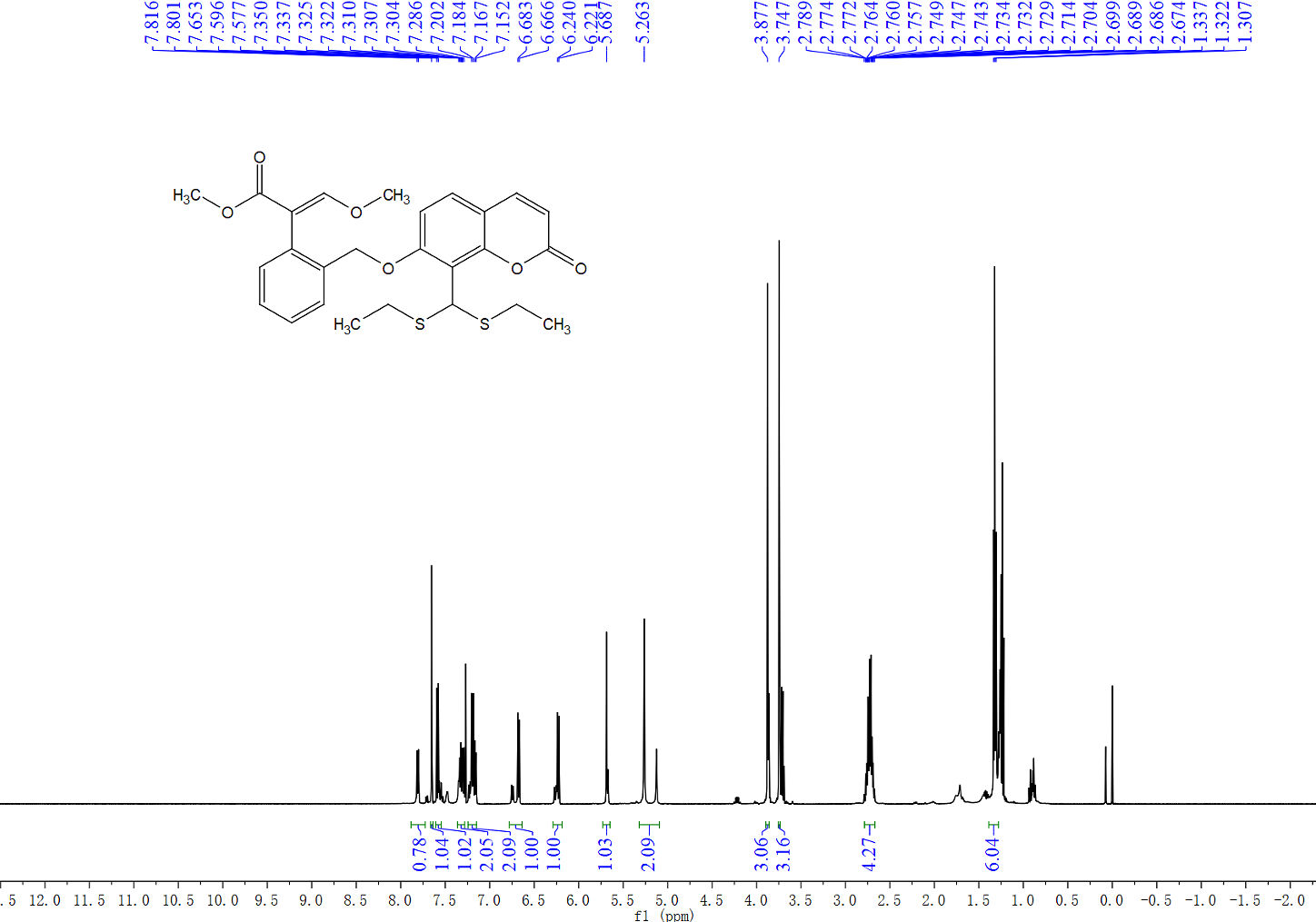


^1^H NMR of compound **D15**


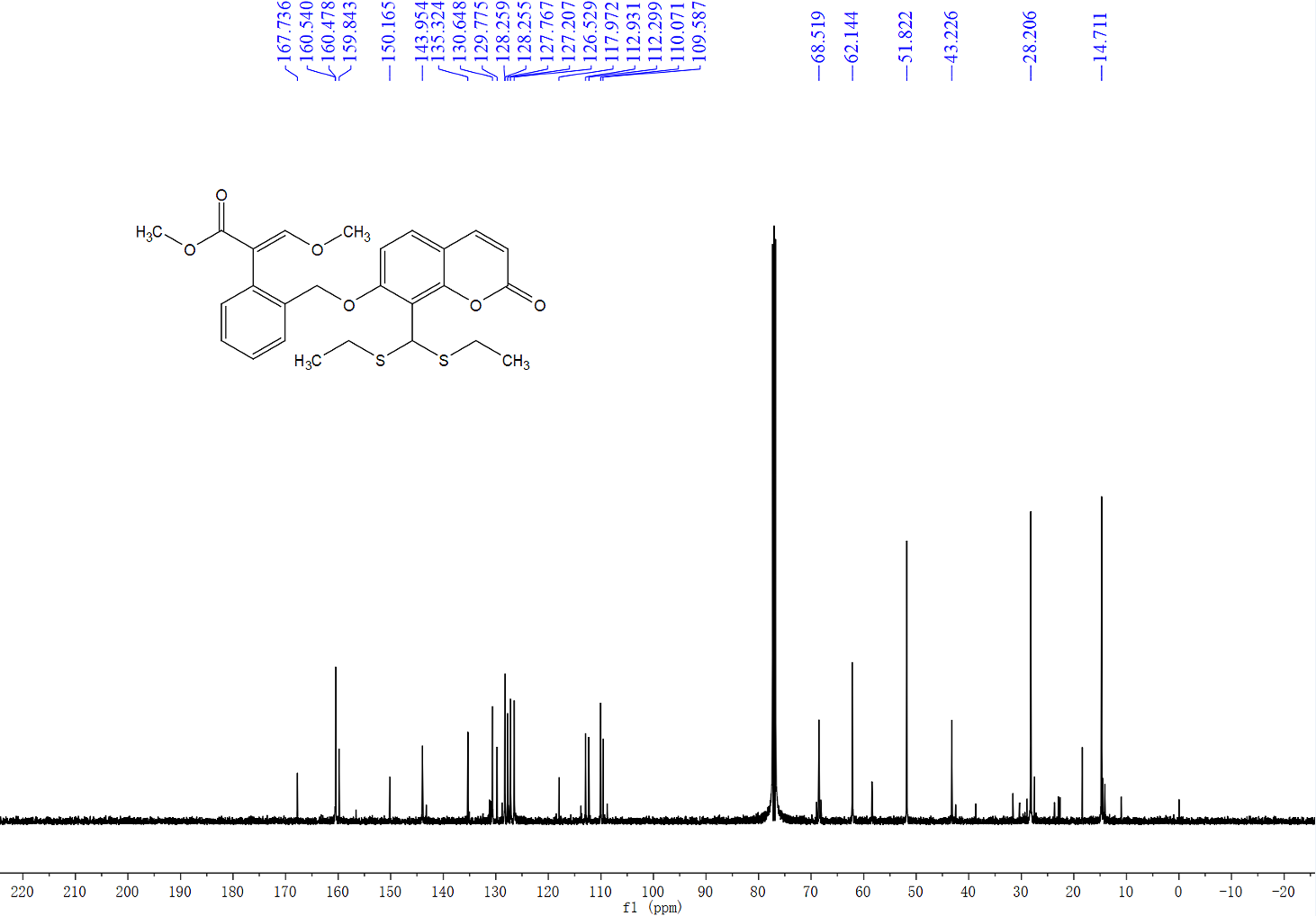


^13^C NMR of compound **D15**


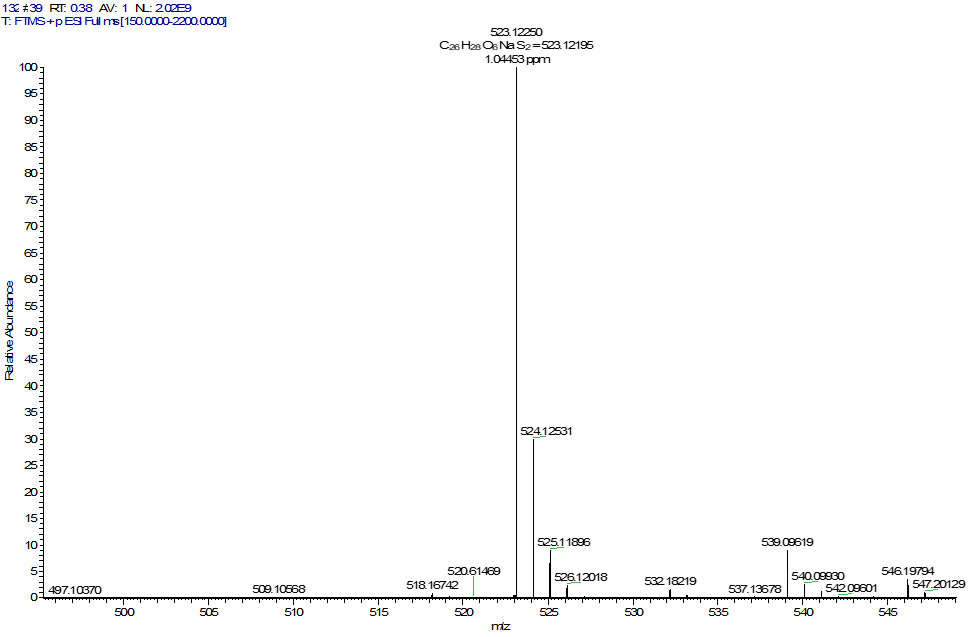


HRMS of compound **D15**


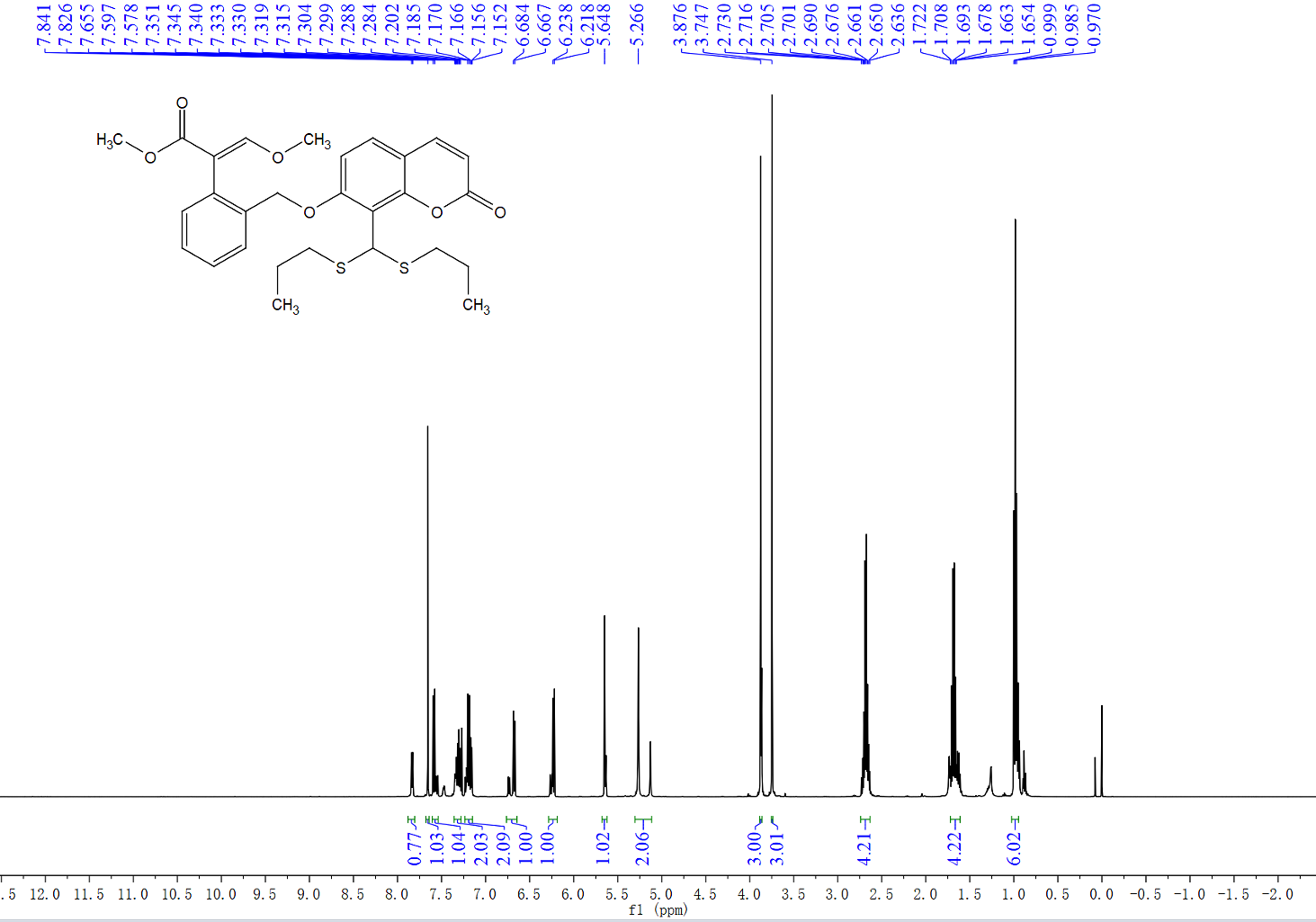


^1^H NMR of compound **D16**


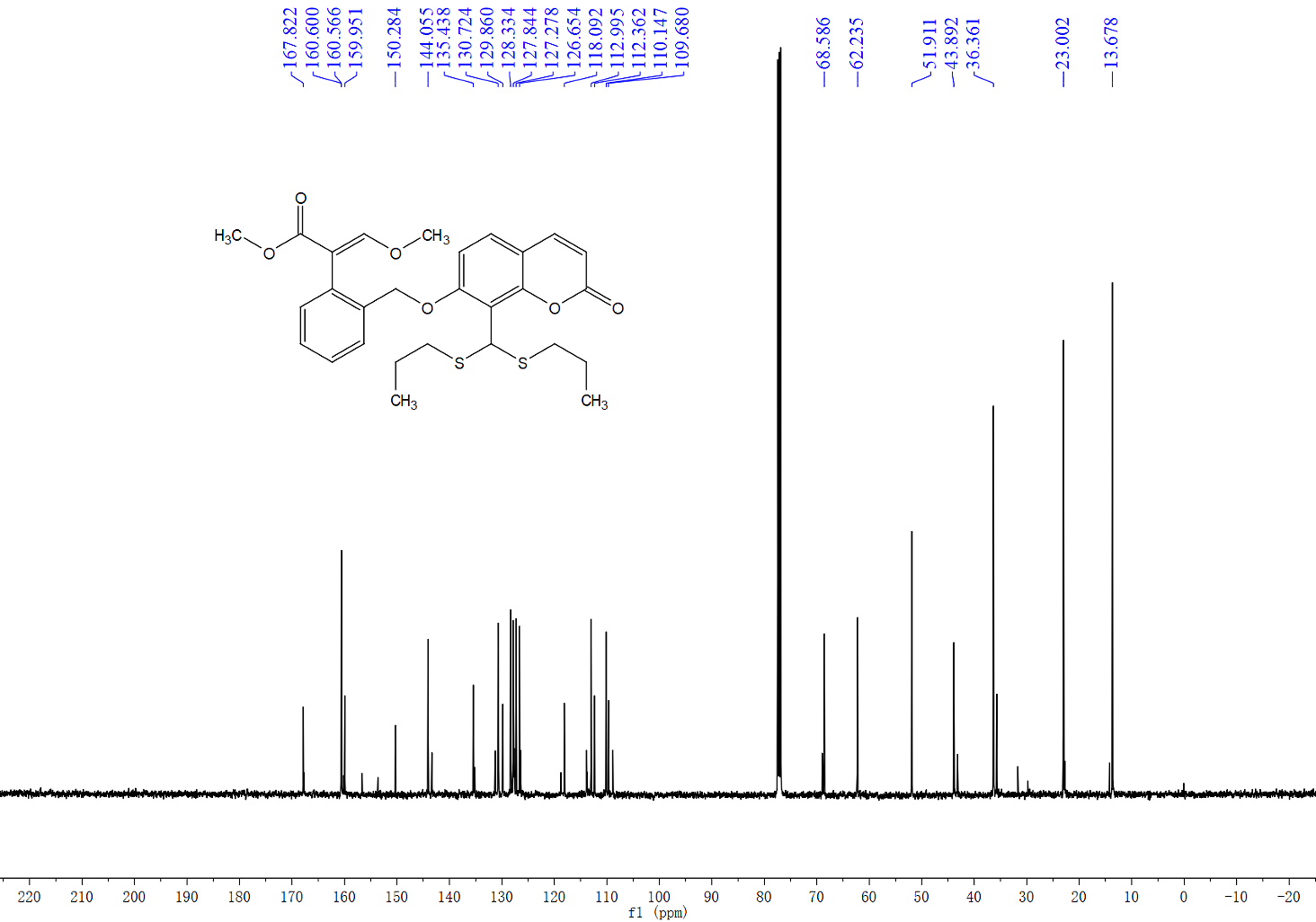


^13^C NMR of compound **D16**


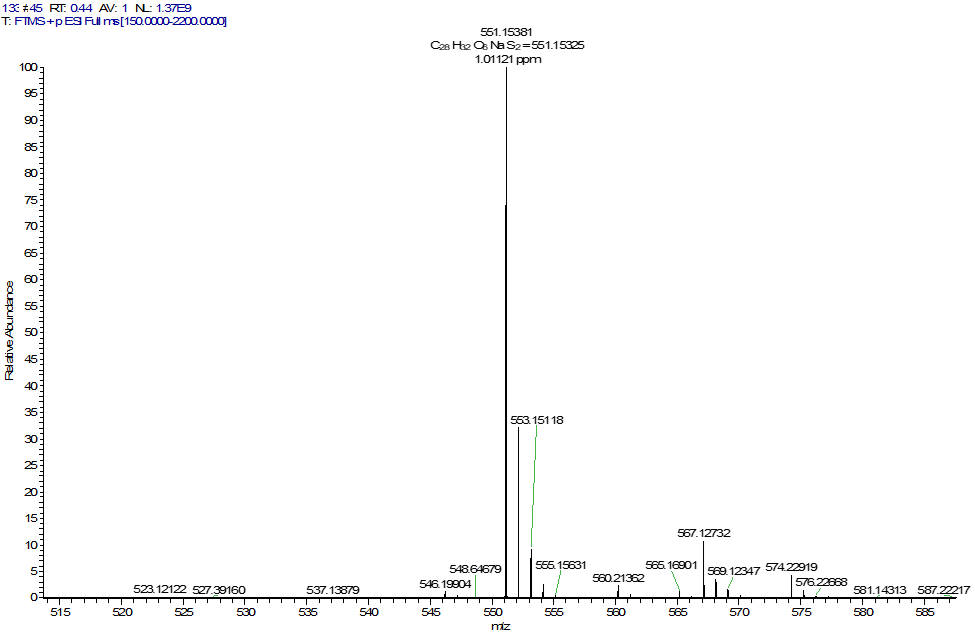


HRMS of compound **D16**


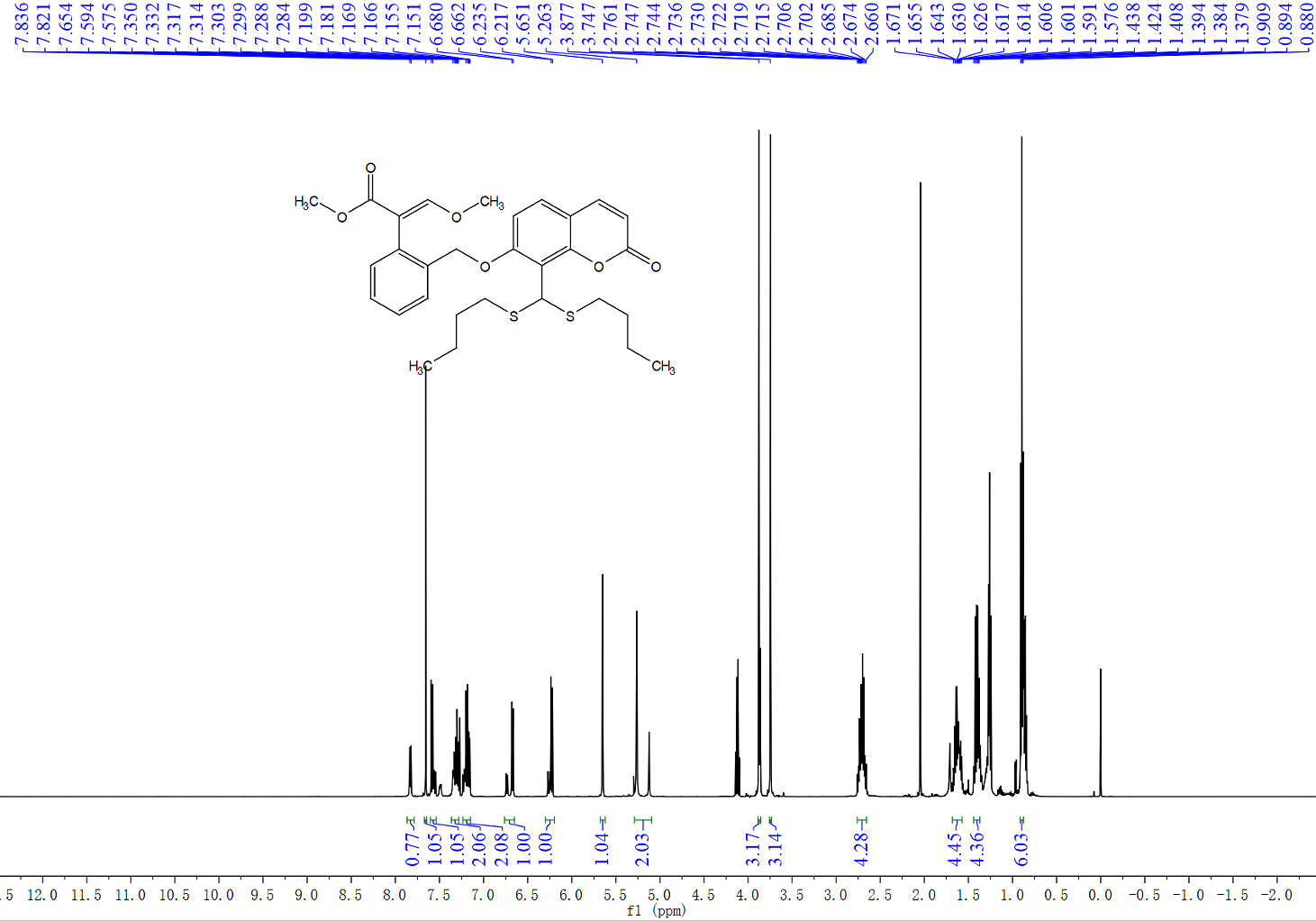


^1^H NMR of compound **D17**


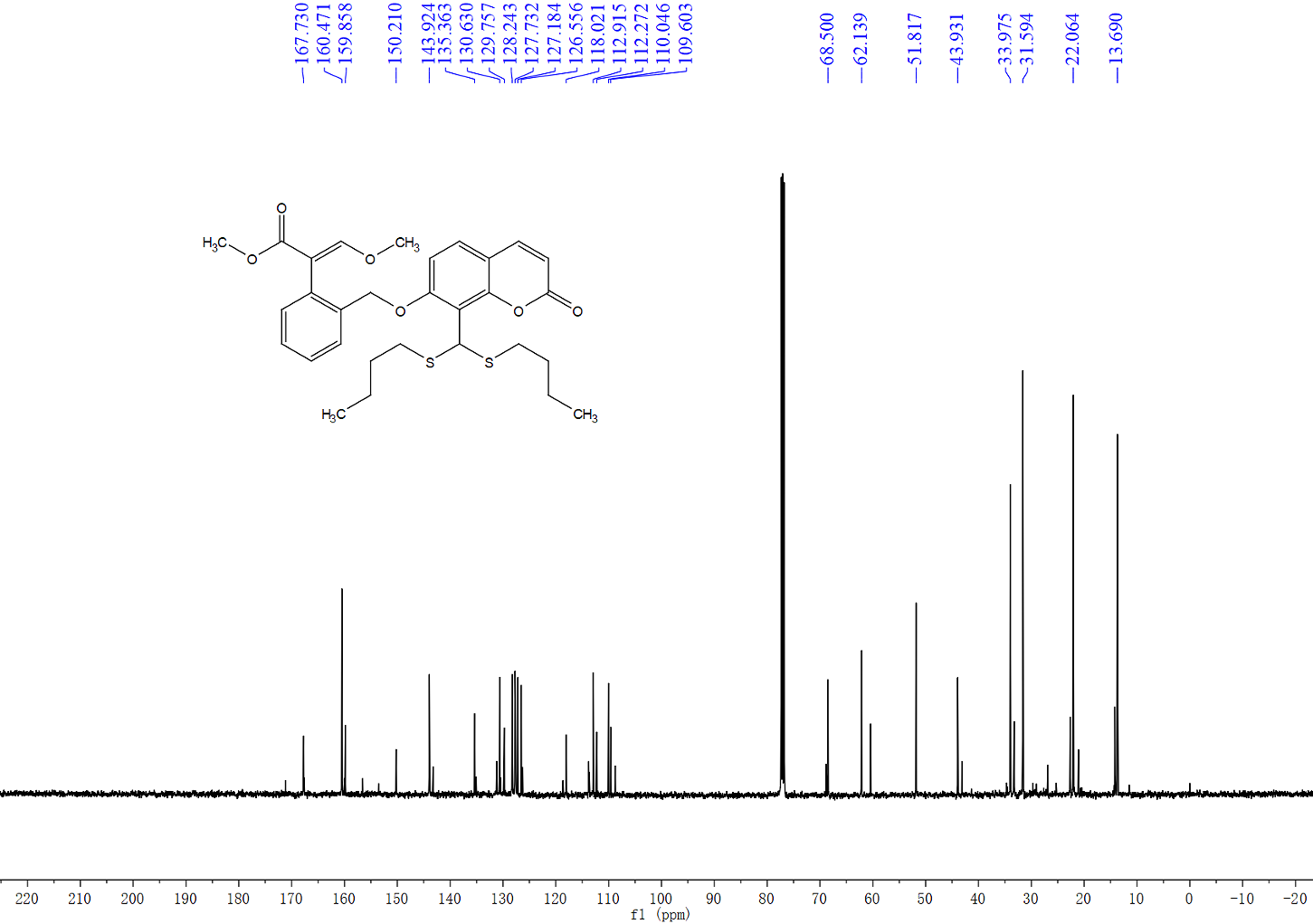


^13^C NMR of compound **D17**


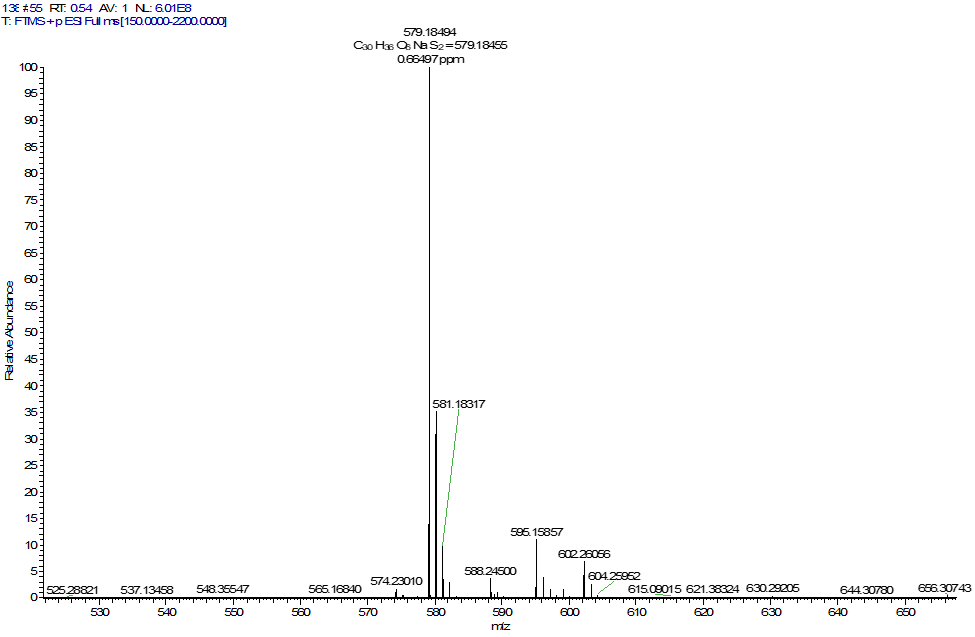


HRMS of compound **D17**


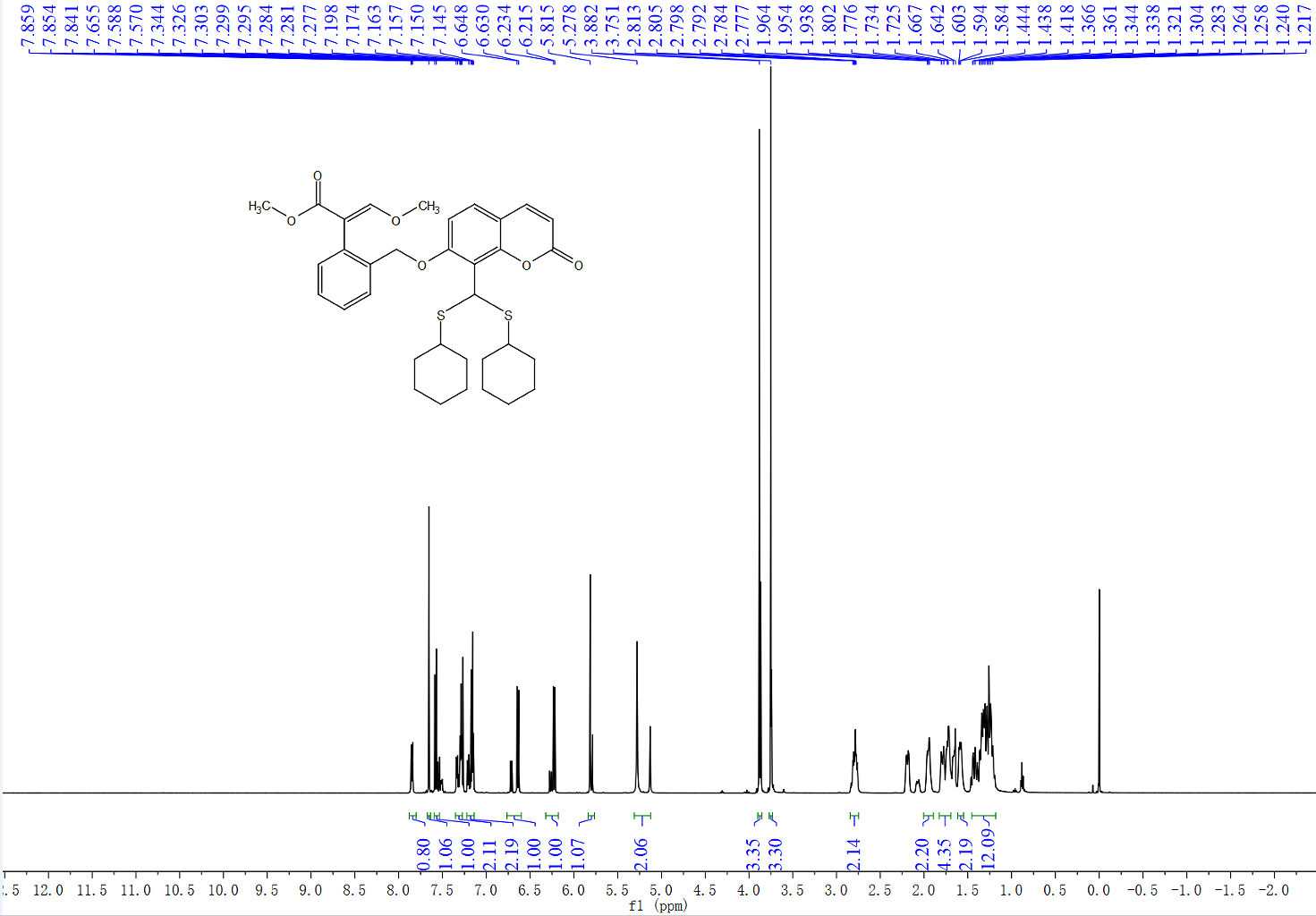


^1^H NMR of compound **D18**


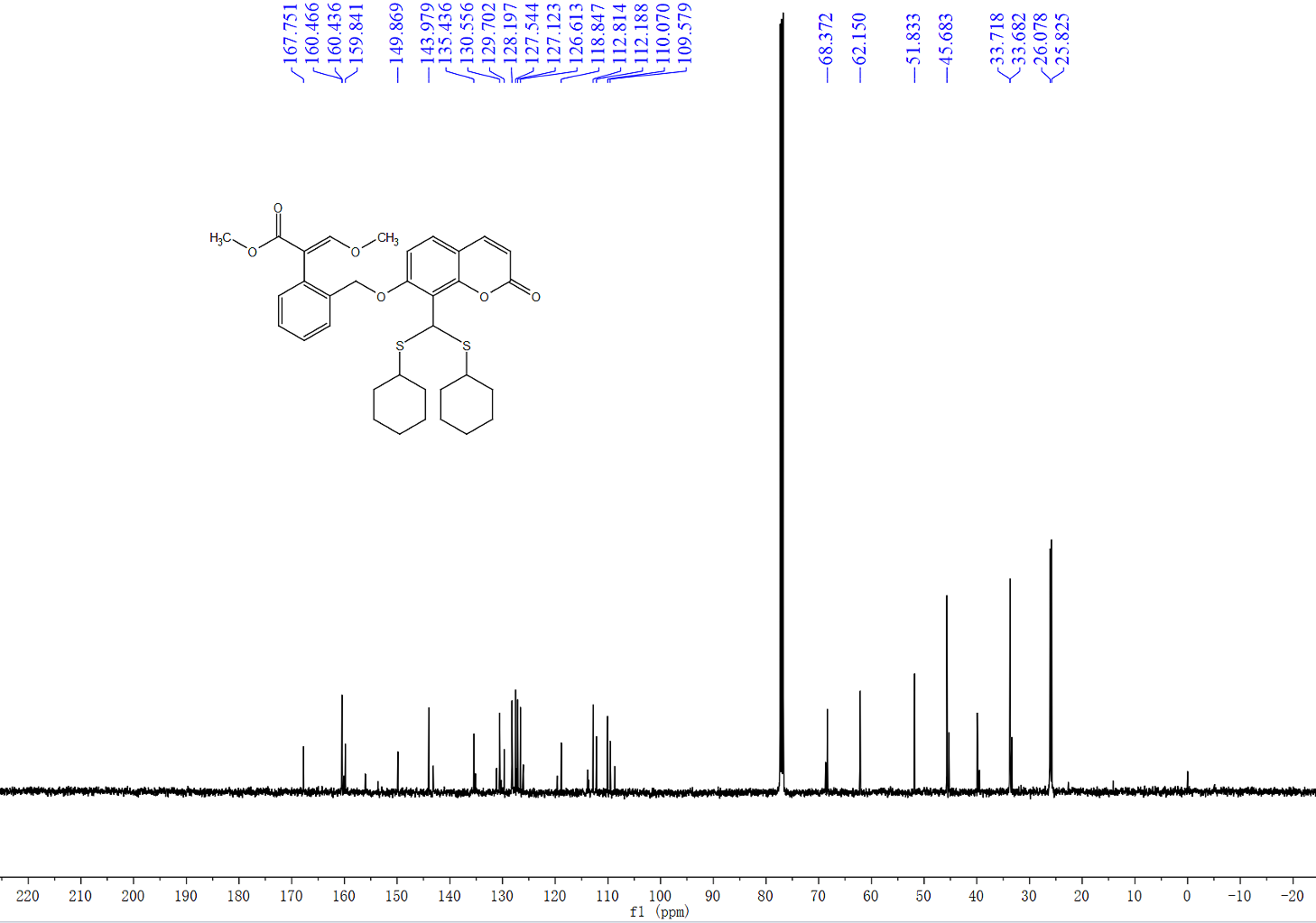


^13^C NMR of compound **D18**


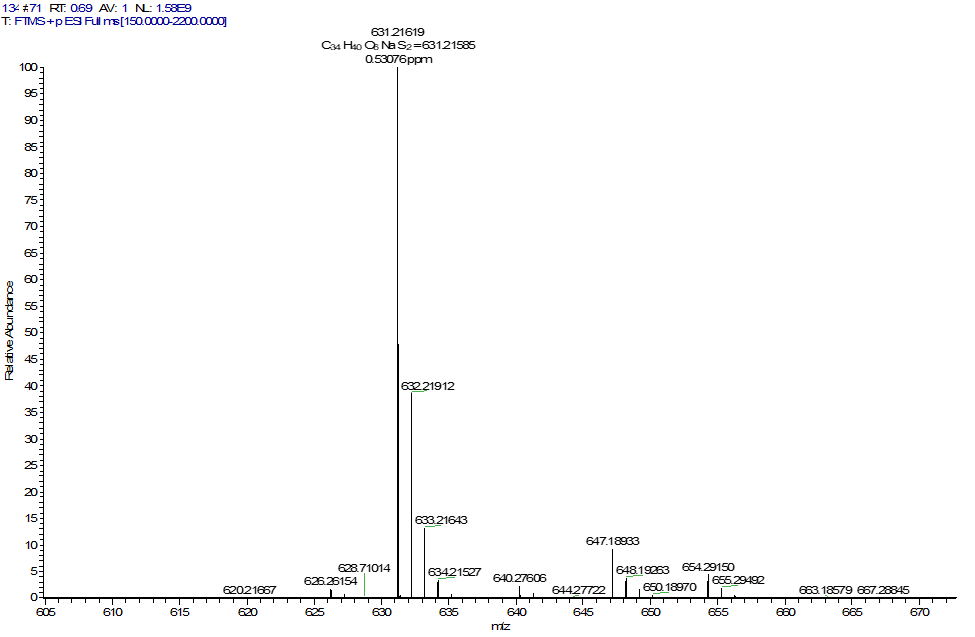


HRMS of compound **D18**


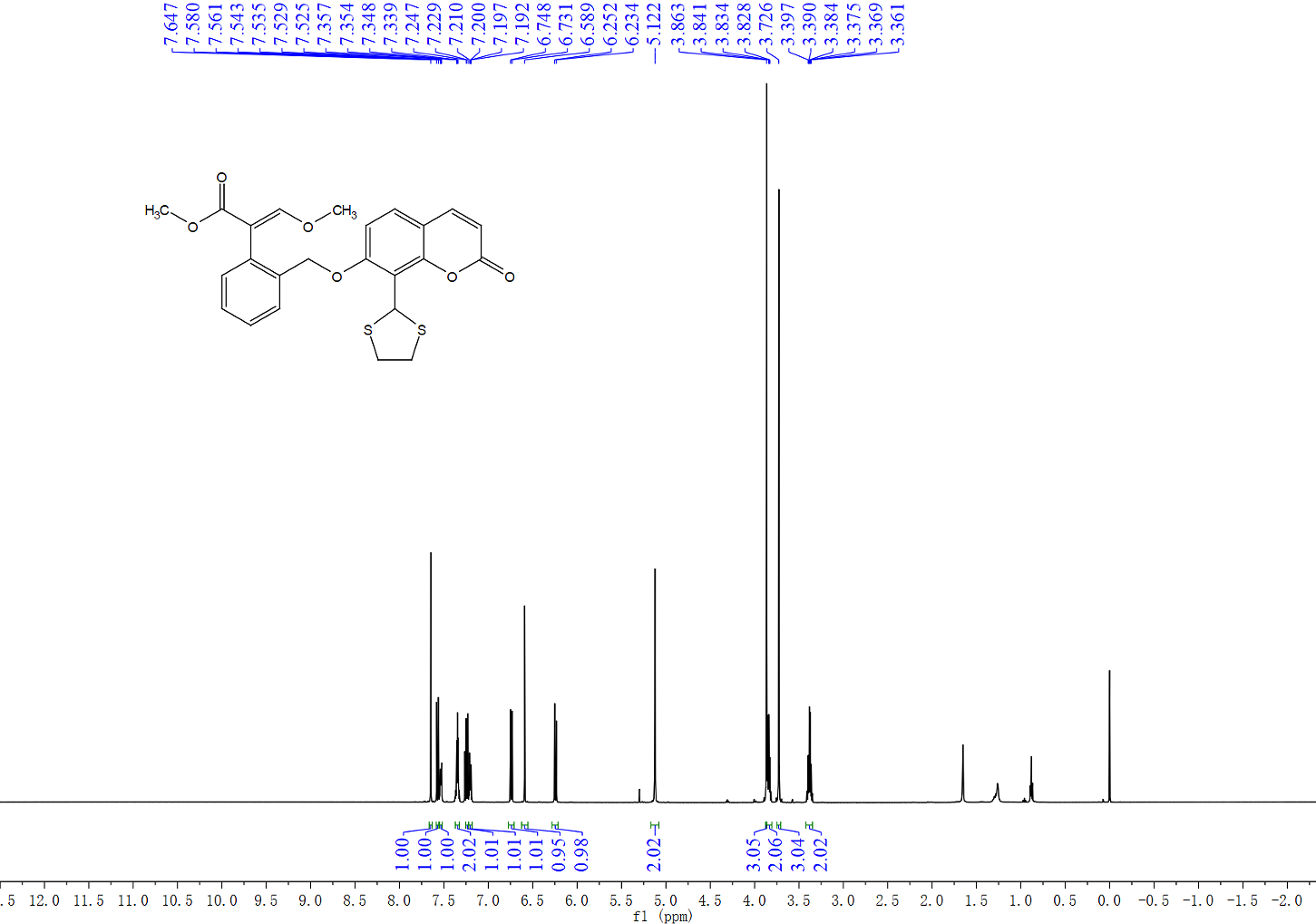


^1^H NMR of compound **D19**


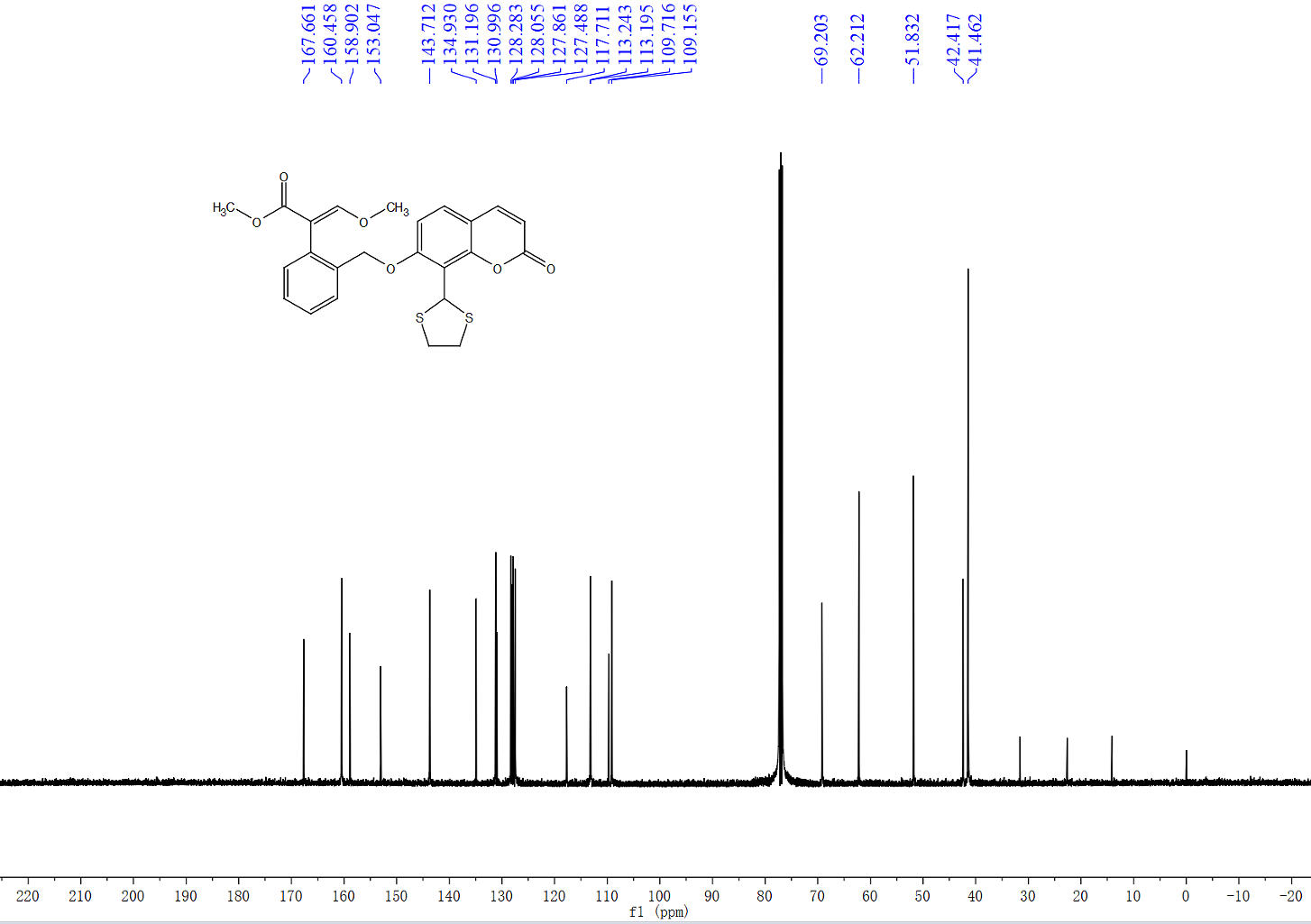


^13^C NMR of compound **D19**


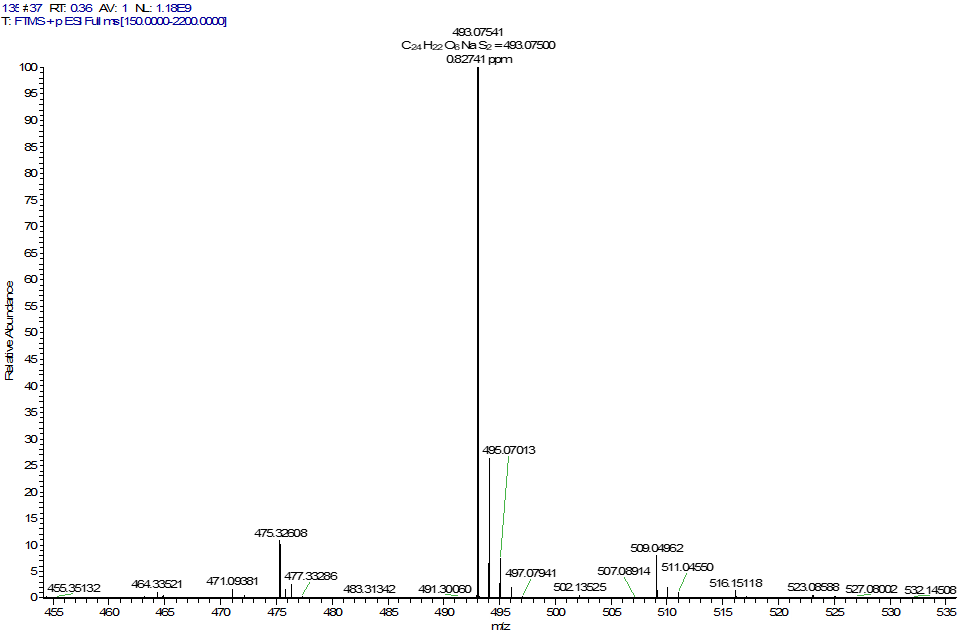


HRMS of compound **D19**


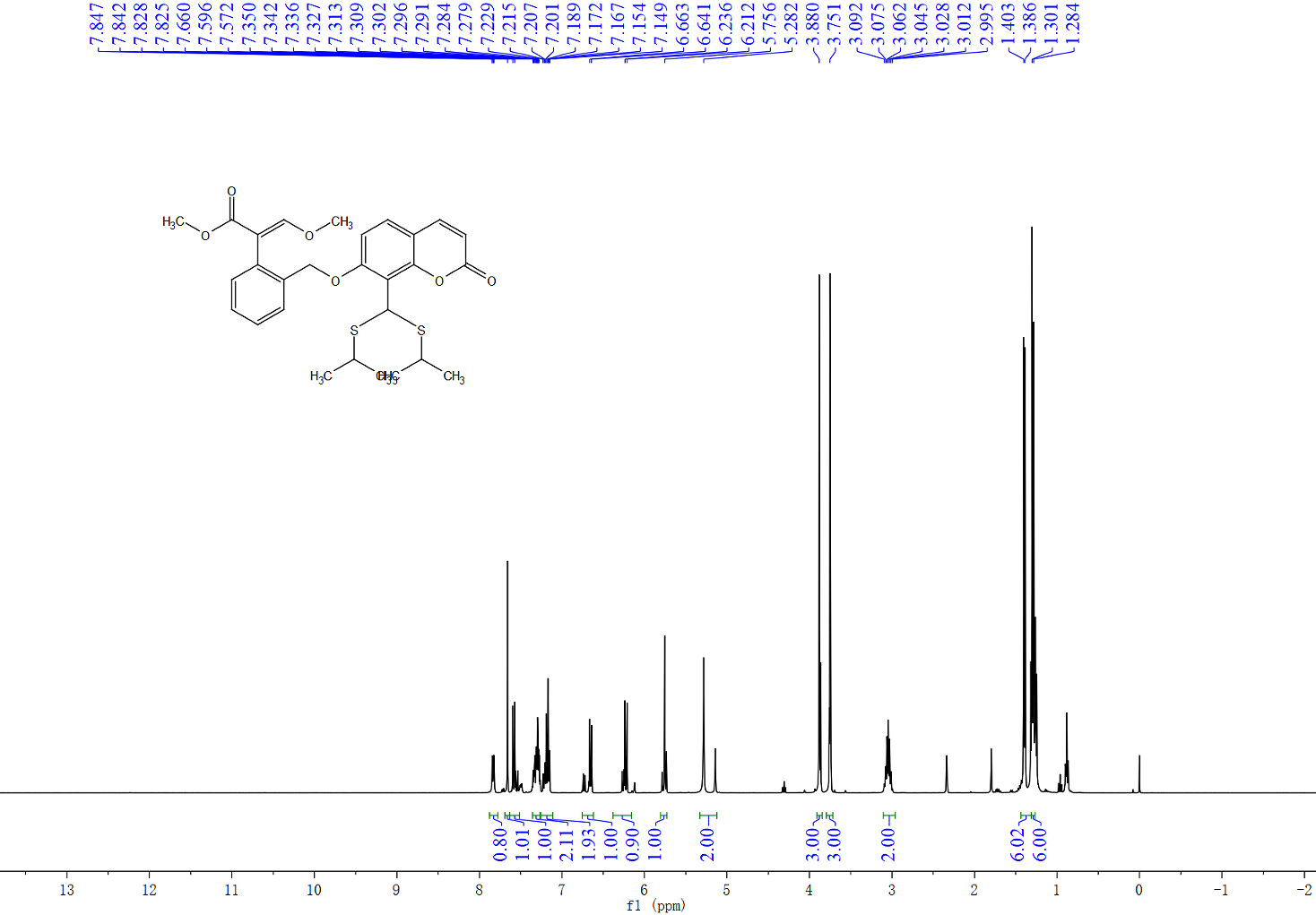


^1^H NMR of compound **D20**


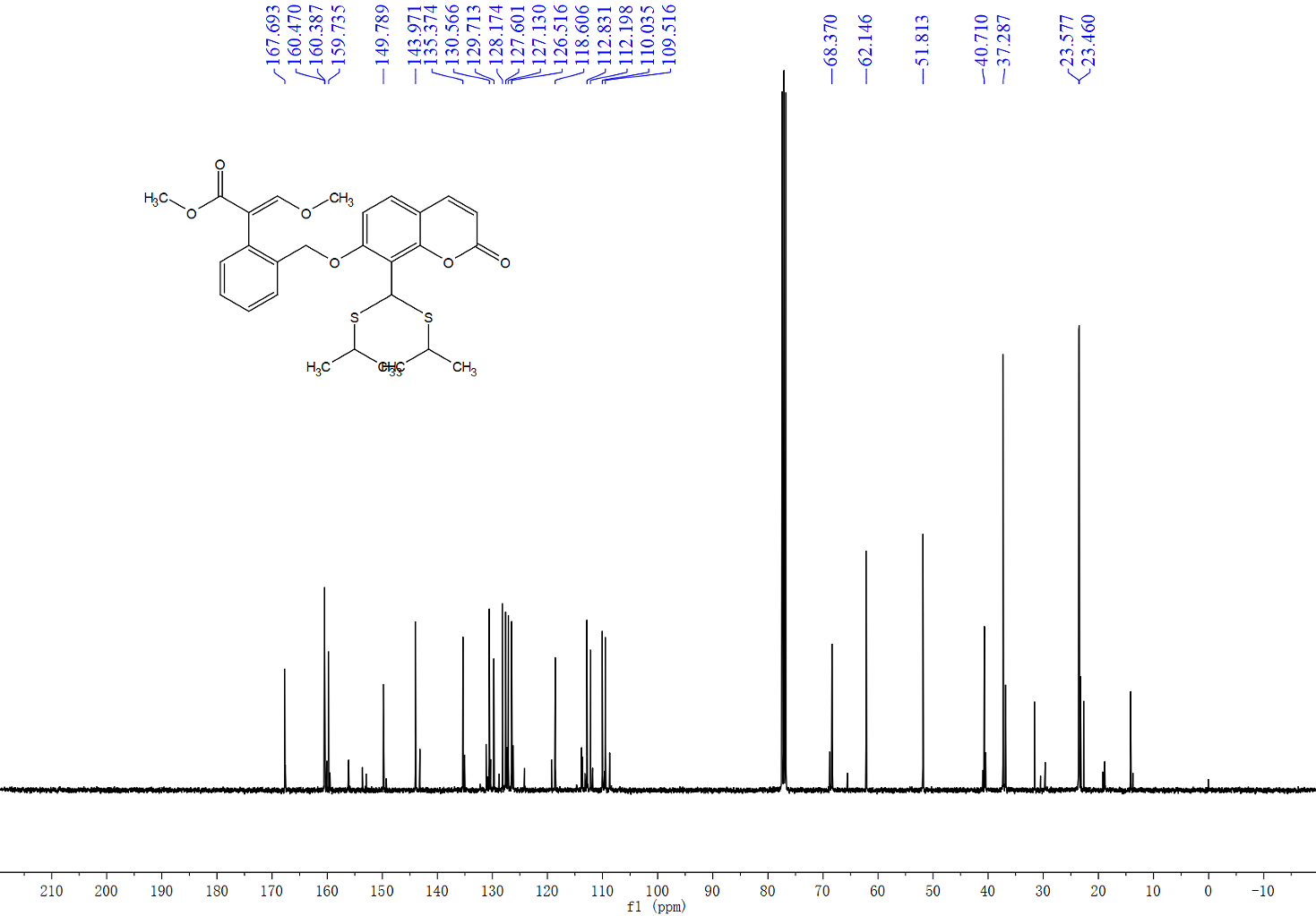


^13^C NMR of compound **D20**


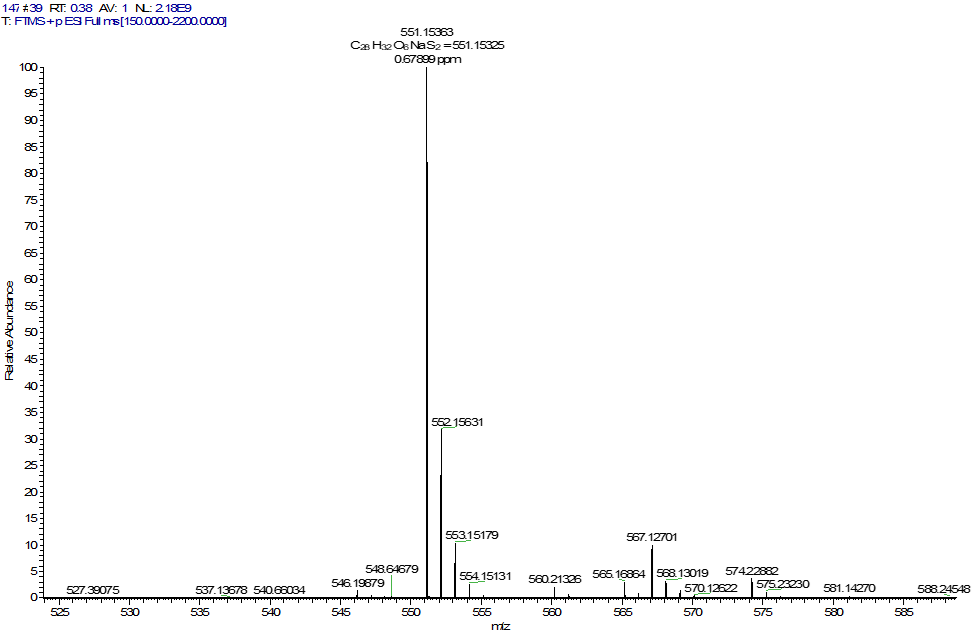


HRMS of compound **D20**


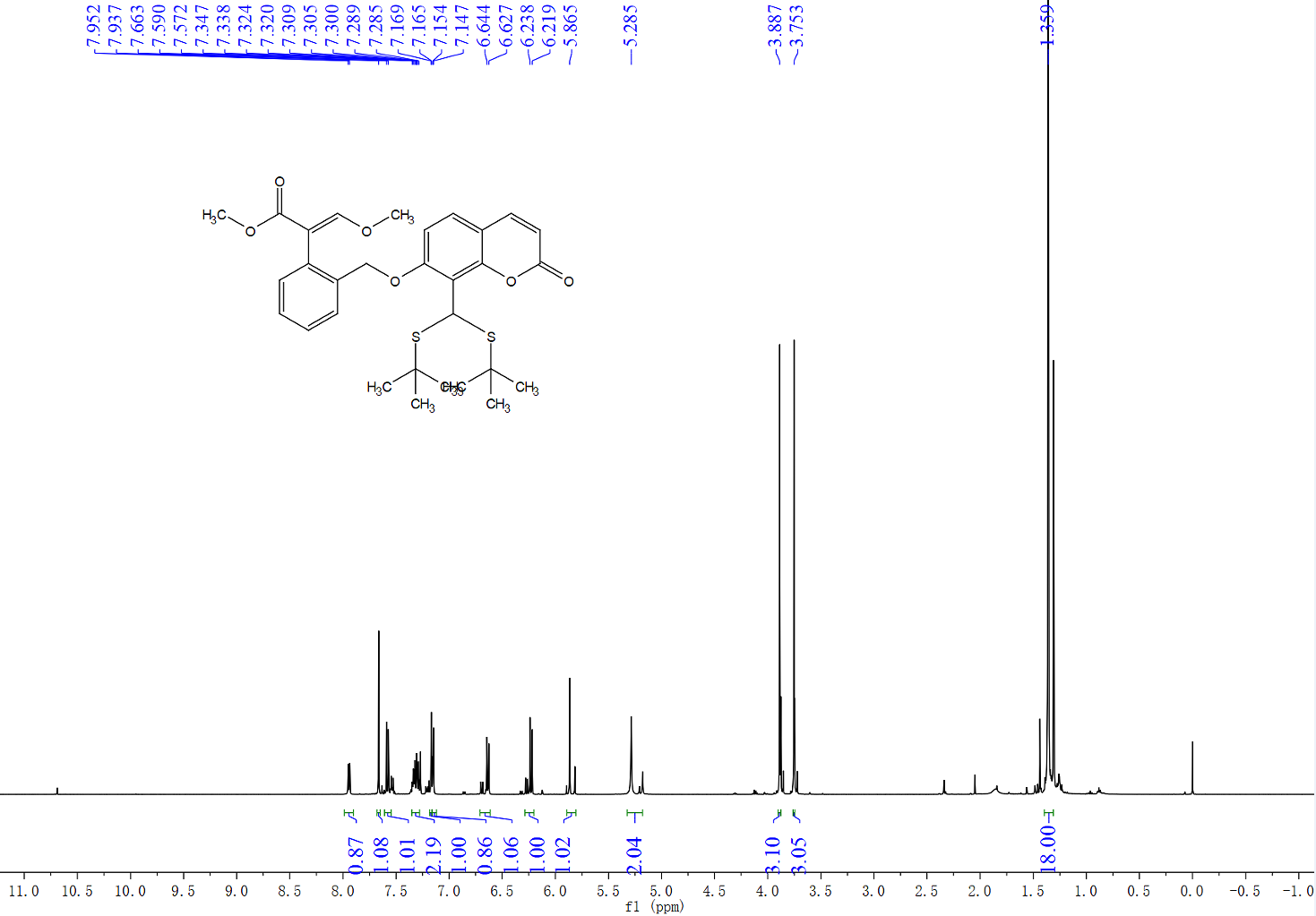


^1^H NMR of compound **D21**


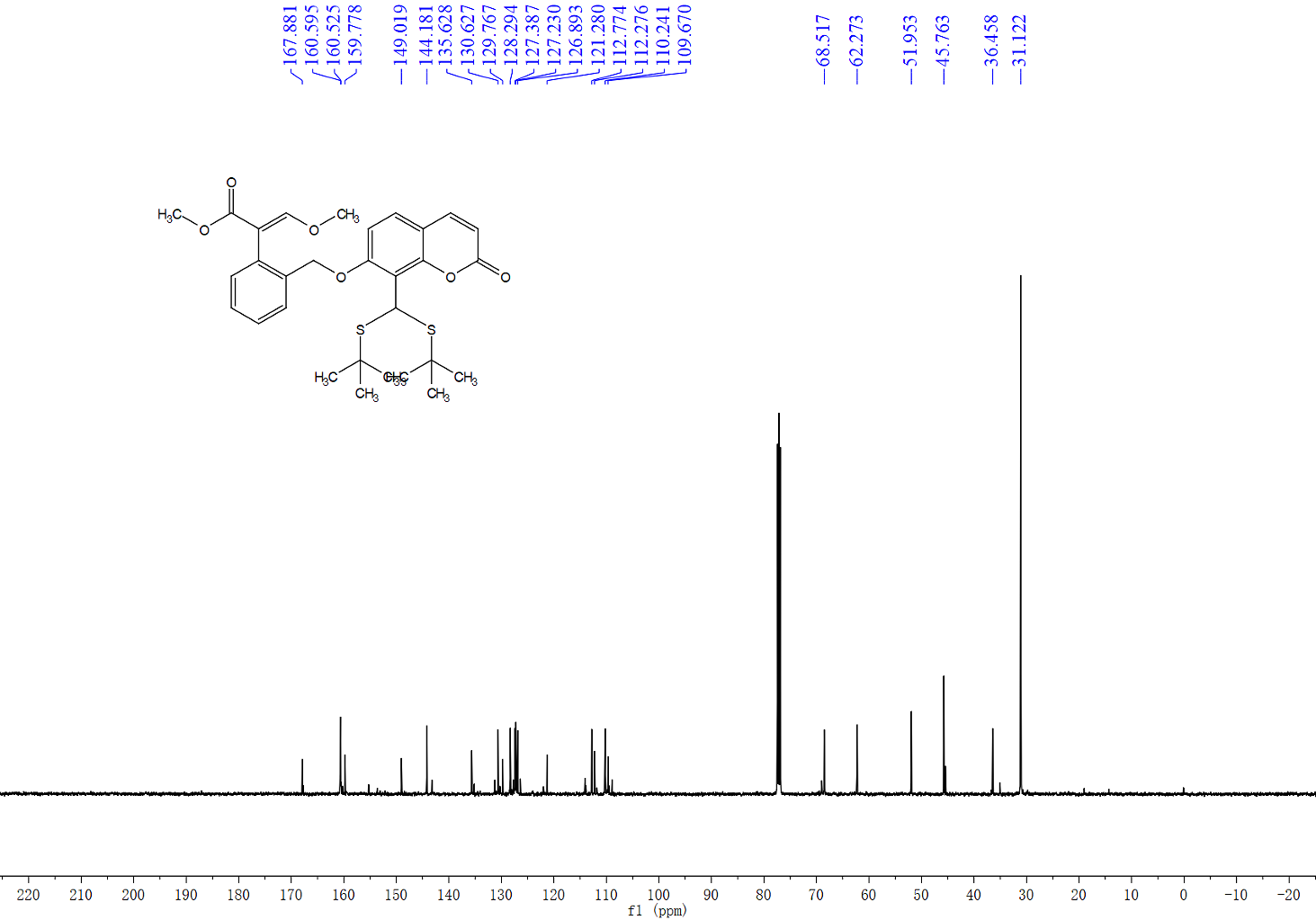


^13^C NMR of compound **D21**


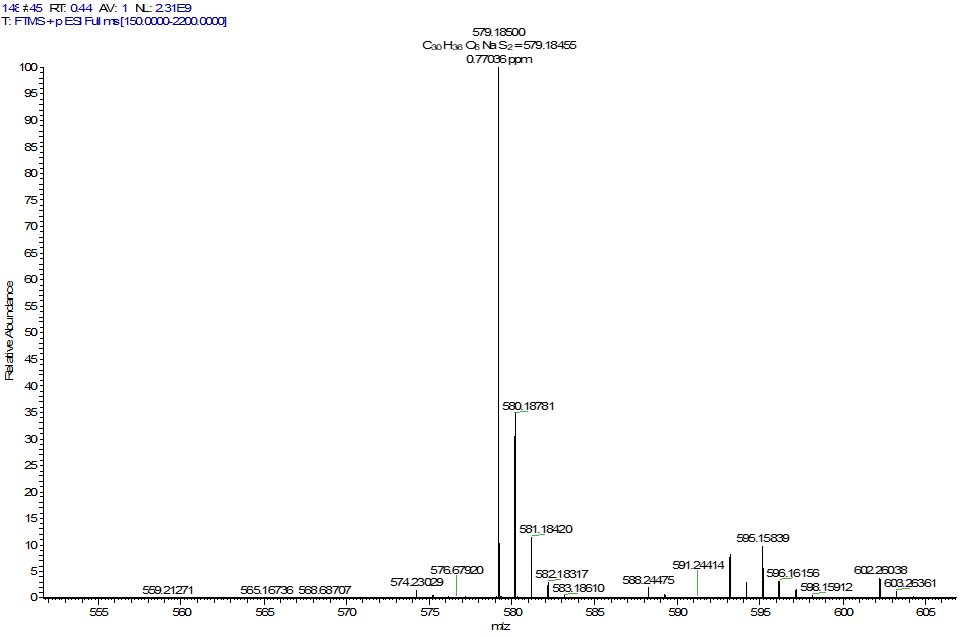


HRMS of compound **D21**


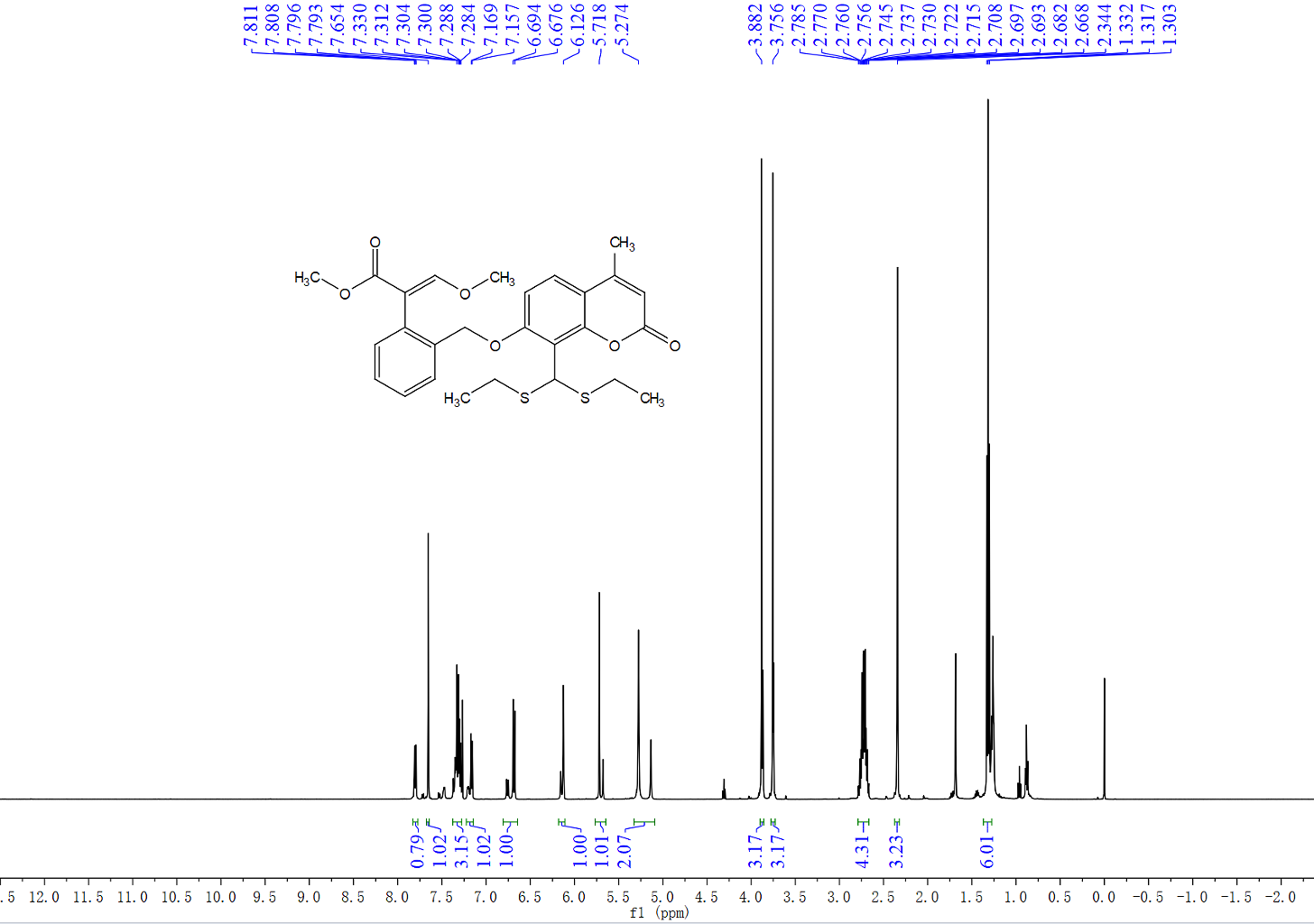


^1^H NMR of compound **D22**


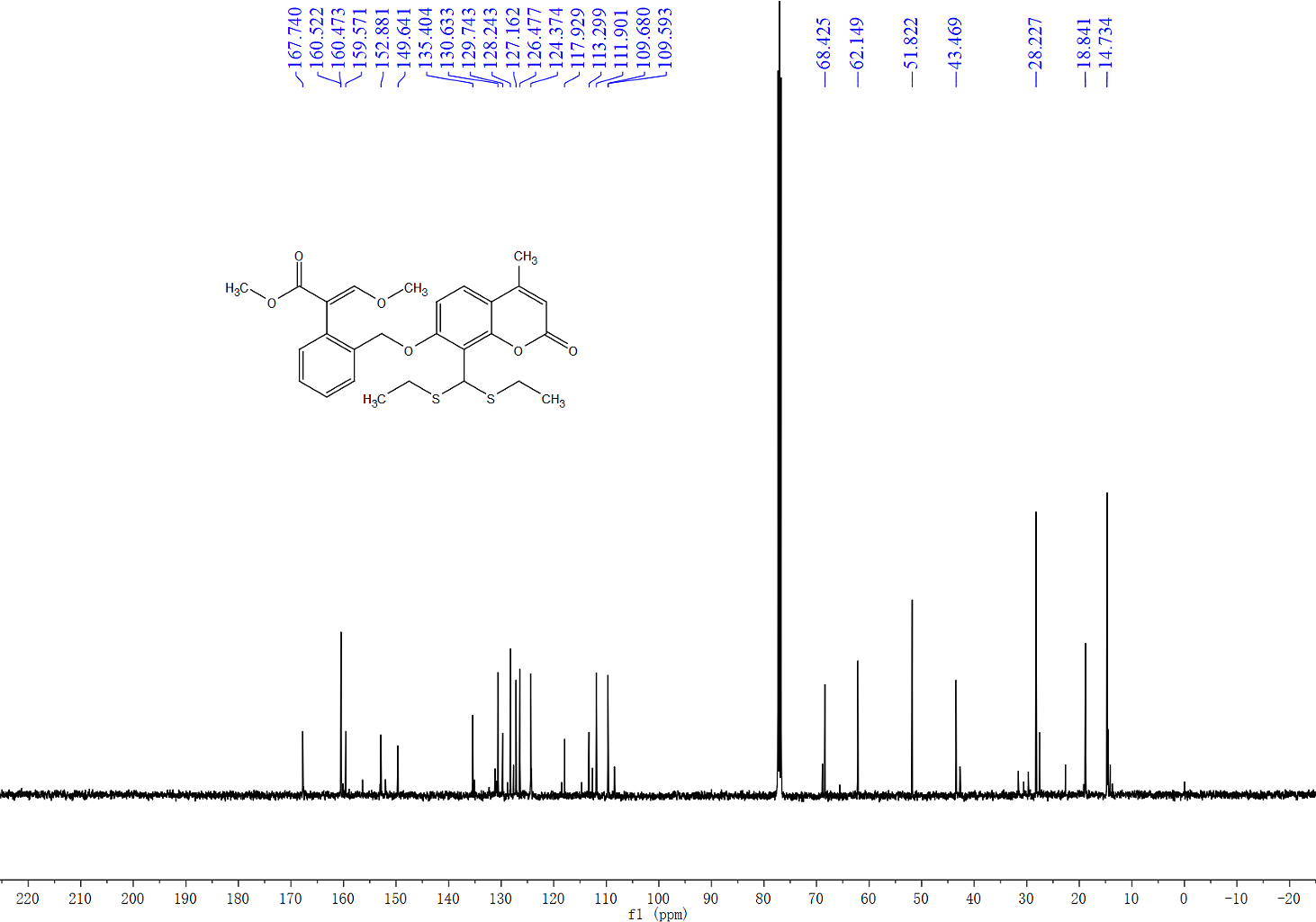


^13^C NMR of compound **D22**


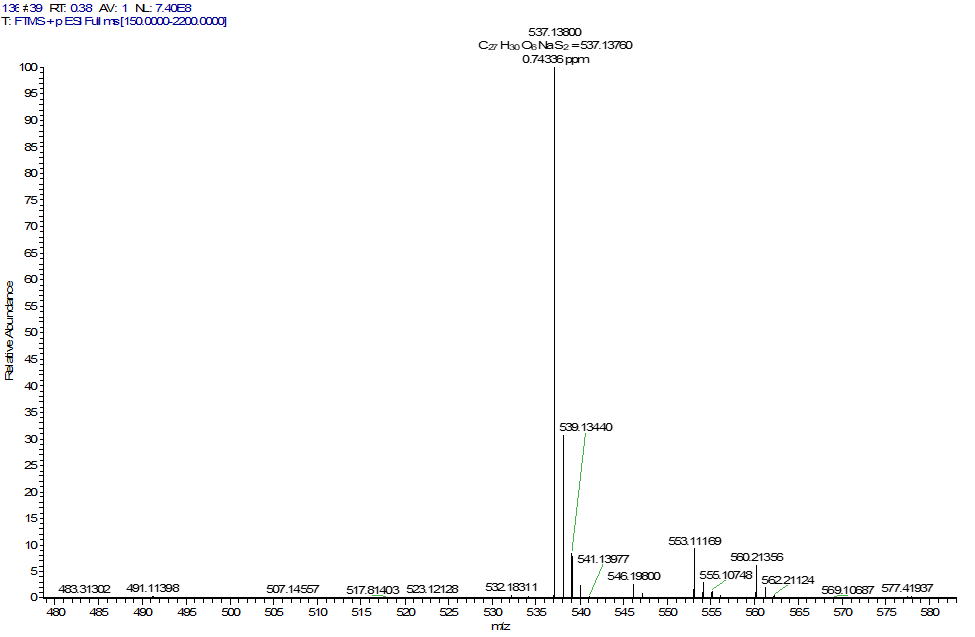


HRMS of compound **D22**


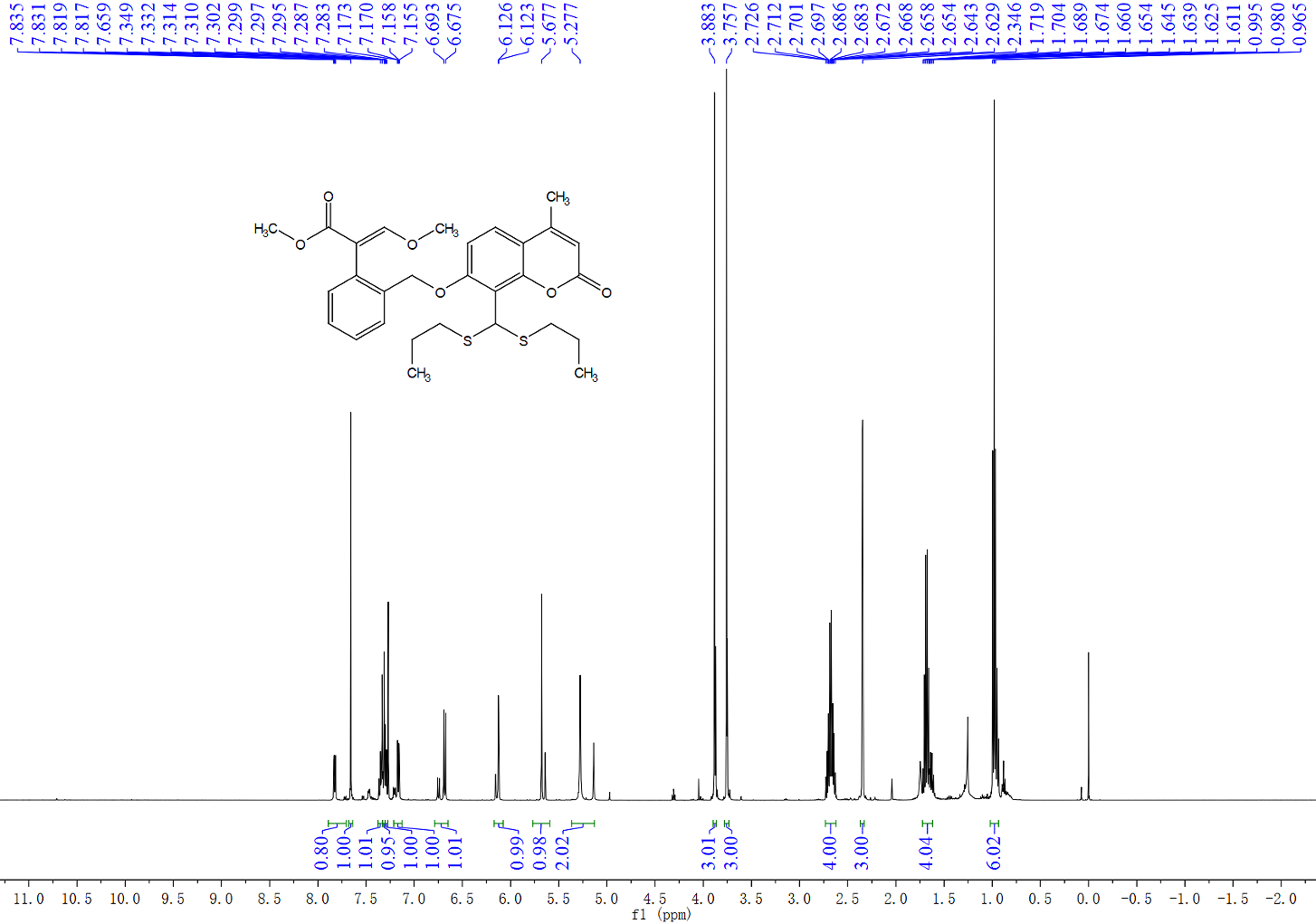


^1^H NMR of compound **D23**


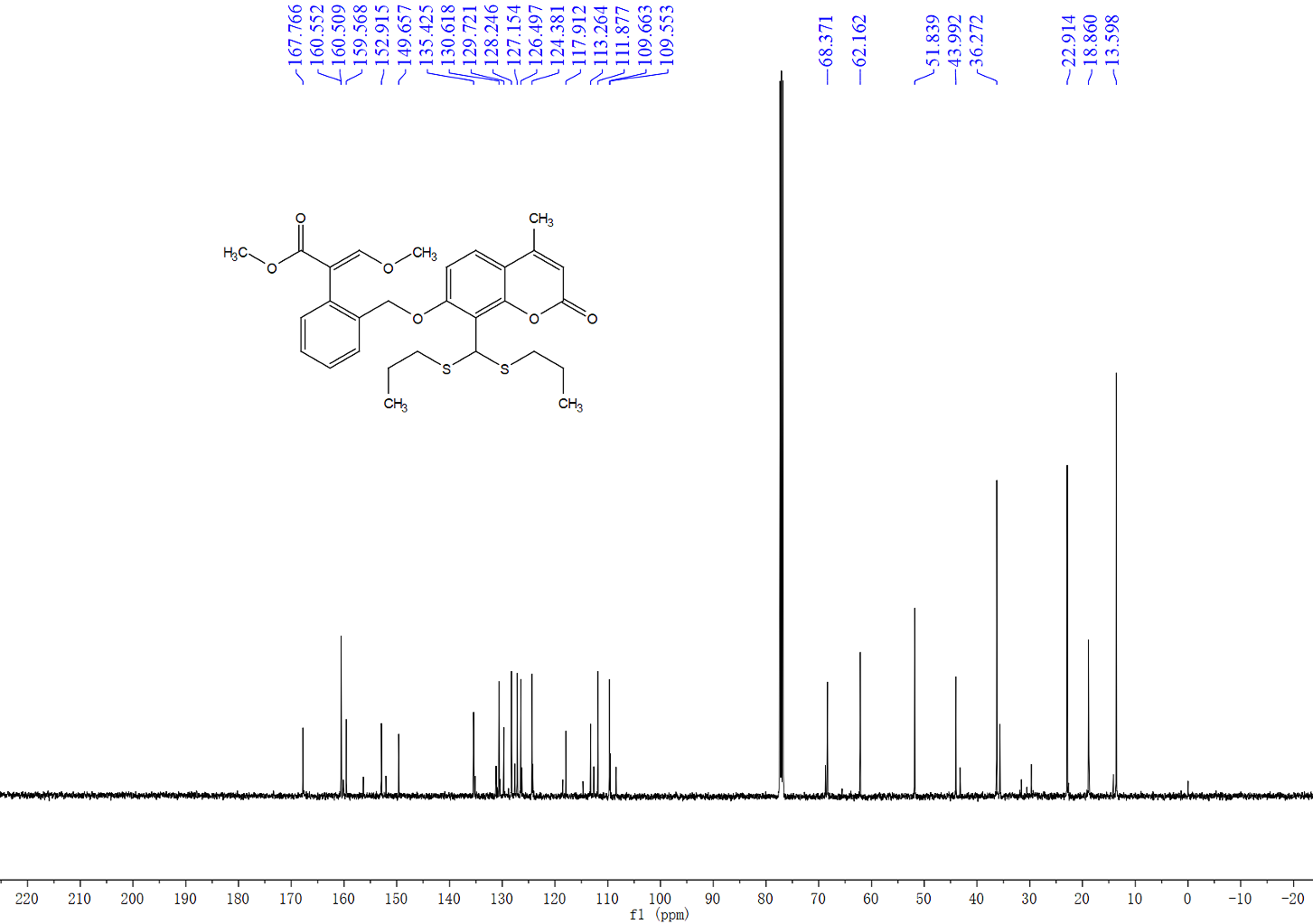


^13^C NMR of compound **D23**


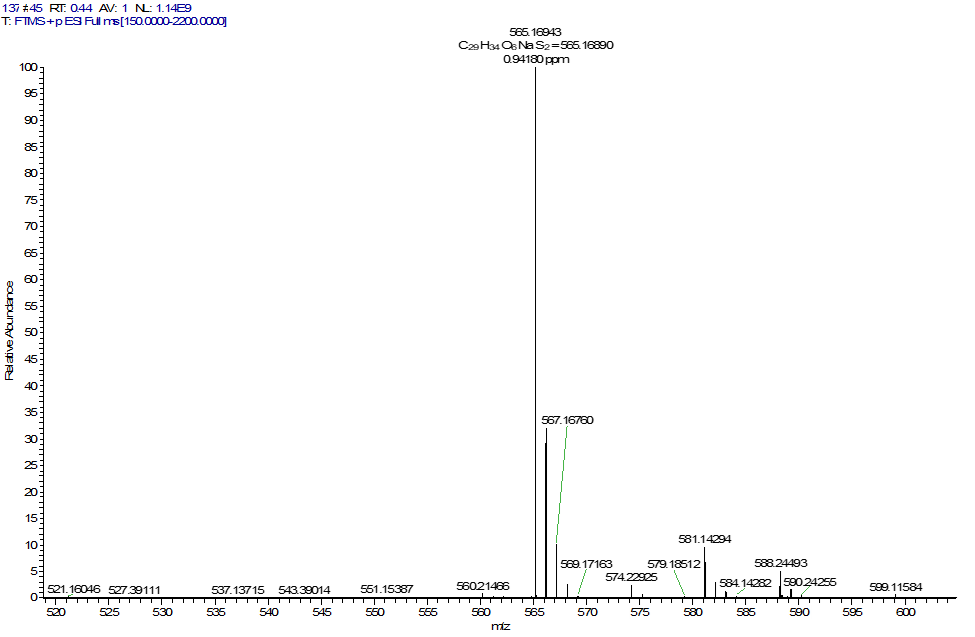


HRMS of compound **D23**


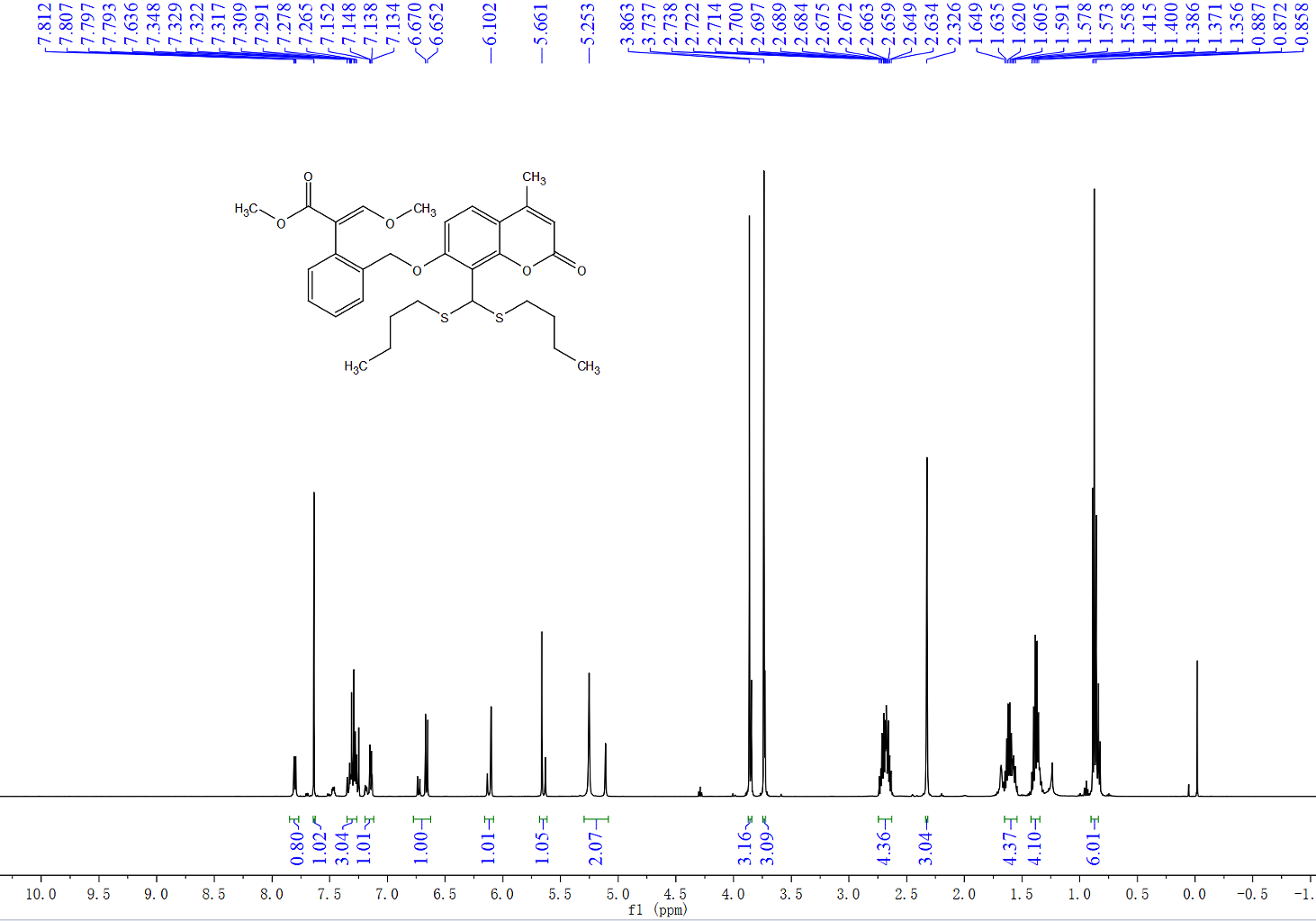


^1^H NMR of compound **D24**


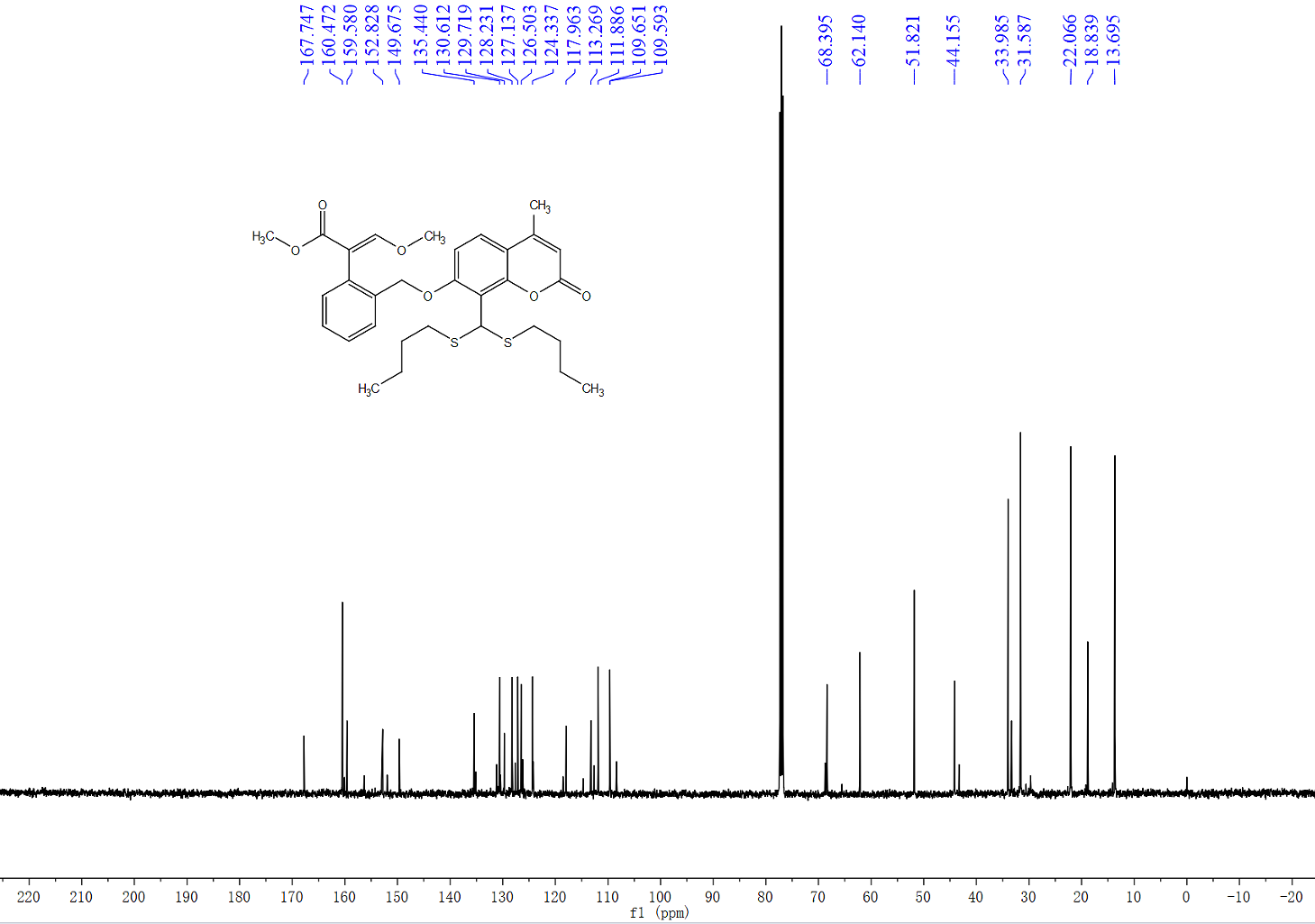


^13^C NMR of compound **D24**


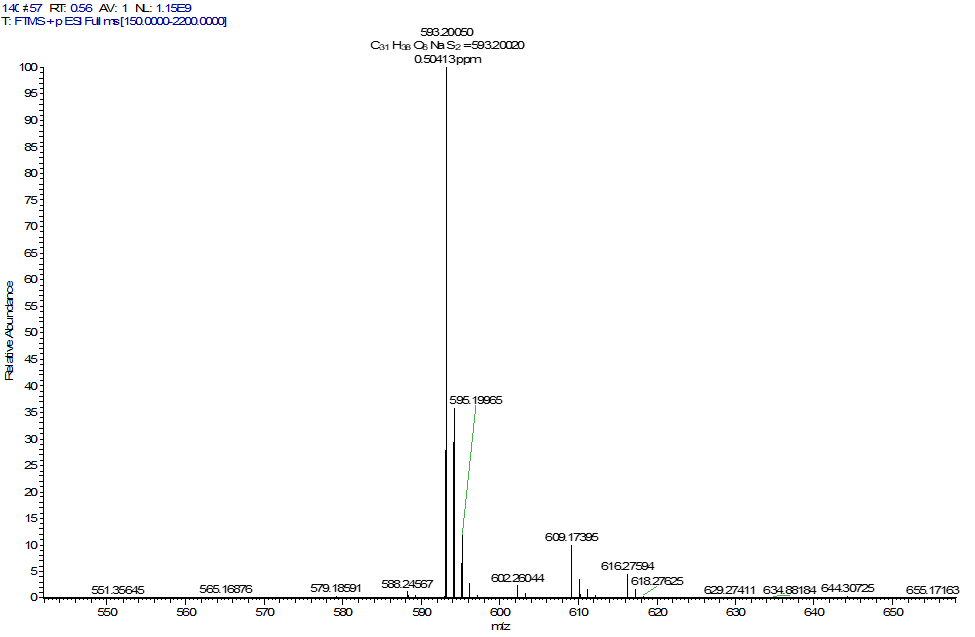


HRMS of compound **D24**


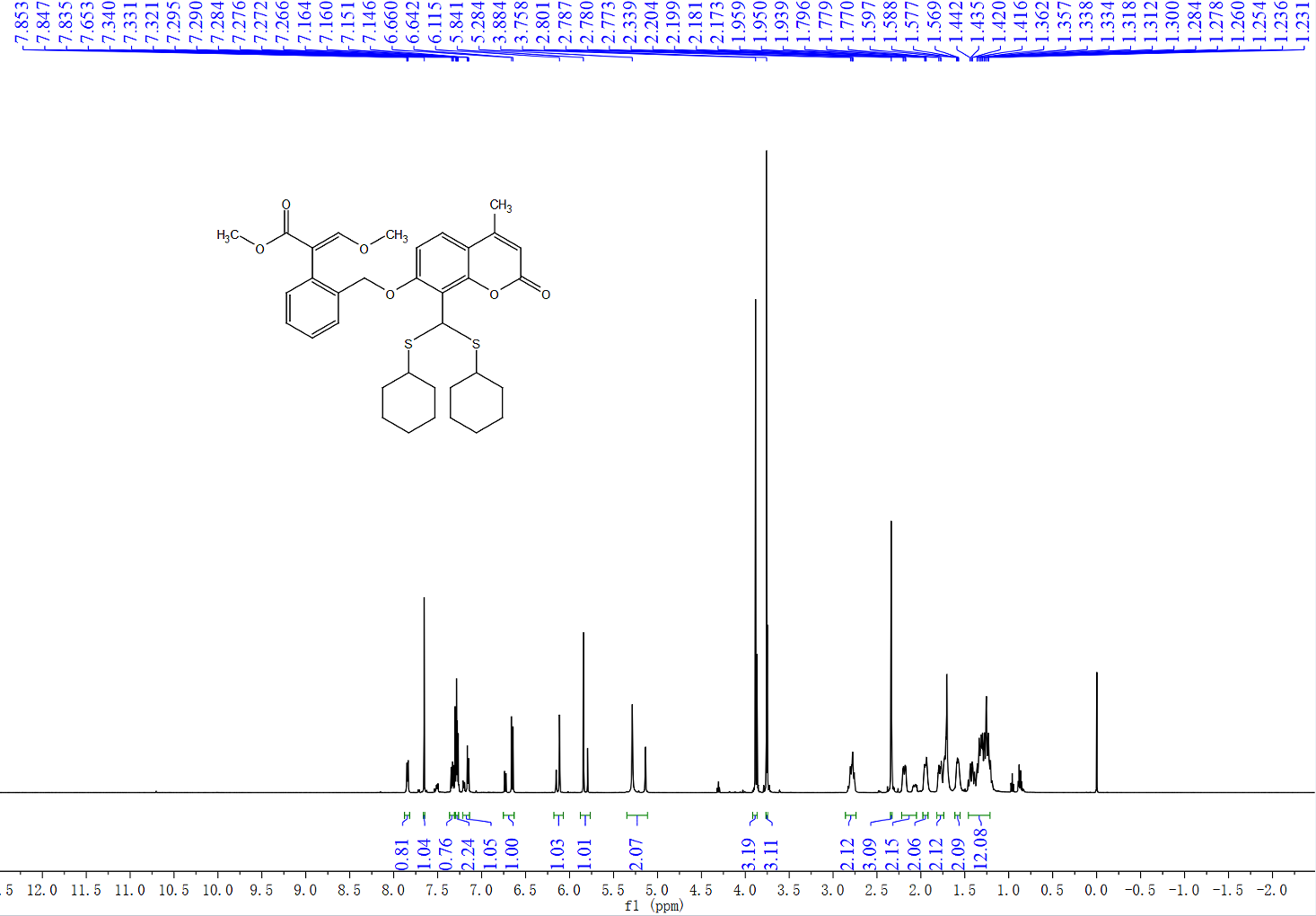


^1^H NMR of compound **D25**


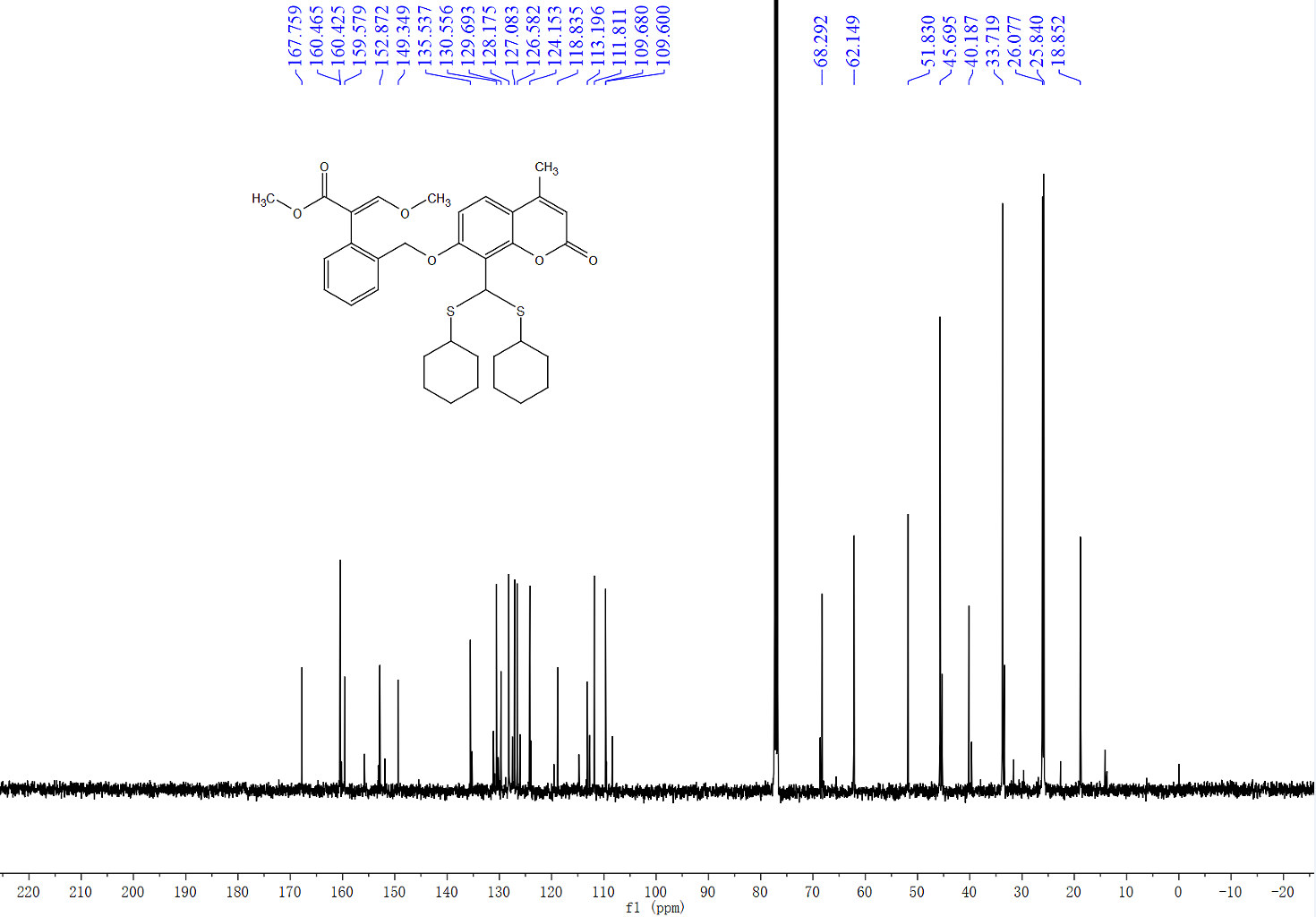


^13^C NMR of compound **D25**


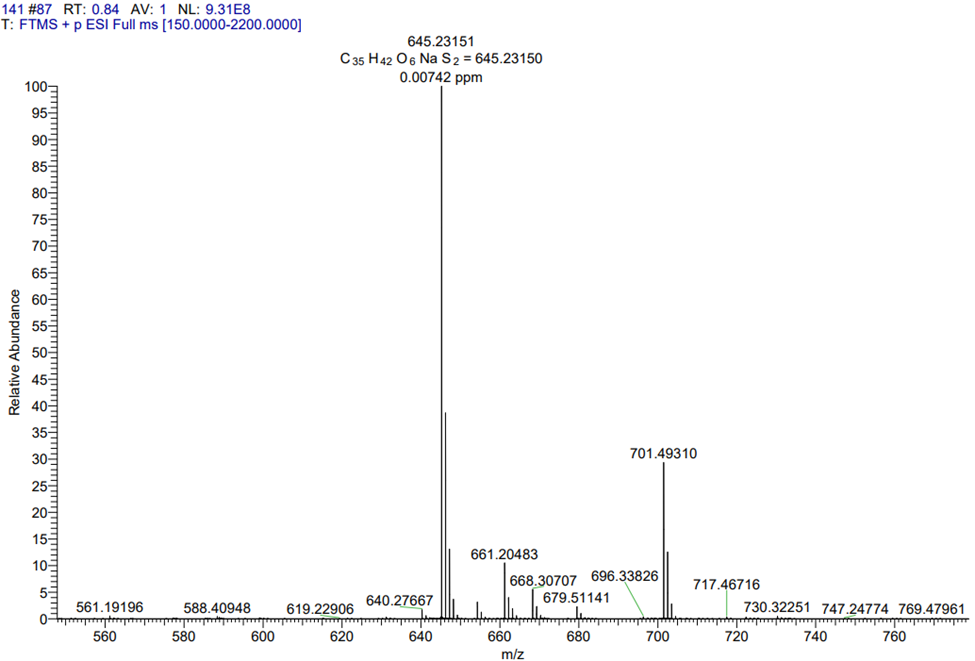


HRMS of compound **D25**


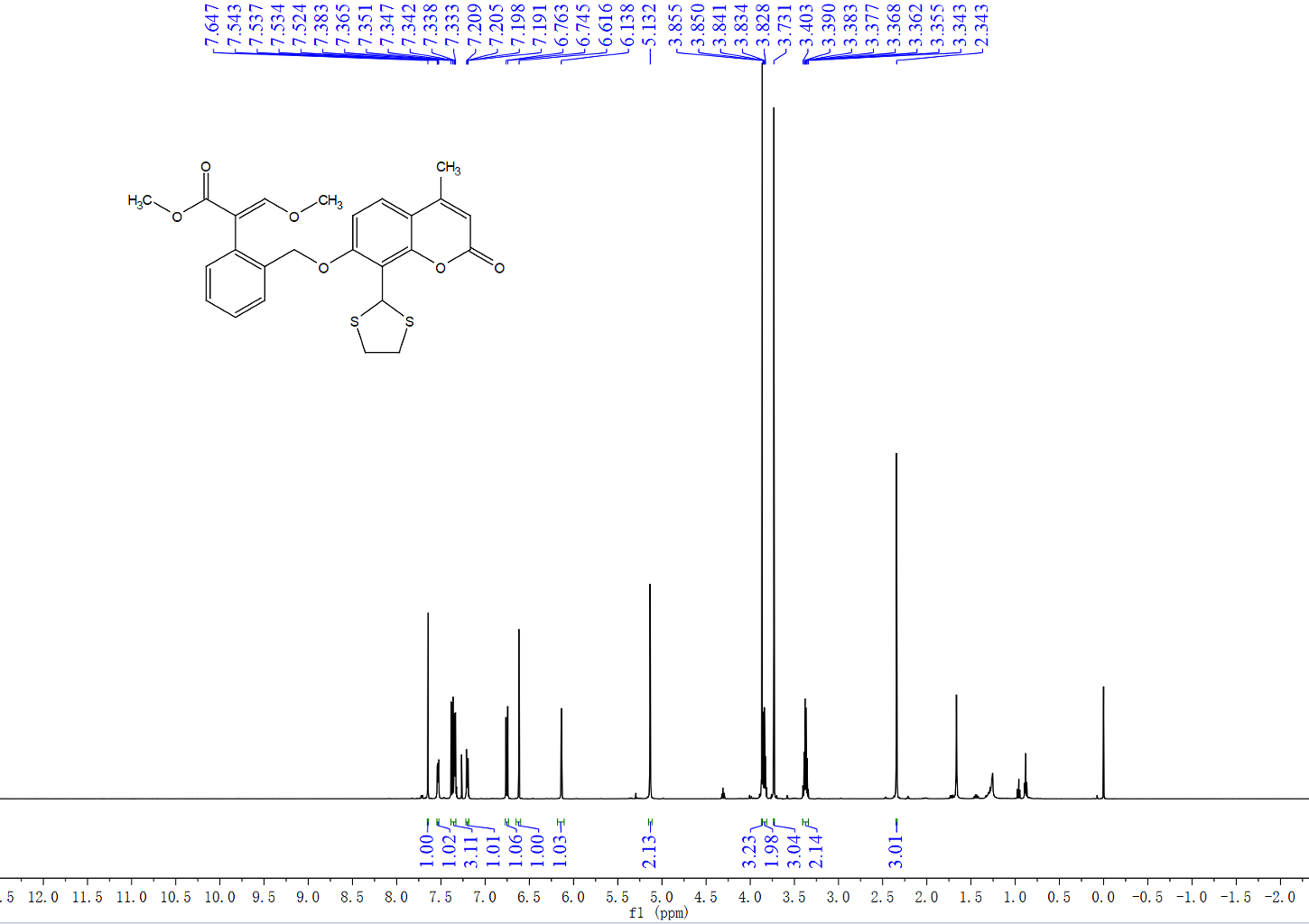


^1^H NMR of compound **D26**


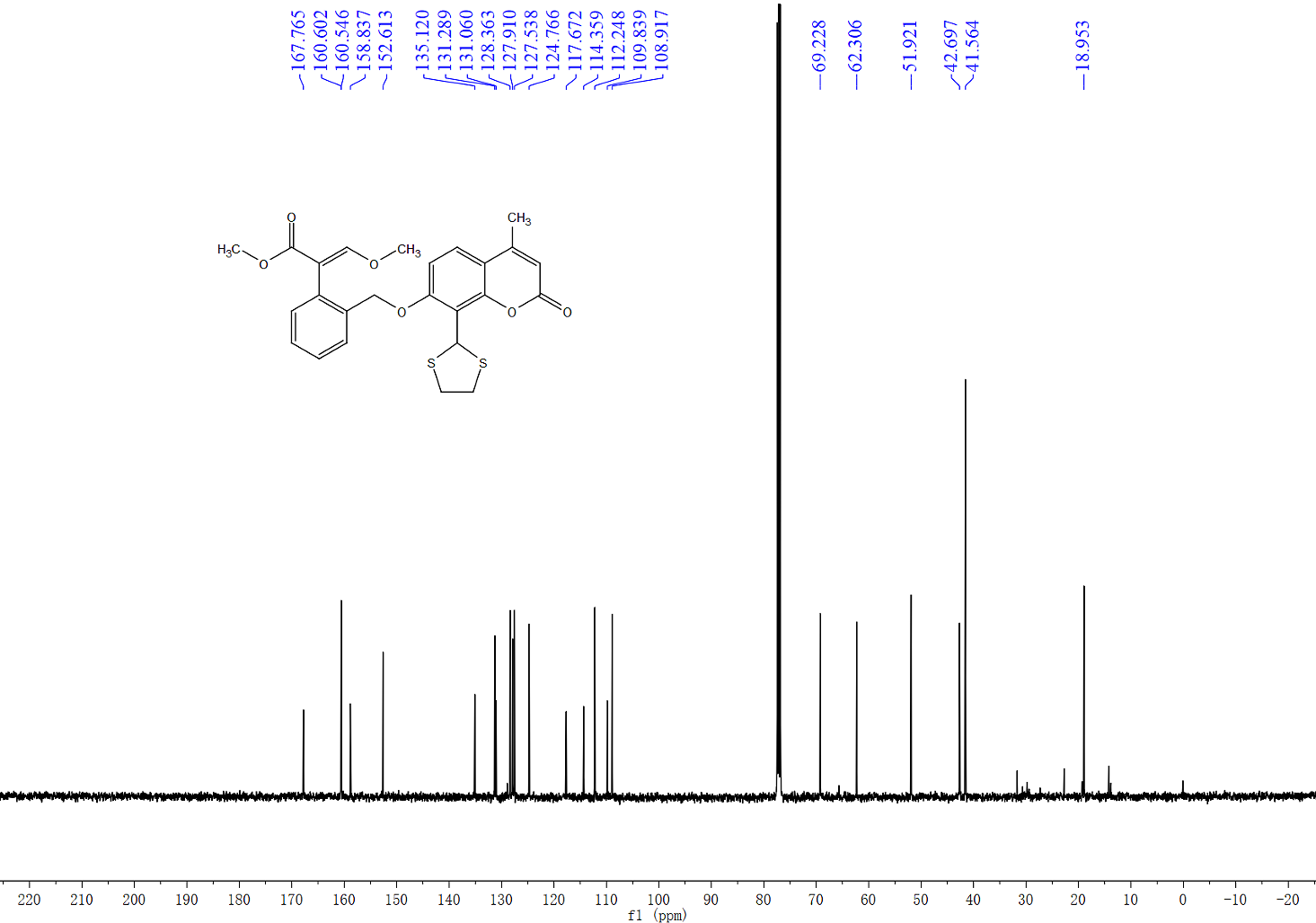


^13^C NMR of compound **D26**


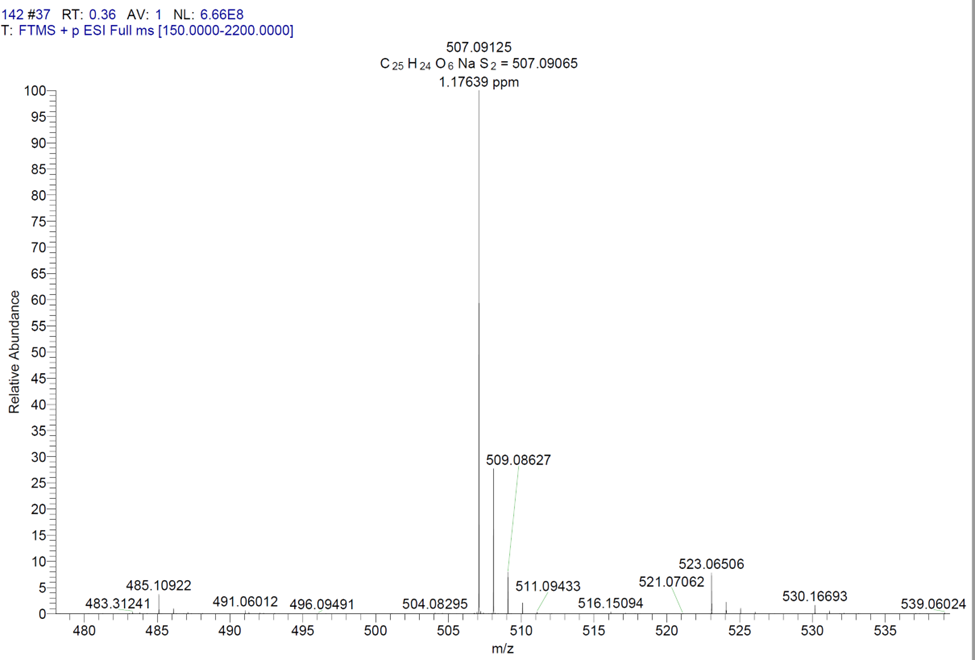


HRMS of compound **D26**


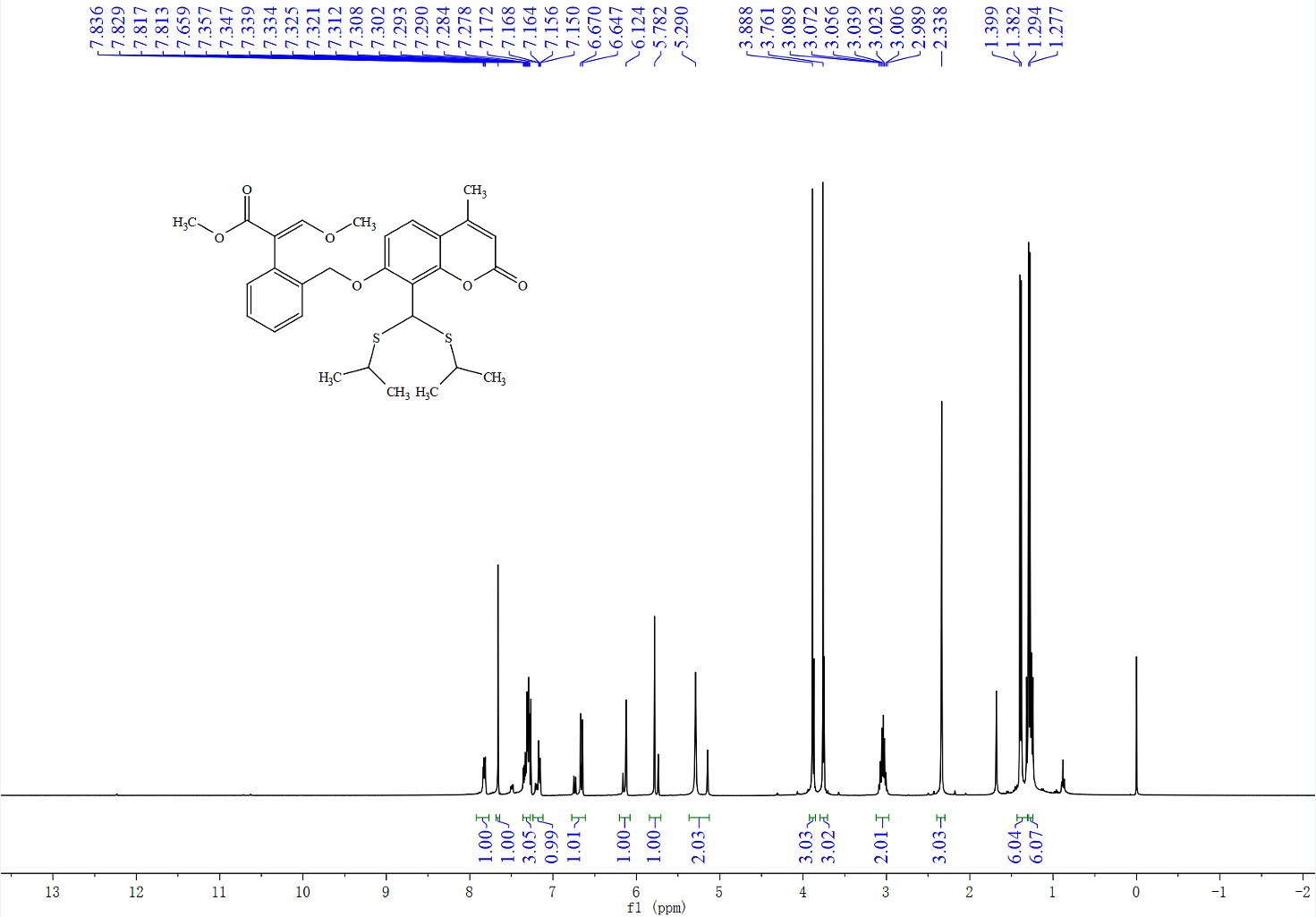


^1^H NMR of compound **D27**


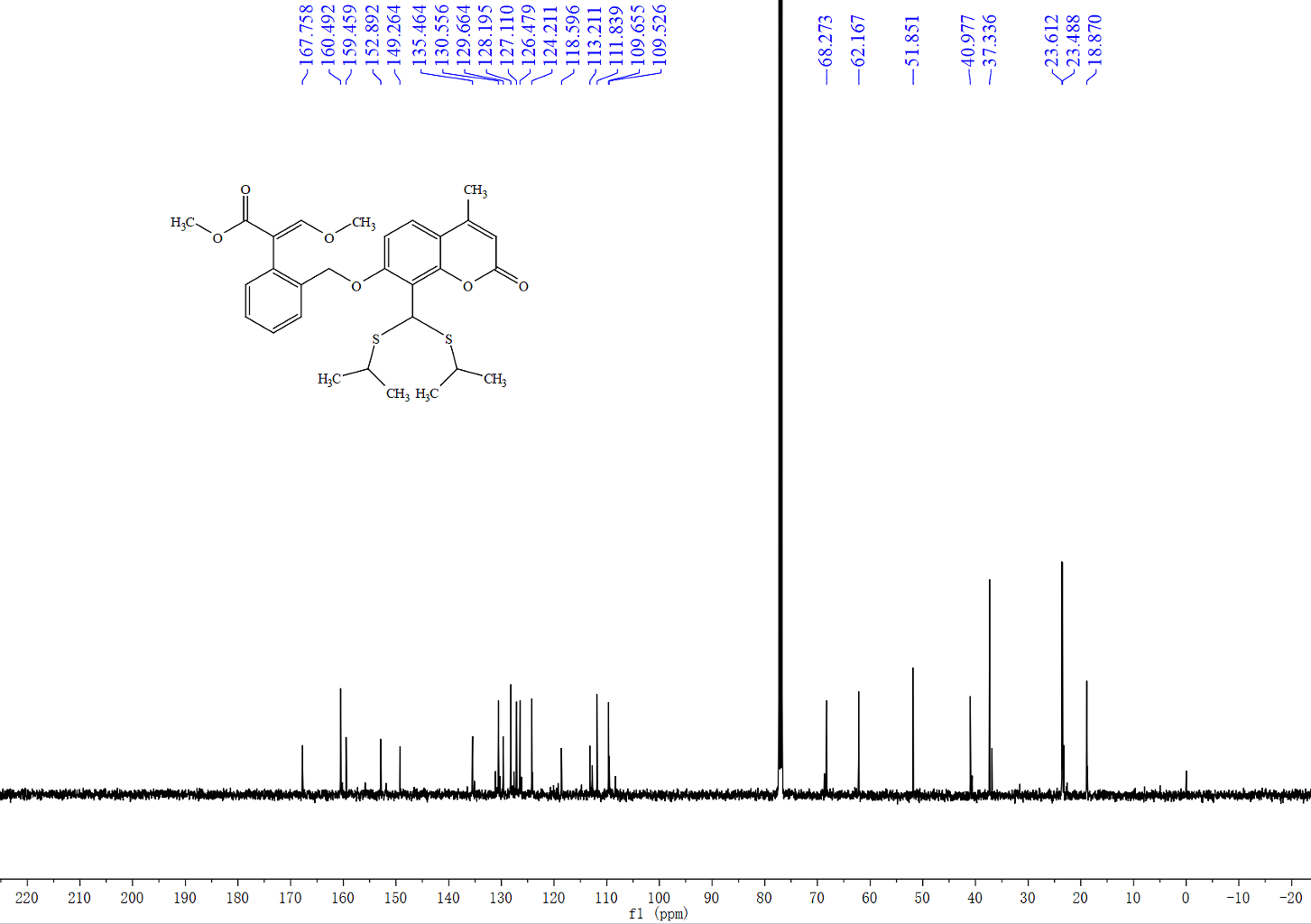


^13^C NMR of compound **D27**


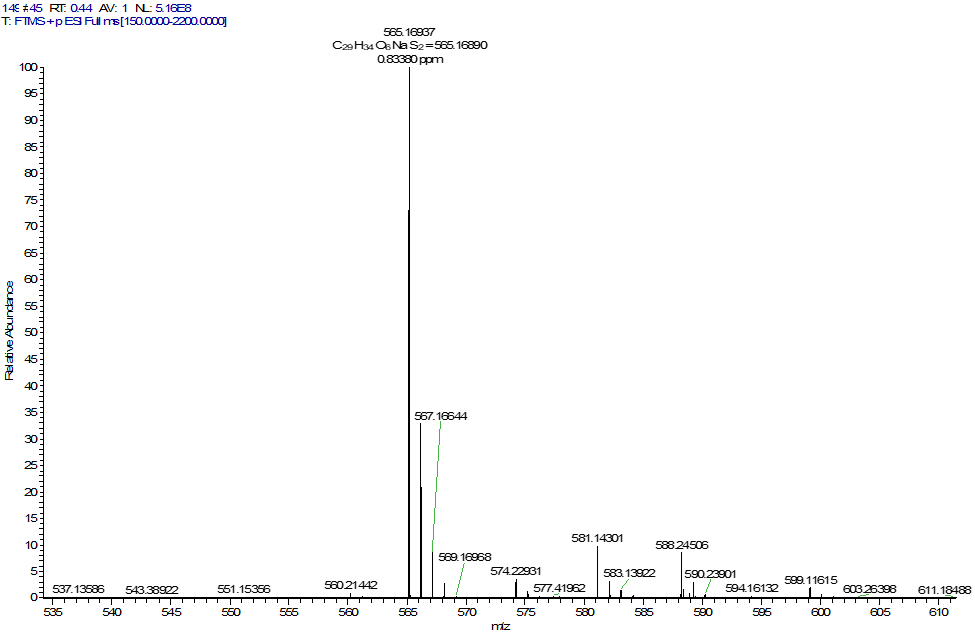


HRMS of compound **D27**


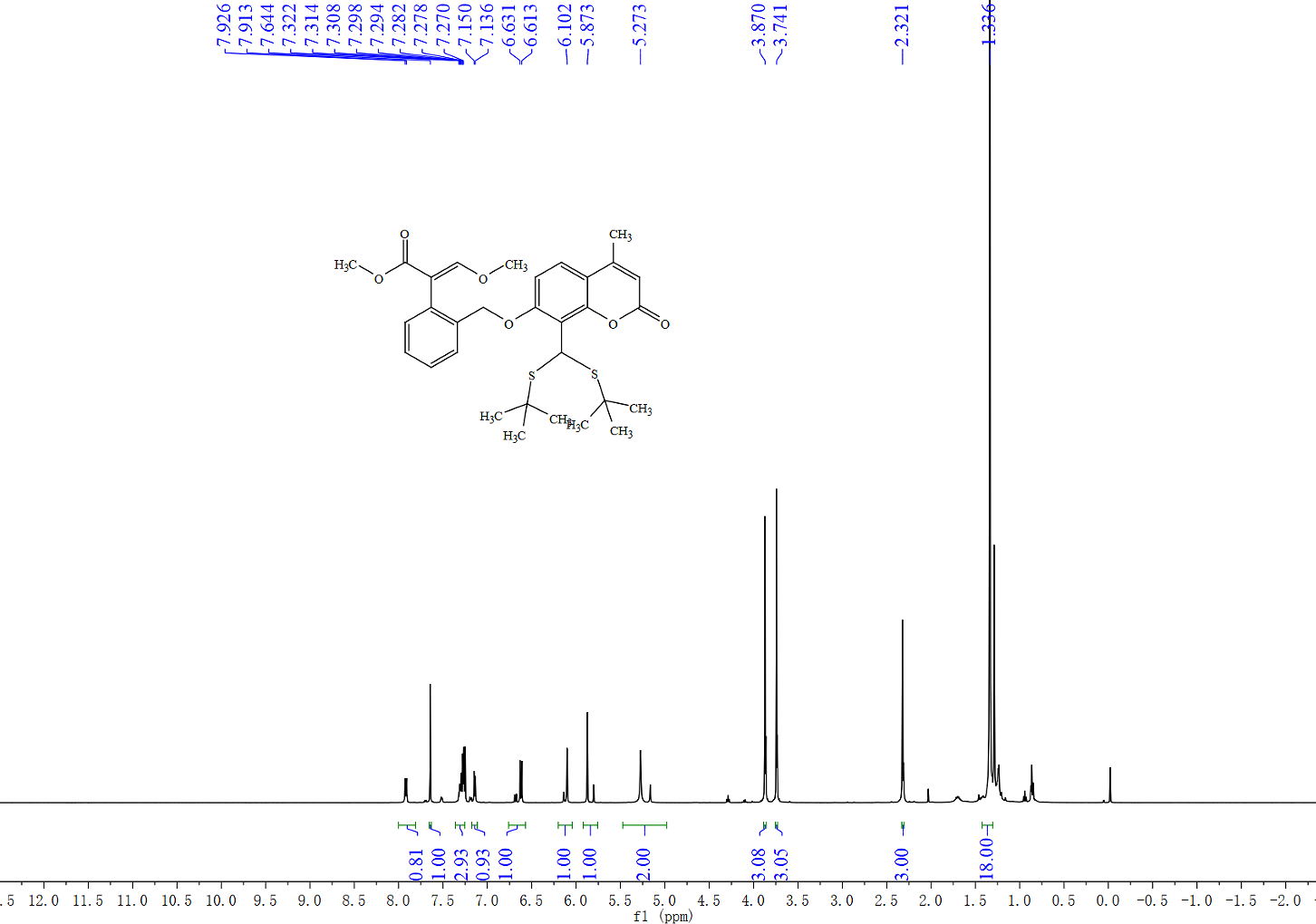


^1^H NMR of compound **D28**


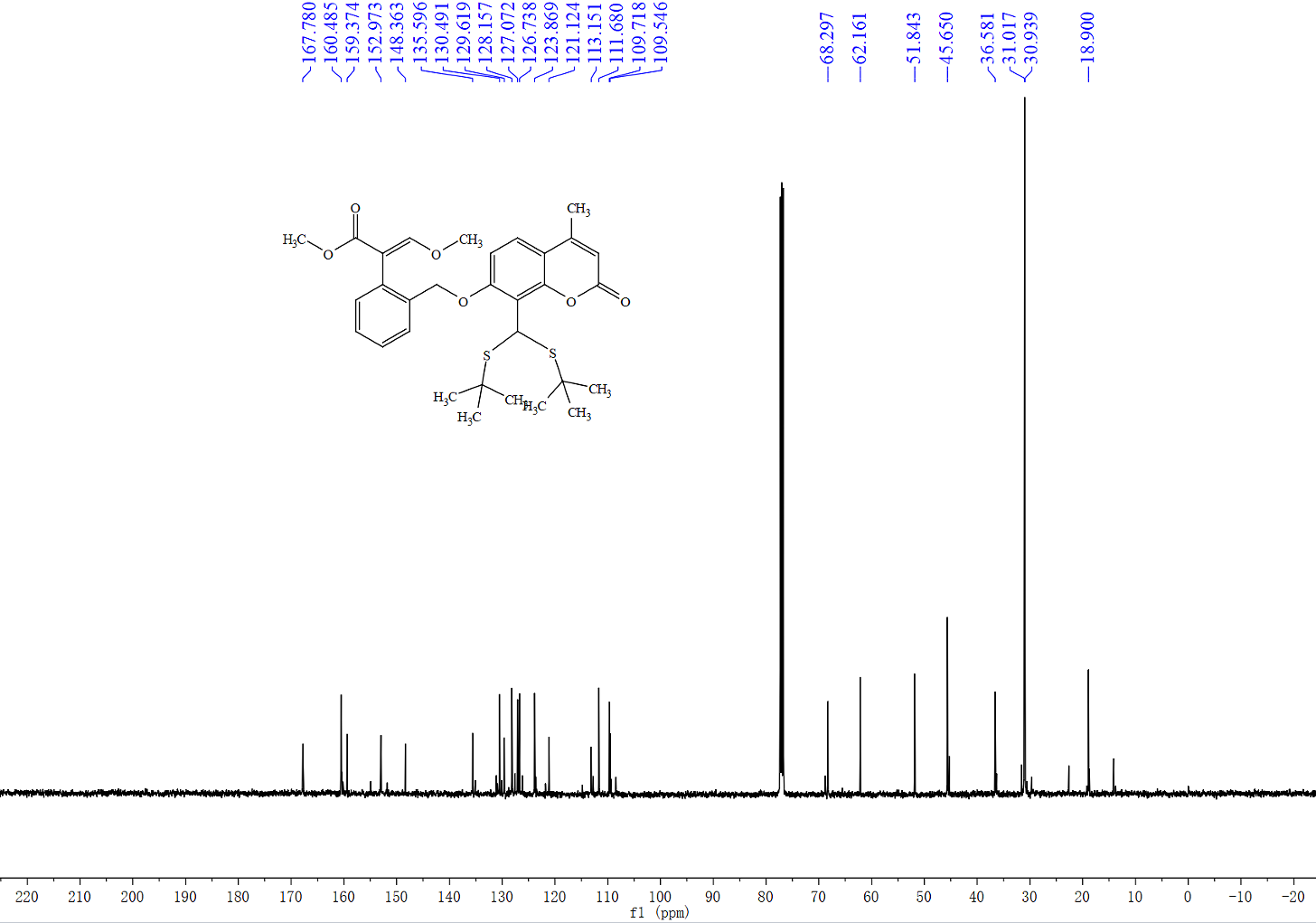


^13^C NMR of compound **D28**


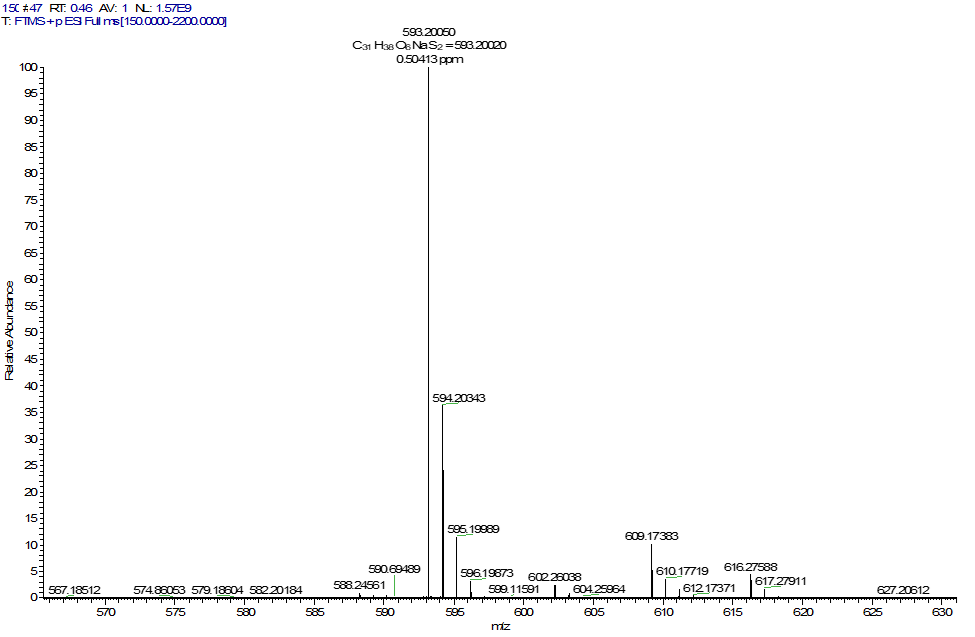


HRMS of compound **D28**


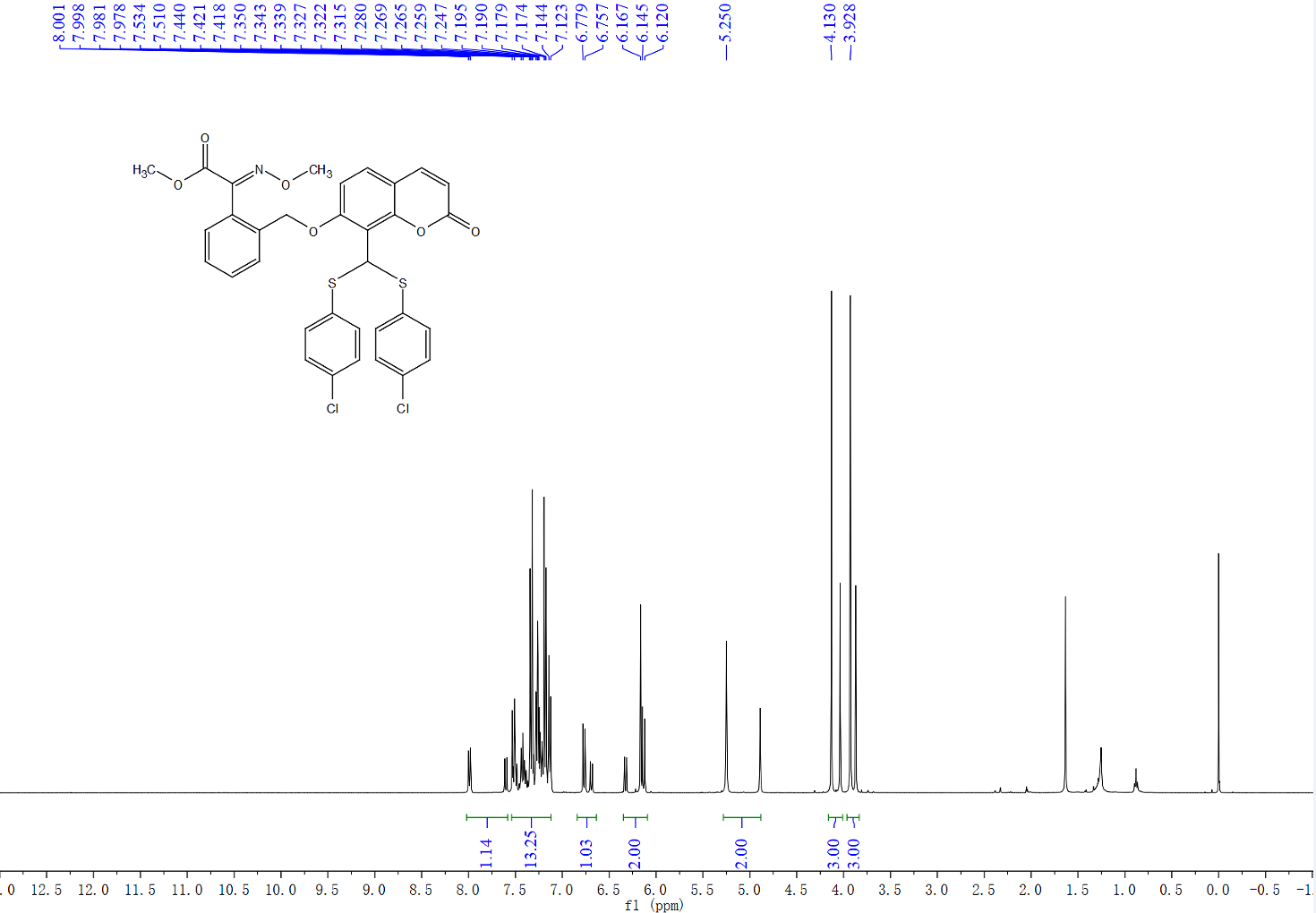


^1^H NMR of compound **D29**


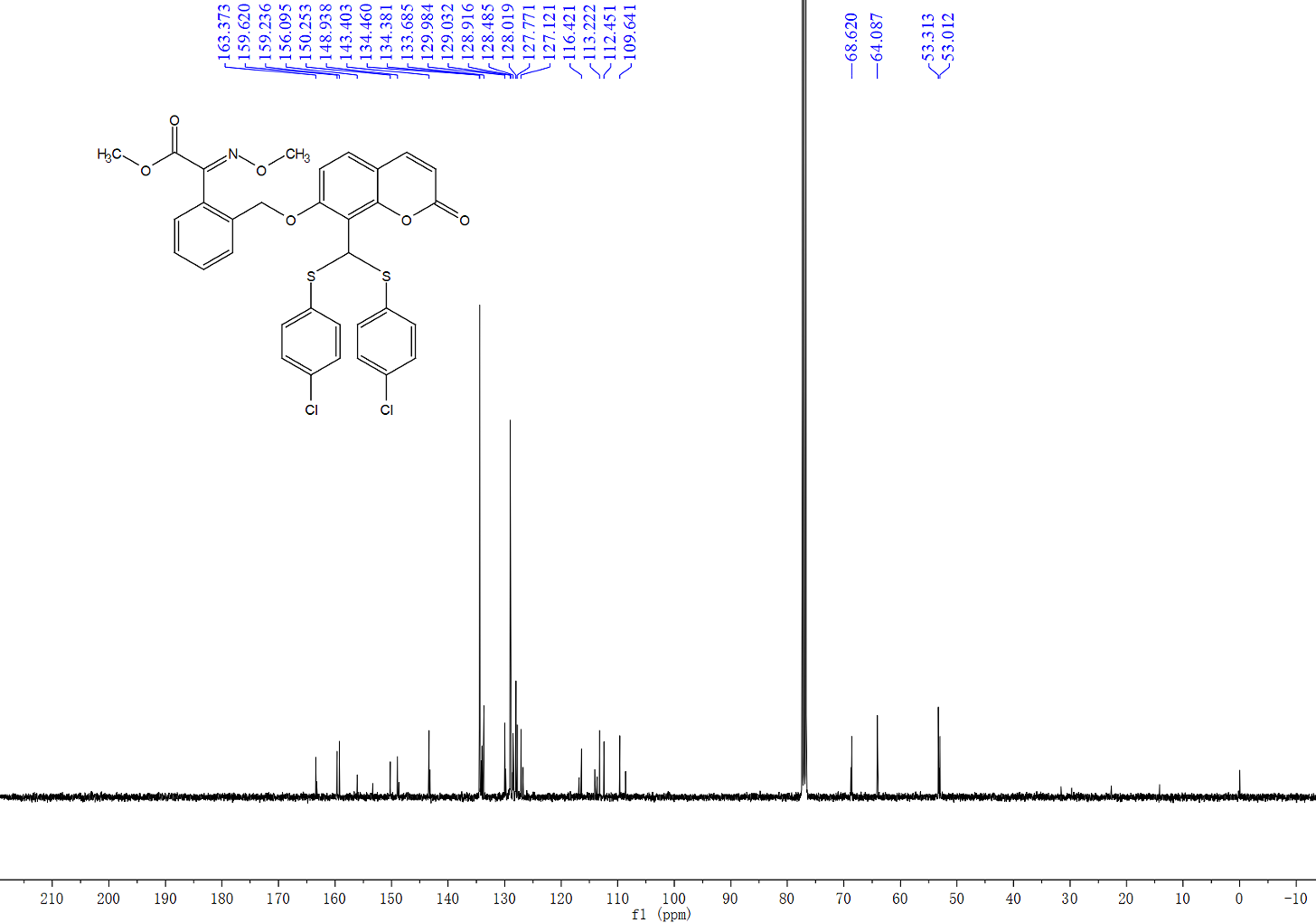


^13^C NMR of compound **D29**


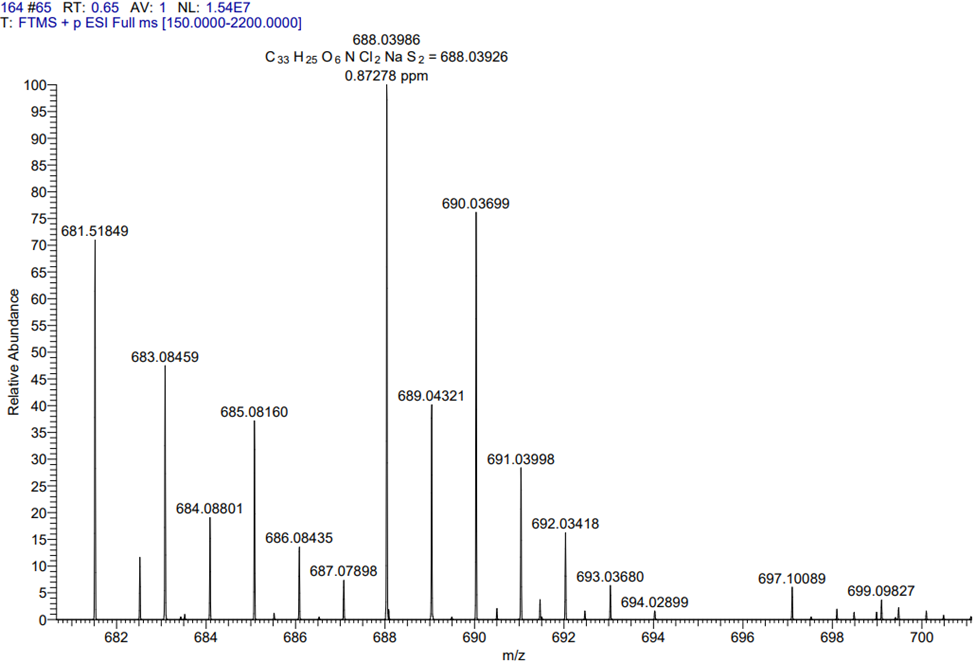


HRMS of compound **D29**


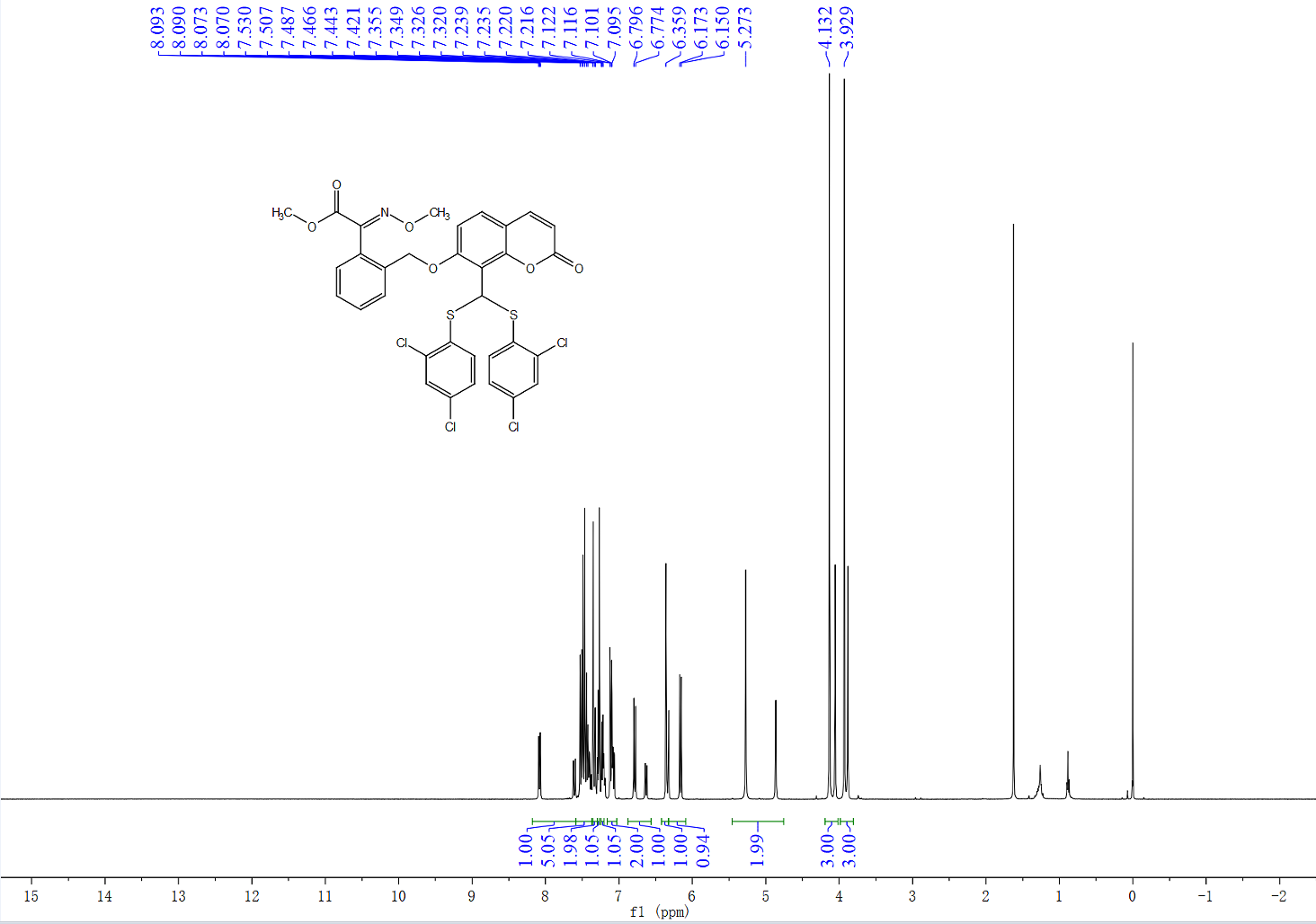


^1^H NMR of compound **D30**

^13^C NMR of compound **D30**

HRMS of compound **D30**

^1^H NMR of compound **D31**

^13^C NMR of compound **D31**

HRMS of compound **D31**

^13^C NMR of compound **D31**

^1^H NMR of compound **D32**

^13^C NMR of compound **D32**

HRMS of compound **D32**

^1^H NMR of compound **D33**

^13^C NMR of compound **D33**

HRMS of compound **D33**

^1^H NMR of compound **D34**

^13^C NMR of compound **D34**

HRMS of compound **D34**
